# From Stable PH‐Ylides to α‐Carbanionic Phosphines as Ligands for Zwitterionic Catalysts

**DOI:** 10.1002/anie.202203950

**Published:** 2022-06-14

**Authors:** Jana‐Alina Zur, Michelle Schmidt, Kai‐Stephan Feichtner, Prakash Duari, Julian Löffler, Thorsten Scherpf, Viktoria H. Gessner

**Affiliations:** ^1^ Chair of Inorganic Chemistry II Faculty of Chemistry and Biochemistry Ruhr-University Bochum Universitätsstrasse 150 44780 Bochum Germany

**Keywords:** Alkali Metals, Carbanions, Homogeneous Catalysis, Phosphine Ligands, Ylides

## Abstract

Although ylides are commonly used reagents in organic synthesis, the parent methylphosphine MePH_2_ only exists in its phosphine form in the condensed phase. Its ylide tautomer H_3_P^+^−CH_2_^−^ is considerably higher in energy. Here, we report on the formation of bis(sulfonyl)methyl‐substituted phosphines of the type (RO_2_S)_2_C(H)−PR_2,_ which form stable PH ylides under ambient conditions, amongst the first examples of an acyclic phosphine which only exists in its PH ylide form. Depending on the exact substitution pattern the phosphines form an equilibrium between the PH ylide and the phosphine form or exist as one of both extremes. These phosphines were found to be ideal starting systems for the facile formation of α‐carbanionic phosphines. The carbanion‐functionalization leads to a switch from electron‐poor to highly electron‐rich phosphines with strong donor abilities and high basicities. Thus, the phosphines readily react with different electrophiles exclusively at the phosphorus atom and not at the carbanionic center. Furthermore, the anionic nature of the phosphines allows the formation of zwitterionic complexes as demonstrated by the isolation of a gold(I) complex with a cationic metal center. The cationic gold center allows for catalytic activity in the hydroamination of alkyne without requiring a further activation step.

## Graphical abstract

PH ylides such as H_2_C=PH_3_ are considerably higher in energy than their corresponding phosphines H_3_C−PH_2_. Using bis(sulfonyl)methyl substituents, the first PH ylides stable at ambient conditions could be isolated in solution and in the solid state. They are ideal precursors to carbanionic phosphines with exclusive phosphine reactivity and thus to highly electron‐rich phosphines.

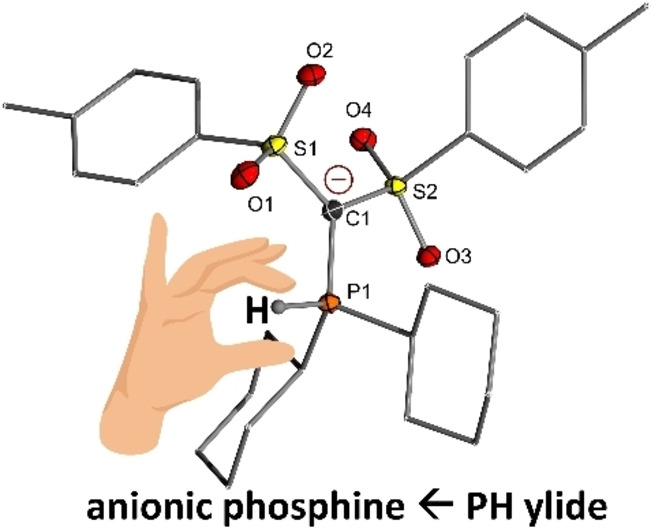

## Introduction

**Figure 1:**
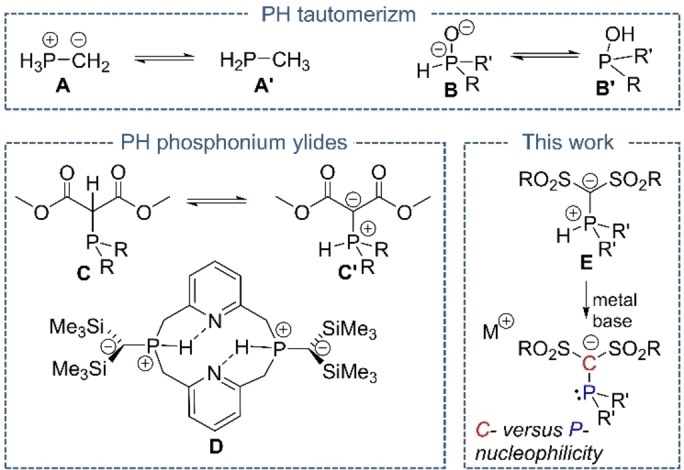
Top: Examples of PH tautomerism. Bottom: Examples of stable PH‐phosphonium ylides (left) and molecules focused on in this work (right).

Phosphorus‐ylides are versatile, routinely used reagents in organic synthesis. The structure of ylides has been discussed repeatedly in the literature but is nowadays accepted to exhibit a dipolar P−C linkage with a carbanionic site, which for simplicity reasons is still often depicted as a P=C double bond. The simplest ylide, H_3_P^+^−CH_2_^−^ (**A**) has been the subject of many theoretical as well as experimental studies and was shown to be more than 200 kJ mol^−1^ less stable than its tautomer, methylphosphine (**A′**, Figure 1).^[1]^ This is in contrast to the oxygen analogue, phosphinous acid H_2_POH. While the acid form **B′** is still slightly favored over the ylidic tautomer **B** for the parent acid, organic derivatives preferentially exist—also due to the more stable P=O bond—as the corresponding phosphine oxides (**B**).^[2]^ In case of the carbon compounds, PH‐phosphonium ylides are usually thermodynamically less stable than the corresponding alkyl phosphines and only very few examples have been isolated. The first detection of a PH ylide at ambient conditions was reported by Kolodiazhny in the 1980s by means of bis(alkoxycarbonyl)methylphosphines **C**, which showed solvent dependent ylide‐phosphine equilibria in solution and the preference of the ylidic form in the solid state based on IR spectroscopic data.^[3]^ The first crystallographic proof of a stable P−H ylide was reported by Niecke in 2002 with P,N‐macrocycle **D**, in which the ylide was further stabilized by an intramolecular hydrogen bond.^[4]^ Few further examples of PH ylides have been reported and their chemistry remains virtually unexplored.^[5]^

Based on our efforts to synthesize electron‐rich phosphines[^6^, ^7^] we became interested in the generation of stable PH ylides. We hypothesized that these ylides should be ideal starting materials to α‐carbanionic phosphines with exclusive P‐centered reactivity. In general, phosphinomethanides have been known for many years and their s‐block metal complexes studied in detail with important contributions being made for example by Karsch, Müller and Izod.[^8^, ^9^] Usually, phosphinomethanides with an non‐stabilized carbanionic center react as carbon nucleophiles to introduce α‐phosphinomethylene moieties.^[10]^ However, with an appropriate molecular design the reactivity can be shifted to the phosphorus center rendering the carbanion as a “simple” electron‐donating group. Such observations have been made by Karsch using silyl‐substituted phosphinomethanides, which still however showed partly *C*‐centered reactivity and *P*‐to‐*C* equilibrium processes.^[11]^ However, competing C‐centered reactivities may lead to undesired side‐reactions when applying these phosphines in transition metal complexes and catalysts. Since PH ylides already undergo protonation at the phosphorus center, the carbanion reactivity in these systems should be sufficiently suppressed. This should allow for exclusive phosphine reactivity and the switching of a phosphine from an electron‐poor (protonated state) to an electron‐rich donor.[^12^, ^13^] We envisioned that due to the excellent anion‐stabilizing ability of sulfonyl groups, bis(sulfonyl)methyl substituents should be ideal for the isolation of stable PH ylides **E** and the carbanionic phosphines with exclusive phosphorus‐centered reactivity.

## Results and Discussion

### Synthesis and Characterization of the PH Ylides

**Scheme 1:**
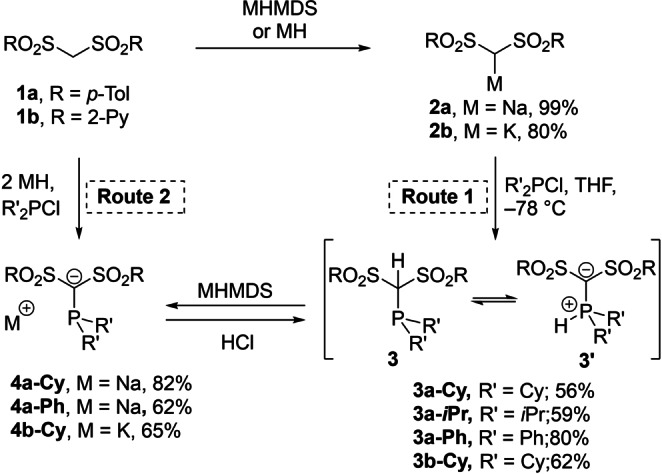
Preparation of α‐carbanionic phosphines. Route 1 for **1 a** and R=Cy, iPr. Route 2 was used for **1 b** and **1 a** with Ph_2_PCl.

To test this hypothesis, we addressed the synthesis of a series of bis‐tosyl functionalized phosphines **3 a‐R** with different substituents at the phosphorus center (Cy, Ph, *i*Pr). For comparison, the pyridyl‐substituted analogues **3 b‐R** were targeted. Both bis(sulfonyl)methane precursors **1 a** and **1 b** were prepared in two steps from the corresponding thiols via coupling with diiodomethane and subsequent oxidation (see the Supporting Information). For the synthesis of phosphines **3‐R**, two different routes were found to be applicable, depending on the basicity of the bis(sulfonyl)methanides **2 a** and **2 b** and the donor strength of the introduced phosphine moiety (Scheme 1): Route 1 proceeds via the corresponding methanides **2 a** and **2 b** and their reaction with the respective chlorophosphines. Since the one‐pot synthesis was unselective, methanide **2 a** was first isolated as a colorless, crystalline solid^[14]^ in quantitative yields and afterwards converted to **3‐R** by treatment with Cy_2_PCl or *i*Pr_2_PCl. In Route 2, the anionic phosphines **4‐R** are directly synthesized by treatment of **1 a** or **1 b** with 2 equiv of base and one equiv. chlorophosphine. Subsequent protonation with hydrochloric acid gives the neutral phosphines **3‐R**. This route was used for the pyridyl‐substituted phosphine **3 b‐Cy** and the diphenylphosphine **3 a‐Ph**, whereas the alkylphosphines **3 a‐Cy** and **3 a‐*i*Pr** were prepared via Route 1. Both pathways delivered the phosphines as colorless solids in 56–80 % yield. Higher yields were prevented by competing re‐protonation of the bis(sulfonyl)methanides and reformation of **1 a** and **1 b** as difficult‐to‐separate byproducts.

The NMR spectra of the phosphines **3‐R** showed remarkable differences and suggested the formation of different tautomeric forms in solution depending on the sulfonyl and R′ group. For example, a solution of **3 a‐Cy** in THF showed two signals at 17.4 and 19.8 ppm in the ^31^P{^1^H} NMR spectrum corresponding to the ylide form **3′** and phosphine **3** in an approx. 4 : 1 ratio. Likewise, the ^1^H NMR spectrum exhibited two sets of signals with the signal of the proton at the SCS linkage appearing as a doublet at 4.89 ppm (^2^ *J*_HP_=1.43 Hz). In contrast, the ylide tautomer could be identified by the characteristic *P*H signal which appears as a doublet of triplets at 6.02 ppm (^1^ *J*_PH_=447.8 Hz; ^3^ *J*_HH_=7.45 Hz). The large ^1^ *J*_PH_ coupling constant compares well with Niecke's PH‐ylide macrocycle (457 Hz).^[4]^ ^31^P decoupling experiments led to a simple triplet, thus confirming the presence of a phosphorus‐bound hydrogen atom. While **3 a‐*i*Pr**, like **3 a‐Cy**, forms an equilibrium between **3‐R** and **3′‐R** with preference of the PH‐ylide in THF solution, **3 a‐Ph** with a less electron‐rich phosphorus center only exists in the phosphine form. For the 2‐pyridyl‐substituted compound **3 b‐Cy** also no equilibrium was observed, but exclusive formation of the PH ylide. **3 b‐Cy** thus represents the first example of an acyclic phosphine which exclusively exists in its PH ylide form.^[15]^ The higher stability of the ylide form for the pyridyl compound compared to its tolyl analogue can be explained by the stronger ability of the pyridyl group to stabilize a negative charge (see chapter 4.2 in the Supporting Information).

**Figure 2:**
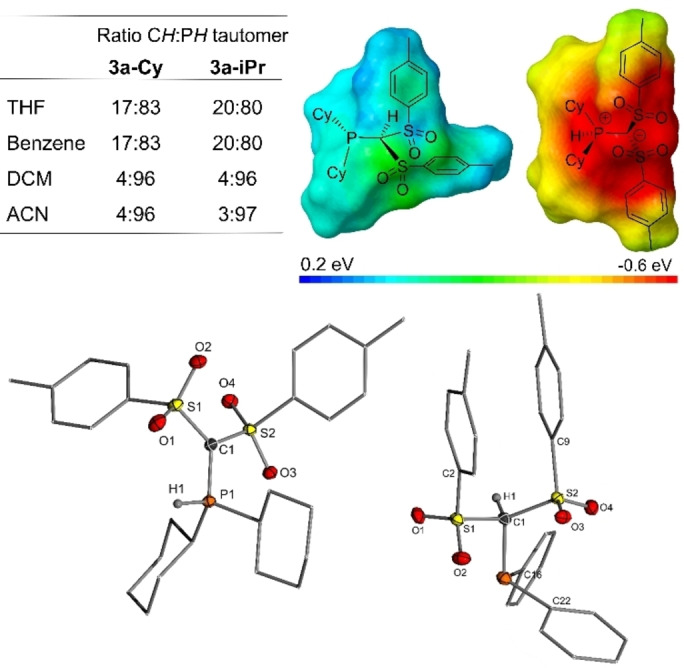
Top: Ratio of CH:PH tautomer in different solvents and electrostatic potential mapped on the Connolly surface (ball size: 2.3 Å) of **3 a‐Cy** (left) and **3 a′‐Cy** (right). Bottom: Molecular structures of **3 a‐Cy** and **3 a‐Ph**. Thermal ellipsoids at the 50 % level, hydrogen atoms are omitted for clarity except for H1. Important bond lengths and angles are given in Table 1.

The NMR spectra of **3 a‐Cy** and **3 a‐*i*Pr** showed different ratios between **3‐R** and **3′‐R** depending on the solvent. To further examine this solvent dependency of the equilibrium, a defined amount of phosphine was dissolved in a specific solvent and the resulting ratio determined by ^31^P{^1^H} NMR spectroscopy (Figure 2). Overall, the ylidic form **3′‐R** seems to be preferred in polar solvents. For example, 17 % of the phosphine form of **3 a‐Cy** is observed in benzene, whereas in acetonitrile (ACN) almost exclusively the PH ylide is formed. Overall, the solvent dependency is only minor^[3]^ and does not perfectly correlate with the polarity of the solvents as judged by their dielectric constants. Therefore, we performed density functional theory (DFT) calculations at the PW6B95‐D3/def2tzvp level to obtain further insights into the structures and energy differences of the tautomers. Overall, the calculations confirmed the preference of the ylide over the phosphine form. The sole exception is the diphenylphosphine system **3 a‐Ph**, for which the phosphine form was favored by Δ*H*=4 kJ mol^−1^ (see Supporting Information for details). Accordingly, the calculated proton affinities (PAs) for the protonation of **4 a‐Cy** and **4 b‐Cy** at the phosphorus atom are larger (ΔPA=10 and 17 kJ mol^−1^, respectively) than those for the protonation of the carbon center (all values are given in Table S47). The electrostatic potential at the surfaces (Connolly surface) of the phosphines and ylides were found to be rather homogeneous, with the positive charges being efficiently shielded in the inner sphere of the molecules. This—together with the energetic differences—agrees well with the minor solvent effects on the equilibrium between CH and PH tautomer.

The tautomers preferred in solution were also found to be the preferred isomers in the solid state (Figure 2). Thus, the phosphine form was obtained for **3 a‐Ph** and the PH ylide form for **3 a‐Cy**, **3 a‐*i*Pr** and **3 b‐Cy**, which represent the first structurally characterized examples of neutral acyclic PH ylides, according to the CCDC database. The structure parameters of the PH ylides (Table 1) differ significantly from those of the phosphines. Due to the additional electrostatic attraction in the ylides, the P1−C1 bonds of approx. 1.735 Å are considerably shorter than the P1−C1 bond in **3 a‐Ph** (1.901(2) Å) and other common phosphines.^[15]^ The same holds true for the C1−S distances. Moreover, the geometry at the central carbon center changes from a tetrahedral geometry in **3 a‐Ph** to a planar environment in the ylides. Notably, the conformation of the structures of the two tautomers **3‐R** and **3′‐R** showed distinct differences. Whereas the phosphine form of **3 a‐Ph** features a compact structure with coplanar tolyl groups (π‐stacking), these groups occupy anti‐parallel positions in the ylidic forms of **3 a′‐Cy** and **3 a′‐iPr**. This arrangement is preferred to stabilize the carbanionic charge into the anti‐bonding σ*(S−C_Tol/Py_) orbital.

**Table 1:** Selected bond lengths [Å] and angles [°] for the phosphines and PH ylides **3‐R** and **3′‐R** and the anionic phosphines **4‐R**.

	P−C [Å]	S−C1 [Å]^[a]^	S1‐C1‐S2 [°]	Σα(C1) [°]
**3 b′‐Cy**	1.741(2)	1.701(6)	119.6(1)	359.9(5)
**3 a′‐Cy**	1.733(1)	1.708(4)	120.6(8)	359.92(7)
**3 a′‐*i*Pr**	1.741(3)	1.719(3)	118.7(2)	358.4(7)
**3 a‐Ph**	1.901(2)	1.820(5)	116.0(1)	–
**4 a‐Cy**	1.793(4)	1.700(3)	118.9(3)	359.7(3)
**4 a‐Ph**	1.791(2)	1.712(5)	118.5(9)	359.7(5)

[a] Average values are given.

### Synthesis and Properties of α‐Carbanionic Phosphines

**Figure 3:**
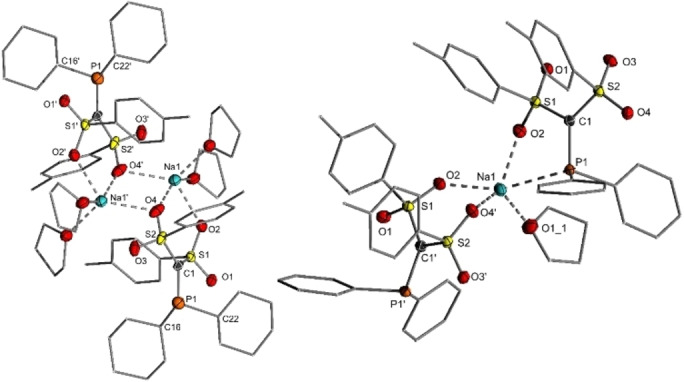
Molecular structures of **4 a‐Cy** and **4 a‐Ph** in the solid state (thermal ellipsoids at the 50 % level, hydrogen atoms omitted for clarity). Important bond lengths and angles are given in Table 1.

With the PH ylides in hand, we attempted the isolation of the corresponding phosphinomethanides **4‐R**. To our delight, the anions can easily be generated by deprotonation with NaHMDS or—as mentioned above—directly from **1**, thus delivering the anionic phosphines **4 a‐Cy**, **4 a‐Ph** and **4 b‐Cy** as colorless solids in moderate to good yields between 60 and 80 % (Scheme 1). The carbanionic phosphines are characterized by a high‐field shift in the ^31^P NMR spectra, e.g. from approx. 18 ppm in **3 a‐Cy** to −0.27 ppm in **4 a‐Cy**. Single crystals could be obtained for the two tosyl‐substituted compounds (Figure 3). **4 a‐Cy** crystallizes as a dimer in which the sodium atoms are coordinated by five oxygen atoms from the sulfonyl groups and coordinating THF molecules. In contrast, **4 a‐Ph** forms a coordination polymer, in which sodium is likewise coordinated by THF and the sulfonyl moieties of two molecules of **4 a‐Ph** as well as by one phosphine group. The P−C1 bond length in both structures amounts to approx. 1.80 Å and is thus significantly elongated relative to the one in the PH ylide **3 a‐PCy** (1.733(1) Å; Table 1) but shortened compared to the phosphine structure of **3 a‐Ph** (1.901(2) Å). This can be explained by the repulsion of the lone pair at the neighboring carbon and phosphorus atom and the change in hybridization from sp^3^ to sp^2^ for the PPh_2_ system. Most notably, both anions don't show any contact between C1 and the sodium ions, thus confirming the efficient stabilization of the carbanion. In contrast, P1 in **4 a‐Ph** binds to the metal center, which thus represents a rare example of a sodium monophosphine complex.^[16]^

The impact of introducing a negative charge in the α‐position to the phosphorus atom on the phosphine nucleophilicity was first studied by computational methods.^[17]^ Interestingly, natural bond orbital (NBO) analyses^[18]^ on the phosphinomethanides **4‐R** in comparison to phosphines **3‐R** showed no increase of negative charge at the phosphorus center. However, the Tolman electronic parameter (TEP)^[19]^ calculated from the LNi(CO)_3_ complexes [TEP_calc_] showed a significant increase of donor strength e.g. by 18 cm^−1^ for **4 a‐Cy**. This increase is also confirmed by the experimental values obtained from the correlation of the ^1^ *J*_PSe_ coupling constants with the TEP values [TEP_exp_].^[20]^ Here, a more pronounced change in the TEP value by 22 and 25 cm^−1^, respectively, was observed for **3 b** and **3 a**.^[21]^ This increase in the TEP value represents a strong change in donor strength of the phosphine by deprotonation. Similar changes have been reported for other tertiary phosphine system by other external stimuli (pH, redox, photo),[^12^, ^13^] and a somewhat strong change for secondary phosphine oxides.^[22]^ Overall, the deprotonation allows the switch of an electron‐poor phosphine to a highly electron‐rich donor. With a TEP of 2045 cm^−1^, the anionic tolyl‐substituted phosphine **4 a‐Cy** revealed to be the most electron‐donating species. This value is significantly lower than those of common neutral (e.g. TEP(P*t*Bu_3_): 2056.1 cm^−1^)^[23]^ and comparable to highly electron‐rich phosphines[^24^, ^25^] such as our ylide‐substituted phosphines.^[6]^ It is noteworthy that the stronger nucleophilicity of the α‐carbanionic phosphines compared to their protonated congeners is also reflected in the increased proton affinities (PAs). In general, carbanion‐functionalization results in an increase of the PAs of the phosphines **3‐R** by 26–33 kcal mol^−1^ compared to **4‐R**. These PAs are always higher than those for the protonation at the carbon atom (except for the diphenylphosphine systems).

**Figure 4:**
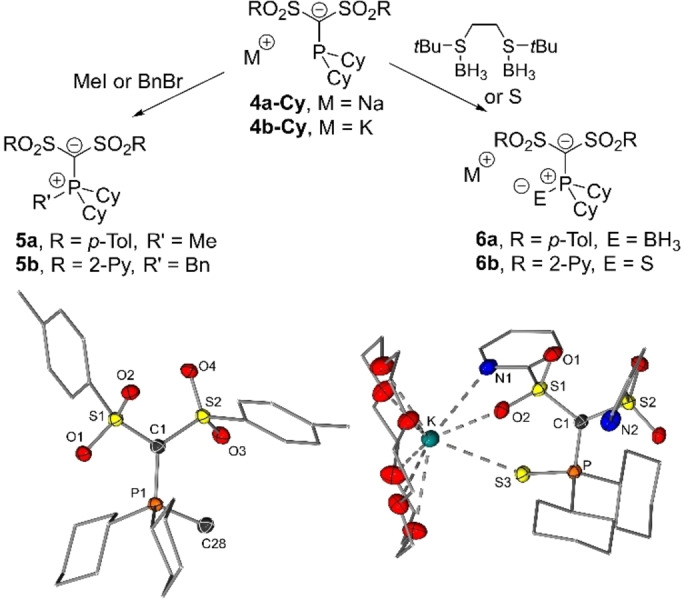
Reactivity studies of the carbanionic phosphines and molecular structures of **5 a** and **6 b**.

The calculated TEP values and PAs suggest a high donor strength and nucleophilicity of the phosphinomethanides **4** at the phosphorus atom. To examine whether this leads to an exclusive phosphorus‐based reactivity, the anions were treated with different electrophiles. At first, special focus was set on small electrophiles to minimize competing steric effects. As electrophiles simple alkyl halides (methyl iodide and benzyl bromide) as well as BH_3_ as its 1,2‐bis(*tert‐*butylthio)ethane complex and elemental sulfur were employed (Figure 4). Fortunately, only a single product was formed in all reactions with the electrophile attacking at the phosphorus center, thus giving way to compounds **5** and **6**. All compounds could be unambiguously characterized and were isolated in high yields between 75 and 99 %. In none of the cases, reaction at the carbon atom was observed, despite the high negative charge located at the carbanionc center (Table 2). It is also noteworthy that the ylide precursors **3′‐R**—in accordance with the weak donor strength of the phosphines—don't or only unselectively react with the same electrophiles. This demonstrates the potential of the stabilized α‐carbanionic phosphines to serve as switchable phosphines depending on the protonation state and the propensity of carbanions to serve not only as reactive sites but also as electron‐donating groups.

**Table 2:** Comparison of the calculated natural charges at phosphorus and carbon and TEP values obtained by correlation of the TEP with the ^1^ *J*_PSe_ coupling constants [TEP_exp_] and from the calculated CO stretching frequencies of the LNi(CO)_3_ complexes [TEP_calc_] as well as the proton affinities for protonation at the phosphorus atom in the anionic phosphines **4** and neutral phosphines **3**. The values for all compounds are given in the Supporting Information. Calculations were performed on the PW6B95‐D3/def2tzvp level of theory. Values are given for the most stable conformers as obtained with the program package CREST.^[17]^

	**4 a‐Cy**	**3 a′‐Cy**	**3 a‐Cy**	**4 a‐Ph**	**3 a‐Ph**	**4 b‐Cy**	**3 b‐Cy**	**3 b′‐Cy**
q(P)	0.866	1.413	0.856	0.896	0.872	0.878	0.856	1.424
q(C)	−1.310	−1.346	−1.062	−1.309	−1.041	−1.294	−1.093	−1.341
TEP_exp_ [cm^−1^]	2045	–	2070	–	–	2046	2068	–
TEP_calc_ [cm^−1^]	2046	–	2064	2049	2074	2048	2065	–
PA (@P) [kcal mol^−1^]	−294.5	–	−267.1	−289.1	−261.2	−296.8	−263.8	–

### Catalytic Applications

**Figure 5:**
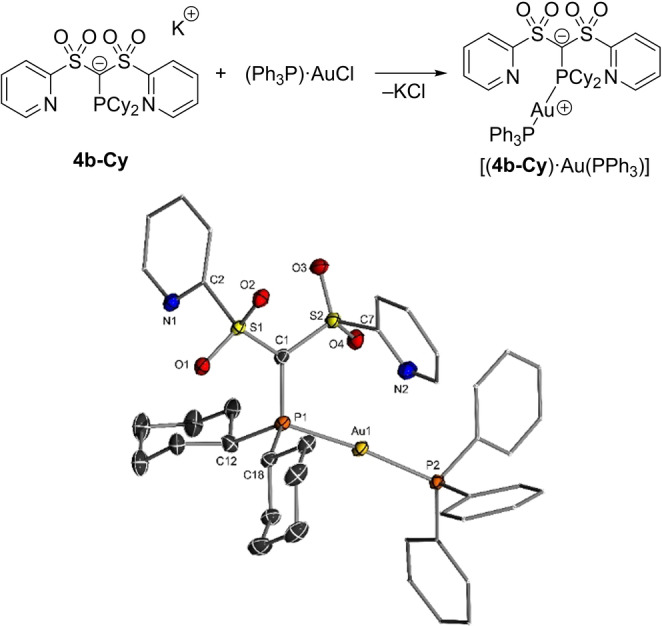
Synthesis of the zwitterionic gold complex [(**4 b‐Cy**)⋅Au(PPh_3_)] (top) and its molecular structure (bottom). Selected bond lengths [Å] and angles [°]: S1−C1 1.711(2), C1−S2 1.713(2), P1−C1 1.775(2), Au1−P2 2.2943(6), Au1−P1 2.3217(6); P2‐Au1‐P1 172.03(2), S1‐C1‐S2 116.51(14), S1‐C1‐P1 122.85(13), S2‐C1‐P1 119.55(13).

Motivated by the exclusive P‐centered reactivity of the α‐carbanionic phosphines, we next evaluated their propensity to function as ligands in metal catalysts. Since cationic gold complexes are often used in catalysis for the activation of alkenes or alkynes, we attempted the synthesis of zwitterionic gold complexes.^[26]^ First experiments focused on the pyridyl system **4 b‐Cy** due to the possible stabilization of the metal center through additional *N*‐coordination. Unfortunately, initial attempts by reacting (THT)AuCl (THT=tetrahydrothiophene) with **4 b‐Cy** were unsuccessful. The reaction repeatedly led to the formation of two products, presumably due to either the replacement of the chloride or THT by the phosphine ligand. However, treatment of **4 b‐Cy** with [(Ph_3_P)AuCl] selectively delivered a single new complex characterized by two doublets in the ^31^P{^1^H} NMR spectrum at δ_P_=42.5 and 50.4 ppm with a large coupling constant of ^2^ *J*_PP_=294.4 Hz. This complex could be identified as the desired zwitterionic complex [(**4 b‐Cy**)⋅Au(PPh_3_)] and structurally characterized by XRD analysis. The molecular structure (Figure 5) of the complex features an almost linear P‐Au‐P unit, with the P1−Au distance (2.3217(6) Å) to the anionic phosphine being slightly longer than the P2−Au bond (2.2943(6) Å) to the PPh_3_ ligand.

The isolation of [(**4 b‐Cy**)⋅Au(PPh_3_)] clearly confirmed the propensity of the carbanionic phosphines to stabilize zwitterionic complexes. To proof the utility of these complexes in catalysis, [(**4 b‐Cy**)⋅Au(PPh_3_)] as well as the complexes in situ formed from [(THT)AuCl] and **4 b‐Cy** were employed in the hydroamination of phenyl acetylene with aniline as test reaction at 50 °C. In general, LAuCl complexes with electron‐rich phosphines or carbenes have been used for this reaction,^[27]^ but require the activation by an additional halide abstraction reaction (e.g. AgBF_4_ or NaBAr^F^) to access the catalytically active cationic gold species.^[28]^ Due to the zwitterionic nature of our complexes and the already present cationic metal center, we expected that no additives should be necessary for generation of the active species. Indeed, whereas the PPh_3_ complex showed no activity—presumably due to the too strong coordination of both phosphines and the blocking of the gold center—the complex generated from [(THT)AuCl] and **4 b‐Cy** revealed to be active. Full conversion was observed with only 0.5 mol % after 24 h (Entry 3, Table 3). Due to the basic reaction medium we envisioned that also an in situ activation of the AuCl complex of the neutral phosphines might be possible through HCl elimination. Indeed, employment of [(**3 b‐Cy**)AuCl)] as catalyst gave 89 % conversion after 24 h (Entry 5). Again, no activation by a halide abstraction reagent was required. This contrasts the reactivity of “simple” neutral phosphines. Here, no conversion was observed with [PPh_3_⋅AuCl], [(THT)AuCl] (Entry 1) or AuCl complexes with more sophisticated ligands such as our ylide‐substituted phosphines (YPhos),^[29]^ when no halide‐abstraction reagent was added. These results clearly demonstrate the utility of our carbanionic phosphines for the formation of zwitterionic catalysts. It must be noted that the catalysts are active in protic media (aniline), confirming the stability of the carbanionic center toward protonation or decomposition under these conditions.

**Table 3:** Gold‐catalysed hydroamination of phenylacetylene with aniline with different gold complexes.^[a]^

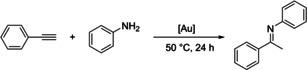			
Entry	Catalyst	Mol %	Yield [%]
1	PPh_3_AuCl or (THT)AuCl	0.5	n.o.
2	[(**4 b‐Cy**)⋅Au(PPh_3_)]	0.5	n.o
**3**	**4 b‐Cy**+(THT)AuCl	0.5	99
4	**4 b‐Cy**+(THT)AuCl	0.1	75
5	[(**3 b‐Cy**)AuCl)]	0.5	89
6	[(**3 b‐Cy**)AuCl)]	0.1	66
7	PPh_3_AuCl+NaBAr^F^	0.1	39

[a] Conditions: 50 °C, 24 h, neat, phenylacetylene (1 equiv), aniline (1.1 equiv). Yields were calculated by NMR spectroscopy. NaBAr^F^=sodium tetrakis‐[3,5‐bis‐(trifluoromethyl)phenyl]‐borate.

## Conclusion

In conclusion, we reported on the synthesis of di(sulfonyl)‐substituted organophosphines which readily form stable PH ylides also in solution. Depending on the substitution pattern an equilibrium between the PH ylide and the corresponding phosphine form was observed. In the case of the pyridyl‐substituted compound the first acyclic alkylphosphine that exclusively exists as its PH ylide could be isolated and structurally authenticated. The phosphines readily gave way to the corresponding phosphinomethanides **4‐R** which exhibited exclusive phosphorus‐centered reactivity when treated with different electrophiles. DFT calculations confirmed the remarkable increase in donor strength and basicity imparted by the carbanionic electron‐donating substituent. This special donor strength makes these α‐carbanionic phosphines to attractive pH‐switchable ligands to access zwitterionic complexes with unique ligand properties. The utility of the ligands were showcased by the formation of zwitterionic complexes of the type [(**4‐Cy**)⋅AuL] with a cationic gold center. These complexes were found to be active catalysts for the hydroamination of phenylacetylene without requiring an additional activation by a halide‐abstraction reagent. Further applications of the carbanionic phosphines in transition metal catalysis are currently being investigated.

## Experimental Section

Experimental details, procedures and compound characterizations, NMR spectra and crystallographic data as well as computational details including cartesian coordinates are provided in the Supporting Information.

## Conflict of interest

The authors declare no conflict of interest.

1

## Supporting information

As a service to our authors and readers, this journal provides supporting information supplied by the authors. Such materials are peer reviewed and may be re‐organized for online delivery, but are not copy‐edited or typeset. Technical support issues arising from supporting information (other than missing files) should be addressed to the authors.

Supporting Information

Supporting Information

Supporting Information

## Data Availability

The data that support the findings of this study are available in the Supporting Information of this article.

## References

[1a] B. F. Yates , W. J. Bouma , L. Radom , J. Am. Chem. Soc. 1984, 106, 5805–5808;

[1b] H. Keck , W. Kuchen , P. Tommes , J. K. Terlow , T. Wong , Angew. Chem. Int. Ed. Engl. 1992, 31, 86;

[2a] J. Chatt , B. T. Heaton , J. Chem. Soc. A 1968, 2745–2757;

[2b] A. Christiansen , C. Li , M. Garland , D. Selent , R. Ludwig , A. Spannenberg , W. Baumann , R. Franke , A. Börner , Eur. J. Org. Chem. 2010, 2733–2741;

[2c] B. Hoge , S. Neufeind , S. Hettel , W. Wiebe , C. Thoesen , J. Organomet. Chem. 2005, 690, 2382–2387;

[2d] B. Hoge , P. Garcia , H. Willner , H. Oberhammer , Chem. Eur. J. 2006, 12, 3567–3574. 16491490 10.1002/chem.200501066

[3] O. I. Kolodiazhnyi , Tetrahedron Lett. 1980, 21, 2269–2272.

[4] S. Ekici , D. Gudat , M. Nieger , L. Nyulaszi , E. Niecke , Angew. Chem. Int. Ed. 2002, 41, 3367–3371; 10.1002/1521-3773(20020916)41:18<3367::AID-ANIE3367>3.0.CO;2-7 12298034

[5a] S. Ito , H. Miyake , M. Yoshifuji , T. Höltzl , T. Veszprémi , Chem. Eur. J. 2005, 11, 59605965; 10.1002/chem.200500374 16052657

[5b] Y. Ueta , K. Mikami , S. Ito , Inorg. Chem. 2015, 54, 8778–8785; 26313533 10.1021/acs.inorgchem.5b01399

[5c] S. Yogendra , F. Hennersdorf , A. Bauzá , A. Frontera , R. Fischer , J. J. Weigand , Dalton Trans. 2017, 46, 15503–15511; 29090714 10.1039/c7dt03271d

[5d] S. Ito , J. Miura , N. Morita , M. Yoshifuji , A. J. Arduengo III , Inorg. Chem. 2009, 48, 8063–8065. 19637863 10.1021/ic901072z

[6a] A. Sarbajna , V. S. V. S. N. Swamy , V. H. Gessner , Chem. Sci. 2021, 12, 2016–2024; 10.1039/d0sc03278f PMC8179322 34163963

[6b] X.-Q. Hu , D. Lichte , I. Rodstein , P. Weber , A.-K. Seitz , T. Scherpf , V. H. Gessner , L. J. Gooßen , Org. Lett. 2019, 21, 7558; 31469570 10.1021/acs.orglett.9b02830

[6c] P. Weber , T. Scherpf , I. Rodstein , D. Lichte , L. T. Scharf , L. J. Gooßen , V. H. Gessner , Angew. Chem. Int. Ed. 2019, 58, 3203–3207; 10.1002/anie.201810696 30451339

[6d] T. Scherpf , H. Steinert , A. Großjohann , K. Dilchert , J. Tappen , R. Rodstein , V. H. Gessner , Angew. Chem. Int. Ed. 2020, 59, 20596–20603; 10.1002/anie.202008866 PMC7692947 32725943

[6e] T. Scherpf , C. Schwarz , L. T. Scharf , J.-A. Zur , A. Helbig , V. H. Gessner , Angew. Chem. Int. Ed. 2018, 57, 12859–12864; 10.1002/anie.201805372 PMC6174943 29862622

[7] For a recent review on super basic phosphines, see: R. F. Weitkamp , B. Neumann , H.-G. Stammler , B. Hoge , Chem. Eur. J. 2021, 27, 10807–10825. 34032319 10.1002/chem.202101065 PMC8362139

[8] For reviews, see:

[8a] K. Izod , Adv. Inorg. Chem. 2000, 50, 33;

[8b] K. Izod , Coord. Chem. Rev. 2002, 227, 153–173.

[9a] H. H. Karsch , G. Müller , Chem. Commun. 1984, 569;

[9b] V. Knapp , G. Müller , Angew. Chem. Int. Ed. 2001, 40, 183–186; 10.1002/1521-3773(20010105)40:1<183::AID-ANIE183>3.0.CO;2-8 29711961

[9c] W. Clegg , K. Izod , P. O'Shaughness , Organometallics 1999, 18, 2939–2940;

[9d] H. H. Karsch , G. Grauvogl , P. Mikulcik , P. Bissinger , G. Mueller , J. Organomet. Chem. 1994, 465, 65–71;

[9e] K. Izod , P. O'Shaughness , W. Clegg , S. T. Liddle , Organometallics 2001, 20, 648–653.

[10] For examples:

[10a] H. Broda , J. Krahmer , F. Tuczek , Eur. J. Inorg. Chem. 2014, 3564–3571;

[10b] H. H. Karsch , E. Witt , J. Organomet. Chem. 1997, 529, 151–169;

[10c] M. Weger , R. K. Grötsch , M. G. Knaus , M. M. Giuman , D. C. Mayer , P. J. Altmann , E. Mossou , B. Dittrich , A. Pöthig , B. Rieger , Angew. Chem. Int. Ed. 2019, 58, 9797–9801; 10.1002/anie.201902833 31046187

[10d] S. R. Klei , T. D. Tilley , R. G. Bergman , Organometallics 2002, 21, 4905–4911;

[10e] T. A. Mobley , R. G. Bergman , J. Am. Chem. Soc. 1998, 120, 3253–3254;

[10f] A. Prades , Fernández , S. D. Pike , M. C. Willis , A. S. Weller , Angew. Chem. Int. Ed. 2015, 54, 8520–8524; 10.1002/anie.201503208 PMC4531818 26069052

[10g] P. Holtkamp , F. Friedrich , W. Stratmann , A. Mix , B. Neumann , H.-G. Stammler , N. W. Mitzel , Angew. Chem. Int. Ed. 2019, 58, 5114–5118; 10.1002/anie.201901037 30758907

[11a] H. H. Karsch , Russ. Chem. Bull. 1993, 42, 1937–1955;

[11b] H. H. Karsch , R. Richter , A. Schier , Z. Naturforsch. 1993, 48b, 1533–1543;

[11c] H. H. Karsch , R. Richter , B. Deubelly , A. Schier , M. Paul , M. Heckel , K. Angermeier , W. Hiller , Z. Naturforsch. 1994, 49b, 1798–1808;

[11d] H. H. Karsch , G. Grauvogel , M. Kawecki , P. Bissinger , Organometallics 1993, 12, 2757–2766.

[12] For pH-switchable phosphines, see:

[12a] P. Mehlmann , F. Dielmann , Chem. Eur. J. 2019, 25, 2352–2357; 30506604 10.1002/chem.201805540

[12b] B. S. Birenheide , F. Krämer , L. Bayer , P. Mehlmann , F. Dielmann , F. Breher , Chem. Eur. J. 2021, 27, 15067–15074. 10.1002/chem.202101969 PMC8596786 34459528

[13] For other switchable phosphines and carbenes, see:

[13a] A. Feyrer , M. K. Armbruster , K. Fink , F. Breher , Chem. Eur. J. 2017, 23, 7402–7408; 28422329 10.1002/chem.201700868

[13b] A. T. Biju , K. Hirano , R. Frohlich , F. Glorius , Chem. Asian J. 2009, 4, 1786–1789; 19790207 10.1002/asia.200900410

[13c] L. Benhamou , V. Cesar , H. Gornitzka , N. Lugan , G. Lavigne , Chem. Commun. 2009, 4720–4722; 10.1039/b907908d 19641821

[13d] B. M. Neilson , V. M. Lynch , C. W. Bielawski , Angew. Chem. Int. Ed. 2011, 50, 10322–10326; 10.1002/anie.201105032 21915980

[13e] S. Vanicek , M. Podewitz , J. Stubbe , D. Schulze , H. Kopacka , K. Wurst , T. Meller , P. Lippmann , S. Haslinger , H. Schottenberger , K. R. Liedl , I. Ott , B. Sarkar , B. Bildstein , Chem. Eur. J. 2018, 24, 3742. 29214677 10.1002/chem.201705051 PMC6100101

[14] Deposition Numbers 2154851 (for **1b**), 2154852 (for **2b**), 2154853 (for **1a**), 2154854 (for **2a**), 2154855 (for **3a-Cy**), 2154856 (for **3a-** * **i** * **Pr**), 2154857 (for **3a-Ph**), 2154858 (for **3b-Cy**), 2154859 (for **4a-Cy**), 2154860 (for **4a-Ph**), 2154861 (for **5a**), 2154862 (for **5b**), 2154863 (for **6a**), 2154864 (for **6b**), 2169766 (for [(**3b-Cy**)AuCl]), and 2169767 (for [(**4b-Cy**)Au(PPh_3_)]) contains the supplementary crystallographic data for this paper. These data are provided free of charge by the joint Cambridge Crystallographic Data Centre and Fachinformationszentrum Karlsruhe Access Structures service.

[15] For a related carbodiphosphorane, see: M. Soleilhavoup , A. Baceiredo , G. Bertrand , Angew. Chem. Int. Ed. Engl. 1993, 32, 1167–1169;

[16a] C. Wills , K. Izod , J. Young , W. Clegg , R. W. Harrington , Dalton Trans. 2009, 6159–6165; 20449112 10.1039/b903119g

[16b] M. N. S. Hill , K. Izod , P. Shaughnessy , W. Clegg , Organometallics 2000, 19, 4531–4535;

[16c] M. A. Stevens , F. H. Hashim , E. S. H. Gwee , E. I. Izgorodina , R. E. Mulvey , V. L. Blair , Chem. Eur. J. 2018, 24, 15669–15677. 30101451 10.1002/chem.201803477

[17] For each compound the conformational space was analyzed with the program package CREST by Grimme. TEP values and charges are based on the most favored conformer. P. Pracht , F. Bohle , S. Grimme , Phys. Chem. Chem. Phys. 2020, 22, 7169. 32073075 10.1039/c9cp06869d

[18] E. D. Glendening , C. R. Landis , F. Weinhold , J. Comput. Chem. 2019, 40, 2234–2241. 31172571 10.1002/jcc.25873

[19] C. A. Tolman , J. Am. Chem. Soc. 1970, 92, 2953–2956.

[20] Unfortunately, attempts to obtain reliable values via IR spectroscopic measurements on L⋅Ni(CO)_3_, L⋅Rh(acac)(CO) or L⋅Ir(CO)Cl failed, either due to the displacement of an additional anionic X ligand (due to the anionic nature of our phosphines) or due to displacement of presumably a second CO ligand.

[21] It must be noted that TEP values calculated from the minimum of the electrostatic potential ( C. H. Suresh , N. Koga , Inorg. Chem. 2002, 41, 1573–1578.) at the phosphorus center suggest an even more dramatic increase in donor strength by deprotonation, e.g. by 45.2 cm^−1^ in case of **4 a-Cy** (see the Supporting Information, Chapter S2). Due to the anionic nature of **4** the measurement of TEP values, which are usually obtained for L-type ligands, may have a large uncertainty.11896726

[22] D. Martin , D. Moraleda , T. Achard , L. Giordano , G. Buono , Chem. Eur. J. 2011, 17, 12729–12740. 21956620 10.1002/chem.201101663

[23] C. A. Tolman , Chem. Rev. 1977, 77, 313–348.

[24a] M. A. Wünsche , P. Mehlmann , T. Witteler , F. Buß , P. Rathmann , F. Dielmann , Angew. Chem. Int. Ed. 2015, 54, 11857–11860; 10.1002/anie.201504993 26265298

[24b] F. Buß , P. Mehlmann , C. Mück-Lichtenfeld , K. Bergander , F. Dielmann , J. Am. Chem. Soc. 2016, 138, 1840–1843; 26824487 10.1021/jacs.5b13116

[24c] F. Buß , C. Mück-Lichtenfeld , P. Mehlmann , F. Dielmann , Angew. Chem. Int. Ed. 2018, 57, 4951–4955; 10.1002/anie.201713206 29437280

[25] S. Ullrich , B. Kovačević , X. Xie , J. Sundermeyer , Angew. Chem. Int. Ed. 2019, 58, 10335–10339; 10.1002/anie.201903342 31037821

[26] For a recent review on zwitterionic catalysts, see: R. Puerta-Oteo , A. I. Ojeda-Amador , M. V. Jiménez , J. J. Pérez-Torrente , Dalton Trans. 2022, 51, 817–830. 34904607 10.1039/d1dt03746c

[27] For examples, see:

[27a] D. Malhotra , M. S. Mashuta , G. B. Hammond , B. Xu , Angew. Chem. Int. Ed. 2014, 53, 4456–4459; 10.1002/anie.201310239 24652771

[27b] Y. Wang , Z. Wang , L. Yuxue , G. Wu , Z. Cao , L. A. Zhang , Nat. Commun. 2014, 5, 3470; 24704803 10.1038/ncomms4470 PMC4119785

[27c] S. Yazdani , G. P. Junor , J. L. Peltier , M. Gembicky , R. Jazzar , D. B. Grotjahn , G. Bertrand , ACS Catal. 2020, 10, 5190–5201;

[27d] W. Wang , G. B. Hammond , B. Xu , J. Am. Chem. Soc. 2012, 134, 5697–5705; 22376128 10.1021/ja3011397

[27e] Y. Tang , I. Benaissa , M. Huynh , L. Vendier , N. Lugan , S. Bastin , P. Belmont , V. César , V. Michelet , Angew. Chem. Int. Ed. 2019, 58, 7977–7981; 10.1002/anie.201901090 30957361

[28a] J. Schießl , J. Schulmeister , A. Doppiu , E. Wörner , M. Rudolph , R. Karch , A. S. K. Hashmi , Adv. Synth. Catal. 2018, 360, 2493;

[28b] A. Homs , C. Obradors , D. Leboeuf , A. M. Echavarren , Adv. Synth. Catal. 2014, 356, 221. 26190958 10.1002/adsc.201300704 PMC4498468

[29a] J. Handelmann , C. Naga Babu , H. Steinert , C. Schwarz , T. Scherpf , A. Kroll , V. H. Gessner , Chem. Sci. 2021, 12, 4329–4337; 34168748 10.1039/d1sc00105a PMC8179644

[29b] C. Schwarz , J. Handelmann , D. M. Baier , A. Ouissa , V. H. Gessner , Catal. Sci. Technol. 2019, 9, 6808–6815.

