# Supplementary material for: From Stable PH‐Ylides to α‐Carbanionic Phosphines as Ligands for Zwitterionic Catalysts
Source: Angew Chem Int Ed Engl. 2022 Jun 14;61(30):e202203950. doi: 10.1002/anie.202203950 (PMC9401067; doi:10.1002/anie.202203950)

checkCIF/PLATON report

Structure factors have been supplied for datablock(s) JAZ-622_auto_a

THIS REPORT IS FOR GUIDANCE ONLY. IF USED AS PART OF A REVIEW PROCEDURE FOR PUBLICATION, IT SHOULD NOT REPLACE THE EXPERTISE OF AN EXPERIENCED CRYSTALLOGRAPHIC REFEREE.

No syntax errors found. CIF dictionary Interpreting this report

Datablock: JAZ-622_auto_a

```
Bond precision:   C-C = 0.0052 A          Wavelength=0.71073

Cell:           a=11.3838(2)       b=11.7213(3)      c=12.1209(3)
                alpha=111.936(2)   beta=96.909(2)    gamma=109.050(2)
Temperature:    100 K

                 Calculated                    Reported
Volume           1362.89(7)                    1362.89(6)
Space group      P -1                          P -1
Hall group       -P 1                          -P 1
Moiety formula   C23 H31 Au Cl N2 O4 P S2      C23 H31 Au Cl N2 O4 P S2
Sum formula      C23 H31 Au Cl N2 O4 P S2      C23 H31 Au Cl N2 O4 P S2
Mr               727.01                        727.00
Dx,g cm-3        1.772                         1.772
Z                2                             2
Mu (mm-1)        5.739                         5.739
F000             716.0                         716.0
F000'            713.33
h,k,lmax         16,16,17                      16,16,16
Nref             8571                          6970
Tmin,Tmax        0.346,0.532                   0.504,1.000
Tmin'            0.193

Correction method= # Reported T Limits: Tmin=0.504 Tmax=1.000
AbsCorr = GAUSSIAN

Data completeness= 0.813            Theta(max)= 30.824

R(reflections)= 0.0267( 6250)                       wR2(reflections)=
                                                    0.0603( 6970)
S = 1.046                    Npar= 322
```

The following ALERTS were generated. Each ALERT has the format
test-name_ALERT_alert-type_alert-level.
Click on the hyperlinks for more details of the test.

Alert level C

```
PLAT601_ALERT_2_C Unit Cell Contains Solvent Accessible VOIDS of .         32 Ang**3
PLAT910_ALERT_3_C Missing # of FCF Reflection(s) Below Theta(Min).          7 Note
PLAT911_ALERT_3_C Missing FCF Refl Between Thmin & STh/L=    0.600          5 Report
```

Alert level G

```
PLAT154_ALERT_1_G The s.u.'s on the Cell Angles are Equal ..(Note)      0.002 Degree
PLAT164_ALERT_4_G Nr. of Refined C-H H-Atoms in Heavy-Atom Struct.          5 Note
PLAT767_ALERT_4_G INS Embedded LIST 6 Instruction Should be LIST 4     Please Check
PLAT883_ALERT_1_G No Info/Value for _atom_sites_solution_primary .     Please Do !
PLAT912_ALERT_4_G Missing # of FCF Reflections Above STh/L=  0.600       1553 Note
PLAT941_ALERT_3_G Average HKL Measurement Multiplicity ...........        3.2 Low
PLAT978_ALERT_2_G Number C-C Bonds with Positive Residual Density.          1 Info
```

0 **ALERT level A** = Most likely a serious problem - resolve or explain
0 **ALERT level B** = A potentially serious problem, consider carefully
3 **ALERT level C** = Check. Ensure it is not caused by an omission or oversight
7 **ALERT level G** = General information/check it is not something unexpected

2 ALERT type 1 CIF construction/syntax error, inconsistent or missing data
2 ALERT type 2 Indicator that the structure model may be wrong or deficient
3 ALERT type 3 Indicator that the structure quality may be low
3 ALERT type 4 Improvement, methodology, query or suggestion
0 ALERT type 5 Informative message, check

It is advisable to attempt to resolve as many as possible of the alerts in all categories. Often the minor alerts point to easily fixed oversights, errors and omissions in your CIF or refinement strategy, so attention to these fine details can be worthwhile. In order to resolve some of the more serious problems it may be necessary to carry out additional measurements or structure refinements. However, the purpose of your study may justify the reported deviations and the more serious of these should normally be commented upon in the discussion or experimental section of a paper or in the "special_details" fields of the CIF. checkCIF was carefully designed to identify outliers and unusual parameters, but every test has its limitations and alerts that are not important in a particular case may appear. Conversely, the absence of alerts does not guarantee there are no aspects of the results needing attention. It is up to the individual to critically assess their own results and, if necessary, seek expert advice.

Publication of your CIF in IUCr journals

A basic structural check has been run on your CIF. These basic checks will be run on all CIFs submitted for publication in IUCr journals (*Acta Crystallographica*, *Journal of Applied Crystallography*, *Journal of Synchrotron Radiation*); however, if you intend to submit to *Acta Crystallographica Section C* or *E* or *IUCrData*, you should make sure that full publication checks are run on the final version of your CIF prior to submission.

Publication of your CIF in other journals

Please refer to the *Notes for Authors* of the relevant journal for any special instructions relating to CIF submission.

PLATON version of 19/02/2022; check.def file version of 19/02/2022

Datablock JAZ-622_auto_a - ellipsoid plot

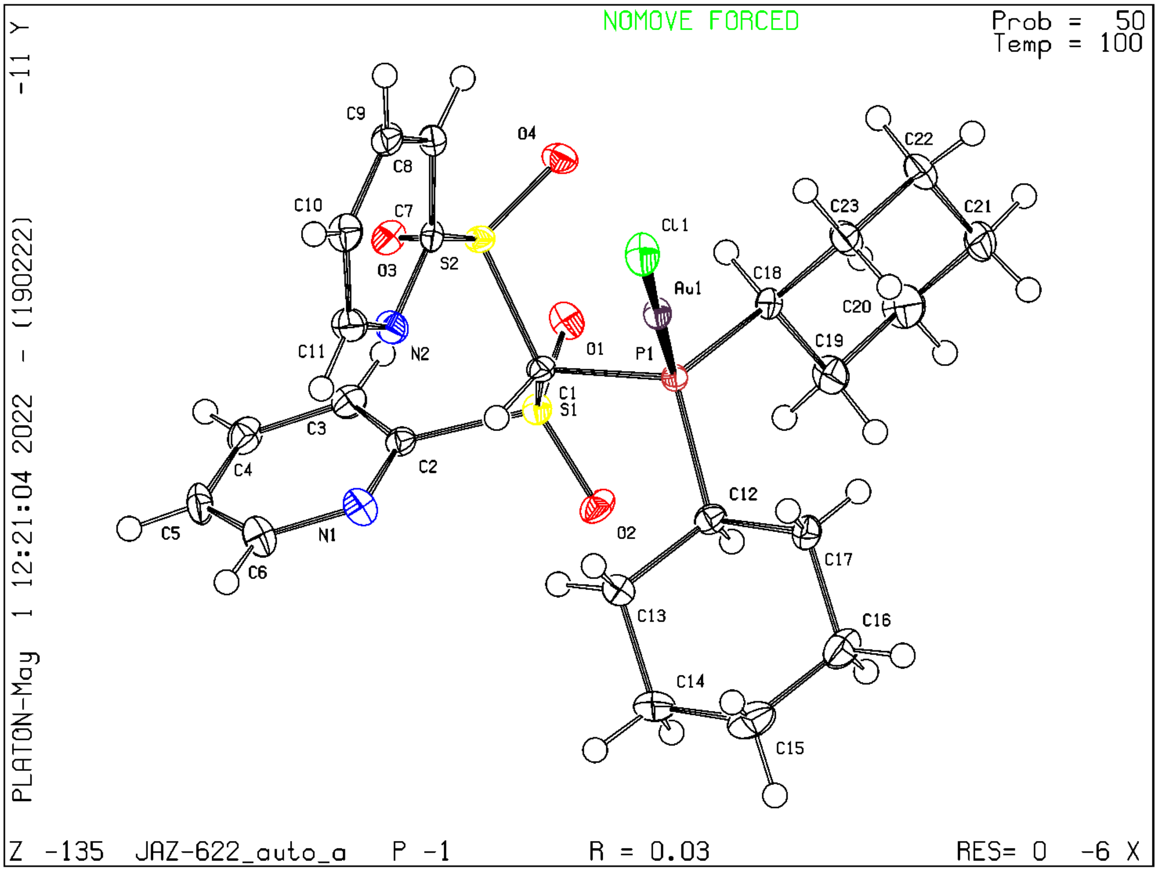

checkCIF/PLATON report

Structure factors have been supplied for datablock(s) platon_sq

THIS REPORT IS FOR GUIDANCE ONLY. IF USED AS PART OF A REVIEW PROCEDURE FOR PUBLICATION, IT SHOULD NOT REPLACE THE EXPERTISE OF AN EXPERIENCED CRYSTALLOGRAPHIC REFEREE.

No syntax errors found. CIF dictionary Interpreting this report

Datablock: platon_sq

Bond precision:	C-C = 0.0039 A		Wavelength=1.54184
Cell:	a=12.9810(2)	b=14.0310(2)	c=27.2465(4)
	alpha=90	beta=90.882(1)	gamma=90
Temperature:	100 K		

	Calculated	Reported
Volume	4961.99(13)	4961.99(13)
Space group	P 21/c	P 21/c
Hall group	-P 2ybc	-P 2ybc
Moiety formula	C41 H45 Au N2 O4 P2 S2, C4 H8 O [+ solvent]	C41 H45 Au N2 O4 P2 S2 , O C4 H8
Sum formula	C45 H53 Au N2 O5 P2 S2 [+ solvent]	C45 H53 Au N2 O5 P2 S2
Mr	1024.92	1024.92
Dx,g cm-3	1.372	1.372
Z	4	4
Mu (mm-1)	7.299	7.299
F000	2072.0	2072.0
F000'	2063.83	
h,k,lmax	15,16,32	15,16,32
Nref	8870	8799
Tmin,Tmax	0.269,0.442	0.589,1.000
Tmin'	0.135	

Correction method= # Reported T Limits: Tmin=0.589 Tmax=1.000
AbsCorr = GAUSSIAN

Data completeness= 0.992 Theta(max)= 67.080

R(reflections)= 0.0223(8532) wR2(reflections)= 0.0534(8799)

S = 1.062 Npar= 569

The following ALERTS were generated. Each ALERT has the format
test-name_ALERT_alert-type_alert-level.
Click on the hyperlinks for more details of the test.

Alert level C

PLAT911_ALERT_3_C Missing FCF Refl Between Thmin & STh/L= 0.597 72 Report

---

Alert level G

PLAT002_ALERT_2_G Number of Distance or Angle Restraints on AtSite 10 Note
PLAT003_ALERT_2_G Number of Uiso or Uij Restrained non-H Atoms ... 12 Report
PLAT042_ALERT_1_G Calc. and Reported Moiety Formula Strings Differ Please Check
PLAT175_ALERT_4_G The CIF-Embedded .res File Contains SAME Records 1 Report
PLAT187_ALERT_4_G The CIF-Embedded .res File Contains RIGU Records 2 Report
PLAT301_ALERT_3_G Main Residue Disorder ....(Resd 1) 12% Note
PLAT398_ALERT_2_G Deviating C-O-C Angle From 120 for O1_1 . 108.5 Degree
PLAT606_ALERT_4_G Solvent Accessible VOID(S) in Structure .... ! Info
PLAT720_ALERT_4_G Number of Unusual/Non-Standard Labels .... 13 Note
PLAT860_ALERT_3_G Number of Least-Squares Restraints .... 80 Note
PLAT869_ALERT_4_G ALERTS Related to the Use of SQUEEZE Suppressed ! Info
PLAT883_ALERT_1_G No Info/Value for _atom_sites_solution_primary . Please Do !
PLAT909_ALERT_3_G Percentage of I>2sig(I) Data at Theta(Max) Still 94% Note
PLAT933_ALERT_2_G Number of HKL-OMIT Records in Embedded .res File 2 Note
PLAT941_ALERT_3_G Average HKL Measurement Multiplicity .... 3.7 Low
PLAT965_ALERT_2_G The SHELXL WEIGHT Optimisation has not Converged Please Check
PLAT978_ALERT_2_G Number C-C Bonds with Positive Residual Density. 3 Info

---

0 **ALERT level A** = Most likely a serious problem - resolve or explain
0 **ALERT level B** = A potentially serious problem, consider carefully
1 **ALERT level C** = Check. Ensure it is not caused by an omission or oversight
17 **ALERT level G** = General information/check it is not something unexpected

2 ALERT type 1 CIF construction/syntax error, inconsistent or missing data
6 ALERT type 2 Indicator that the structure model may be wrong or deficient
5 ALERT type 3 Indicator that the structure quality may be low
5 ALERT type 4 Improvement, methodology, query or suggestion
0 ALERT type 5 Informative message, check

It is advisable to attempt to resolve as many as possible of the alerts in all categories. Often the minor alerts point to easily fixed oversights, errors and omissions in your CIF or refinement strategy, so attention to these fine details can be worthwhile. In order to resolve some of the more serious problems it may be necessary to carry out additional measurements or structure refinements. However, the purpose of your study may justify the reported deviations and the more serious of these should normally be commented upon in the discussion or experimental section of a paper or in the "special_details" fields of the CIF. checkCIF was carefully designed to identify outliers and unusual parameters, but every test has its limitations and alerts that are not important in a particular case may appear. Conversely, the absence of alerts does not guarantee there are no aspects of the results needing attention. It is up to the individual to critically assess their own results and, if necessary, seek expert advice.

Publication of your CIF in IUCr journals

A basic structural check has been run on your CIF. These basic checks will be run on all CIFs submitted for publication in IUCr journals (*Acta Crystallographica*, *Journal of Applied Crystallography*, *Journal of Synchrotron Radiation*); however, if you intend to submit to *Acta Crystallographica Section C* or *E* or *IUCrData*, you should make sure that full publication checks are run on the final version of your CIF prior to submission.

Publication of your CIF in other journals

Please refer to the *Notes for Authors* of the relevant journal for any special instructions relating to CIF submission.

PLATON version of 19/02/2022; check.def file version of 19/02/2022

Datablock platon_sq - ellipsoid plot

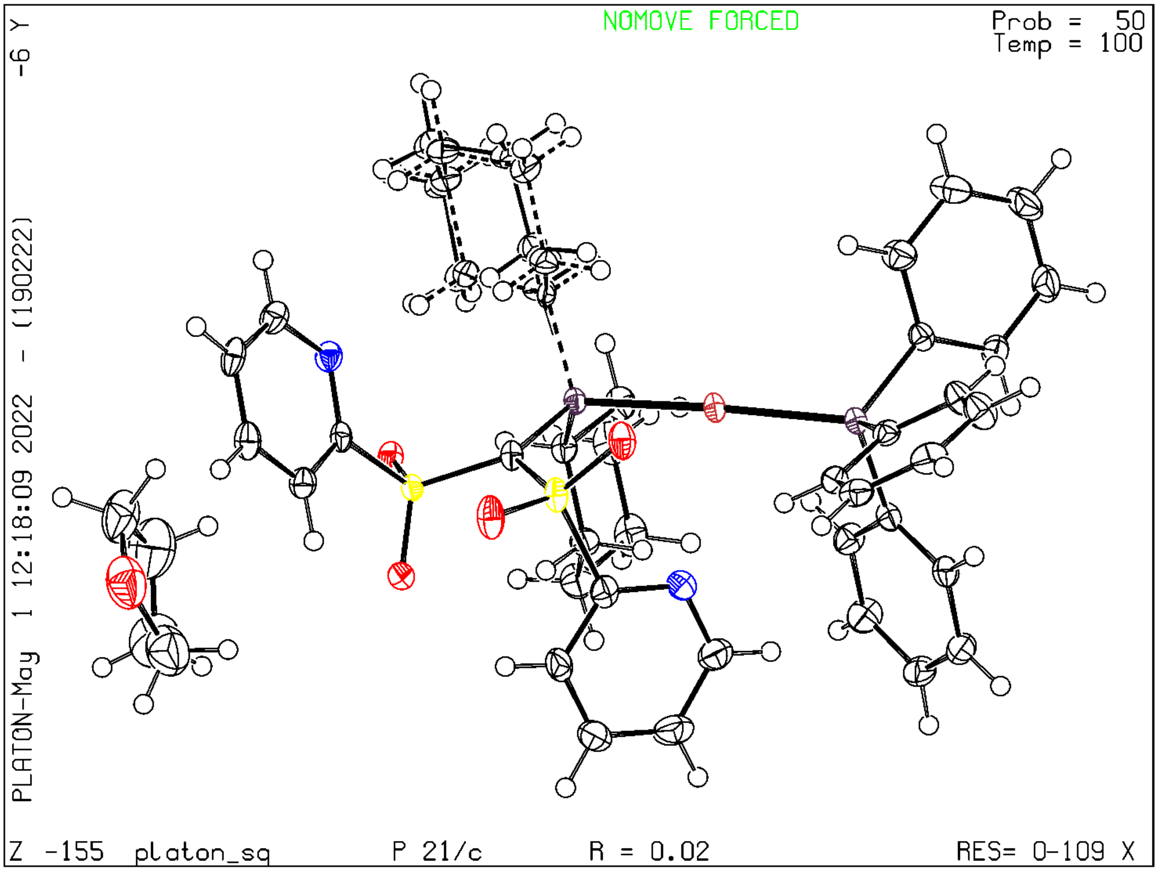

Supplement: Supplementary file 1 — Supporting Information [file ANIE-61-0-s003.pdf]
