# Supplementary material for: From Stable PH‐Ylides to α‐Carbanionic Phosphines as Ligands for Zwitterionic Catalysts
Source: Angew Chem Int Ed Engl. 2022 Jun 14;61(30):e202203950. doi: 10.1002/anie.202203950 (PMC9401067; doi:10.1002/anie.202203950)

# checkCIF/PLATON report

Structure factors have been supplied for datablock(s) ST091

THIS REPORT IS FOR GUIDANCE ONLY. IF USED AS PART OF A REVIEW PROCEDURE FOR PUBLICATION, IT SHOULD NOT REPLACE THE EXPERTISE OF AN EXPERIENCED CRYSTALLOGRAPHIC REFEREE.

No syntax errors found.     CIF dictionary     Interpreting this report

# Datablock: ST091

---

```
Bond precision:   C-C = 0.0020 A          Wavelength=1.54184

Cell:              a=15.5871(7)      b=11.6942(5)        c=8.1780(3)
                   alpha=90          beta=91.832(4)      gamma=90
Temperature:       100 K

                 Calculated                  Reported
Volume           1489.91(11)                 1489.90(11)
Space group      C 2/c                       C 2/c
Hall group       -C 2yc                      -C 2yc
Moiety formula   C15 H16 O4 S2               C15 H16 O4 S2
Sum formula      C15 H16 O4 S2               C15 H16 O4 S2
Mr               324.40                      324.40
Dx,g cm-3        1.446                       1.446
Z                4                           4
Mu (mm-1)        3.360                       3.360
F000             680.0                       680.0
F000'            684.50
h,k,lmax         19,14,10                    19,14,10
Nref             1524                        1524
Tmin,Tmax        0.542,0.932                 0.309,1.000
Tmin'            0.325

Correction method= # Reported T Limits: Tmin=0.309 Tmax=1.000
AbsCorr = GAUSSIAN

Data completeness= 1.000           Theta(max)= 74.461

R(reflections)= 0.0316( 1428)                          wR2(reflections)=
                                                       0.0931( 1524)
S = 1.069                  Npar= 97
```

The following ALERTS were generated. Each ALERT has the format
**test-name_ALERT_alert-type_alert-level.**
Click on the hyperlinks for more details of the test.

### Alert level G

```
PLAT300_ALERT_4_G Atom Site Occupancy of H1A        Constrained at        0.5 Check
PLAT300_ALERT_4_G Atom Site Occupancy of H1B        Constrained at        0.5 Check
PLAT883_ALERT_1_G No Info/Value for _atom_sites_solution_primary .     Please Do !
PLAT941_ALERT_3_G Average HKL Measurement Multiplicity ...........        3.8 Low
PLAT965_ALERT_2_G The SHELXL WEIGHT Optimisation has not Converged     Please Check
PLAT978_ALERT_2_G Number C-C Bonds with Positive Residual Density.          7 Info
```

0 **ALERT level A** = Most likely a serious problem - resolve or explain
0 **ALERT level B** = A potentially serious problem, consider carefully
0 **ALERT level C** = Check. Ensure it is not caused by an omission or oversight
6 **ALERT level G** = General information/check it is not something unexpected

1 ALERT type 1 CIF construction/syntax error, inconsistent or missing data
2 ALERT type 2 Indicator that the structure model may be wrong or deficient
1 ALERT type 3 Indicator that the structure quality may be low
2 ALERT type 4 Improvement, methodology, query or suggestion
0 ALERT type 5 Informative message, check

# checkCIF publication errors

### Alert level G

PUBL017_ALERT_1_G The _publ_section_references section is missing or
empty.

0 **ALERT level A** = Data missing that is essential or data in wrong format
1 **ALERT level G** = General alerts. Data that may be required is missing

**Publication of your CIF**

You should attempt to resolve as many as possible of the alerts in all categories. Often the minor alerts point to easily fixed oversights, errors and omissions in your CIF or refinement strategy, so attention to these fine details can be worthwhile. In order to resolve some of the more serious problems it may be necessary to carry out additional measurements or structure refinements. However, the nature of your study may justify the reported deviations from journal submission requirements and the more serious of these should be commented upon in the discussion or experimental section of a paper or in the "special_details" fields of the CIF. *checkCIF* was carefully designed to identify outliers and unusual parameters, but every test has its limitations and alerts that are not important in a particular case may appear. Conversely, the absence of alerts does not guarantee there are no aspects of the results needing attention. It is up to the individual to critically assess their own results and, if necessary, seek expert advice.

If you wish to submit your CIF for publication in Acta Crystallographica Section C or E, you should upload your CIF via the web. If you wish to submit your CIF for publication in IUCrData you should upload your CIF via the web. If your CIF is to form part of a submission to another IUCr journal, you will be asked, either during electronic submission or by the Co-editor handling your paper, to upload your CIF via our web site.

---

**PLATON version of 18/12/2021; check.def file version of 18/12/2021**

**Datablock ST091** - ellipsoid plot

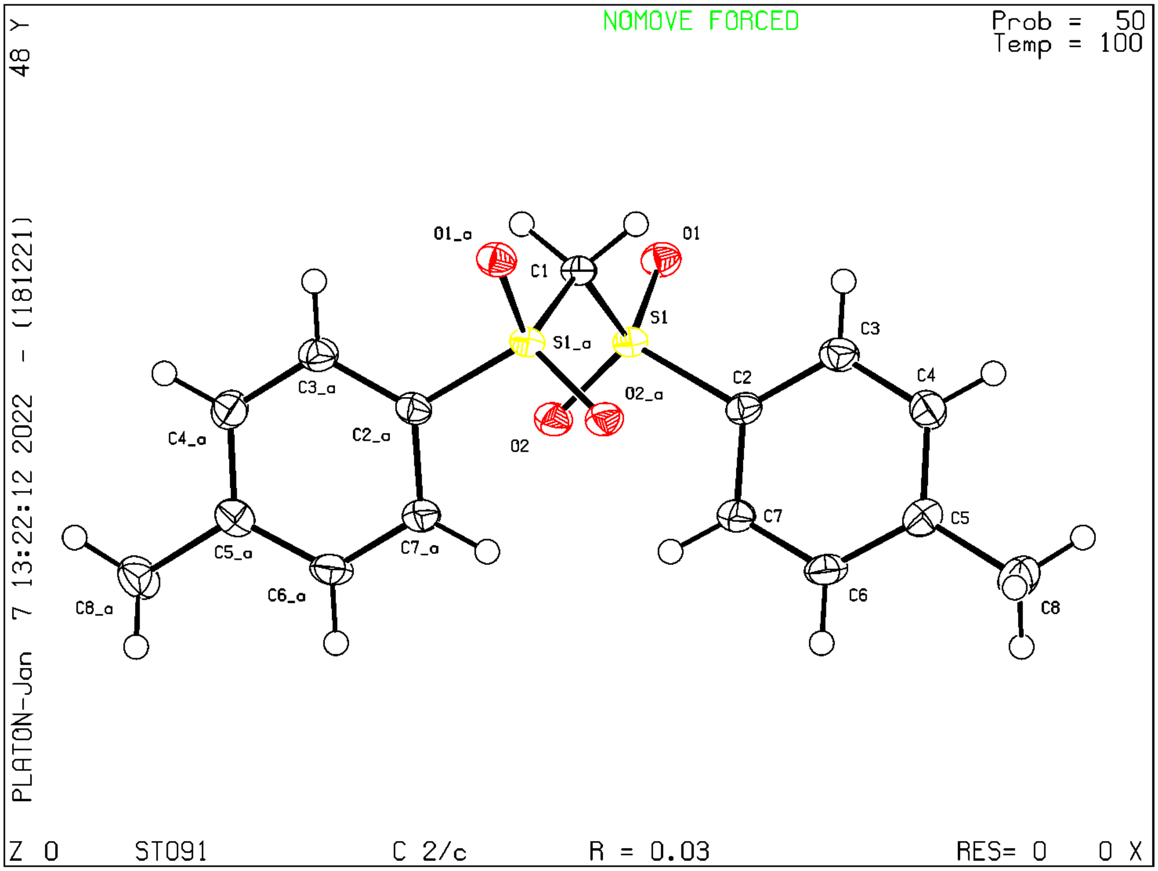

# checkCIF/PLATON report

Structure factors have been supplied for datablock(s) sad

THIS REPORT IS FOR GUIDANCE ONLY. IF USED AS PART OF A REVIEW PROCEDURE FOR PUBLICATION, IT SHOULD NOT REPLACE THE EXPERTISE OF AN EXPERIENCED CRYSTALLOGRAPHIC REFEREE.

No syntax errors found. CIF dictionary Interpreting this report

## Datablock: sad

---

| | | | |
|---|---|---|---|
| Bond precision: | C-C = 0.0035 A | | Wavelength=0.71073 |
| Cell: | a=15.6736(9) | b=5.3657(3) | c=7.5242(4) |
| | alpha=90 | beta=108.337(1) | gamma=90 |
| Temperature: | 100 K | | |

| | Calculated | Reported |
|---|---|---|
| Volume | 600.65(6) | 600.65(6) |
| Space group | C 2 | C 2 |
| Hall group | C 2y | C 2y |
| Moiety formula | C11 H10 N2 O4 S2 | C11 H10 N2 O4 S2 |
| Sum formula | C11 H10 N2 O4 S2 | C11 H10 N2 O4 S2 |
| Mr | 298.33 | 298.33 |
| Dx,g cm-3 | 1.650 | 1.649 |
| Z | 2 | 2 |
| Mu (mm-1) | 0.455 | 0.455 |
| F000 | 308.0 | 308.0 |
| F000’ | 308.64 | |
| h,k,lmax | 18,6,8 | 18,6,8 |
| Nref | 1050[ 586] | 1052 |
| Tmin,Tmax | 0.882,0.930 | 0.712,0.746 |
| Tmin’ | 0.849 | |

Correction method= # Reported T Limits: Tmin=0.712 Tmax=0.746
AbsCorr = EMPIRICAL

Data completeness= 1.80/1.00 Theta(max)= 24.991

R(reflections)= 0.0166( 1044) wR2(reflections)= 0.0441( 1052)

S = 1.105 Npar= 87

The following ALERTS were generated. Each ALERT has the format
**test-name_ALERT_alert-type_alert-level.**
Click on the hyperlinks for more details of the test.

### Alert level C

```
PLAT089_ALERT_3_C Poor Data / Parameter Ratio (Zmax < 18) ........       6.75 Note
PLAT761_ALERT_1_C CIF Contains no X-H Bonds ......................     Please Check
PLAT762_ALERT_1_C CIF Contains no X-Y-H or H-Y-H Angles ..........     Please Check
```

### Alert level G

```
PLAT033_ALERT_4_G Flack x Value Deviates > 3.0 * sigma from Zero .      0.130 Note
PLAT128_ALERT_4_G Alternate Setting for Input Space Group  C2             I2 Note
PLAT300_ALERT_4_G Atom Site Occupancy of H1A       Constrained at        0.5 Check
PLAT300_ALERT_4_G Atom Site Occupancy of H1B       Constrained at        0.5 Check
PLAT883_ALERT_1_G No Info/Value for _atom_sites_solution_primary .     Please Do !
PLAT909_ALERT_3_G Percentage of I>2sig(I) Data at Theta(Max) Still       100% Note
PLAT967_ALERT_5_G Note: Two-Theta Cutoff Value in Embedded .res ..       50.0 Degree
PLAT978_ALERT_2_G Number C-C Bonds with Positive Residual Density.          2 Info
```

0 **ALERT level A** = Most likely a serious problem - resolve or explain
0 **ALERT level B** = A potentially serious problem, consider carefully
3 **ALERT level C** = Check. Ensure it is not caused by an omission or oversight
8 **ALERT level G** = General information/check it is not something unexpected

3 ALERT type 1 CIF construction/syntax error, inconsistent or missing data
1 ALERT type 2 Indicator that the structure model may be wrong or deficient
2 ALERT type 3 Indicator that the structure quality may be low
4 ALERT type 4 Improvement, methodology, query or suggestion
1 ALERT type 5 Informative message, check

# checkCIF publication errors

### Alert level G

PUBL017_ALERT_1_G The _publ_section_references section is missing or
empty.

0 **ALERT level A** = Data missing that is essential or data in wrong format
1 **ALERT level G** = General alerts. Data that may be required is missing

**Publication of your CIF**

You should attempt to resolve as many as possible of the alerts in all categories. Often the minor alerts point to easily fixed oversights, errors and omissions in your CIF or refinement strategy, so attention to these fine details can be worthwhile. In order to resolve some of the more serious problems it may be necessary to carry out additional measurements or structure refinements. However, the nature of your study may justify the reported deviations from journal submission requirements and the more serious of these should be commented upon in the discussion or experimental section of a paper or in the "special_details" fields of the CIF. *checkCIF* was carefully designed to identify outliers and unusual parameters, but every test has its limitations and alerts that are not important in a particular case may appear. Conversely, the absence of alerts does not guarantee there are no aspects of the results needing attention. It is up to the individual to critically assess their own results and, if necessary, seek expert advice.

If you wish to submit your CIF for publication in Acta Crystallographica Section C or E, you should upload your CIF via the web. If you wish to submit your CIF for publication in IUCrData you should upload your CIF via the web. If your CIF is to form part of a submission to another IUCr journal, you will be asked, either during electronic submission or by the Co-editor handling your paper, to upload your CIF via our web site.

---

**PLATON version of 18/12/2021; check.def file version of 18/12/2021**

**Datablock sad** - ellipsoid plot

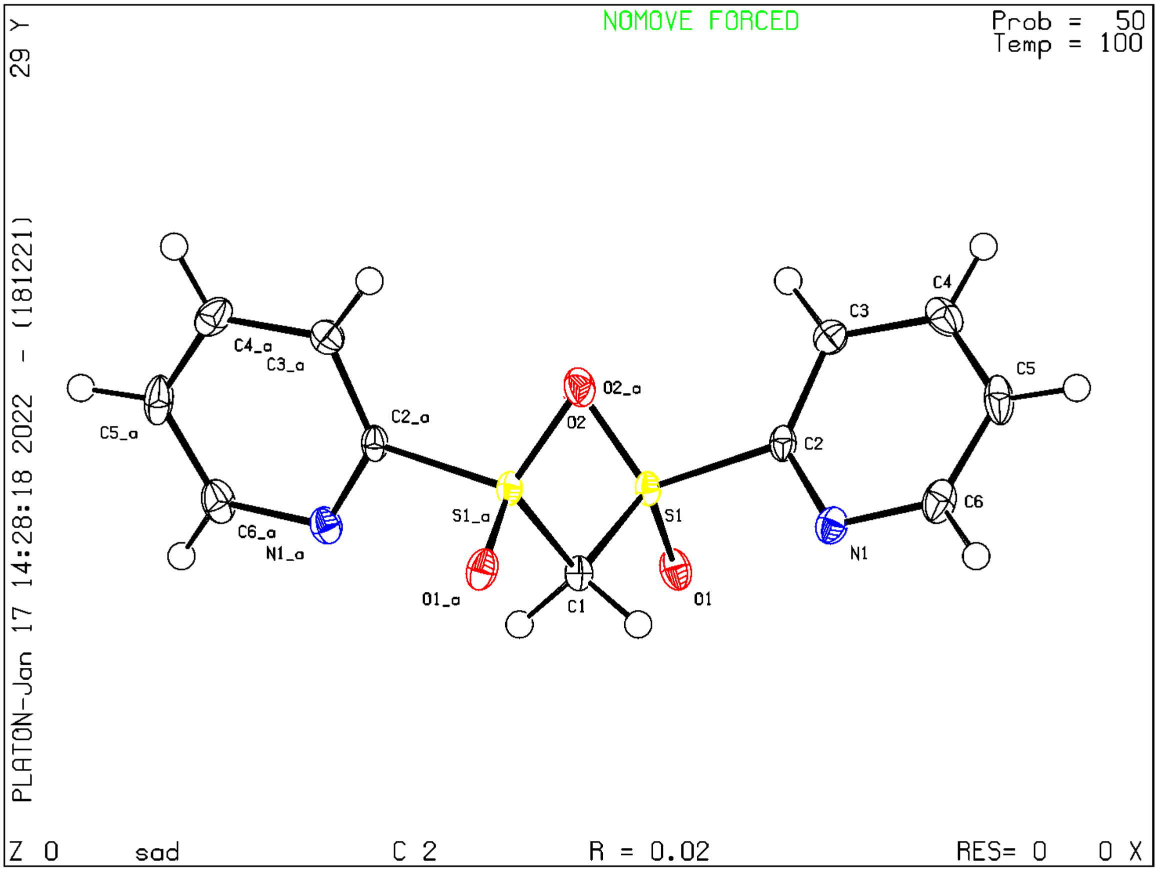

# checkCIF/PLATON report

Structure factors have been supplied for datablock(s) KSF-1673_4_a

THIS REPORT IS FOR GUIDANCE ONLY. IF USED AS PART OF A REVIEW PROCEDURE FOR PUBLICATION, IT SHOULD NOT REPLACE THE EXPERTISE OF AN EXPERIENCED CRYSTALLOGRAPHIC REFEREE.

No syntax errors found. CIF dictionary Interpreting this report

## Datablock: KSF-1673_4_a

---

```
Bond precision:   C-C = 0.0038 A          Wavelength=1.54184

Cell:           a=7.9090(1)        b=11.2437(1)       c=15.9851(1)
                alpha=96.994(1)    beta=102.424(1)    gamma=91.521(1)
Temperature:    100 K

                 Calculated                   Reported
Volume           1375.84(2)                   1375.84(2)
Space group      P 1                          P 1
Hall group       P 1                          P 1
Moiety formula   C25 H35 Na O9 S2             C25 H35 Na O9 S2
Sum formula      C25 H35 Na O9 S2             C25 H35 Na O9 S2
Mr               566.64                       566.64
Dx,g cm-3        1.368                        1.368
Z                2                            2
Mu (mm-1)        2.338                        2.338
F000             600.0                        600.0
F000'            603.33
h,k,lmax         9,13,19                      9,13,19
Nref             9822[ 4911]                  9587
Tmin,Tmax        0.799,0.911                  0.760,1.000
Tmin'            0.755

Correction method= # Reported T Limits: Tmin=0.760 Tmax=1.000
AbsCorr = GAUSSIAN

Data completeness= 1.95/0.98       Theta(max)= 67.073

R(reflections)= 0.0244( 9471)          wR2(reflections)= 0.0631( 9587)

S = 1.034                  Npar= 698
```

The following ALERTS were generated. Each ALERT has the format
**test-name_ALERT_alert-type_alert-level.**
Click on the hyperlinks for more details of the test.

**Alert level C**

```
PLAT089_ALERT_3_C Poor Data / Parameter Ratio (Zmax < 18) ........       7.03 Note
PLAT911_ALERT_3_C Missing FCF Refl Between Thmin & STh/L=    0.597          4 Report
```

**Alert level G**

```
PLAT153_ALERT_1_G The s.u.'s on the Cell Axes   are Equal ..(Note)     0.0001 Ang.
PLAT154_ALERT_1_G The s.u.'s on the Cell Angles are Equal ..(Note)      0.001 Degree
PLAT301_ALERT_3_G Main Residue  Disorder .............(Resd  1  )          5% Note
PLAT410_ALERT_2_G Short Intra H...H Contact  H7        ..H25D     .       2.14 Ang.
                                                  x,y,z  =      1_555 Check
PLAT410_ALERT_2_G Short Intra H...H Contact  H16B      ..H25A     .       2.13 Ang.
                                                  x,y,z  =      1_555 Check
PLAT410_ALERT_2_G Short Intra H...H Contact  H23A      ..H24C     .       1.90 Ang.
                                                  x,y,z  =      1_555 Check
PLAT791_ALERT_4_G Model has Chirality at S1          (Sohnke SpGr)          S Verify
PLAT791_ALERT_4_G Model has Chirality at S2          (Sohnke SpGr)          R Verify
PLAT791_ALERT_4_G Model has Chirality at S3          (Sohnke SpGr)          S Verify
PLAT791_ALERT_4_G Model has Chirality at S4          (Sohnke SpGr)          R Verify
PLAT909_ALERT_3_G Percentage of I>2sig(I) Data at Theta(Max) Still        97% Note
PLAT978_ALERT_2_G Number C-C Bonds with Positive Residual Density.          8 Info
```

0 **ALERT level A** = Most likely a serious problem - resolve or explain
0 **ALERT level B** = A potentially serious problem, consider carefully
2 **ALERT level C** = Check. Ensure it is not caused by an omission or oversight
12 **ALERT level G** = General information/check it is not something unexpected

2 ALERT type 1 CIF construction/syntax error, inconsistent or missing data
4 ALERT type 2 Indicator that the structure model may be wrong or deficient
4 ALERT type 3 Indicator that the structure quality may be low
4 ALERT type 4 Improvement, methodology, query or suggestion
0 ALERT type 5 Informative message, check

# checkCIF publication errors

**Alert level G**

PUBL017_ALERT_1_G The _publ_section_references section is missing or
empty.

0 **ALERT level A** = Data missing that is essential or data in wrong format
1 **ALERT level G** = General alerts. Data that may be required is missing

**Publication of your CIF**

You should attempt to resolve as many as possible of the alerts in all categories. Often the minor alerts point to easily fixed oversights, errors and omissions in your CIF or refinement strategy, so attention to these fine details can be worthwhile. In order to resolve some of the more serious problems it may be necessary to carry out additional measurements or structure refinements. However, the nature of your study may justify the reported deviations from journal submission requirements and the more serious of these should be commented upon in the discussion or experimental section of a paper or in the "special_details" fields of the CIF. *checkCIF* was carefully designed to identify outliers and unusual parameters, but every test has its limitations and alerts that are not important in a particular case may appear. Conversely, the absence of alerts does not guarantee there are no aspects of the results needing attention. It is up to the individual to critically assess their own results and, if necessary, seek expert advice.

If you wish to submit your CIF for publication in Acta Crystallographica Section C or E, you should upload your CIF via the web. If you wish to submit your CIF for publication in IUCrData you should upload your CIF via the web. If your CIF is to form part of a submission to another IUCr journal, you will be asked, either during electronic submission or by the Co-editor handling your paper, to upload your CIF via our web site.

---

**PLATON version of 20/01/2022; check.def file version of 19/01/2022**

**Datablock KSF-1673_4_a** - ellipsoid plot

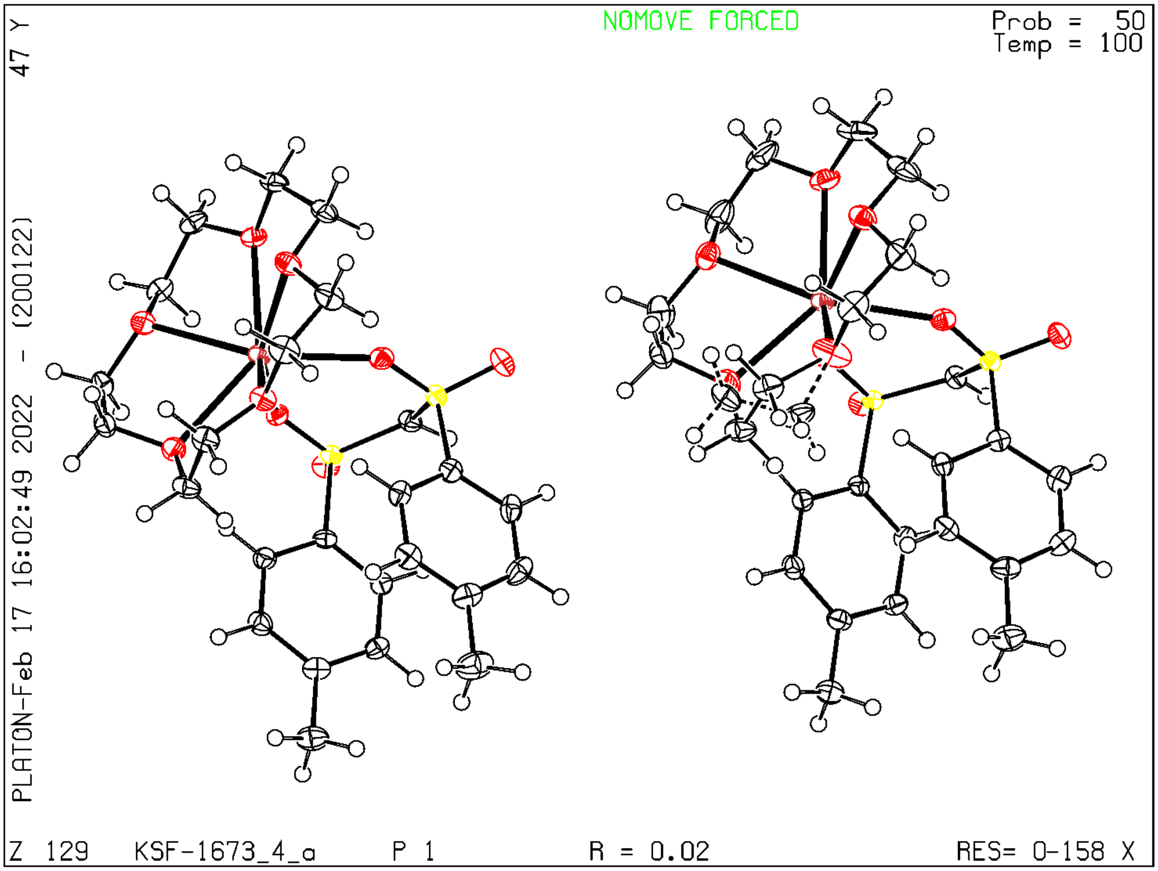

# checkCIF/PLATON report

Structure factors have been supplied for datablock(s) sad

THIS REPORT IS FOR GUIDANCE ONLY. IF USED AS PART OF A REVIEW PROCEDURE FOR PUBLICATION, IT SHOULD NOT REPLACE THE EXPERTISE OF AN EXPERIENCED CRYSTALLOGRAPHIC REFEREE.

No syntax errors found. CIF dictionary Interpreting this report

## Datablock: sad

---

```
Bond precision:   C-C = 0.0024 A          Wavelength=0.71073

Cell:            a=28.7858(17)    b=9.9519(6)          c=18.6156(11)
                 alpha=90         beta=113.5476(16)    gamma=90
Temperature:     100 K

                 Calculated                    Reported
Volume           4888.8(5)                     4888.8(5)
Space group      C 2/c                         C 2/c
Hall group       -C 2yc                        -C 2yc
Moiety formula   C21 H29 N2 Na O9 S2           C21 H29 N2 Na O9 S2
Sum formula      C21 H29 N2 Na O9 S2           C21 H29 N2 Na O9 S2
Mr               540.57                        540.57
Dx,g cm-3        1.469                         1.469
Z                8                             8
Mu (mm-1)        0.290                         0.290
F000             2272.0                        2272.0
F000'            2275.36
h,k,lmax         34,11,22                      34,11,22
Nref             4300                          4300
Tmin,Tmax        0.903,0.957                   0.711,0.746
Tmin'            0.903

Correction method= # Reported T Limits: Tmin=0.711 Tmax=0.746
AbsCorr = EMPIRICAL

Data completeness= 1.000            Theta(max)= 24.998

R(reflections)= 0.0281( 3996)                          wR2(reflections)=
                                                       0.0719( 4300)
S = 1.024                    Npar= 339
```

The following ALERTS were generated. Each ALERT has the format
**test-name_ALERT_alert-type_alert-level.**
Click on the hyperlinks for more details of the test.

**Alert level G**

```
PLAT083_ALERT_2_G SHELXL Second Parameter in WGHT  Unusually Large       7.54 Why ?
PLAT128_ALERT_4_G Alternate Setting for Input Space Group     C2/c       I2/a Note
PLAT230_ALERT_2_G Hirshfeld Test Diff for    O8       --C15A     .        5.6 s.u.
PLAT230_ALERT_2_G Hirshfeld Test Diff for    O9A      --C14B     .        6.8 s.u.
PLAT301_ALERT_3_G Main Residue  Disorder .............(Resd  1  )          6% Note
PLAT410_ALERT_2_G Short Intra H...H Contact  H13A     ..H14C     .       2.00 Ang.
                                                   x,y,z  =      1_555 Check
PLAT793_ALERT_4_G Model has Chirality at S1          (Centro SPGR)          R Verify
PLAT793_ALERT_4_G Model has Chirality at S2          (Centro SPGR)          S Verify
PLAT883_ALERT_1_G No Info/Value for _atom_sites_solution_primary .     Please Do !
PLAT909_ALERT_3_G Percentage of I>2sig(I) Data at Theta(Max) Still        85% Note
PLAT967_ALERT_5_G Note: Two-Theta Cutoff Value in Embedded .res ..       50.0 Degree
PLAT978_ALERT_2_G Number C-C Bonds with Positive Residual Density.          9 Info
```

0 **ALERT level A** = Most likely a serious problem - resolve or explain
0 **ALERT level B** = A potentially serious problem, consider carefully
0 **ALERT level C** = Check. Ensure it is not caused by an omission or oversight
12 **ALERT level G** = General information/check it is not something unexpected

1 ALERT type 1 CIF construction/syntax error, inconsistent or missing data
5 ALERT type 2 Indicator that the structure model may be wrong or deficient
2 ALERT type 3 Indicator that the structure quality may be low
3 ALERT type 4 Improvement, methodology, query or suggestion
1 ALERT type 5 Informative message, check

# checkCIF publication errors

**Alert level G**

PUBL017_ALERT_1_G The _publ_section_references section is missing or
empty.

0 **ALERT level A** = Data missing that is essential or data in wrong format
1 **ALERT level G** = General alerts. Data that may be required is missing

**Publication of your CIF**

You should attempt to resolve as many as possible of the alerts in all categories. Often the minor alerts point to easily fixed oversights, errors and omissions in your CIF or refinement strategy, so attention to these fine details can be worthwhile. In order to resolve some of the more serious problems it may be necessary to carry out additional measurements or structure refinements. However, the nature of your study may justify the reported deviations from journal submission requirements and the more serious of these should be commented upon in the discussion or experimental section of a paper or in the "special_details" fields of the CIF. *checkCIF* was carefully designed to identify outliers and unusual parameters, but every test has its limitations and alerts that are not important in a particular case may appear. Conversely, the absence of alerts does not guarantee there are no aspects of the results needing attention. It is up to the individual to critically assess their own results and, if necessary, seek expert advice.

If you wish to submit your CIF for publication in Acta Crystallographica Section C or E, you should upload your CIF via the web. If you wish to submit your CIF for publication in IUCrData you should upload your CIF via the web. If your CIF is to form part of a submission to another IUCr journal, you will be asked, either during electronic submission or by the Co-editor handling your paper, to upload your CIF via our web site.

---

**PLATON version of 18/12/2021; check.def file version of 18/12/2021**

**Datablock sad** - ellipsoid plot

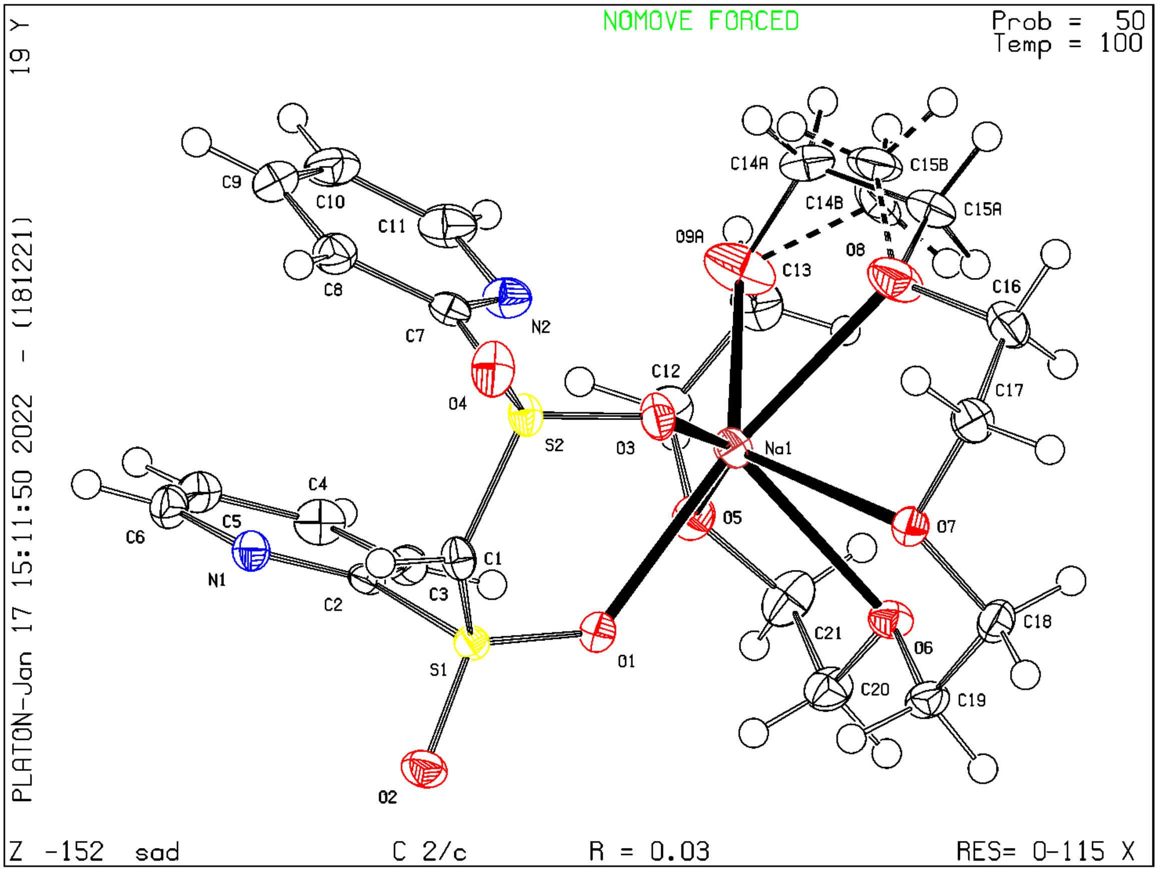

# checkCIF/PLATON report

Structure factors have been supplied for datablock(s) JAZ014_a

THIS REPORT IS FOR GUIDANCE ONLY. IF USED AS PART OF A REVIEW PROCEDURE FOR PUBLICATION, IT SHOULD NOT REPLACE THE EXPERTISE OF AN EXPERIENCED CRYSTALLOGRAPHIC REFEREE.

No syntax errors found. CIF dictionary Interpreting this report

# Datablock: JAZ014_a

---

| | | | |
|---|---|---|---|
| Bond precision: | C-C = 0.0026 A | | Wavelength=1.54184 |
| Cell: | a=13.4340(3) | b=9.3182(2) | c=18.9262(5) |
| | alpha=90 | beta=93.188(2) | gamma=90 |
| Temperature: | 100 K | | |

| | Calculated | Reported |
|---|---|---|
| Volume | 2365.53(10) | 2365.53(10) |
| Space group | P 21/n | P 21/n |
| Hall group | -P 2yn | -P 2yn |
| Moiety formula | C27 H25 O4 P S2 | C27 H25 O4 P S2 |
| Sum formula | C27 H25 O4 P S2 | C27 H25 O4 P S2 |
| Mr | 508.56 | 508.56 |
| Dx,g cm-3 | 1.428 | 1.428 |
| Z | 4 | 4 |
| Mu (mm-1) | 2.956 | 2.956 |
| F000 | 1064.0 | 1064.0 |
| F000' | 1070.51 | |
| h,k,lmax | 17,11,23 | 16,11,23 |
| Nref | 5029 | 4879 |
| Tmin,Tmax | 0.660,0.762 | 0.544,1.000 |
| Tmin' | 0.590 | |

Correction method= # Reported T Limits: Tmin=0.544 Tmax=1.000
AbsCorr = GAUSSIAN

Data completeness= 0.970 Theta(max)= 77.545

R(reflections)= 0.0380( 4747) wR2(reflections)= 0.1107( 4879)

S = 1.073 Npar= 309

The following ALERTS were generated. Each ALERT has the format
**test-name_ALERT_alert-type_alert-level.**
Click on the hyperlinks for more details of the test.

### Alert level C

```
PLAT761_ALERT_1_C CIF Contains no X-H Bonds ......................     Please Check
PLAT762_ALERT_1_C CIF Contains no X-Y-H or H-Y-H Angles ..........     Please Check
```

### Alert level G

```
PLAT883_ALERT_1_G No Info/Value for _atom_sites_solution_primary .     Please Do !
PLAT912_ALERT_4_G Missing # of FCF Reflections Above STh/L=  0.600        148 Note
PLAT941_ALERT_3_G Average HKL Measurement Multiplicity ...........        3.5 Low
PLAT965_ALERT_2_G The SHELXL WEIGHT Optimisation has not Converged     Please Check
PLAT978_ALERT_2_G Number C-C Bonds with Positive Residual Density.         12 Info
```

0 **ALERT level A** = Most likely a serious problem - resolve or explain
0 **ALERT level B** = A potentially serious problem, consider carefully
2 **ALERT level C** = Check. Ensure it is not caused by an omission or oversight
5 **ALERT level G** = General information/check it is not something unexpected

3 ALERT type 1 CIF construction/syntax error, inconsistent or missing data
2 ALERT type 2 Indicator that the structure model may be wrong or deficient
1 ALERT type 3 Indicator that the structure quality may be low
1 ALERT type 4 Improvement, methodology, query or suggestion
0 ALERT type 5 Informative message, check

# checkCIF publication errors

### Alert level G

PUBL017_ALERT_1_G The _publ_section_references section is missing or
empty.

0 **ALERT level A** = Data missing that is essential or data in wrong format
1 **ALERT level G** = General alerts. Data that may be required is missing

**Publication of your CIF**

You should attempt to resolve as many as possible of the alerts in all categories. Often the minor alerts point to easily fixed oversights, errors and omissions in your CIF or refinement strategy, so attention to these fine details can be worthwhile. In order to resolve some of the more serious problems it may be necessary to carry out additional measurements or structure refinements. However, the nature of your study may justify the reported deviations from journal submission requirements and the more serious of these should be commented upon in the discussion or experimental section of a paper or in the "special_details" fields of the CIF. *checkCIF* was carefully designed to identify outliers and unusual parameters, but every test has its limitations and alerts that are not important in a particular case may appear. Conversely, the absence of alerts does not guarantee there are no aspects of the results needing attention. It is up to the individual to critically assess their own results and, if necessary, seek expert advice.

If you wish to submit your CIF for publication in Acta Crystallographica Section C or E, you should upload your CIF via the web. If you wish to submit your CIF for publication in IUCrData you should upload your CIF via the web. If your CIF is to form part of a submission to another IUCr journal, you will be asked, either during electronic submission or by the Co-editor handling your paper, to upload your CIF via our web site.

---

**PLATON version of 20/01/2022; check.def file version of 19/01/2022**

**Datablock JAZ014_a** - ellipsoid plot

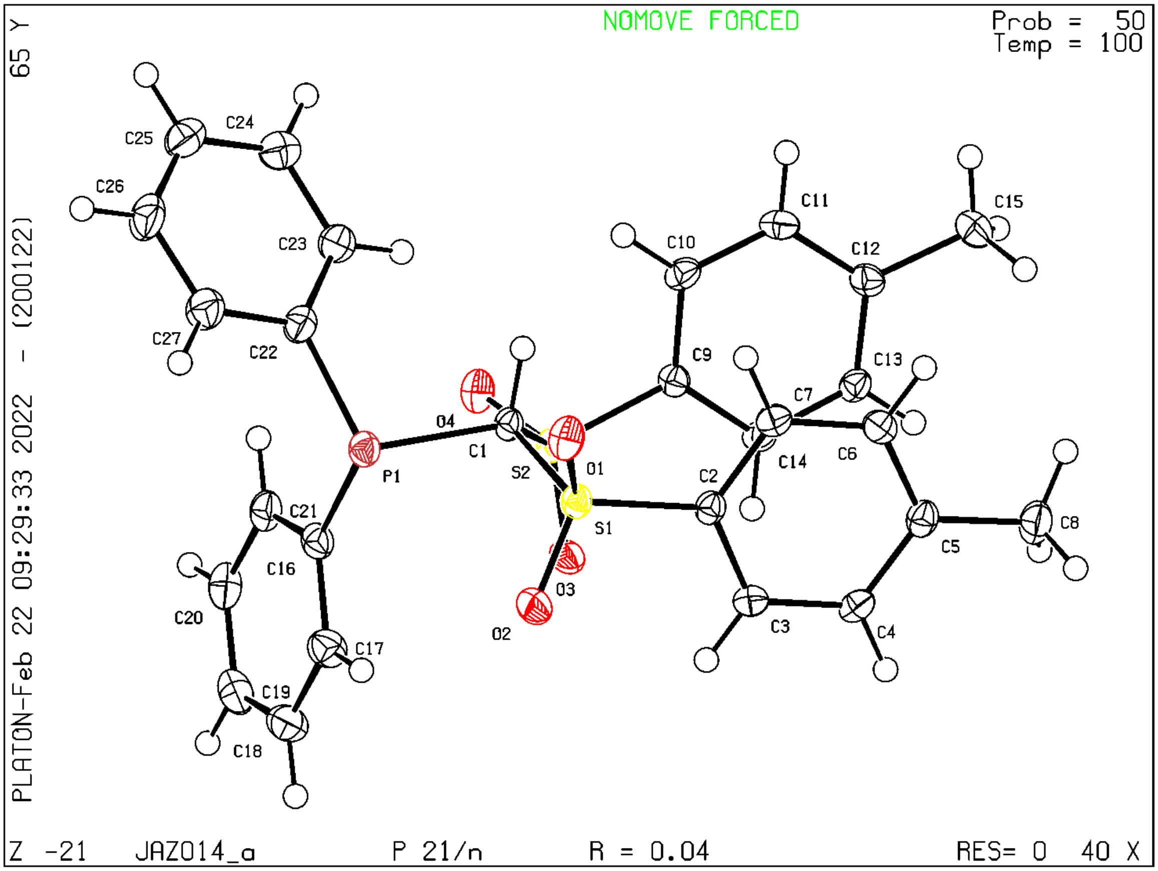

# checkCIF/PLATON report

Structure factors have been supplied for datablock(s) ST098

THIS REPORT IS FOR GUIDANCE ONLY. IF USED AS PART OF A REVIEW PROCEDURE FOR PUBLICATION, IT SHOULD NOT REPLACE THE EXPERTISE OF AN EXPERIENCED CRYSTALLOGRAPHIC REFEREE.

No syntax errors found. CIF dictionary Interpreting this report

# Datablock: ST098

---

| | | | |
|---|---|---|---|
| Bond precision: | C-C = 0.0019 A | | Wavelength=1.54184 |
| Cell: | a=11.0689(15) | b=11.8156(6) | c=20.5881(14) |
| | alpha=90 | beta=103.469(9) | gamma=90 |
| Temperature: | 100 K | | |

| | Calculated | Reported |
|---|---|---|
| Volume | 2618.6(4) | 2618.6(4) |
| Space group | P 21/n | P 21/n |
| Hall group | -P 2yn | -P 2yn |
| Moiety formula | C27 H37 O4 P S2 | C27 H37 O4 P S2 |
| Sum formula | C27 H37 O4 P S2 | C27 H37 O4 P S2 |
| Mr | 520.66 | 520.65 |
| Dx,g cm-3 | 1.321 | 1.321 |
| Z | 4 | 4 |
| Mu (mm-1) | 2.671 | 2.671 |
| F000 | 1112.0 | 1112.0 |
| F000' | 1118.51 | |
| h,k,lmax | 13,14,25 | 13,14,25 |
| Nref | 5371 | 5363 |
| Tmin,Tmax | 0.694,0.821 | 0.687,1.000 |
| Tmin' | 0.630 | |

Correction method= # Reported T Limits: Tmin=0.687 Tmax=1.000
AbsCorr = GAUSSIAN

Data completeness= 0.999 Theta(max)= 74.754

R(reflections)= 0.0288( 5011) wR2(reflections)= 0.0774( 5363)

S = 1.070 Npar= 418

The following ALERTS were generated. Each ALERT has the format
**test-name_ALERT_alert-type_alert-level.**
Click on the hyperlinks for more details of the test.

### Alert level G

PLAT883_ALERT_1_G No Info/Value for _atom_sites_solution_primary . Please Do !
PLAT912_ALERT_4_G Missing # of FCF Reflections Above STh/L= 0.600 9 Note
PLAT978_ALERT_2_G Number C-C Bonds with Positive Residual Density. 14 Info

0 **ALERT level A** = Most likely a serious problem - resolve or explain
0 **ALERT level B** = A potentially serious problem, consider carefully
0 **ALERT level C** = Check. Ensure it is not caused by an omission or oversight
3 **ALERT level G** = General information/check it is not something unexpected

1 ALERT type 1 CIF construction/syntax error, inconsistent or missing data
1 ALERT type 2 Indicator that the structure model may be wrong or deficient
0 ALERT type 3 Indicator that the structure quality may be low
1 ALERT type 4 Improvement, methodology, query or suggestion
0 ALERT type 5 Informative message, check

# checkCIF publication errors

### Alert level G

PUBL017_ALERT_1_G The _publ_section_references section is missing or empty.

0 **ALERT level A** = Data missing that is essential or data in wrong format
1 **ALERT level G** = General alerts. Data that may be required is missing

**Publication of your CIF**

You should attempt to resolve as many as possible of the alerts in all categories. Often the minor alerts point to easily fixed oversights, errors and omissions in your CIF or refinement strategy, so attention to these fine details can be worthwhile. In order to resolve some of the more serious problems it may be necessary to carry out additional measurements or structure refinements. However, the nature of your study may justify the reported deviations from journal submission requirements and the more serious of these should be commented upon in the discussion or experimental section of a paper or in the "special_details" fields of the CIF. *checkCIF* was carefully designed to identify outliers and unusual parameters, but every test has its limitations and alerts that are not important in a particular case may appear. Conversely, the absence of alerts does not guarantee there are no aspects of the results needing attention. It is up to the individual to critically assess their own results and, if necessary, seek expert advice.

If you wish to submit your CIF for publication in Acta Crystallographica Section C or E, you should upload your CIF via the web. If you wish to submit your CIF for publication in IUCrData you should upload your CIF via the web. If your CIF is to form part of a submission to another IUCr journal, you will be asked, either during electronic submission or by the Co-editor handling your paper, to upload your CIF via our web site.

---

**PLATON version of 19/02/2022; check.def file version of 19/01/2022**

**Datablock ST098** - ellipsoid plot

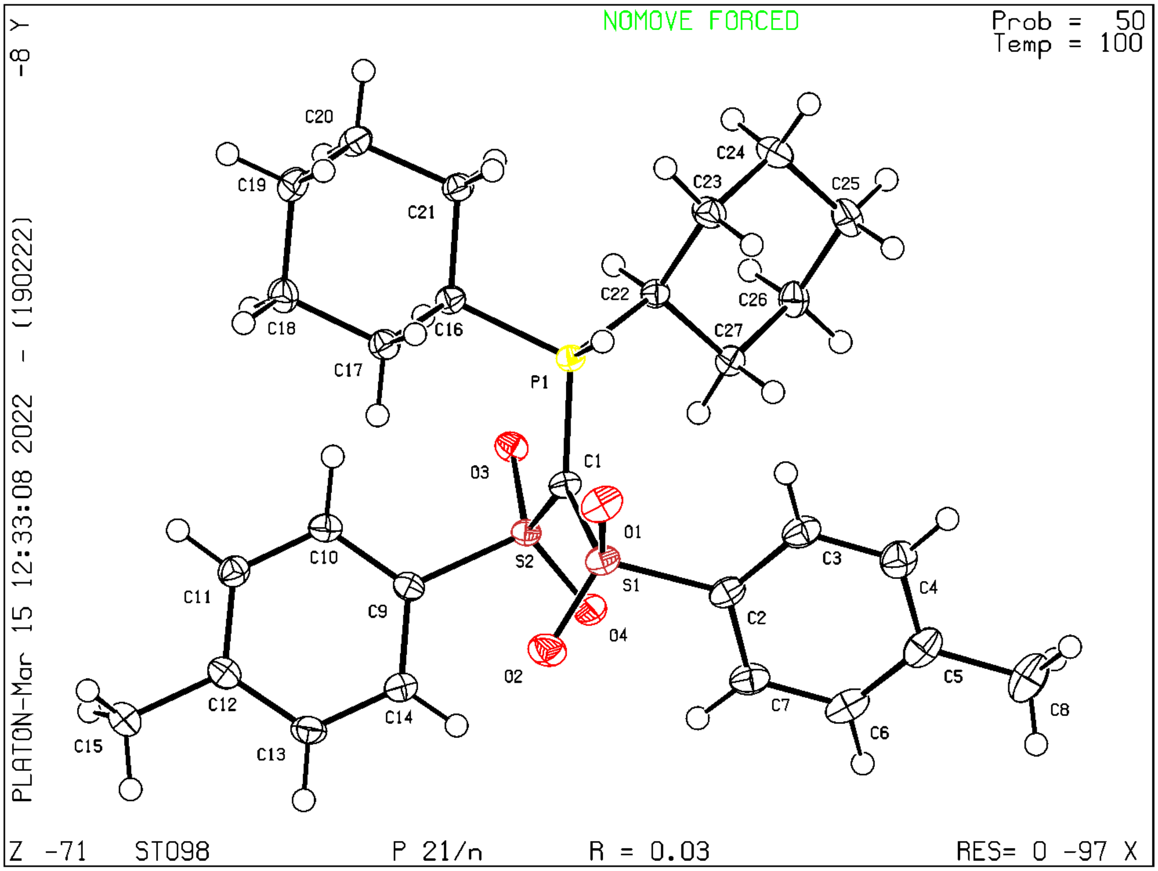

# checkCIF/PLATON report

Structure factors have been supplied for datablock(s) JAZ-474_a

THIS REPORT IS FOR GUIDANCE ONLY. IF USED AS PART OF A REVIEW PROCEDURE FOR PUBLICATION, IT SHOULD NOT REPLACE THE EXPERTISE OF AN EXPERIENCED CRYSTALLOGRAPHIC REFEREE.

No syntax errors found. CIF dictionary Interpreting this report

# Datablock: JAZ-474_a

---

```
Bond precision:   C-C = 0.0040 A          Wavelength=1.54184

Cell:              a=9.8069(2)     b=16.2526(3)       c=13.8974(3)
                   alpha=90        beta=95.198(2)     gamma=90
Temperature:       100 K

                 Calculated                      Reported
Volume           2205.96(8)                      2205.96(8)
Space group      P 21/n                          P 21/n
Hall group       -P 2yn                          -P 2yn
Moiety formula   C21 H29 O4 P S2                 C21 H29 O4 P S2
Sum formula      C21 H29 O4 P S2                 C21 H29 O4 P S2
Mr               440.53                          440.53
Dx,g cm-3        1.327                           1.326
Z                4                               4
Mu (mm-1)        3.072                           3.072
F000             936.0                           936.0
F000'            942.10
h,k,lmax         12,20,17                        12,20,17
Nref             4719                            4680
Tmin,Tmax        0.810,0.890                     0.487,1.000
Tmin'            0.451

Correction method= # Reported T Limits: Tmin=0.487 Tmax=1.000
AbsCorr = GAUSSIAN

Data completeness= 0.992           Theta(max)= 77.856

                                                     wR2(reflections)=
R(reflections)= 0.0555( 4329)                        0.1465( 4680)

S = 1.076                    Npar= 263
```

The following ALERTS were generated. Each ALERT has the format
**test-name_ALERT_alert-type_alert-level.**
Click on the hyperlinks for more details of the test.

### Alert level C

```
PLAT222_ALERT_3_C NonSolvent Resd 1  H   Uiso(max)/Uiso(min) Range        4.1 Ratio
PLAT245_ALERT_2_C U(iso) H1        Smaller than U(eq) P1        by      0.014 Ang**2
PLAT906_ALERT_3_C Large K Value in the Analysis of Variance ......      4.930 Check
```

### Alert level G

```
PLAT883_ALERT_1_G No Info/Value for _atom_sites_solution_primary .     Please Do !
PLAT912_ALERT_4_G Missing # of FCF Reflections Above STh/L=  0.600        39 Note
PLAT965_ALERT_2_G The SHELXL WEIGHT Optimisation has not Converged     Please Check
PLAT978_ALERT_2_G Number C-C Bonds with Positive Residual Density.         7 Info
PLAT992_ALERT_5_G Repd & Actual _reflns_number_gt Values Differ by         3 Check
```

0 **ALERT level A** = Most likely a serious problem - resolve or explain
0 **ALERT level B** = A potentially serious problem, consider carefully
3 **ALERT level C** = Check. Ensure it is not caused by an omission or oversight
5 **ALERT level G** = General information/check it is not something unexpected

1 ALERT type 1 CIF construction/syntax error, inconsistent or missing data
3 ALERT type 2 Indicator that the structure model may be wrong or deficient
2 ALERT type 3 Indicator that the structure quality may be low
1 ALERT type 4 Improvement, methodology, query or suggestion
1 ALERT type 5 Informative message, check

# checkCIF publication errors

### Alert level G

PUBL017_ALERT_1_G The _publ_section_references section is missing or
empty.

0 **ALERT level A** = Data missing that is essential or data in wrong format
1 **ALERT level G** = General alerts. Data that may be required is missing

**Publication of your CIF**

You should attempt to resolve as many as possible of the alerts in all categories. Often the minor alerts point to easily fixed oversights, errors and omissions in your CIF or refinement strategy, so attention to these fine details can be worthwhile. In order to resolve some of the more serious problems it may be necessary to carry out additional measurements or structure refinements. However, the nature of your study may justify the reported deviations from journal submission requirements and the more serious of these should be commented upon in the discussion or experimental section of a paper or in the "special_details" fields of the CIF. *checkCIF* was carefully designed to identify outliers and unusual parameters, but every test has its limitations and alerts that are not important in a particular case may appear. Conversely, the absence of alerts does not guarantee there are no aspects of the results needing attention. It is up to the individual to critically assess their own results and, if necessary, seek expert advice.

If you wish to submit your CIF for publication in Acta Crystallographica Section C or E, you should upload your CIF via the web. If you wish to submit your CIF for publication in IUCrData you should upload your CIF via the web. If your CIF is to form part of a submission to another IUCr journal, you will be asked, either during electronic submission or by the Co-editor handling your paper, to upload your CIF via our web site.

---

**PLATON version of 18/12/2021; check.def file version of 18/12/2021**

**Datablock JAZ-474_a** - ellipsoid plot

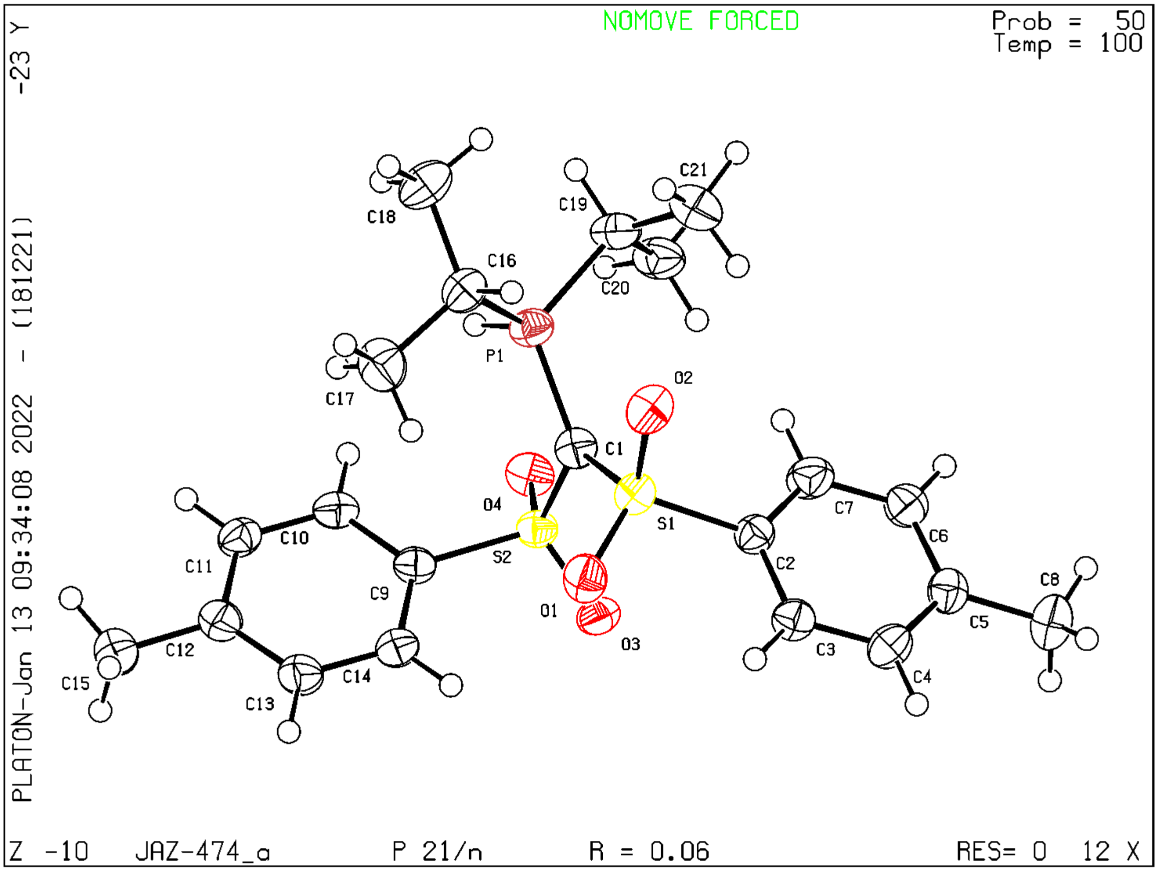

# checkCIF/PLATON report

Structure factors have been supplied for datablock(s) st152

THIS REPORT IS FOR GUIDANCE ONLY. IF USED AS PART OF A REVIEW PROCEDURE FOR PUBLICATION, IT SHOULD NOT REPLACE THE EXPERTISE OF AN EXPERIENCED CRYSTALLOGRAPHIC REFEREE.

No syntax errors found. CIF dictionary Interpreting this report

# Datablock: st152

---

```
Bond precision:   C-C = 0.0031 A          Wavelength=0.71073

Cell:           a=11.3732(3)        b=11.6904(2)     c=12.9393(3)
                alpha=84.4544(18)   beta=65.771(3)   gamma=70.783(2)
Temperature:    100 K
```

| | Calculated | Reported |
|---|---|---|
| Volume | 1479.94(7) | 1479.93(7) |
| Space group | P -1 | P -1 |
| Hall group | -P 1 | -P 1 |
| Moiety formula | 2(C23 H31 N2 O4 P S2), C4 H8 O, C5 H12 | 2(C23 H31 N2 O4 P S2), C4 H8 O, C5 H12 |
| Sum formula | C55 H82 N4 O9 P2 S4 | C55 H82 N4 O9 P2 S4 |
| Mr | 1133.43 | 1133.42 |
| Dx,g cm-3 | 1.272 | 1.272 |
| Z | 1 | 1 |
| Mu (mm-1) | 0.270 | 0.270 |
| F000 | 606.0 | 606.0 |
| F000' | 606.93 | |
| h,k,lmax | 14,14,16 | 14,14,16 |
| Nref | 6051 | 6044 |
| Tmin,Tmax | 0.970,0.980 | 0.677,1.000 |
| Tmin' | 0.953 | |

```
Correction method= # Reported T Limits: Tmin=0.677 Tmax=1.000
AbsCorr = GAUSSIAN

Data completeness= 0.999            Theta(max)= 26.372

R(reflections)= 0.0437( 5381)         wR2(reflections)= 0.1220( 6044)

S = 1.043                  Npar= 376
```

The following ALERTS were generated. Each ALERT has the format
**test-name_ALERT_alert-type_alert-level.**
Click on the hyperlinks for more details of the test.

### Alert level C

```
PLAT094_ALERT_2_C Ratio of Maximum / Minimum Residual Density ....       2.41 Report
PLAT260_ALERT_2_C Large Average Ueq of Residue Including      C1_2      0.120 Check
PLAT906_ALERT_3_C Large K Value in the Analysis of Variance ......      2.152 Check
PLAT910_ALERT_3_C Missing # of FCF Reflection(s) Below Theta(Min).          8 Note
```

### Alert level G

```
PLAT002_ALERT_2_G Number of Distance or Angle Restraints on AtSite         10 Note
PLAT171_ALERT_4_G The CIF-Embedded .res File Contains EADP Records          1 Report
PLAT175_ALERT_4_G The CIF-Embedded .res File Contains SAME Records          2 Report
PLAT187_ALERT_4_G The CIF-Embedded .res File Contains RIGU Records          2 Report
PLAT300_ALERT_4_G Atom Site Occupancy of O1_1       Constrained at        0.5 Check
PLAT300_ALERT_4_G Atom Site Occupancy of C1_1       Constrained at        0.5 Check
PLAT300_ALERT_4_G Atom Site Occupancy of C2_1       Constrained at        0.5 Check
PLAT300_ALERT_4_G Atom Site Occupancy of C3_1       Constrained at        0.5 Check
PLAT300_ALERT_4_G Atom Site Occupancy of C4_1       Constrained at        0.5 Check
PLAT300_ALERT_4_G Atom Site Occupancy of H1A_1      Constrained at        0.5 Check
PLAT300_ALERT_4_G Atom Site Occupancy of H1B_1      Constrained at        0.5 Check
PLAT300_ALERT_4_G Atom Site Occupancy of H2A_1      Constrained at        0.5 Check
PLAT300_ALERT_4_G Atom Site Occupancy of H2B_1      Constrained at        0.5 Check
PLAT300_ALERT_4_G Atom Site Occupancy of H3A_1      Constrained at        0.5 Check
PLAT300_ALERT_4_G Atom Site Occupancy of H3B_1      Constrained at        0.5 Check
PLAT300_ALERT_4_G Atom Site Occupancy of H4A_1      Constrained at        0.5 Check
PLAT300_ALERT_4_G Atom Site Occupancy of H4B_1      Constrained at        0.5 Check
PLAT300_ALERT_4_G Atom Site Occupancy of C1_2       Constrained at        0.5 Check
PLAT300_ALERT_4_G Atom Site Occupancy of C2_2       Constrained at        0.5 Check
PLAT300_ALERT_4_G Atom Site Occupancy of C3_2       Constrained at        0.5 Check
PLAT300_ALERT_4_G Atom Site Occupancy of C4_2       Constrained at        0.5 Check
PLAT300_ALERT_4_G Atom Site Occupancy of C5_2       Constrained at        0.5 Check
PLAT300_ALERT_4_G Atom Site Occupancy of H1A_2      Constrained at        0.5 Check
PLAT300_ALERT_4_G Atom Site Occupancy of H1B_2      Constrained at        0.5 Check
PLAT300_ALERT_4_G Atom Site Occupancy of H1C_2      Constrained at        0.5 Check
PLAT300_ALERT_4_G Atom Site Occupancy of H2A_2      Constrained at        0.5 Check
PLAT300_ALERT_4_G Atom Site Occupancy of H2B_2      Constrained at        0.5 Check
PLAT300_ALERT_4_G Atom Site Occupancy of H3A_2      Constrained at        0.5 Check
PLAT300_ALERT_4_G Atom Site Occupancy of H3B_2      Constrained at        0.5 Check
PLAT300_ALERT_4_G Atom Site Occupancy of H4A_2      Constrained at        0.5 Check
PLAT300_ALERT_4_G Atom Site Occupancy of H4B_2      Constrained at        0.5 Check
PLAT300_ALERT_4_G Atom Site Occupancy of H5A_2      Constrained at        0.5 Check
PLAT300_ALERT_4_G Atom Site Occupancy of H5B_2      Constrained at        0.5 Check
PLAT300_ALERT_4_G Atom Site Occupancy of H5C_2      Constrained at        0.5 Check
PLAT302_ALERT_4_G Anion/Solvent/Minor-Residue Disorder (Resd  2  )       100% Note
PLAT302_ALERT_4_G Anion/Solvent/Minor-Residue Disorder (Resd  3  )       100% Note
PLAT304_ALERT_4_G Non-Integer Number of Atoms in ..... (Resd  2  )       6.50 Check
PLAT304_ALERT_4_G Non-Integer Number of Atoms in ..... (Resd  3  )       8.50 Check
PLAT398_ALERT_2_G Deviating  C-O-C   Angle From 120 for O1_1      .     108.0 Degree
PLAT720_ALERT_4_G Number of Unusual/Non-Standard Labels ..........         30 Note
PLAT789_ALERT_4_G Atoms with Negative _atom_site_disorder_group  #         30 Check
PLAT860_ALERT_3_G Number of Least-Squares Restraints .............         58 Note
PLAT883_ALERT_1_G No Info/Value for _atom_sites_solution_primary .     Please Do !
```

```
PLAT978_ALERT_2_G Number C-C Bonds with Positive Residual Density.          4 Info
PLAT992_ALERT_5_G Repd & Actual _reflns_number_gt Values Differ by          2 Check
```

---

```
   0 ALERT level A = Most likely a serious problem - resolve or explain
   0 ALERT level B = A potentially serious problem, consider carefully
   4 ALERT level C = Check. Ensure it is not caused by an omission or oversight
  45 ALERT level G = General information/check it is not something unexpected

   1 ALERT type 1 CIF construction/syntax error, inconsistent or missing data
   5 ALERT type 2 Indicator that the structure model may be wrong or deficient
   3 ALERT type 3 Indicator that the structure quality may be low
  39 ALERT type 4 Improvement, methodology, query or suggestion
   1 ALERT type 5 Informative message, check
```

---

# checkCIF publication errors

---

**Alert level G**

```
PUBL017_ALERT_1_G The _publ_section_references section is missing or
            empty.
```

---

```
   0 ALERT level A = Data missing that is essential or data in wrong format
   1 ALERT level G = General alerts. Data that may be required is missing
```

---

**Publication of your CIF**

You should attempt to resolve as many as possible of the alerts in all categories. Often the minor alerts point to easily fixed oversights, errors and omissions in your CIF or refinement strategy, so attention to these fine details can be worthwhile. In order to resolve some of the more serious problems it may be necessary to carry out additional measurements or structure refinements. However, the nature of your study may justify the reported deviations from journal submission requirements and the more serious of these should be commented upon in the discussion or experimental section of a paper or in the "special_details" fields of the CIF. *checkCIF* was carefully designed to identify outliers and unusual parameters, but every test has its limitations and alerts that are not important in a particular case may appear. Conversely, the absence of alerts does not guarantee there are no aspects of the results needing attention. It is up to the individual to critically assess their own results and, if necessary, seek expert advice.

If you wish to submit your CIF for publication in Acta Crystallographica Section C or E, you should upload your CIF via the web. If you wish to submit your CIF for publication in IUCrData you should upload your CIF via the web. If your CIF is to form part of a submission to another IUCr journal, you will be asked, either during electronic submission or by the Co-editor handling your paper, to upload your CIF via our web site.

## PLATON version of 20/01/2022; check.def file version of 19/01/2022

**Datablock st152** - ellipsoid plot

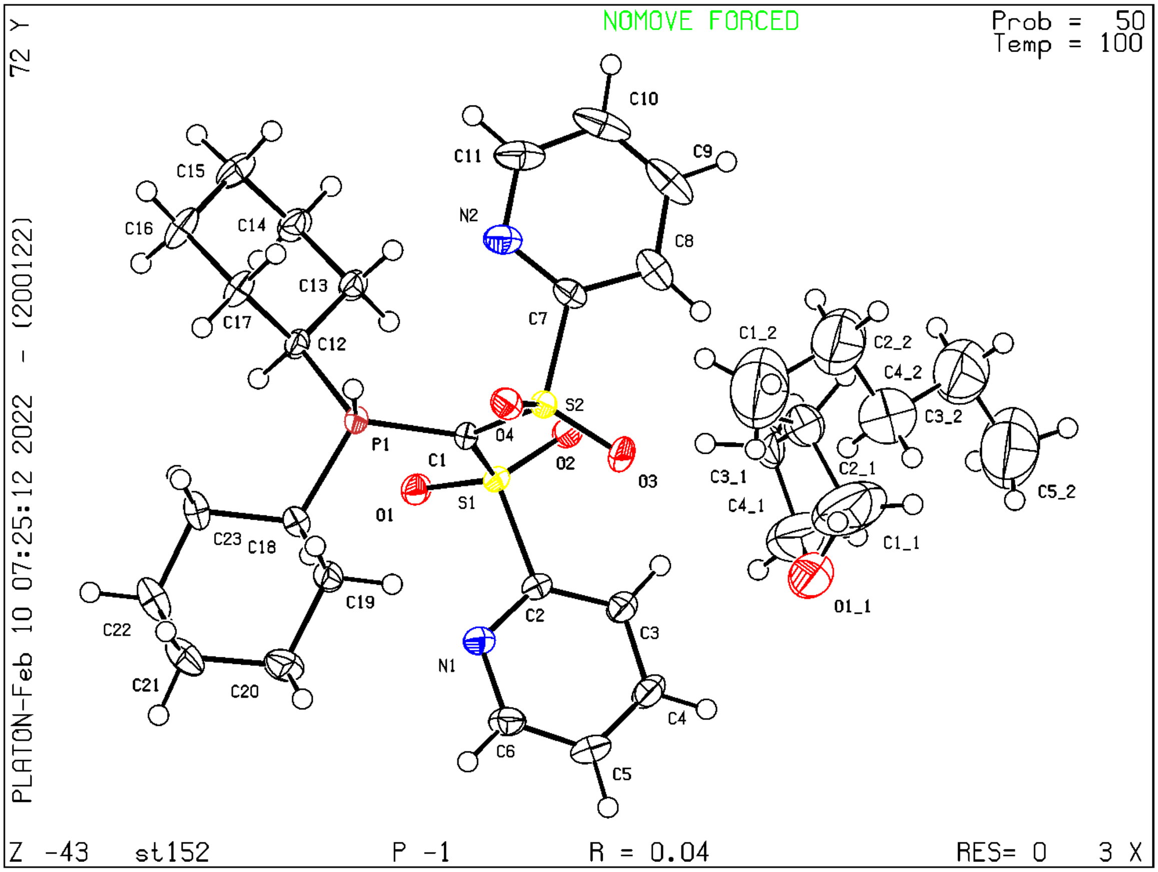

# checkCIF/PLATON report

Structure factors have been supplied for datablock(s) shelxt_a

THIS REPORT IS FOR GUIDANCE ONLY. IF USED AS PART OF A REVIEW PROCEDURE FOR PUBLICATION, IT SHOULD NOT REPLACE THE EXPERTISE OF AN EXPERIENCED CRYSTALLOGRAPHIC REFEREE.

No syntax errors found. CIF dictionary Interpreting this report

## Datablock: shelxt_a

---

| | | | |
|---|---|---|---|
| Bond precision: | C-C = 0.0071 A | | Wavelength=0.71073 |
| Cell: | a=12.6701(3) | b=14.4364(2) | c=19.2951(4) |
| | alpha=90 | beta=104.570(2) | gamma=90 |
| Temperature: | 100 K | | |

| | Calculated | Reported |
|---|---|---|
| Volume | 3415.78(12) | 3415.78(12) |
| Space group | P 21/c | P 21/c |
| Hall group | -P 2ybc | -P 2ybc |
| Moiety formula | C70 H104 Na2 O12 P2 S4 | C70 H104 Na2 O12 P2 S4 |
| Sum formula | C70 H104 Na2 O12 P2 S4 | C70 H104 Na2 O12 P2 S4 |
| Mr | 1373.69 | 1373.69 |
| Dx,g cm-3 | 1.336 | 1.336 |
| Z | 2 | 2 |
| Mu (mm-1) | 0.260 | 0.260 |
| F000 | 1472.0 | 1472.0 |
| F000' | 1474.10 | |
| h,k,lmax | 15,17,22 | 15,17,22 |
| Nref | 6010 | 12944 |
| Tmin,Tmax | 0.963,0.991 | 0.365,1.000 |
| Tmin' | 0.948 | |

Correction method= # Reported T Limits: Tmin=0.365 Tmax=1.000
AbsCorr = GAUSSIAN

Data completeness= 2.154 Theta(max)= 24.997

R(reflections)= 0.0770( 10288) wR2(reflections)= 0.1891( 12944)

S = 1.069 Npar= 409

The following ALERTS were generated. Each ALERT has the format
**test-name_ALERT_alert-type_alert-level.**
Click on the hyperlinks for more details of the test.

**Alert level C**

```
PLAT220_ALERT_2_C NonSolvent   Resd 1  C   Ueq(max)/Ueq(min) Range        3.6 Ratio
PLAT340_ALERT_3_C Low Bond Precision on  C-C Bonds ...............    0.00709 Ang.
PLAT906_ALERT_3_C Large K Value in the Analysis of Variance ......      9.406 Check
PLAT906_ALERT_3_C Large K Value in the Analysis of Variance ......      2.252 Check
PLAT910_ALERT_3_C Missing # of FCF Reflection(s) Below Theta(Min).          8 Note
```

**Alert level G**

```
PLAT083_ALERT_2_G SHELXL Second Parameter in WGHT  Unusually Large       6.10 Why ?
PLAT720_ALERT_4_G Number of Unusual/Non-Standard Labels ..........         42 Note
PLAT793_ALERT_4_G Model has Chirality at S1          (Centro SPGR)          S Verify
PLAT793_ALERT_4_G Model has Chirality at S2          (Centro SPGR)          S Verify
PLAT870_ALERT_4_G ALERTS Related to Twinning Effects Suppressed ..          ! Info
PLAT883_ALERT_1_G No Info/Value for _atom_sites_solution_primary .     Please Do !
PLAT909_ALERT_3_G Percentage of I>2sig(I) Data at Theta(Max) Still        64% Note
PLAT931_ALERT_5_G CIFcalcFCF  Twin Law ( 1 0 0)          Est.d BASF       0.21 Check
PLAT941_ALERT_3_G Average HKL Measurement Multiplicity ...........        2.2 Low
PLAT967_ALERT_5_G Note: Two-Theta Cutoff Value in Embedded .res ..       50.0 Degree
PLAT992_ALERT_5_G Repd & Actual _reflns_number_gt Values Differ by          2 Check
```

0 **ALERT level A** = Most likely a serious problem - resolve or explain
0 **ALERT level B** = A potentially serious problem, consider carefully
5 **ALERT level C** = Check. Ensure it is not caused by an omission or oversight
11 **ALERT level G** = General information/check it is not something unexpected

1 ALERT type 1 CIF construction/syntax error, inconsistent or missing data
2 ALERT type 2 Indicator that the structure model may be wrong or deficient
6 ALERT type 3 Indicator that the structure quality may be low
4 ALERT type 4 Improvement, methodology, query or suggestion
3 ALERT type 5 Informative message, check

# checkCIF publication errors

**Alert level G**

PUBL017_ALERT_1_G The _publ_section_references section is missing or
empty.

0 **ALERT level A** = Data missing that is essential or data in wrong format
1 **ALERT level G** = General alerts. Data that may be required is missing

**Publication of your CIF**

You should attempt to resolve as many as possible of the alerts in all categories. Often the minor alerts point to easily fixed oversights, errors and omissions in your CIF or refinement strategy, so attention to these fine details can be worthwhile. In order to resolve some of the more serious problems it may be necessary to carry out additional measurements or structure refinements. However, the nature of your study may justify the reported deviations from journal submission requirements and the more serious of these should be commented upon in the discussion or experimental section of a paper or in the "special_details" fields of the CIF. *checkCIF* was carefully designed to identify outliers and unusual parameters, but every test has its limitations and alerts that are not important in a particular case may appear. Conversely, the absence of alerts does not guarantee there are no aspects of the results needing attention. It is up to the individual to critically assess their own results and, if necessary, seek expert advice.

If you wish to submit your CIF for publication in Acta Crystallographica Section C or E, you should upload your CIF via the web. If you wish to submit your CIF for publication in IUCrData you should upload your CIF via the web. If your CIF is to form part of a submission to another IUCr journal, you will be asked, either during electronic submission or by the Co-editor handling your paper, to upload your CIF via our web site.

---

**PLATON version of 18/12/2021; check.def file version of 18/12/2021**

**Datablock shelxt_a** - ellipsoid plot

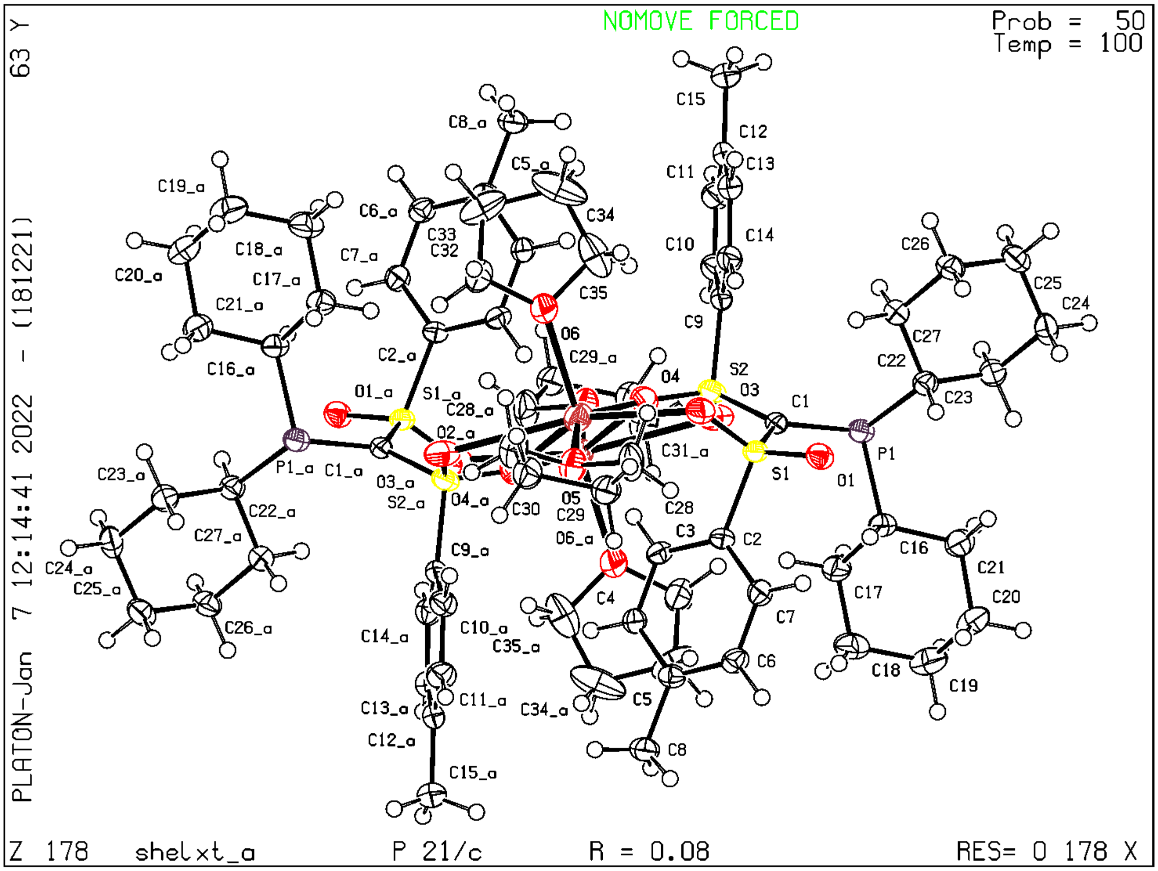

# checkCIF/PLATON report

Structure factors have been supplied for datablock(s) JAZ018_a

THIS REPORT IS FOR GUIDANCE ONLY. IF USED AS PART OF A REVIEW PROCEDURE FOR PUBLICATION, IT SHOULD NOT REPLACE THE EXPERTISE OF AN EXPERIENCED CRYSTALLOGRAPHIC REFEREE.

No syntax errors found. CIF dictionary Interpreting this report

## Datablock: JAZ018_a

```
Bond precision:   C-C = 0.0023 A          Wavelength=1.54184

Cell:           a=12.4991(1)       b=15.0439(1)      c=15.8625(1)
                alpha=88.871(1)    beta=88.286(1)    gamma=85.862(1)
Temperature:    100 K
```

| | Calculated | Reported |
|---|---|---|
| Volume | 2973.14(4) | 2973.14(4) |
| Space group | P -1 | P -1 |
| Hall group | -P 1 | -P 1 |
| Moiety formula | C62 H64 Na2 O10 P2 S4 | C62 H64 Na2 O10 P2 S4 |
| Sum formula | C62 H64 Na2 O10 P2 S4 | C62 H64 Na2 O10 P2 S4 |
| Mr | 1205.30 | 1205.29 |
| Dx,g cm-3 | 1.346 | 1.346 |
| Z | 2 | 2 |
| Mu (mm-1) | 2.595 | 2.595 |
| F000 | 1264.0 | 1264.0 |
| F000' | 1271.49 | |
| h,k,lmax | 15,19,20 | 15,19,20 |
| Nref | 12640 | 12225 |
| Tmin,Tmax | 0.705,0.802 | 0.495,1.000 |
| Tmin' | 0.626 | |

```
Correction method= # Reported T Limits: Tmin=0.495 Tmax=1.000
AbsCorr = GAUSSIAN

Data completeness= 0.967          Theta(max)= 77.574

R(reflections)= 0.0351( 11525)         wR2(reflections)= 0.0947( 12225)

S = 1.062                    Npar= 725
```

The following ALERTS were generated. Each ALERT has the format
**test-name_ALERT_alert-type_alert-level.**
Click on the hyperlinks for more details of the test.

### Alert level C

```
PLAT220_ALERT_2_C NonSolvent   Resd 1  C   Ueq(max)/Ueq(min) Range        4.7 Ratio
PLAT241_ALERT_2_C High   'MainMol' Ueq as Compared to Neighbors of       C3_1 Check
PLAT241_ALERT_2_C High   'MainMol' Ueq as Compared to Neighbors of      C3'_1 Check
PLAT911_ALERT_3_C Missing FCF Refl Between Thmin & STh/L=    0.600          8 Report
```

### Alert level G

```
PLAT004_ALERT_5_G Polymeric Structure Found with Maximum Dimension          1 Info
PLAT154_ALERT_1_G The s.u.'s on the Cell Angles are Equal ..(Note)      0.001 Degree
PLAT720_ALERT_4_G Number of Unusual/Non-Standard Labels ..........         29 Note
PLAT883_ALERT_1_G No Info/Value for _atom_sites_solution_primary .     Please Do !
PLAT912_ALERT_4_G Missing # of FCF Reflections Above STh/L=  0.600        407 Note
PLAT941_ALERT_3_G Average HKL Measurement Multiplicity ...........        3.3 Low
PLAT978_ALERT_2_G Number C-C Bonds with Positive Residual Density.          5 Info
PLAT992_ALERT_5_G Repd & Actual _reflns_number_gt Values Differ by          2 Check
```

0 **ALERT level A** = Most likely a serious problem - resolve or explain
0 **ALERT level B** = A potentially serious problem, consider carefully
4 **ALERT level C** = Check. Ensure it is not caused by an omission or oversight
8 **ALERT level G** = General information/check it is not something unexpected

2 ALERT type 1 CIF construction/syntax error, inconsistent or missing data
4 ALERT type 2 Indicator that the structure model may be wrong or deficient
2 ALERT type 3 Indicator that the structure quality may be low
2 ALERT type 4 Improvement, methodology, query or suggestion
2 ALERT type 5 Informative message, check

# checkCIF publication errors

### Alert level G

PUBL017_ALERT_1_G The _publ_section_references section is missing or empty.

0 **ALERT level A** = Data missing that is essential or data in wrong format
1 **ALERT level G** = General alerts. Data that may be required is missing

**Publication of your CIF**

You should attempt to resolve as many as possible of the alerts in all categories. Often the minor alerts point to easily fixed oversights, errors and omissions in your CIF or refinement strategy, so attention to these fine details can be worthwhile. In order to resolve some of the more serious problems it may be necessary to carry out additional measurements or structure refinements. However, the nature of your study may justify the reported deviations from journal submission requirements and the more serious of these should be commented upon in the discussion or experimental section of a paper or in the "special_details" fields of the CIF. *checkCIF* was carefully designed to identify outliers and unusual parameters, but every test has its limitations and alerts that are not important in a particular case may appear. Conversely, the absence of alerts does not guarantee there are no aspects of the results needing attention. It is up to the individual to critically assess their own results and, if necessary, seek expert advice.

If you wish to submit your CIF for publication in Acta Crystallographica Section C or E, you should upload your CIF via the web. If you wish to submit your CIF for publication in IUCrData you should upload your CIF via the web. If your CIF is to form part of a submission to another IUCr journal, you will be asked, either during electronic submission or by the Co-editor handling your paper, to upload your CIF via our web site.

---

**PLATON version of 13/07/2021; check.def file version of 13/07/2021**

**Datablock JAZ018_a** - ellipsoid plot

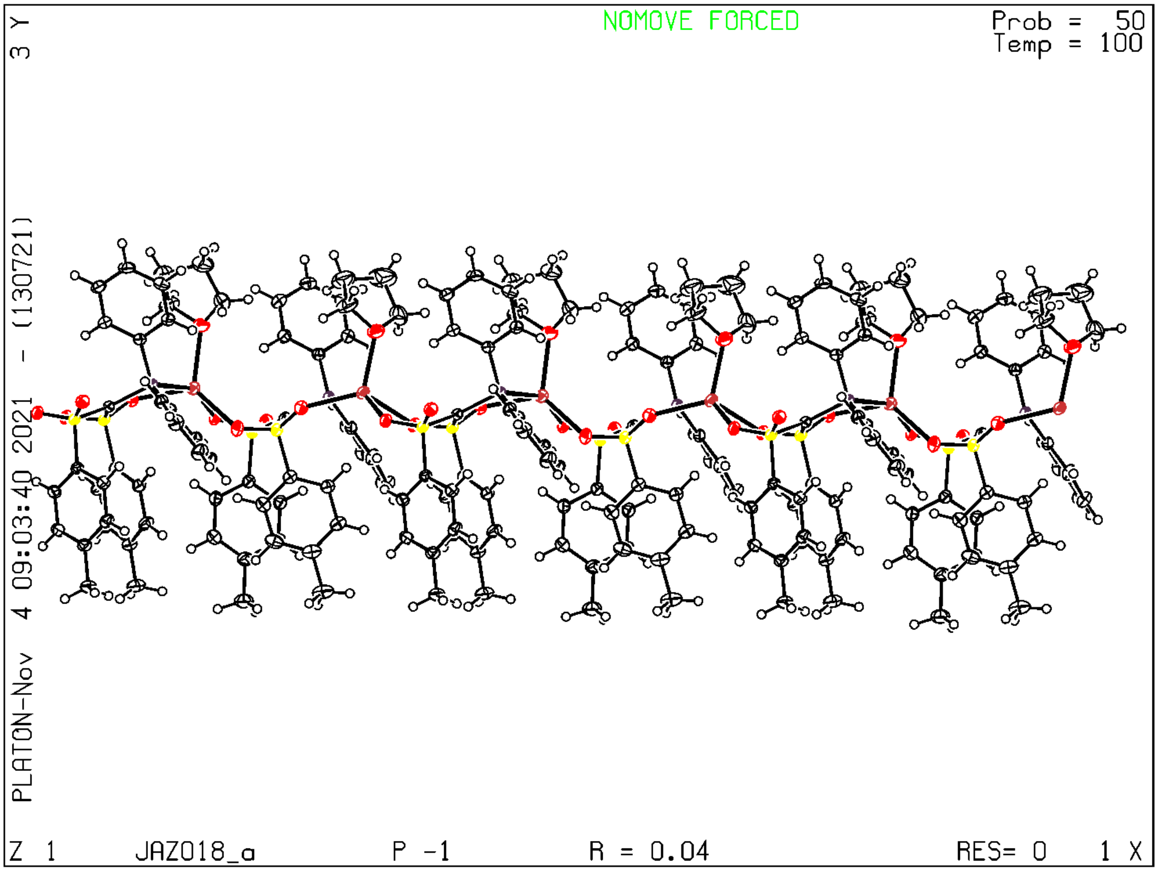

# checkCIF/PLATON report

Structure factors have been supplied for datablock(s) JAZ-481_a

THIS REPORT IS FOR GUIDANCE ONLY. IF USED AS PART OF A REVIEW PROCEDURE FOR PUBLICATION, IT SHOULD NOT REPLACE THE EXPERTISE OF AN EXPERIENCED CRYSTALLOGRAPHIC REFEREE.

No syntax errors found. CIF dictionary Interpreting this report

## Datablock: JAZ-481_a

---

```
Bond precision:  C-C = 0.0030 A          Wavelength=1.54184

Cell:           a=12.3390(1)    b=15.0623(2)       c=14.9335(2)
                alpha=90        beta=102.592(1)    gamma=90
Temperature:    100 K

                Calculated                Reported
Volume          2708.69(6)                2708.69(6)
Space group     P 21/n                    P 21/n
Hall group      -P 2yn                    -P 2yn
Moiety formula  C28 H39 O4 P S2           C28 H39 O4 P S2
Sum formula     C28 H39 O4 P S2           C28 H39 O4 P S2
Mr              534.68                    534.68
Dx,g cm-3       1.311                     1.311
Z               4                         4
Mu (mm-1)       2.596                     2.596
F000            1144.0                    1144.0
F000'           1150.58
h,k,lmax        15,19,18                  15,19,18
Nref            5771                      5720
Tmin,Tmax       0.788,0.871               0.702,1.000
Tmin'           0.788

Correction method= # Reported T Limits: Tmin=0.702 Tmax=1.000
AbsCorr = GAUSSIAN

Data completeness= 0.991          Theta(max)= 77.771

R(reflections)= 0.0400( 5312)     wR2(reflections)= 0.1014( 5720)

S = 1.091                  Npar= 319
```

---

The following ALERTS were generated. Each ALERT has the format
**test-name_ALERT_alert-type_alert-level**.
Click on the hyperlinks for more details of the test.

### Alert level C

```
PLAT906_ALERT_3_C Large K Value in the Analysis of Variance ......      3.176 Check
```

### Alert level G

```
PLAT883_ALERT_1_G No Info/Value for _atom_sites_solution_primary .     Please Do !
PLAT912_ALERT_4_G Missing # of FCF Reflections Above STh/L=  0.600         51 Note
PLAT978_ALERT_2_G Number C-C Bonds with Positive Residual Density.          9 Info
```

```
   0 ALERT level A = Most likely a serious problem - resolve or explain
   0 ALERT level B = A potentially serious problem, consider carefully
   1 ALERT level C = Check. Ensure it is not caused by an omission or oversight
   3 ALERT level G = General information/check it is not something unexpected

   1 ALERT type 1 CIF construction/syntax error, inconsistent or missing data
   1 ALERT type 2 Indicator that the structure model may be wrong or deficient
   1 ALERT type 3 Indicator that the structure quality may be low
   1 ALERT type 4 Improvement, methodology, query or suggestion
   0 ALERT type 5 Informative message, check
```

# checkCIF publication errors

### Alert level G

```
PUBL017_ALERT_1_G The _publ_section_references section is missing or
            empty.
```

```
   0 ALERT level A = Data missing that is essential or data in wrong format
   1 ALERT level G = General alerts. Data that may be required is missing
```

**Publication of your CIF**

You should attempt to resolve as many as possible of the alerts in all categories. Often the minor alerts point to easily fixed oversights, errors and omissions in your CIF or refinement strategy, so attention to these fine details can be worthwhile. In order to resolve some of the more serious problems it may be necessary to carry out additional measurements or structure refinements. However, the nature of your study may justify the reported deviations from journal submission requirements and the more serious of these should be commented upon in the discussion or experimental section of a paper or in the "special_details" fields of the CIF. *checkCIF* was carefully designed to identify outliers and unusual parameters, but every test has its limitations and alerts that are not important in a particular case may appear. Conversely, the absence of alerts does not guarantee there are no aspects of the results needing attention. It is up to the individual to critically assess their own results and, if necessary, seek expert advice.

If you wish to submit your CIF for publication in Acta Crystallographica Section C or E, you should upload your CIF via the web. If you wish to submit your CIF for publication in IUCrData you should upload your CIF via the web. If your CIF is to form part of a submission to another IUCr journal, you will be asked, either during electronic submission or by the Co-editor handling your paper, to upload your CIF via our web site.

## PLATON version of 05/12/2020; check.def file version of 05/12/2020

**Datablock JAZ-481_a** - ellipsoid plot

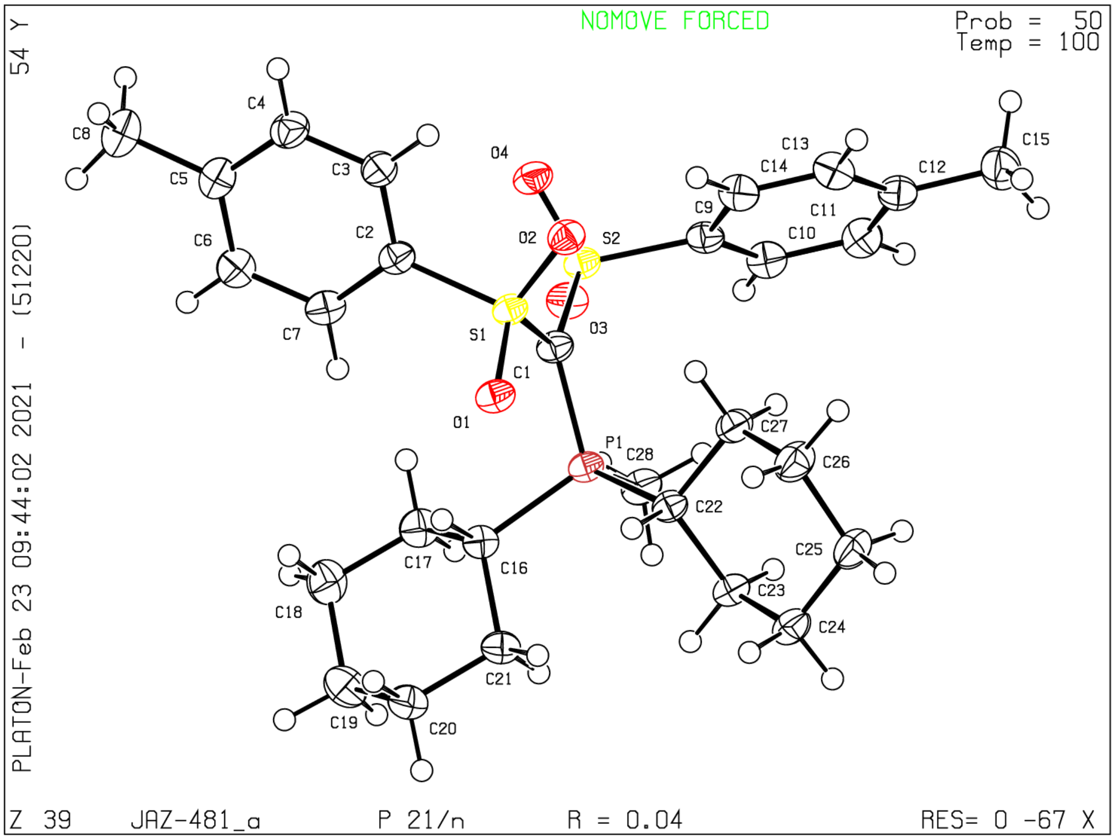

# checkCIF/PLATON report

Structure factors have been supplied for datablock(s) JAZ_030_a

THIS REPORT IS FOR GUIDANCE ONLY. IF USED AS PART OF A REVIEW PROCEDURE FOR PUBLICATION, IT SHOULD NOT REPLACE THE EXPERTISE OF AN EXPERIENCED CRYSTALLOGRAPHIC REFEREE.

No syntax errors found. CIF dictionary Interpreting this report

## Datablock: JAZ_030_a

---

| | | | |
|---|---|---|---|
| Bond precision: | C-C = 0.0032 A | | Wavelength=1.54184 |
| Cell: | a=14.9214(1) | b=10.4987(1) | c=21.2849(2) |
| | alpha=90 | beta=98.406(1) | gamma=90 |
| Temperature: | 100 K | | |

| | Calculated | Reported |
|---|---|---|
| Volume | 3298.57(5) | 3298.57(5) |
| Space group | P 21/c | P 21/c |
| Hall group | -P 2ybc | -P 2ybc |
| Moiety formula | C30 H37 N2 O4 P S2, C4 H8 O | C30 H37 N2 O4 P S2, C4 H8 O |
| Sum formula | C34 H45 N2 O5 P S2 | C34 H45 N2 O5 P S2 |
| Mr | 656.81 | 656.81 |
| Dx,g cm-3 | 1.323 | 1.323 |
| Z | 4 | 4 |
| Mu (mm-1) | 2.276 | 2.276 |
| F000 | 1400.0 | 1400.0 |
| F000' | 1407.39 | |
| h,k,lmax | 18,13,26 | 18,13,26 |
| Nref | 6795 | 6649 |
| Tmin,Tmax | 0.833,0.907 | 0.553,0.829 |
| Tmin' | 0.766 | |

Correction method= # Reported T Limits: Tmin=0.553 Tmax=0.829
AbsCorr = GAUSSIAN

Data completeness= 0.979 Theta(max)= 74.996

R(reflections)= 0.0403( 5938) wR2(reflections)= 0.1083( 6649)

S = 1.033 Npar= 398

The following ALERTS were generated. Each ALERT has the format
**test-name_ALERT_alert-type_alert-level.**
Click on the hyperlinks for more details of the test.

### Alert level C

```
PLAT906_ALERT_3_C Large K Value in the Analysis of Variance ......      2.626 Check
PLAT911_ALERT_3_C Missing FCF Refl Between Thmin & STh/L=    0.600          4 Report
```

### Alert level G

```
PLAT002_ALERT_2_G Number of Distance or Angle Restraints on AtSite          4 Note
PLAT142_ALERT_4_G s.u. on b - Axis Small or Missing .............     0.00010 Ang.
PLAT143_ALERT_4_G s.u. on c - Axis Small or Missing .............     0.00020 Ang.
PLAT172_ALERT_4_G The CIF-Embedded .res File Contains DFIX Records          1 Report
PLAT398_ALERT_2_G Deviating  C-O-C   Angle From 120 for O1_1     .      108.5 Degree
PLAT720_ALERT_4_G Number of Unusual/Non-Standard Labels ..........         13 Note
PLAT860_ALERT_3_G Number of Least-Squares Restraints .............          3 Note
PLAT883_ALERT_1_G No Info/Value for _atom_sites_solution_primary .    Please Do !
PLAT912_ALERT_4_G Missing # of FCF Reflections Above STh/L=  0.600        143 Note
PLAT941_ALERT_3_G Average HKL Measurement Multiplicity ...........        3.8 Low
PLAT967_ALERT_5_G Note: Two-Theta Cutoff Value in Embedded .res ..      150.0 Degree
PLAT978_ALERT_2_G Number C-C Bonds with Positive Residual Density.          7 Info
```

0 **ALERT level A** = Most likely a serious problem - resolve or explain
0 **ALERT level B** = A potentially serious problem, consider carefully
2 **ALERT level C** = Check. Ensure it is not caused by an omission or oversight
12 **ALERT level G** = General information/check it is not something unexpected

1 ALERT type 1 CIF construction/syntax error, inconsistent or missing data
3 ALERT type 2 Indicator that the structure model may be wrong or deficient
4 ALERT type 3 Indicator that the structure quality may be low
5 ALERT type 4 Improvement, methodology, query or suggestion
1 ALERT type 5 Informative message, check

# checkCIF publication errors

### Alert level G

PUBL017_ALERT_1_G The _publ_section_references section is missing or empty.

0 **ALERT level A** = Data missing that is essential or data in wrong format
1 **ALERT level G** = General alerts. Data that may be required is missing

**Publication of your CIF**

You should attempt to resolve as many as possible of the alerts in all categories. Often the minor alerts point to easily fixed oversights, errors and omissions in your CIF or refinement strategy, so attention to these fine details can be worthwhile. In order to resolve some of the more serious problems it may be necessary to carry out additional measurements or structure refinements. However, the nature of your study may justify the reported deviations from journal submission requirements and the more serious of these should be commented upon in the discussion or experimental section of a paper or in the "special_details" fields of the CIF. *checkCIF* was carefully designed to identify outliers and unusual parameters, but every test has its limitations and alerts that are not important in a particular case may appear. Conversely, the absence of alerts does not guarantee there are no aspects of the results needing attention. It is up to the individual to critically assess their own results and, if necessary, seek expert advice.

If you wish to submit your CIF for publication in Acta Crystallographica Section C or E, you should upload your CIF via the web. If you wish to submit your CIF for publication in IUCrData you should upload your CIF via the web. If your CIF is to form part of a submission to another IUCr journal, you will be asked, either during electronic submission or by the Co-editor handling your paper, to upload your CIF via our web site.

---

**PLATON version of 20/01/2022; check.def file version of 19/01/2022**

**Datablock JAZ_030_a** - ellipsoid plot

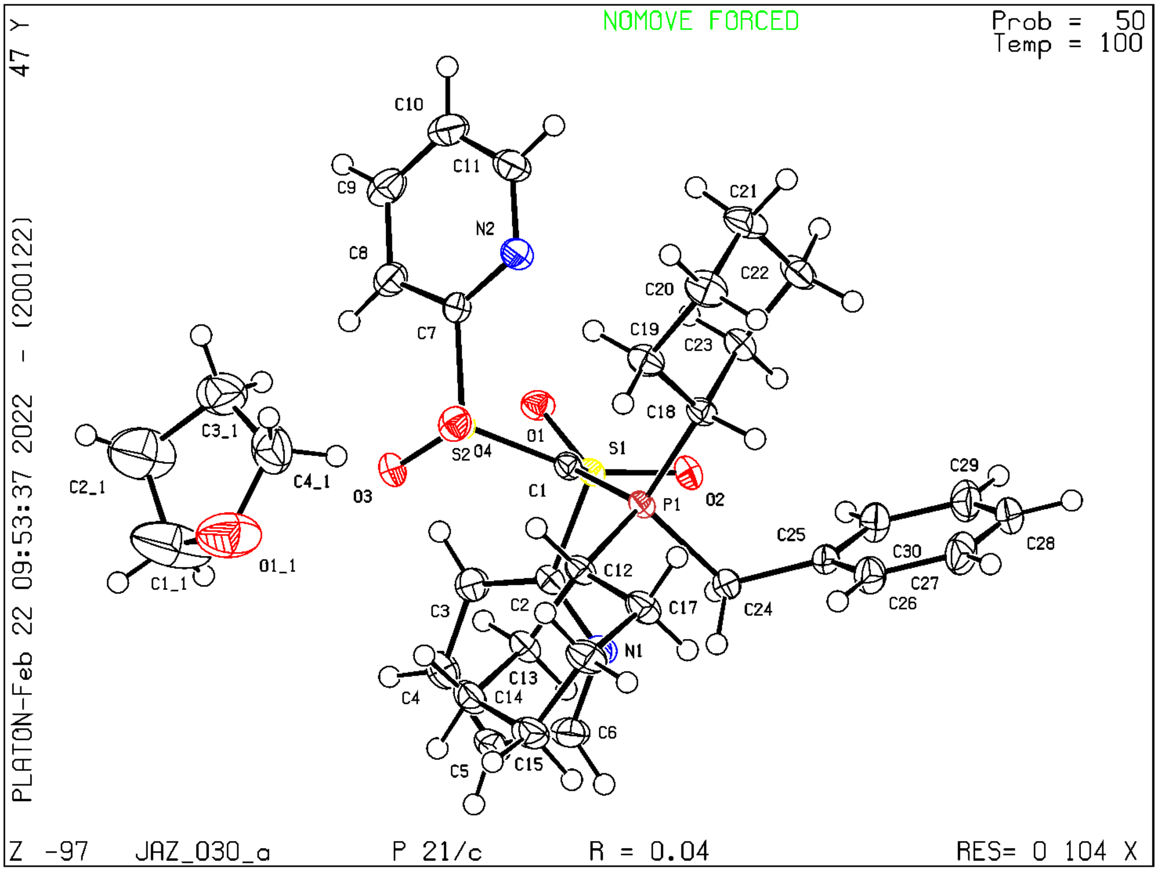

# checkCIF/PLATON report

Structure factors have been supplied for datablock(s) JAZ-520

THIS REPORT IS FOR GUIDANCE ONLY. IF USED AS PART OF A REVIEW PROCEDURE FOR PUBLICATION, IT SHOULD NOT REPLACE THE EXPERTISE OF AN EXPERIENCED CRYSTALLOGRAPHIC REFEREE.

No syntax errors found. CIF dictionary Interpreting this report

# Datablock: JAZ-520

---

Bond precision: C-C = 0.0025 A Wavelength=1.54184

| | | | |
|---|---|---|---|
| Cell: | a=13.37392(6) | b=20.13807(8) | c=27.48704(13) |
| | alpha=90 | beta=98.9050(4) | gamma=90 |
| Temperature: | 100 K | | |

| | Calculated | Reported |
|---|---|---|
| Volume | 7313.71(6) | 7313.71(5) |
| Space group | P 21/c | P 21/c |
| Hall group | -P 2ybc | -P 2ybc |
| Moiety formula | C66 H102 B2 Na2 O11 P2 S4, C4 H8 O | C66 H102 B2 Na2 O11 P2 S4, C4 H8 O |
| Sum formula | C70 H110 B2 Na2 O12 P2 S4 | C70 H110 B2 Na2 O12 P2 S4 |
| Mr | 1401.37 | 1401.37 |
| Dx,g cm-3 | 1.273 | 1.273 |
| Z | 4 | 4 |
| Mu (mm-1) | 2.189 | 2.189 |
| F000 | 3008.0 | 3008.0 |
| F000’ | 3023.97 | |
| h,k,lmax | 16,25,34 | 16,25,34 |
| Nref | 15488 | 15230 |
| Tmin,Tmax | 0.648,0.717 | 0.390,1.000 |
| Tmin’ | 0.524 | |

Correction method= # Reported T Limits: Tmin=0.390 Tmax=1.000
AbsCorr = GAUSSIAN

Data completeness= 0.983 Theta(max)= 77.092

R(reflections)= 0.0378( 14201) wR2(reflections)= 0.1010( 15230)

S = 1.040 Npar= 863

The following ALERTS were generated. Each ALERT has the format
**test-name_ALERT_alert-type_alert-level.**
Click on the hyperlinks for more details of the test.

### Alert level C

```
PLAT220_ALERT_2_C NonSolvent   Resd 1  C   Ueq(max)/Ueq(min) Range        4.5 Ratio
PLAT230_ALERT_2_C Hirshfeld Test Diff for    O7       --C31      .        5.9 s.u.
PLAT241_ALERT_2_C High   'MainMol' Ueq as Compared to Neighbors of        C30 Check
PLAT243_ALERT_4_C High   'Solvent' Ueq as Compared to Neighbors of       O1_1 Check
PLAT244_ALERT_4_C Low    'Solvent' Ueq as Compared to Neighbors of       C3_1 Check
PLAT356_ALERT_3_C Short   X-BH3      Distance  B1       - H2             1.10 Ang.
PLAT356_ALERT_3_C Short   X-BH3      Distance  B1       - H79            1.10 Ang.
PLAT911_ALERT_3_C Missing FCF Refl Between Thmin & STh/L=    0.600          2 Report
PLAT934_ALERT_3_C Number of (Iobs-Icalc)/Sigma(W) > 10 Outliers ..          1 Check
```

### Alert level G

```
PLAT083_ALERT_2_G SHELXL Second Parameter in WGHT  Unusually Large       5.54 Why ?
PLAT142_ALERT_4_G s.u. on b - Axis Small or Missing .............    0.00008 Ang.
PLAT143_ALERT_4_G s.u. on c - Axis Small or Missing .............    0.00013 Ang.
PLAT145_ALERT_4_G s.u. on beta     Small or Missing .............     0.0004 Degree
PLAT303_ALERT_2_G Full Occupancy Atom H5        with # Connections       2.00 Check
PLAT303_ALERT_2_G Full Occupancy Atom H78       with # Connections       2.00 Check
PLAT398_ALERT_2_G Deviating  C-O-C    Angle From 120 for O1_1           105.2 Degree
PLAT412_ALERT_2_G Short Intra XH3 .. XHn     H49      ..H80      .       2.12 Ang.
                                                     x,y,z  =      1_555 Check
PLAT720_ALERT_4_G Number of Unusual/Non-Standard Labels .........          11 Note
PLAT779_ALERT_4_G Suspect or Irrelevant (Bond) Angle(s) in CIF ...      37.50 Deg.
              NA2  -B2   -H5      1_555   1_555   1_555 ......     #   68 Check
PLAT779_ALERT_4_G Suspect or Irrelevant (Bond) Angle(s) in CIF ...      36.50 Deg.
              O3   -S2   -NA2     1_555   1_555   1_555 ......     #  135 Check
PLAT793_ALERT_4_G Model has Chirality at S1          (Centro SPGR)          S Verify
PLAT793_ALERT_4_G Model has Chirality at S2          (Centro SPGR)          S Verify
PLAT793_ALERT_4_G Model has Chirality at S3          (Centro SPGR)          R Verify
PLAT793_ALERT_4_G Model has Chirality at S4          (Centro SPGR)          S Verify
PLAT883_ALERT_1_G No Info/Value for _atom_sites_solution_primary .     Please Do !
PLAT912_ALERT_4_G Missing # of FCF Reflections Above STh/L=  0.600        256 Note
PLAT933_ALERT_2_G Number of HKL-OMIT Records in Embedded .res File          2 Note
PLAT978_ALERT_2_G Number C-C Bonds with Positive Residual Density.          5 Info
```

```
   0 ALERT level A = Most likely a serious problem - resolve or explain
   0 ALERT level B = A potentially serious problem, consider carefully
   9 ALERT level C = Check. Ensure it is not caused by an omission or oversight
  19 ALERT level G = General information/check it is not something unexpected

   1 ALERT type 1 CIF construction/syntax error, inconsistent or missing data
  10 ALERT type 2 Indicator that the structure model may be wrong or deficient
   4 ALERT type 3 Indicator that the structure quality may be low
  13 ALERT type 4 Improvement, methodology, query or suggestion
   0 ALERT type 5 Informative message, check
```

# checkCIF publication errors

**Alert level G**

```
PUBL017_ALERT_1_G The _publ_section_references section is missing or
            empty.
```

```
   0 ALERT level A = Data missing that is essential or data in wrong format
   1 ALERT level G = General alerts. Data that may be required is missing
```

## Publication of your CIF

You should attempt to resolve as many as possible of the alerts in all categories. Often the minor alerts point to easily fixed oversights, errors and omissions in your CIF or refinement strategy, so attention to these fine details can be worthwhile. In order to resolve some of the more serious problems it may be necessary to carry out additional measurements or structure refinements. However, the nature of your study may justify the reported deviations from journal submission requirements and the more serious of these should be commented upon in the discussion or experimental section of a paper or in the "special_details" fields of the CIF. *checkCIF* was carefully designed to identify outliers and unusual parameters, but every test has its limitations and alerts that are not important in a particular case may appear. Conversely, the absence of alerts does not guarantee there are no aspects of the results needing attention. It is up to the individual to critically assess their own results and, if necessary, seek expert advice.

If you wish to submit your CIF for publication in Acta Crystallographica Section C or E, you should upload your CIF via the web. If you wish to submit your CIF for publication in IUCrData you should upload your CIF via the web. If your CIF is to form part of a submission to another IUCr journal, you will be asked, either during electronic submission or by the Co-editor handling your paper, to upload your CIF via our web site.

**PLATON version of 18/12/2021; check.def file version of 18/12/2021**

**Datablock JAZ-520** - ellipsoid plot

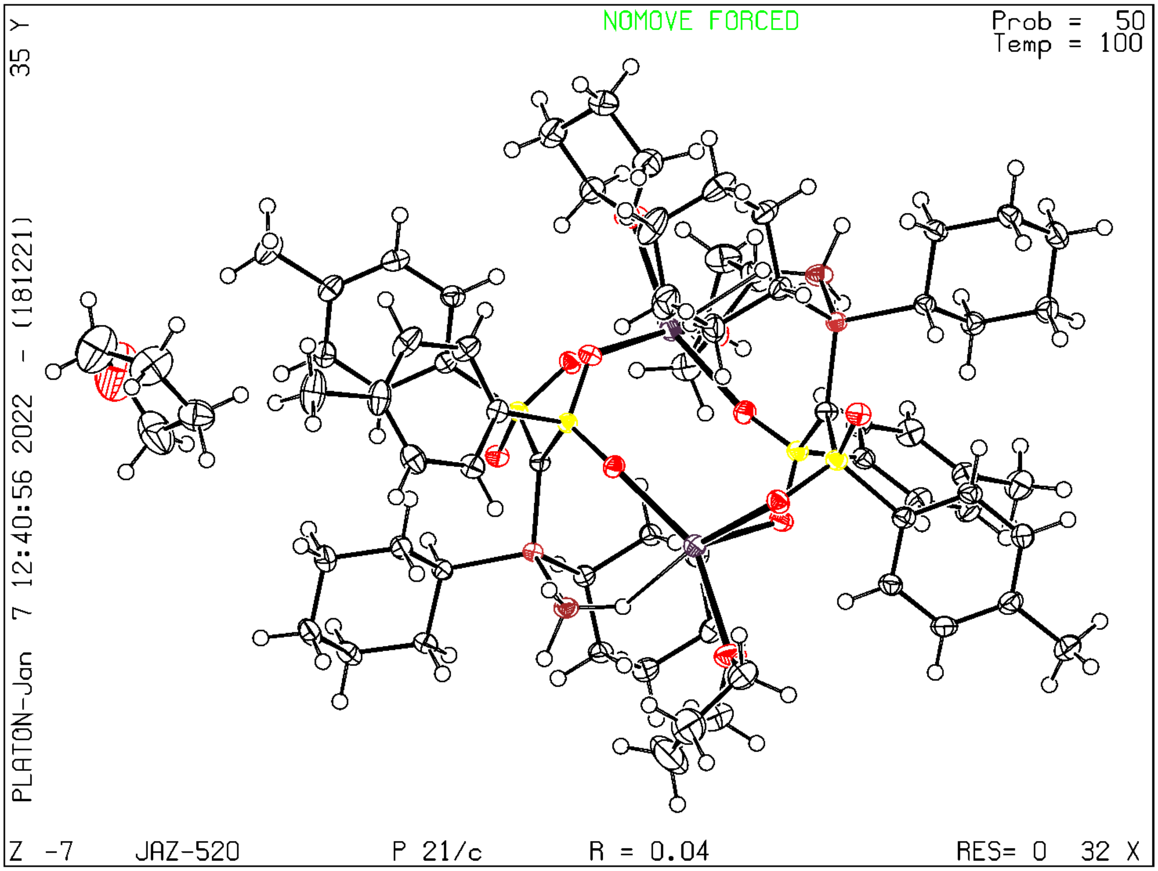

# checkCIF/PLATON report

Structure factors have been supplied for datablock(s) JAZ-508_2_a

THIS REPORT IS FOR GUIDANCE ONLY. IF USED AS PART OF A REVIEW PROCEDURE FOR PUBLICATION, IT SHOULD NOT REPLACE THE EXPERTISE OF AN EXPERIENCED CRYSTALLOGRAPHIC REFEREE.

No syntax errors found.     CIF dictionary     Interpreting this report

# Datablock: JAZ-508_2_a

---

Bond precision: C-C = 0.0075 A     Wavelength=1.54184

| | | | |
|---|---|---|---|
| Cell: | a=20.6243(1) | b=12.1486(1) | c=32.5383(2) |
| | alpha=90 | beta=90 | gamma=90 |
| Temperature: | 100 K | | |

| | Calculated | Reported |
|---|---|---|
| Volume | 8152.68(9) | 8152.68(9) |
| Space group | P n a 21 | P n a 21 |
| Hall group | P 2c -2n | P 2c -2n |
| Moiety formula | C35 H50 K N2 O10 P S3, C35 H54 K N2 O10 P S3 | C35 H50 K N2 O10 P S3, C35 H54 K N2 O10 P S3 |
| Sum formula | C70 H104 K2 N4 O20 P2 S6 | C70 H104 K2 N4 O20 P2 S6 |
| Mr | 1654.07 | 1654.07 |
| Dx,g cm-3 | 1.348 | 1.348 |
| Z | 4 | 4 |
| Mu (mm-1) | 3.411 | 3.411 |
| F000 | 3504.0 | 3504.0 |
| F000’ | 3526.69 | |
| h,k,lmax | 26,15,41 | 26,15,40 |
| Nref | 17306[ 8811] | 16118 |
| Tmin,Tmax | 0.544,0.759 | 0.446,1.000 |
| Tmin’ | 0.493 | |

Correction method= # Reported T Limits: Tmin=0.446 Tmax=1.000
AbsCorr = GAUSSIAN

Data completeness= 1.83/0.93     Theta(max)= 77.297

R(reflections)= 0.0423( 15597)     wR2(reflections)= 0.1121( 16118)

S = 1.024     Npar= 938

The following ALERTS were generated. Each ALERT has the format
      **test-name_ALERT_alert-type_alert-level.**
Click on the hyperlinks for more details of the test.

### Alert level C

```
STRVA01_ALERT_4_C           Flack test results are ambiguous.
           From the CIF: _refine_ls_abs_structure_Flack    0.470
           From the CIF: _refine_ls_abs_structure_Flack_su    0.011
PLAT094_ALERT_2_C Ratio of Maximum / Minimum Residual Density ....       2.41 Report
PLAT241_ALERT_2_C High   'MainMol' Ueq as Compared to Neighbors of        C55 Check
PLAT340_ALERT_3_C Low Bond Precision on  C-C Bonds ...............    0.00746 Ang.
PLAT411_ALERT_2_C Short Inter H...H Contact  H10       ..H23B     .       2.12 Ang.
                                              1/2+x,1/2-y,z  =      3_555 Check
PLAT411_ALERT_2_C Short Inter H...H Contact  H35       ..H49B     .       2.11 Ang.
                                              1-x,1-y,1/2+z  =      2_665 Check
PLAT975_ALERT_2_C Check Calcd Resid. Dens.  0.97Ang From C18      .       0.48 eA-3
PLAT975_ALERT_2_C Check Calcd Resid. Dens.  0.99Ang From C12      .       0.48 eA-3
PLAT975_ALERT_2_C Check Calcd Resid. Dens.  0.87Ang From O5       .       0.42 eA-3
```

### Alert level G

```
PLAT083_ALERT_2_G SHELXL Second Parameter in WGHT  Unusually Large       6.73 Why ?
PLAT142_ALERT_4_G s.u. on b - Axis Small or Missing .............    0.00010 Ang.
PLAT143_ALERT_4_G s.u. on c - Axis Small or Missing .............    0.00020 Ang.
PLAT343_ALERT_2_G Unusual sp?      Angle Range in Main Residue for        C12 Check
PLAT343_ALERT_2_G Unusual sp?      Angle Range in Main Residue for        C18 Check
PLAT367_ALERT_2_G Long?  C(sp?)-C(sp?) Bond  C12       - C13      .       1.54 Ang.
PLAT367_ALERT_2_G Long?  C(sp?)-C(sp?) Bond  C12       - C17      .       1.53 Ang.
PLAT367_ALERT_2_G Long?  C(sp?)-C(sp?) Bond  C18       - C19      .       1.53 Ang.
PLAT367_ALERT_2_G Long?  C(sp?)-C(sp?) Bond  C18       - C23      .       1.54 Ang.
PLAT720_ALERT_4_G Number of Unusual/Non-Standard Labels ..........          3 Note
PLAT792_ALERT_1_G Model has Chirality at S1          (Polar  SPGR)          R Verify
PLAT792_ALERT_1_G Model has Chirality at S4          (Polar  SPGR)          R Verify
PLAT883_ALERT_1_G No Info/Value for _atom_sites_solution_primary .     Please Do !
PLAT910_ALERT_3_G Missing # of FCF Reflection(s) Below Theta(Min).          1 Note
PLAT912_ALERT_4_G Missing # of FCF Reflections Above STh/L=  0.600         71 Note
PLAT978_ALERT_2_G Number C-C Bonds with Positive Residual Density.          0 Info
```

0 **ALERT level A** = Most likely a serious problem - resolve or explain
0 **ALERT level B** = A potentially serious problem, consider carefully
9 **ALERT level C** = Check. Ensure it is not caused by an omission or oversight
16 **ALERT level G** = General information/check it is not something unexpected

3 ALERT type 1 CIF construction/syntax error, inconsistent or missing data
15 ALERT type 2 Indicator that the structure model may be wrong or deficient
2 ALERT type 3 Indicator that the structure quality may be low
5 ALERT type 4 Improvement, methodology, query or suggestion
0 ALERT type 5 Informative message, check

## checkCIF publication errors

### Alert level G

```
PUBL017_ALERT_1_G The _publ_section_references section is missing or
            empty.
```

```
   0 ALERT level A = Data missing that is essential or data in wrong format
   1 ALERT level G = General alerts. Data that may be required is missing
```

## Publication of your CIF

You should attempt to resolve as many as possible of the alerts in all categories. Often the minor alerts point to easily fixed oversights, errors and omissions in your CIF or refinement strategy, so attention to these fine details can be worthwhile. In order to resolve some of the more serious problems it may be necessary to carry out additional measurements or structure refinements. However, the nature of your study may justify the reported deviations from journal submission requirements and the more serious of these should be commented upon in the discussion or experimental section of a paper or in the "special_details" fields of the CIF. *checkCIF* was carefully designed to identify outliers and unusual parameters, but every test has its limitations and alerts that are not important in a particular case may appear. Conversely, the absence of alerts does not guarantee there are no aspects of the results needing attention. It is up to the individual to critically assess their own results and, if necessary, seek expert advice.

If you wish to submit your CIF for publication in Acta Crystallographica Section C or E, you should upload your CIF via the web. If you wish to submit your CIF for publication in IUCrData you should upload your CIF via the web. If your CIF is to form part of a submission to another IUCr journal, you will be asked, either during electronic submission or by the Co-editor handling your paper, to upload your CIF via our web site.

**PLATON version of 18/12/2021; check.def file version of 18/12/2021**

**Datablock JAZ-508_2_a** - ellipsoid plot

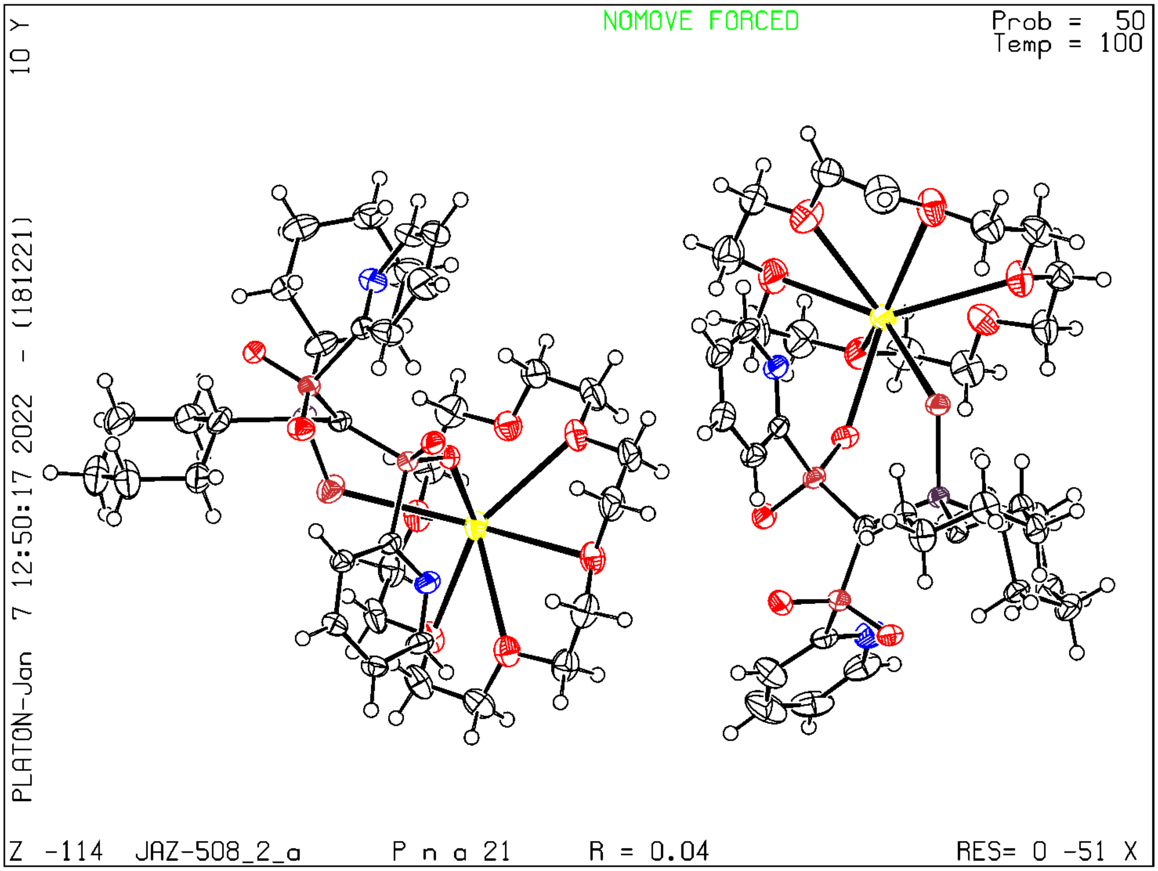

Supplement: Supplementary file 2 — Supporting Information [file ANIE-61-0-s001.pdf]
