# Supplementary material for: From Stable PH‐Ylides to α‐Carbanionic Phosphines as Ligands for Zwitterionic Catalysts
Source: Angew Chem Int Ed Engl. 2022 Jun 14;61(30):e202203950. doi: 10.1002/anie.202203950 (PMC9401067; doi:10.1002/anie.202203950)
Supplement: Supplementary file 3 — Supporting Information [file ANIE-61-0-s002.pdf]

Angewandte Chemie
Eine Zeitschrift der Gesellschaft Deutscher Chemiker

Supporting Information

From Stable PH-Ylides to α-Carbanionic Phosphines as Ligands for Zwitterionic Catalysts

*J.-A. Zur, M. Schmidt, K.-S. Feichtner, P. Duari, J. Löffler, T. Scherpf, V. H. Gessner**

SUPPORTING INFORMATION

Table of Contents

1. Experimental Procedures **3**

1.1. Reaction conditions 3

1.2. Analytical methods 3

1.3. bis(p-toluenesulfonyl)methane (**1a**) and bis(2-pyridylsulfonyl)methane (**1b**) 3

1.4. Preparation of the (bissulfonyl)methanides **2a** and **2b** 4

1.5. Preparation of **3a-Cy/3a'-Cy** 5

1.6. Preparation of **4a-Cy** 6

1.7. Preparation of **3a-iPr/3a'-iPr** 6

1.8. Preparation of **4a-Ph** 6

1.9. Preparation of **3a'-Ph** 7

1.10. Preparation of **3b-Cy** and **4b-Cy** 7

1.11. Preparation of **5** and **6** 8

1.12. Preparation of gold complex [(**3b**-Cy)AuCl)] and [(**4b**-Cy)·Au(PPh_3)] 9

1.13. Procedure for the determination of the ylide/phosphine equilibria 10

1.14 Procedure for the preparation of Phosphineselenides 10

1.15 Procedure for the gold-catalysed hydroamination reaction 12

1 NMR Spectra **13**

2.1 NMR Spectra of the isolated compounds 13

2.2 NMR spectra for determination of the ylide/phosphine equilibria 35

2.3 NMR spectra of the gold complexes 39

2.4 NMR spectra of the phosphine selenides for TEP determination 42

3. Crystal Data **49**

3.1 Crystal data and structure refinement details for all compounds 49

3.2 Crystal Strucuture Determination of **1a** and **1b** 56

3.3 Crystal Structure Determination of **2a** and **2b** 58

3.4 Crystal Structure Determination of **3a-Cy** 65

3.5 Crystal Structure Determination of **4a-Cy** 67

3.6 Crystal Structure Determination of **3a-iPr** 71

3.7 Crystal Structure Determination of **3a-Ph** 73

3.8 Crystal Structure Determination of **3b-Cy** 76

3.9 Crystal Structure Determination of **4a-Ph** 79

3.10 Crystal Structure Determination of **5a** and **5b** 84

3.11 Crystal Structure Determination of **6a** and **6b** 90

3.12 Crystal Structure Determination of the gold complexes 102

4. Calculations **108**

4.1. Computational Details 108

4.2. Results of DFT calculations 109

4.2 Calculation of Tolman Electronic Parameter (TEP) 111

4.3 Proton affinities 113

4.4. Connolly surfaces mapped with electrostatic potential 115

4.5. Cartesian Coordinates 116

5. References **150**

1. Experimental Procedures

1.1. Reaction conditions

All experiments (if not stated otherwise) were carried out under a dry, oxygen-free argon atmosphere using standard Schlenk techniques. Argon (99.999%) was purchased from *Air Liquide*.

Solvents and chemicals. Involved solvents were dried using an MBraun SPS 800 (THF, toluene, diethyl ether, *n*-hexane) or dried in accordance with standard procedures and stored under an argon atmosphere over 3 Å or 4 Å molecular sieves. Chlorodicyclohexylphosphine was prepared according to literature procedure.[1] All other reagents were purchased from Sigma Aldrich, ABCR, Rockwood Lithium or Acros Organics and used without further purification. References to the other compounds and synthetic procedures used from literature references are given below.

1.2. Analytical methods

NMR spectra. ^{1}H, $^{11}B\{^{1}H\}$, $^{13}C\{^{1}H\}$, $^{31}P\{^{1}H\}$ and $^{77}Se\{^{1}H\}$ NMR spectra were recorded on an Avance III 400 spectrometer from Bruker at 25°C (if not stated otherwise). All values of the chemical shift are in ppm regarding the δ-scale. All spin-spin coupling constants (J) are given in Hertz (Hz). For multiplicities and signal forms the following abbreviations were used: s = singlet, d = doublet, t = triplet, m = multiplet, dd = doublet of doublet, dt = doublet of triplet, ddd= doublet of doublet of doublet, br = broad signal. Signal assignment was supported by DEPT, APT, HSQC, HMBC and COSY experiments and by literature studies on similar compounds.

Elemental analyses were performed on an Elementar vario MICRO cube elemental analyzer in the in-house analytical facility.

IR-Spectra were recorded on a Thermo Nicolet iS5 FT-IR in transmission mode with a Specac “Omni-cell” with KBr plates and a 0.1 mm spacer or with an ATR module at 22 °C.

Melting points were recorded on a Stuart SMP 30 with a heat up speed of 2 °C per minute.

X-ray crystallography: Details are given in section 3.

1.3. bis(*p*-toluenesulfonyl)methane (1a) and bis(2-pyridylsulfonyl)methane (1b)

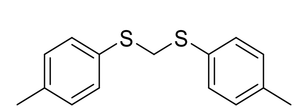

(adapted from literature)[2]

20.2 mL (143.0 mmol) of triethylamine were dissolved in 150 mL of acetonitrile and cooled to 0 °C. 17.8 g (143.0 mmol) of thiocresol were added in portions to the colorless solution and 5.78 mL (71.1 mmol) of diiodomethane were added. The solution stirred for one hour at 0 °C. During this time the solution turned yellow and changed back to colorless. The rection mixture was stirred overnight at room temperature. 100 mL water were added to the reaction mixture, the layers were separated, and the aqueous layer was extracted four times with diethyl ether. The collected organic phases were dried over sodium sulphate and the solvent was removed under reduced pressure. The resulting oil was cooled to induce crystallization. The resulting solid was washed two times with cold methanol. The product was dried *in vacuo* and obtained as colorless solid (15.4 g, 59.1 mmol, 82%).

^{1}H NMR (80.10 MHz, chloroform-d[1]): δ = 2.43 (s, 6H, CH_3), 4.36 (s, 2H, SCH_2S), 7.16-7.58 (m, 8H, C$H_{arom.}$) ppm.

Other spectroscopic data match those reported in literature.[3]

(adapted from literature)[2]

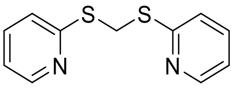

1.27 mL (9.00 mmol) triethylamine were dissolved in 5 mL of acetonitrile and were cooled to 0 °C. 1.00 g (9.00 mmol) mercaptopyridine and 0.365 mL (4.50 mmol) of diiodomethane were added to the solution and the reaction mixture was stirred for one hour at 0 °C before the reaction was allowed to warm to room temperature and was stirred overnight. 10 mL water were added to the solution, the layers were separated, and the aqueous layer was extracted three times with diethyl ether. The collected organic phases were dried with sodium sulphate. The solvent was evaporated yielding a yellow solid. This solid was recrystallized from ethanol to obtain the product as light yellow needles (820 mg, 3.49 mmol, 78 %).

^{1}H NMR (400.1 MHz, chloroform-d^1): δ = 5.08 (s, 2H, SC*H*$_2$S), 7.02 (dd, $^3J_{HH}$ = 7.3 Hz, $^4J_{HH}$ = 5.0 Hz, 2H, C*H*$_{arom.}$), 7.17-7.19 (m, 2H, S C*H*$_{arom.\ ortho}$), 7.47-7.51 (m, 2H, C*H*$_{arom.}$), 8.49-8.50 (m, 2H, NC*H*) ppm.

Other spectroscopic data match those reported in literature.[4]

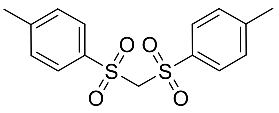

4.81 g (18.47 mmol) of bis(*p*-tolyl) thiomethane were suspended in 30 mL acetic acid. 18.9 mL of hydrogen peroxide in water (184.7 mmol, 30% w/w) were added slowly to the suspension. During this the suspension turned yellow. The reaction mixture was heated to 100 °C for five hours. Upon cooling to room temperature, the product precipitated from the solution. The solid was filtered off and was washed with water till no peroxides were present in the filtrate. The solid was washed with cold ethanol and yielded the product as colorless solid (4.20 g, 12.95 mmol, 70 %).

^{1}H NMR (400.1 MHz, chloroform-d^1): δ = 2.47 (s, 6H, C*H*$_3$), 4.68 (s, 2H, SC*H*$_2$S), 7.37 (d, 4H, $^3J_{HH}$ = 8.0 Hz, C*H*$_{arom.}$), 7.84 (d, 4H, $^3J_{HH}$ = 8.0 Hz, C*H*$_{arom.}$) ppm. **^{13}C{^{1}H} NMR** (101.7 MHz, DMSO-d^6): δ = 21.1 (*C*H$_3$), 72.0 (S*C*H$_2$S), 128.3 (*C*H$_{arom.}$), 129.5 (*C*H$_{arom.}$), 135.9 (*C*$_{arom.\ para}$), 145.0 (*C*$_{arom.\ ipso}$) ppm. **Anal. Calcd.** for $C_{15}H_{16}O_4S_2$**:** C 55.54, H 4.97, O 19.73, S 19.77, found C 55.54, H 4.88, S 19.49.

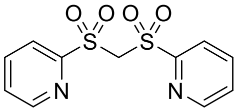

10.0 g (42.7 mmol) of bis(2-pyridyl) thiomethane were dissolved in 500 mL chloroform. 42.1 g (171 mmol) *meta*-chloroperoxybenzoic acid were added in portions to the yellow solution. The solution was heated under reflux conditions for 19 hours. The solvent was removed *in vacuo*. The white solid was washed with 150 mL ethyl acetate and was recrystallized from hot THF giving colorless needles (6.01 g, 20.1 mmol, 47 %).

^{1}H NMR (400.1 MHz, DMSO-d^6): δ = 5.98 (s, 2H; SC*H*$_2$S), 7.77 (dd, $^3J_{HH}$ = 7.7 Hz, $^3J_{HH}$ = 4.7 Hz, 2H; C*H*$_{arom}$), 7.99 (d, $^3J_{HH}$ = 7.9 Hz, 2H; C*H*$_{arom}$), 8.15 (td, $^3J_{HH}$ = 7.8 Hz, $^4J_{HH}$ = 1.7 Hz, 2H; C*H*$_{arom}$), 8.75 (d, $^3J_{HH}$ = 4.7 Hz, 2H; NC*H*) ppm. **^{13}C{^{1}H}-NMR:** (100.7 MHz, d^6-DMSO): δ = 65.3 (SCH2S), 122.4 (*C*H$_{arom}$), 128.6 (*C*H$_{arom}$), 139.2 (*C*H$_{arom}$), 150.3 (NC*C*H), 155.7 (S*C*S). **m.p.** 178.9 °C. **Anal. Calcd.** C 44.28, H 3.38, N 9.39, S 21.50, found C 44.49, H 3.42, N 9.18, S 21.88.

1.4. Preparation of the (bissulfonyl)methanides 2a and 2b

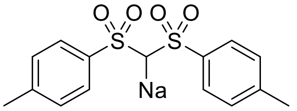

2a-Na: 1.50 g (4.62 mmol) **1a** and 110 mg (4.62 mmol) of sodium hydride were suspended in diethyl ether. The suspension was stirred until the hydrogen evolution was finished. The unreacted sodium hydride was filtered off. The filtrate was evaporated under reduced pressure and the product was obtained as light-yellow solid (1.58 g, 4.57 mmol, 99 %).

^{1}H NMR (400.1 MHz, DMSO-d^6): δ = 2.48 (s, 6H, C*H*$_3$), 3.81 (s, 1H, SC*H*S), 7.27 (d, $^3J_{HH}$ = 7.8 Hz, 4H, C*H*$_{arom.\ meta}$), 7.72 (d, $^3J_{HH}$ = 7.8 Hz, 4H, C*H*$_{arom.\ ortho}$) ppm. **^{13}C{^{1}H} NMR** (100.7 MHz, DMSO-d^6): δ = 20.8 (*C*H$_3$), 64.2 (S*C*HS), 125.2 (*C*H$_{arom.\ ortho}$), 128.1 (*C*H$_{arom.\ meta}$), 138.4 (*C*$_{arom.\ para}$), 147.5 (*C*$_{arom.\ ipso}$) ppm. **FT-IR** (KBr) ṽ [cm^{-1}] = 450 (s), 650 (s), 804 (m), 817 (m), 1073 (s), 1120 (s), 1130 (s), 1205 (m), 1270 (s), 1400 (w), 1490 (w), 1600 (w), 2860 (s),

2915 (s), 2860 (m), 3020 (m), 3060 (s). **m. p**. decomposition at 304.8 °C. **Anal. Calcd.** for $C_{15}H_{15}O_4S_2Na$: C 52.01, H 4.37, S 18.51, found C 51.48, H 4.27, S 18.23.

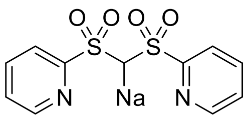

2b-Na. 4.00 g (13.4 mmol) of **1b** and 322 mg (13.4 mmol) NaH were suspended in 30 ml THF. The mixture was stirred until no further gas evolution could be detected, filtered and the obtained colourless solid dried *in vacuo*. (4.10 g, 12.8 mmol, 96 %).

^{1}H NMR (400.1 MHz, DMSO-d^6): δ = 3.81 (s, 1H, SC*H*S), 7.27-7.30 (m, 2H; C*H*$_{arom}$), 7.65-7.67 (m, 2H; SCC*H*), 7.71-7.74 (m, 2H; C*H*$_{arom}$), 8.32 (d, $^3J_{HH}$= 4.6 Hz, 2H; NC*H*). **^{13}C{^{1}H}-NMR:** (100.7 MHz, d^6-DMSO): δ = 62.0 (S*C*HS), 119.6 (*C*H$_{arom}$), 124.1 (*C*H$_{arom}$), 137.1 (*C*H$_{arom}$), 148.6 (N*C*H), 164.0 (S*C*). **Anal. Calcd.** C 41.25, H 2.83, N 8.75, S 20.02 found C 41.15, H 2.85, N 8.71, S 19.46.

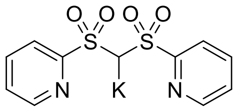

2b-K. 200 mg (670 µmol) of **1b** and 134 mg (670 µmol) of KHMDS were dissolved in 6 ml THF and the resulting suspension stirred for 18 h at RT. The solvent was removed under reduced pressure and the resulting residue washed five times with 10 ml pentane. Drying in vacuo gave **2b-K** as colourless solid in 91 % (205 mg, 609 µmol) yield.

^{1}H NMR (400.3 MHz, d^6-DMSO): δ = 3.78 (s, 1H; SC*H*S), 7.28 (ddd, $^3J_{HH}$ = 7.4 Hz, $^3J_{HH}$ = 4.7Hz, $^4J_{HH}$ = 1.3 Hz, 2H; C*H*$_{arom}$), 7.62-7.65 (m, 2H; SCC*H*), 7.69-7.74 (m, 2H; C*H*$_{arom}$), 8.30-8.32 (m, 2H; NC*H*). **^{13}C{^{1}H} NMR** (100.7 MHz, d^6-DMSO): 61.8 (S*C*HS), 119.67(SC*C*H), 123.9 (*C*Harom), 136.9 (*C*Harom), 148.5 (N*C*H), 164.2 (N*C*). **Anal. Calcd.** for $C_{11}H_9KN_2O_4S_2$: C 39.27, H 2.70. N 8.33, S 19.06, found C 38.98, H 2.73, N 8.42, S 18.52.

1.5. Preparation of 3a-Cy/3a'-Cy

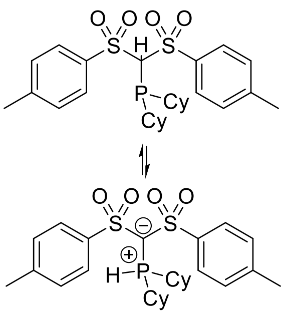

3a-Cy$_2$

3a'-Cy$_2$

950 mg (2.93 mmol) of **2a** were suspended in 40 mL THF and cooled down to –78 °C. 716 mg (3.07 mmol) of chlorodicyclohexylphosphine were added to the suspension. The reaction mixture was stirred for 17 h, during which it turned yellowish and cloudy. The solvent was removed *in vacuo* and the crude residue was washed four times with diethyl ether. The residue was dissolved in dichloromethane and the formed sodium chloride was filtered off. The solvent was evaporated and the residue was recrystallized from toluene. The supernatant solution was removed, and the remaining solid was washed with hexane. After drying the product was obtained as colorless solid (854 mg, 1.64 mmol, 56 %).

^{1}H NMR (400.1 MHz, THF-d^8) (signals assigned proportionally for **12-CH** (~15 %), wherever possible)**: 12-CH** δ = 2.43 (s, 1H, C*H*$_3$), 2.43-2.53 (m, 0.5 H, C*H*$_{Cy\text{-}1}$), 4.89 (d, $^3J_{PH}$ = 1.43 Hz, 0.15H, SC*H*S), 7.35 (d, $^3J_{HH}$ = 8.6 Hz, 0.7H, C*H*$_{arom.\ meta}$), 7.83 (d, $^3J_{HH}$ = 8.6 Hz, 0.7H, C*H*$_{arom.\ ortho}$) ppm. **12-PH** δ =1.03-1.25 (m, 7H, C*H*$_{2\ Cy}$), 1.26-1.34 (m, 5H, C*H*$_{2\ Cy}$), 1.59-1.80 (m, 8H, C*H*$_{2\ Cy}$), 1.82-1.92 (m, 5H, C*H*$_{2\ Cy}$), 2.38 (s, 6H, C*H*$_3$), 2.45-2.55 (m, 2H, C*H*$_{Cy\text{-}1}$), 6.02 (dt, $^1J_{PH}$ = 447.8 Hz, $^3J_{HH}$ = 7.5 Hz, 1H, P*H*), 7.22 (d, $^3J_{HH}$ = 8.20 Hz, 4H, C*H*$_{arom.\ meta}$), 7.91 (d, $^3J_{HH}$ = 8.20 Hz, 4H, C*H*$_{arom.\ ortho}$) ppm. The sum of the cyclohexyl protons is 27 in total for **12-PH**. As the peaks of both tautomers overlap a separation is not possible. Thus, roughly five of the assigned protons belong to **12-CH**. **^{13}C{^{1}H} NMR** (101.7 MHz, THF-d^8): **12-CH** δ = 21.5 (*C*H$_3$), 26.5 (*C*H$_{2\ Cy}$), 27.2 (*C*H$_{2\ Cy}$), 27.4 (*C*H$_{2\ Cy}$), 28.5 (*C*H$_{2\ Cy}$), 29.8 (*C*H$_{2\ Cy}$), 33.7 (d, $^1J_{PC}$ = 49.4 Hz, *C*H$_{Cy\text{-}1}$), 64.1 (d, $^1J_{PC}$ = 97.9 Hz, S*C*HS), 128.4 (*C*H$_{arom.\ ortho}$) ,129.4 (*C*H$_{arom.\ meta}$), 142.3 (*C*$_{arom.\ para}$), 146.1 (*C*$_{arom.\ ipso}$) ppm. **12-PH** δ = 21.6 (*C*H$_3$), 26.6 (*C*H$_{2\ Cy}$), 27.1 (*C*H$_{2\ Cy}$), 27.9 (J_{PC} = 10.3 Hz, *C*H$_{2\ Cy}$), 28.5 (*C*H$_{2\ Cy}$), 31.3 (d, J_{PC} = 15.4 Hz, *C*H$_{2\ Cy}$), 31.8 (d, $^1J_{PC}$ = 19.6 Hz; *C*H$_{Cy\text{-}1}$), 81.2 (S*C*S), 130.0 (*C*H$_{arom.\ meta}$), 130.8 (*C*H$_{arom.\ ortho}$), 131.3 (*C*$_{arom.\ para}$), 145.8 (*C*$_{arom.\ ipso}$) ppm. **^{31}P{^{1}H} NMR** (162.1 MHz, THF-d^8): **12-CH** δ = 17.4 ppm, **12-PH** δ = 19.8 ppm. **FT-IR (KBr)** ṽ [cm^{-1}] = 550 (s), 660 (s), 810 (s), 1140 (s), 1180 (s), 1300 (s), 1450 (m), 1490 (m), 1600 (s), 2920 (m), 2850 (m). **m. p.** 172.9 °C. **Anal. Calcd .** for $C_{27}H_{37}O_4PS_2$: C 62.28, H 7.16, S 12.31, found C 61.86, H 6.78, S 12.67.

1.6. Preparation of 4a-Cy

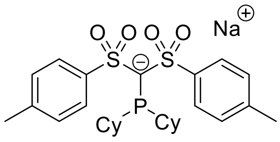

37.0 mg (72.0 µmol) of **3a-Cy$_2$** and 2.00 mg (76.0 µmol) sodium hydride were suspended in 5 mL THF. The colorless suspension was stirred until the hydrogen evolution was finished. The suspension was filtered, and the solvent was removed under reduced pressure. The product was obtained as colorless solid (31.9 mg, 61 µmol, 82 %).

^{1}H NMR (400.1 MHz, THF-d^8): 0.65-0.77 (m, 2H, C*H*$_{2\ \mathrm{Cy}}$), 0.97-1.10 (m, 6H, C*H*$_{2\ \mathrm{Cy}}$), 1.11-1.21 (m, 2H, C*H*$_{2\ \mathrm{Cy}}$), 1.34-1.44 (m, 2H, C*H*$_{2\ \mathrm{Cy}}$), 1.45-1.64 (m, 6H, C*H*$_{2\ \mathrm{Cy}}$), 1.65-1.71 (m, 2H, C*H*$_{2\ \mathrm{Cy}}$), 2.04-2.13 (m, 2H, C*H*$_{\mathrm{Cy\text{-}1}}$), 2.30 (s, 6H, C*H*$_3$), 6.80-6.99 (br, 2H, C*H*$_{\mathrm{arom.\ meta}}$*)*, 7.00-7.20 (br, 2H, C*H*$_{\mathrm{arom.\ meta}}$), 7.23-7.44 (br, 2H, C*H*$_{\mathrm{arom.\ ortho}}$), 7.66-7.81 (br, 2H, C*H*$_{\mathrm{arom.\ ortho}}$) ppm. **^{13}C{^{1}H} NMR** (101.7 MHz, THF-d^8): 21.0 (*C*H$_3$), 27.5 (*C*H$_{2\ \mathrm{Cy}}$),27.8 (s, *C*H$_{2\ \mathrm{Cy}}$), 28.1 (*C*H$_{2\ \mathrm{Cy}}$), 28.2 (d, J_{CP} = 4.24 Hz, *C*H$_{2\ \mathrm{Cy}}$), 31.3 (d, J_{CP} = 11.4 Hz, *C*H$_{2\ \mathrm{Cy}}$), 33.0 (d, J_{CP} = 22.9 Hz, *C*H$_{2\ \mathrm{Cy}}$), 35.8 (d, $^1J_{CP}$ = 11.4 Hz, *C*H$_{\mathrm{Cy\text{-}1}}$), 71.4 (d, $^1J_{CP}$ = 49.1 Hz, S*C*S), 127.4 (*C*H$_{\mathrm{arom.\ ortho}}$), 128.0 (*C*H$_{\mathrm{arom.\ meta}}$), 128.6 (*C*H$_{\mathrm{arom.\ meta}}$), 139.8 (br, 2x *C*$_{\mathrm{arom.\ para}}$), 146.6 (*C*$_{\mathrm{arom.\ ipso}}$), 148.2 (*C*$_{\mathrm{arom.\ ipso}}$) ppm. **^{31}P{^{1}H} NMR** (162.1 MHz, THF-d^8): δ = –0.22 ppm. **m.p.** decomposition at 255.7 °C. **Anal. Calcd.** for $C_{27}H_{36}O_4PS_2Na$: C 59.76, H 6.69, S 11.57, found C 59.76, H 6.68. S 11.57.

1.7. Preparation of 3a-*i*Pr/3a'-*i*Pr

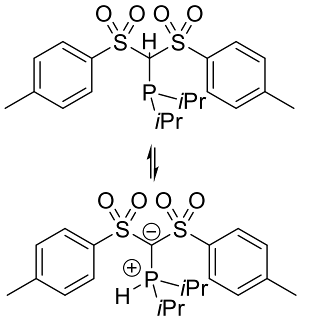

3a-***i*Pr$_2$**

3a'-***i*Pr$_2$**

1.09 g (3.15 mmol) of **2a** were suspended in THF and cooled down to –78 °C. 0.53 mL (3.31 mmol) of chlorodiisopropylphosphine were added dropwise to the suspension. After 12 hours the solvent was removed *in vacuo* and the crude product was washed four times with diethyl ether. The residue was dissolved in toluene and centrifugated to remove the formed sodium chloride. After removal of the solvent under reduced pressure, the residue was recrystallized from toluene. The supernatant solution was removed, and the solid residue was washed with hexane and dried under reduced pressure. This yielded a colorless solid (810 mg, 1.84 mmol, 59 %).

^{1}H NMR (400.1 MHz, THF-d^8): **3a-*i*Pr$_2$** (protons assigned proportionally (~25 %)) δ = 1.04 (dd, 1.5H, $^3J_{HH}$ = 7.0 Hz, $^3J_{PH}$= 15.1 Hz, C*H*$_{3\ \mathrm{P}i\mathrm{Pr}}$), 1.14 (dd, $^3J_{HH}$ = 7.0 Hz, $^3J_{PH}$ = 15.1 Hz, 1.5H C*H*$_{3\ \mathrm{P}i\mathrm{Pr}}$), 2.43 (s, 2H, C*H*$_{3\ p\text{-}\mathrm{Tol}}$), 2.61-2.76 (m, 0.5H, C*H*$_{\mathrm{P}i\mathrm{Pr}}$), 4.84 (d, $^2J_{PH}$ = 1.19 Hz, 0.25H, SC*H*S), 7.35 (d, 1H, $^3J_{HH}$ = 8.4 Hz, C*H*$_{\mathrm{arom.}}$), 7.83 (d, $^3J_{HH}$ = 8.4 Hz, 1H, C*H*$_{\mathrm{arom.}}$) ppm. **3a'-*i*Pr$_2$** δ = 1.10 (dd, 6H, $^3J_{HH}$ = 7.2 Hz, $^3J_{PH}$ = 19.5 Hz, C*H*$_{3\ \mathrm{P}i\mathrm{Pr}}$), 1.21 (dd, $^3J_{HH}$ = 7.2 Hz, $^3J_{PH}$ = 19.5 Hz, 6H, C*H*$_{3\ \mathrm{P}i\mathrm{Pr}}$), 2.37 (s, 6H, C*H*$_{3\ p\text{-}\mathrm{Tol}}$), 2.63-2.76 (m, 2H, C*H*$_{\mathrm{P}i\mathrm{Pr}}$), 6.03 (dt, 1H, $^1J_{PH}$ = 448 Hz, $^3J_{HH}$ = 7.3 Hz, P*H*), 7.22 (d, $^3J_{HH}$ = 8.1 Hz, 4H, C*H*$_{\mathrm{arom.}}$), 7.90 (d, $^3J_{HH}$ = 8.1 Hz, 4H, C*H*$_{\mathrm{arom.}}$) ppm. **^{13}C{^{1}H} NMR** (101.7 MHz, THF-d^8): **3a-*i*Pr$_2$** δ = 20.5 (d, $^2J_{PC}$ = 16.4 Hz, *C*H$_{3\ \mathrm{P}i\mathrm{Pr}}$), 21.7 (CH$_{3\ p\text{-}\mathrm{Tol.}}$), 22.4 (d, $^1J_{PC}$ = 20.5 Hz, *C*H$_{\mathrm{P}i\mathrm{Pr.}}$), 22.5 (d, $^2J_{PC}$ = 19.6 Hz, *C*H$_{3\ \mathrm{P}i\mathrm{Pr}}$), 82.5 (d, $^1J_{PC}$ = 75.4 Hz, S*C*HS), 130.8 (d, $^4J_{PC}$ = 1.1 Hz, *C*H$_{\mathrm{arom.}}$), 130.0 (*C*H$_{\mathrm{arom.}}$), 139.5 (*C*$_{\mathrm{arom.\ para}}$), 146.0 (*C*$_{\mathrm{arom.\ ipso}}$) ppm. **3a'-*i*Pr$_2$** δ = 18.2 (d, $^3J_{PC}$ = 49.7 Hz, *C*H$_{3\ \mathrm{PiPr}}$), 18.7 (*C*H$_{3\ \mathrm{P}i\mathrm{Pr}}$), 21.5 (*C*H$_{3\ p\text{-}\mathrm{Tol}}$), 24.8 (d, $^1J_{PC}$ = 49.7 Hz, *C*H$_{\mathrm{P}i\mathrm{Pr}}$), 65.3 (d, $^1J_{PC}$ = 49.7 Hz, S*C*S), 128.3 (4x *C*H$_{\mathrm{arom.}}$), 129.5 (4x *C*H$_{\mathrm{arom.}}$), 142.3 (*C*$_{\mathrm{arom.\ para}}$), 146.0 (*C*$_{\mathrm{arom.\ ipso}}$) ppm. **^{31}P{^{1}H} NMR** (162.1 MHz, THF-d^8): **3a-*i*Pr$_2$** δ = 26.3 ppm. **3a'-*i*Pr$_2$** δ = 28.9 ppm. **m.p.** 173.4-173.8 °C. **Anal. Calcd.** for $C_{21}H_{29}O_4PS_2$: C 57.25, H 6.63, S 14.56, found C 57.40, H 6.35, S 14.26.

1.8. Preparation of 4a-Ph

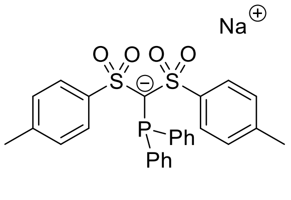

150 mg (0.460 mmol) of **1a** and 28 mg (1.15 mmol) sodium hydride were suspended in THF. The reaction mixture turned light yellow. When the hydrogen evolution was almost finished, 0.09 mL (0.506 mmol) chlorodiphenylphosphine were added dropwise to the solution. After stirring for 20 hours the mixture was filtered over Celite, the solvent was removed under reduced pressure and the residue was washed three times with hexane. The crude product was heated up in toluene when a white solid precipitated from the

solution. The precipitate was dried *in vacuo* and the product was obtained as off-white solid (0.151 g, 0.283 mmol, 62 %).

^{1}H NMR (400.3 MHz, THF-d^8): δ = 2.29 (s, 6H, CH_3), 6.81-6.91 (m, 4H, C$H_{p\text{-Tol meta}}$), 6.92-7.03 (m, 3H, C$H_{p\text{-Tol ortho}}$), 7.04-7.14 (m, 6H, CH_{PPh}), 7.15-7.26 (m, 1H, C$H_{p\text{-Tol ortho}}$), 7.33-7.40 (m, 4H, CH_{PPh}) ppm. **^{13}C{^{1}H} NMR** (100.7 MHz, THF-d^8): δ = 20.3 (CH$_3$), 74.1 (d, $^1J_{PC}$ = 38.8 Hz, SCS), 126.2 (d, $^4J_{PC}$ = 2.0 Hz, CH$_{\text{PPh para}}$), 126.5 (CH$_{p\text{-Tol}}$), 126.7 (d, $^3J_{PC}$ = 6.6 Hz, CH$_{\text{PPh meta}}$), 127.6 (CH$_{p\text{-Tol}}$), 133.3 (d, $^2J_{PC}$ = 20.3 Hz, CH$_{\text{PPh ortho}}$), 139.5 ($C_{p\text{-Tol para}}$), 139.9 (d, $^1J_{PC}$ = 8.3 Hz, $C_{\text{PPh ipso}}$), 145.2 ($C_{p\text{-Tol ipso}}$) ppm. **^{31}P{^{1}H} NMR** (162.1 MHz, THF-d^8): δ = –11.2 ppm. **m.p.** decomposition at 220.6 °C. **Anal. Calcd.** for $C_{27}H_{24}NaO_4PS_2$: C 61.01, H 4.74, S 12.09 found C 61.02. H 4.69, S 11.93.

1.9. Preparation of 3a'-Ph

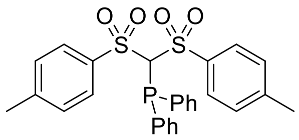

275 mg (0.518 mmol) of **3a-Ph$_2$** were dissolved in THF. 0.260 mL (0.518, 2 mol/L) etheric hydrogen chloride were added to the solution. While adding the hydrogen chloride the solution turned cloudy. After 45 minutes the solvent was removed *in vacuo*. The residue was dissolved in DCM and the salt was filtered off. The solid was washed three times with hexane and dried under reduced pressure. The product was obtained as colorless solid (213 mg, 0.419 mmol, 80 %).

^{1}H NMR (400.1 MHz, DCM-d^2): δ = 2.45 (s, 6H, CH_3), 5.26-5.29 (m, 1H, SCHS), 7.23-7.39 (m, 10H, C$H_{\text{arom.}}$), 7.44-7.51 (m, 4H, C$H_{\text{arom.}}$), 7.67-7.72 (m, 4H, C$H_{\text{arom.}}$) ppm. **^{13}C{^{1}H} NMR** (101.7 MHz, DCM-d^2): δ = 22.0 (CH$_3$), 85.5 (d, $^1J_{PC}$ = 77.4 Hz, SCS), 128.9 (d, $^3J_{PC}$= 7.8 Hz, CH$_{\text{PPh meta}}$), 129.9 (CH$_{p\text{-Tol meta}}$), 130.1 (d, $^4J_{PC}$ = 2.2 Hz, CH$_{\text{PPh para}}$), 130.5 (CH$_{p\text{-Tol ortho}}$), 131.4 (d, $^1J_{PC}$ = 11.0 Hz, $C_{\text{PPh ipso}}$), 134.5 (d, $^2J_{PC}$ = 23.3 Hz, CH$_{\text{PPh ortho}}$), 137.0 ($C_{p\text{-Tol para}}$), 146.2 ($C_{p\text{-Tol ipso}}$) ppm. **^{31}P{^{1}H} NMR** (162.1 MHz, DCM-d^2): δ = –3.41 ppm.

1.10. Preparation of 3b-Cy and 4b-Cy

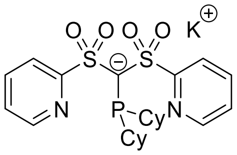

1.50 g (5.03 mmol) of **1b** and 410 mg (10.3 mmol) of potassium hydride were suspended in 15 mL THF and the colorless suspension was stirred for one hour. To the suspension, 1.20 mL (5.28 mmol) of chlorodicyclohexylphosphine were added. The reaction mixture was stirred for sixteen hours during which the resulting reaction mixture turned pink. The remaining potassium hydride was filtered off and the solvent was removed under reduced pressure. The solid was washed three times with 10 mL diethyl ether and was dried *in vacuo*. The crude solid was dissolved in toluene, filtered and left overnight to precipitate. The precipitate was separated from the solvent and was dried under reduced pressure giving the product as light pink solid (1.70 g, 3.19 mmol, 64 %).

^{1}H NMR (400.1 MHz, THF-d^8): δ = 0.51-0.71 (m, 2H, C$H_{2\text{ Cy-2/2'}}$), 1.02-1.25 (m, 8H, C$H_{2\text{, Cy}}$), 1.41-1.59 (m, 4H, C$H_{2\text{ Cy}}$), 1.65 (d, $^2J_{PH}$ = 11.94 Hz, 2H, C$H_{2\text{ Cy}}$), 1.74-1.84 (m, 4H, C$H_{2\text{ Cy}}$), 2.12-2.24 (m, 2H, C$H_{\text{Cy-1}}$), 7.10-7.19 (m, 2H, C$H_{\text{arom. para}}$), 7.1-7.81 (m, 2H, C$H_{\text{arom. meta}}$), 7.98-8.12 (m, 2H, C$H_{\text{arom. ortho}}$), 8.40 (m, 2H, NCH) ppm. **^{13}C{^{1}H} NMR** (101.7 MHz, THF-d^8): δ = 27.5 (CH$_{2\text{ Cy-4}}$), 27.9 (d, $^3J_{CP}$ = 4.20 Hz, CH$_{2\text{ Cy-3/3'}}$), 28.0 (d, $^3J_{CP}$ = 20.0 Hz, CH$_{2\text{ Cy 3/3'}}$), 31.1 (d, $^2J_{CP}$ = 10.8 Hz; CH$_{2\text{ Cy2/2'}}$), 33.0 (d, $^2J_{CP}$ = 22.7 Hz; CH$_{2\text{ Cy 2/2'}}$), 35.8 (d, $^1J_{CP}$ = 11.1 Hz CH$_{\text{Cy-1}}$), 68.2 (SCS), 121.8 (2x CH$_{\text{arom. ortho}}$), 124.2 (CH$_{\text{arom. para}}$), 124.8 (CH$_{\text{arom. para}}$), 137.2 (CH$_{\text{arom. meta}}$), 137.6 (CH$_{\text{arom. meta}}$), 148.6 (NCH), 149.0 (NCH), 165.4 ($C_{\text{arom. ipso}}$), 167.0 ($C_{\text{arom. ipso}}$) ppm. **^{31}P{^{1}H} NMR** (162.1 MHz, THF-d^8): δ = 2.12 ppm. **m. p.** decomposition at 188.9 °C. **Anal. Calcd.** for $C_{23}H_{30}N_2O_4PS_2$: C 51.86, H 5.68. N 5.26, S 12.04, found C 52.11, H 5.87, N 5.58, S 11.78.

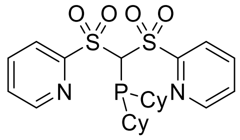

208 mg (390 µmol) of **4b-Cy** were dissolved in 20 mL THF and 195 µl (390 µmol) of etheric hydrochloric acid (2 mol/l) were added dropwise to the light pink THF solution. The reaction mixture was stirred for forty minutes. Then solvent was removed under reduced pressure, the residue was dissolved in dichloromethane and filtered. The solvent was removed, and the product was obtained from recrystallization in toluene as colorless solid (120.0 mg, 242 µmol, 62 %).

^{1}H NMR (400.1 MHz, DCM-d^{2}): δ = 1.19-1.47 (m, 6H, C$H_{2\,Cy}$), 1.54-1.72 (m, 4H, C$H_{2\,Cy\text{-}2/2'}$), 1.73-1.81 (m, 2H, C$H_{2\,Cy}$), 1.82-1.99 (m, 4H, C$H_{2\,Cy}$), 2.01-2.17 (m, 4H, C$H_{2\,Cy\text{-}2/2'}$), 2.58-2.77 (m, 2H, C$H_{Cy\text{-}1}$), 6.30 (dt, $^{1}J_{PH}$ = 456.6 Hz, $^{3}J_{HH}$ = 7.3 Hz, 1H, P*H*), 7.41 (d, $^{3}J_{HH}$ = 7.5 Hz, 2H, C$H_{arom.\,ortho}$), 7.86 (d, $^{3}J_{HH}$ = 7.5 Hz, 2H, C$H_{arom.}$), 7.96-8.02 (m, 2H, C$H_{arom.}$), 8.59 (d, $^{3}J_{HH}$ = 7.5 Hz, 2H, NC*H*) ppm. **^{13}C{^{1}H} NMR** (101.7 MHz, DCM-d^{2}): δ = 26.2 (d, $^{4}J_{CP}$ = 1.7 H, $CH_{2\,Cy\text{-}4}$), 27.0 (d, $^{3}J_{CP}$ = 6.6 Hz, $CH_{2\,C\text{-}3/3'}$), 27.2 (d, $^{3}J_{CP}$ = 6.1 Hz, $CH_{2\,Cy\text{-}3/3'}$), 28.2 (d, $^{2}J_{CP}$ = 3.5 Hz, $CH_{2\,Cy\text{-}2/2'}$), 29.3 ($CH_{2\,Cy\text{-}2/2'}$), 33.6 (d, $^{1}J_{CP}$ = 48.0 Hz, $CH_{Cy\text{-}1}$), 58.7 (d, $^{1}J_{CP}$ = 99.3 Hz, S*C*S), 122.5 ($CH_{arom.}$), 126.3 ($CH_{arom.}$), 138.0 ($CH_{arom.}$), 149.6 (N*C*H), 162.6 ($C_{arom.\,ipso}$) ppm. **^{31}P{^{1}H} NMR** (162.1 MHz, DCM-d^{2}): δ = 23.2 ppm. **FT-IR** (KBr) ṽ [cm^{-1}] = 550 (s), 600 (w), 605 (s), 704 (m), 760 (s), 870 (w), 1030 (w), 1090 (m), 1110 (s), 1160 (s), 1270 (s), 1310 (s), 1430 (m), 1450 (m), 1560 (m), 1575 (m), 2330 (m), 2360 (m), 2850 (s), 2930 (s.), 3060 (w). **m. p.** 172.2-172.9 °C. **Anal. Calcd.** for $C_{23}H_{31}N_2O_4PS_2$: C 55.85, H 6.32, N 5.66, S 12.96, found C 55.43, H 6.32, N 5.73, S 12.70.

1.11. Preparation of 5 and 6

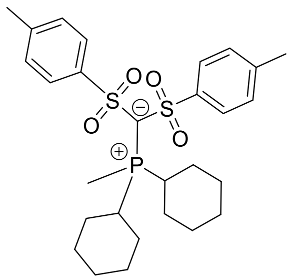

5a. 100 mg (0.192 mmol) of **3a-Cy$_2$** and 35.2 mg of sodium bis(trimethylsilyl)amide (0.192 mmol) were dissolved in THF and stirred for one hour. 12.1 µl (0.192 mmol) of methyl iodide were added to the colorless solution that was stirred for another hour and turned slightly cloudy. The solvent was removed under reduced pressure and the residue was dissolved in acetonitrile. The salt was filtered off, the solvent was evaporated, and the product was washed three times with hexane, dried and was obtained as colorless solid (80.0 mg, 0.150 mmol, 78 %).

^{1}H NMR (400.1 MHz, THF-d^{8}): δ = 0.96-1.11 (m, 2H, C$H_{2\,Cy}$), 1.11-1.21 (m, 2H, C$H_{2\,Cy}$), 1.22-1.41 (6H, m, C$H_{2\,Cy}$), 1.62 (d, $^{2}J_{PH}$ = 12.2 Hz, 3H, PCH_3), 1.67-1.84 (m, 8H, C$H_{2\,Cy}$), 2.37 (s, 6H, CH_3), 2.56-2.70 (m, 2H, C$H_{Cy\text{-}1}$), 7.23 (d, $^{3}J_{HH}$ = 8.0 Hz, 4H, C$H_{arom.\,meta}$), 7.94 (d, $^{3}J_{HH}$ = 8.0 Hz, 4H, C$H_{arom.\,ortho}$) ppm. **^{13}C{^{1}H} NMR** (101.7 MHz, THF-d^{8}): δ = 1.20 (d, $^{1}J_{PC}$ = 54.0 Hz, PCH_3), 20.1 (CH_3), 25.4 (d, $^{4}J_{PC}$ = 1.9 Hz, C$H_{2\,Cy\text{-}4}$), 26.1 (d, $^{3}J_{PC}$ = 1.9 Hz, C$H_{2\,Cy\text{-}3/3'}$), 26.2 (d, $^{3}J_{PC}$= 1.9 Hz, C$H_{2\,Cy\text{-}3/3'}$), 26.3 (d, $^{2}J_{PC}$= 7.1 Hz, C$H_{2\,Cy\text{-}2/2'}$), 27.0 (d, $^{2}J_{PC}$= 2.6 Hz, C$H_{2\,Cy\text{-}2/2'}$), 35.8 (d, $^{1}J_{PC}$ = 52.5 Hz, $CH_{2\,Cy\text{-}1}$), 66.1 (d, $^{1}J_{PC}$ = 93.9 Hz, S*C*S), 127.0 ($CH_{arom.\,ortho}$), 127.9 ($CH_{arom.\,meta}$), 140.7 ($C_{arom.\,para}$), 145.3 ($C_{arom.\,ipso}$) ppm. **^{31}P{^{1}H} NMR** (162.1 MHz, THF-d^{8}): δ = 25.9 ppm.

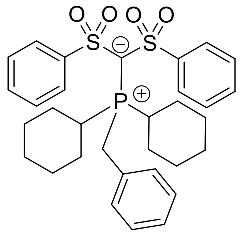

5b. 57 mg (0.107 mmol) of **4b-Cy** were dissolved in THF and 12.8 µl (0.102 mmol) benzyl bromide were added to the solution. After one hour the solution turned significantly cloudy. After three hours the solution was filtered, and the solvent was removed under reduced pressure. Slow diffusion of hexane into a toluene solution of the residue yielded crystals that were washed with hexane. The product was obtained as colorless solid (62.6 mg, 0.077 mmol, 72 %).

^{1}H NMR (400.1 MHz, THF-d^{8}): δ = 0.82-0.91 (m, 1H, C$H_{2\,Cy}$), 1.01-1.44 (m, 8H, C$H_{2\,Cy}$), 1.46-1.69 (m, 5H, C$H_{2\,Cy}$), 1.80-1.98 (m, 4H, C$H_{2\,Cy}$), 2.13-2.34 (br, 2H, C$H_{2\,Cy}$), 2.78-2.98 (br, 2H, C$H_{Cy\text{-}1}$), 4.69 (d, $^{2}J_{PH}$=15.9 Hz, C$H_{2\,benzylic}$), 7.25-7.32 (m, 1H, C$H_{Ph\,para}$), 7.33-7.39 (m, 2H, C$H_{Ph\,meta}$), 7.40-7.43 (m, 2H, C$H_{Pyridyl\,para}$), 7.51-7.56 (m, 2H, C$H_{Ph\,ortho}$), 7.87 (td, 2H, $^{3}J_{PH}$ = 7.72, $^{4}J_{PH}$ = 1,76, C$H_{Pyridyl\,meta}$), 7.99-8.04 (m, 2H, C$H_{Pyridyl\,ortho}$), 8.60-8.64 (m, 2H, NC*H*) ppm. **^{13}C{^{1}H} NMR** (101.7 MHz, THF-d^{8}): δ = 26.3 (d, $^{4}J_{PC}$ = 3.5 Hz, $CH_{2\,Cy\text{-}4}$), 26.9 (d, $^{1}J_{PC}$ = 75.0 Hz, $CH_{2\,benzyl.}$), 26.8 (d, $^{3}J_{PC}$ = 2.6 Hz, $CH_{2\,Cy\text{-}3/3'}$), 27.0 (d, $^{3}J_{PC}$ = 1.4 Hz, $CH_{2\,Cy\text{-}3/3'}$), 27.8 (d, $^{2}J_{PC}$ = 13.5 Hz, $CH_{2\,Cy\text{-}2/2'}$), 28.1 (d, $^{2}J_{PC}$ = 13.6 Hz, $CH_{2\,Cy\text{-}3/3'}$), 34.8 (d, $^{1}J_{PC}$ = 48.9 Hz, $CH_{Cy\text{-}1}$), 68.4 (S*C*S), 123.4

($CH_{Pyridyl\ meta}$), 126.0 ($CH_{Pyridyl\ para}$), 128.4 (d, $^5J_{PC}$ = 3.2 Hz, $CH_{Ph\ para}$), 129.6 (d, $^4J_{PC}$ = 2.6 Hz, $CH_{Ph\ meta}$), 132.4 (d, $^3J_{PC}$ = 5.1 Hz, $CH_{Ph\ ortho}$), 134.4 (d, $^2J_{PC}$ = 6.1 Hz, $C_{Ph\ ipso}$), 138.0 ($CH_{Pyridyl,\ ortho}$), 149.3 (N*C*H), 165.4 ($C_{Pyridyl,\ ipso}$) ppm. **^{31}P{^{1}H} NMR** (162.1 MHz, THF-d^8): δ = 27.9 ppm. **m.p.** 129.1 °C. **Anal. Calcd.** for $C_{30}H_{37}N_2O_4PS_2$: C 61.62, H 6.38, N 4.79, S 10.97, found C 61.35, H 6.30, N 4.83, S 10.69.

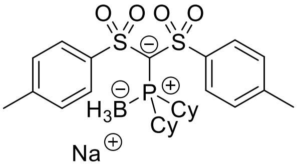

6a. 60 mg (0.115 mmol) of **3a-Cy$_2$**, 21.1 mg (0.115 mmol) of sodium bis(trimethylsilyl)amide and 26.9 mg (0.115 mmol) of borane-1,2-bis (*tert* butylthio)-ethane complex were dissolved in THF and stirred for two hours. The solvent was removed under reduced pressure and the residue washed three times with hexane. The product was obtained as colorless solid (55.6 mg, 0.100 mmol, 87 %).

^{1}H NMR (400.1 MHz, ACN-d^3): δ = 0.25-0.59 (br, 3H, B*H*$_3$), 1.22-1.43 (m, 8H, $CH_{2\ Cy}$), 1.51-1.63 (m, 2H, $CH_{2\ Cy}$), 1.78-1.95 (m, 8H, $CH_{2\ Cy}$), 2.01-2.06 (m, 2H, $CH_{2\ Cy}$), 2.50-2.62 (m, 8H, CH_3, $CH_{Cy\text{-}1}$), 7.34 (d, $^3J_{HH}$ = 8.0 Hz, 4H, $CH_{arom.\ ortho}$), 7.97 (d, $^3J_{HH}$ = 8.0 Hz, 4H, $CH_{arom.\ meta}$) ppm. **^{11}B-NMR** (128.4 MHz, ACN-d^3) = –27.9 (dq, $^1J_{BP}$ = 101 Hz, $^1J_{BH}$ = 33 Hz, *B*H$_3$) ppm. **^{13}C{^{1}H} NMR** (101.7 MHz, ACN-d^3): δ = 21.3 (CH_3), 27.2 (d, $^4J_{PC}$ = 1.8 Hz, $CH_{2\ Cy\text{-}4}$), 27.9 (d, $^3J_{PC}$= 2.8 Hz, $CH_{2\ Cy\text{-}3/3'}$), 28.0 (d, $^3J_{PC}$= 2.8 Hz, $CH_{2\ Cy\text{-}3/3'}$), 28.7 ($CH_{2\ Cy\text{-}2/2'}$), 29.5 ($CH_{2\ Cy\text{-}2/2'}$), 37.2 (d, $^1J_{PC}$= 38.2 Hz, $CH_{Cy\text{-}1}$), 67.8 (d, $^1J_{PC}$ = 42.2 Hz, SCS), 128.0 ($CH_{arom.\ ortho}$), 128.9 ($CH_{arom.\ meta}$), 141.2 ($C_{arom.\ para}$), 147.9 ($C_{arom.\ ipso}$) ppm. **^{31}P{^{1}H} NMR** (162.1 MHz, ACN-d^3): δ = 25.8 (d, $^1J_{PB}$ = 73.6 Hz) ppm.

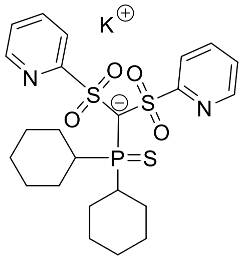

6b. 249.5 mg of **4b-Cy** (0.468 mmol) and 15.0 mg (0.468 mmol) of sulfur were dissolved in THF and the pink solution was stirred overnight. The solvent was removed under reduced pressure. The solid was recrystallized from toluene. The product was obtained as light orange solid (262.0 mg, 0.464 mmol, 99 %).

^{1}H NMR (400.1 MHz, ACN-d^3): δ = 1.05-1.41 (m, 8H, $CH_{2\ Cy}$), 1.47-1.58 (m, 2H, $CH_{2\ Cy}$), 1.59-1.68 (m, 2H, $CH_{2\ Cy}$), 1.68-1.83 (m, 6H, $CH_{2\ Cy}$), 2.08-2.19 (m, 2H, $CH_{2\ Cy}$), 2.53-2.65 (m, 2H, $CH_{Cy\text{-}1}$), 7.39 (dd, J_{HH} = 7.6 Hz, 4.7 Hz, 2H, $CH_{arom.}$), 7.85 (td, J_{HH} = 7.6 Hz, 4.7 Hz, 2H, $CH_{arom.}$), 8.16 (d, $^3J_{HH}$ = 7.6 Hz, 2H, $CH_{arom.\ ortho}$), 8.54 (d, $^3J_{HH}$ = 4.7 Hz, 2H, NC*H*) ppm. **^{13}C{^{1}H} NMR** (101.7 MHz, ACN-d^3): δ = 27.1 (d, $^4J_{PC}$ = 1.5 Hz, $CH_{2\ Cy\text{-}4}$), 27.1-27.7 (m, 6x CH_{Cy}) 27.9-28.1 (m, 2x CH_{Cy}), 42.3 (d, $^1J_{CP}$ = 56.2 Hz, $CH_{Cy\text{-}1}$), 122.9 ($CH_{arom.\ ortho}$), 125.5 ($CH_{arom.}$), 137.8 ($CH_{arom.}$), 148.6 (N*C*H), 165.3 ($C_{arom.\ ipso}$) ppm. **^{31}P{^{1}H} NMR** (162.1 MHz, ACN-d^3): δ = 57.7 (s) ppm. **Anal. Calcd.** for $C_{23}H_{30}KN_2O_4PS_3$: C 48.91, H 5.35, N 4.96, S 17.03, found C 48.85, H 5.58, N 5.43, S 16.67.

1.12. Preparation of gold complex [(3b-Cy)AuCl)] and [(4b-Cy)·Au(PPh$_3$)]

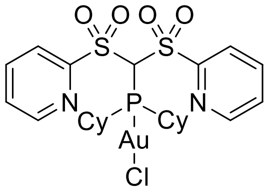

60.0 mg of **3b-Cy** (121.0 µmol) and 39.0 mg of tetrahydrothiophene gold(I)chloride (121.0 µmol) were dissolved in 5 mL THF. The solution was stirred for one hour and the solvent was removed under reduced pressure. Washing with diethyl ether gave the product [(**3b-Cy**)AuCl] as colorless solid (69.8 mg, 96 µmol, 80 %). Single crystals could be obtained by diffusing hexane into a THF solution of [(**3b-Cy**)AuCl].

^{1}H NMR (400.1 MHz, THF-d8): δ = 1.22-1.37 (m, 2H, C*H*2, Cy), 1.40-1.61 (m, 8H, C*H*2 Cy), 1.66-1.75 (m, 2H, C*H*2 Cy), 1.82-1.94 (m, 4H, C*H*2 Cy), 2.10-2.38 (m, 2H, C*H*2 Cy), 2.40-2.57 (m, 2H, C*H*2 Cy), 3.03-3.19 (m, 2H, C*H*2 Cy), 7.60-7.70 (m, 2H, C*H*arom. meta), 7.94 (d, 2*J*PH = 5.4 Hz, 1H, SC*H*S), 8.00-8.14 (m, 4H, C*H*arom.), 8.69 (d, 3*J*HH = 4.7 Hz, 2H, NC*H*) ppm. **^{13}C{^{1}H} NMR** (101.7 MHz, THF-d8): δ = 26.5 (*C*H2, Cy-4), 27.6 (*C*H2, Cy-3/3'), 27.1-27.4 (m, *C*H2, Cy-2/2'), 28.6-27.7 (m, *C*H2, Cy-2/2'), 35.6 (d, 1*J*PC = 28.4 Hz, *C*HCy-1), 82.4 (S*C*HS), 123.4 (*C*Harom. para), 129.4 (*C*Harom. meta), 140.4 (*C*Harom. ortho), 150.7 (N*C*H), 158.6 (*C*ipso) ppm. **^{31}P{^{1}H} NMR** (162.1 MHz, THF-d8): δ = 48.1 ppm. **Anal. Calcd.** for $C_{23}H_{31}AuClN_2O_4PS_2$: C 38.00, H 4.30, N 3.85, S 8.84, found C 37.77, H 4.32, N 3.92, S 8.78.

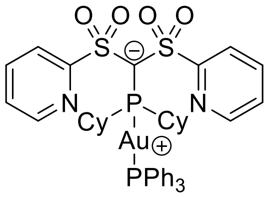

100 mg (187 µmol) **4b-Cy** and 88.0 mg (187 µmol) triphenylposphine-gold(I)chloride were dissolved in 4 ml THF and was stirred for twenty hours. The solution was filtered, and the solvent removed under reduced pressure. The crude mixture was washed three times with 3 ml diethyl ether and dried *in vacuo* giving the product as a colorless solid (59.4 mg, 62.4 µmol, 30 %; non-optimized yield). Single crystals were obtained by diffusing pentane into a THF solution.

^{1}H NMR (400 MHz, THF-d^8): δ = 1.06-1.22 (m, 9H; C$H_{2,\,Cy}$), 1.50-1.60 (m, 5H; C$H_{2,\,Cy}$), 1.64-1.72 (m, 2H; C$H_{2,\,Cy}$), 1.92-2.07 (m, 4H; C$H_{2,\,Cy}$), 2.75-2.88 (m, 2H; C$H_{,\,Cy,\,ipso}$), 6.89-7.25 (br, 2H; C$H_{arom.}$), 7.32-7.45 (m, 10H; C$H_{arom.}$), 7.57-7.71 (m, 8H; C$H_{arom.}$), 7.99 (d, $^3J_{HH}$= 7.9 Hz, 2H; NC*H*), 8.31-8.53 (br, 1H; C$H_{arom.}$). **^{31}P{^{1}H} NMR** (162 MHz, THF-d^8): δ = 42.7 (d, $^2J_{PP}$= 294.4 Hz; *P*Ph$_3$), 50.2 (d, $^2J_{PP}$ = 294.4 Hz; *P*Cy$_2$) ppm. **Anal. Calcd.** for $C_{41}H_{46}AuN_2O_4P_2S_2$: C 51.68, H 4.76, N 2.94, S 6.73, found C 51.36, H 4.67, N 3.14, S 6.46.

1.13. Procedure for the determination of the ylide/phosphine equilibria

10 mg of **3a-Cy$_2$** or **3a-*i*Pr$_2$** were dissolved in 0.5 mL of the corresponding solvent. After 24 hours ^{1}H-NMR spectra were recorded, and the ratio was determined by integration of the peaks for the proton of the ylidic and phosphine with respect to each other. This protocol was repeated several times to determine an average value for the systems. The following results were obtained:

Table S1. Ratios between the ylide and phosphine tautomers for **3a-Cy** and **3a-iPr** in different solvents.

	**3a-Cy**	**3a-iPr**
THF	17:83	20:80
Benzene	17:83	20:80
DCM	4:96	4:96
ACN	4:96	3:97

1.14 Procedure for the preparation of Phosphineselenides

Procedure for determining the regression of the ^{1}J(P-Se) copling constant with the TEP value
5 mg grey selenium (0.063 mmol) and an equimolar amount of the respective phosphine were suspended in C_6D_6 in a *J. Young* NMR tube and shaken for 2h. The reaction with tris(4-fluorophenyl)phosphine was heated at 50°C for 2 h. Afterwards, ^{31}P{^{1}H} NMR and ^{77}Se{^{1}H} NMR spectra were measured.

tri-tert-butylphosphine selenide:

^{31}P{^{1}H} NMR (162.1 MHz, C_6D_6): δ = 92.2 ppm.

^{77}Se{^{1}H} NMR (76 MHz, C_6D_6) δ = -423.5 (d, $^1J_{PSe}$ = 711.32

tricyclohexylphosphine selenide:

^{31}P{^{1}H} NMR (162.1 MHz, C_6D_6): δ = 57.8 ppm.

^{77}Se{^{1}H} NMR (76 MHz, C_6D_6) δ = -469.9 (d, $^1J_{PSe}$ = 707.67 Hz).

triisopropylphosphine selenide:

^{31}P{^{1}H} NMR (162.1 MHz, C_6D_6): δ = 67.2 ppm.

$^{77}Se\{^1H\}$ NMR (76 MHz, C_6D_6) δ = -492.8 (d, $^1J_{PSe}$ = 716.19 Hz).

methyldiphenylphosphine selenide:

$^{31}P\{^1H\}$ NMR (162.1 MHz, C_6D_6): δ = 22.6 ppm.

$^{77}Se\{^1H\}$ NMR (76 MHz, C_6D_6) δ = -292.9 (d, $^1J_{PSe}$ = 747.76 Hz).

benzyldiphenylphosphine selenide:

$^{31}P\{^1H\}$ NMR (162.1 MHz, C_6D_6): δ = 34.2 ppm.

$^{77}Se\{^1H\}$ NMR (76 MHz, C_6D_6) δ = -332.8 (d, $^1J_{PSe}$ = 762.59 Hz).

triphenylphosphine selenide:

$^{31}P\{^1H\}$ NMR (162.1 MHz, C_6D_6): δ = 35.4 ppm.

$^{77}Se\{^1H\}$ NMR (76 MHz, C_6D_6) δ = -278.2 (d, $^1J_{PSe}$ = 756.80 Hz).

tris(4-fluorophenyl)phosphine selenide:

$^{31}P\{^1H\}$ NMR (162.1 MHz, C_6D_6): δ = 27.9 ppm.

$^{77}Se\{^1H\}$ NMR (76 MHz, C_6D_6) δ = -260.7 (d, $^1J_{PSe}$ = 765.04 Hz).

Correlation between the $^1J_{PSe}$ coupling constant and TEP value

Table S2. Literature TEP values and measured $^1J_{PSe}$ coupling constants in C_6D_6.

Phosphine	TEP [cm^{-1}]	$^1J_{PSe}$ [Hz]
$P(tBu)_3$	2056.1	711.33
PCy_3	2056.4	707.91
$P(iPr)_3$	2059.2	715.54
PPh_2Me	2067	747.76
$PPh_2(CH_2Ph)$	2068.4	762.59
PPh_3	2068.9	756.8
$P(pC_6H_4F)_3$	2071.3	765

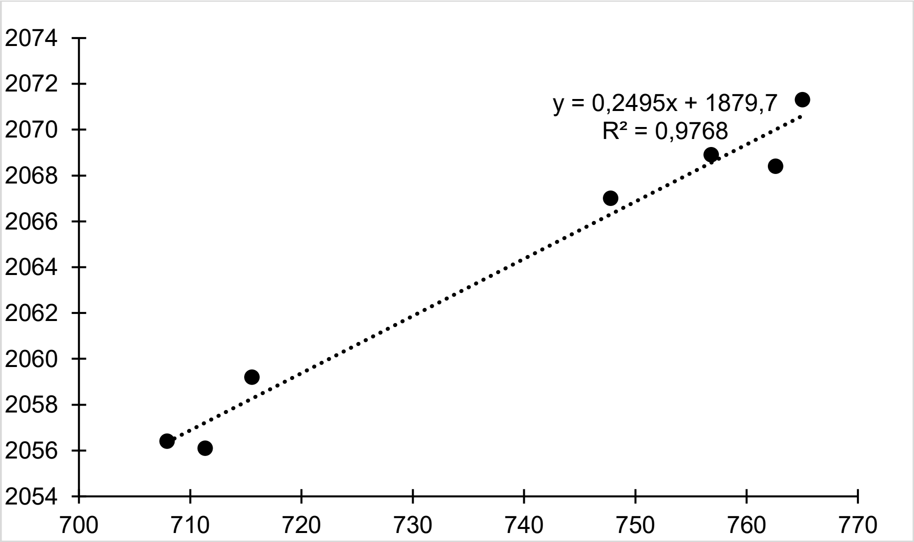

Figure S1. Correlation of Literature TEP values and measured $^1J_{PSe}$ coupling constants in C_6D_6.

Calculation of TEP from the relationship between vCO for $Ni(CO)_3(L)$ and $^1J_{SeJ}$ constant in selenides:

Reaction of 3a-Cy and 3b-Cy with Se

19.2 µmol of neutral ligands (**3a-Cy** and **3b-Cy**) and 21.1 µmol of selenium were weighed inside a J. Young NMR tube and C_6D_6 was added. The tube was heated at 70 °C temperature for 24 h. NMR was taken directly from the reaction mixture.

For **3a-Cy**: **^{31}P NMR** (162 MHz, C_6D_6): δ 65.10 (d, $^1J_{PSe}$ = 762.0 Hz). **^{77}Se NMR** (76 MHz, C_6D_6): -349.43 (d, $^1J_{PSe}$ = 762.0 Hz). (75 % conversion)

For **3b-Cy**: **^{31}P NMR** (162 MHz, C_6D_6): δ 61.98 (d, $^1J_{PSe}$ = 753.0 Hz). **^{77}Se NMR** (76 MHz, C_6D_6): δ -364.96 (d, $^1J_{PSe}$ = 753.0 Hz) (90 % conversion)

Reaction of 4a-Cy and 4b-Cy with Se

19.2 µmol of anionic ligands (**4a-Cy** and **4b-Cy**) and 21.1 µmol of selenium were weighed inside a J. Young NMR tube and THF-d8 was added. The tube was heated at 70 °C temperature for overnight. NMR was taken directly from the reaction mixture.

For **4a-Cy**: **^{31}P NMR** (162 MHz, THF-d^8): δ 47.89 (d, $^1J_{PSe}$ = 661.9 Hz). **^{77}Se NMR** (76 MHz, C_6D_6): -427.05 (d, $^1J_{PSe}$ = 661.9 Hz). (100 % conversion)

For **4b-Cy**: **^{31}P NMR** (162 MHz, THF-d^8): δ 48.31 (d, $^1J_{PSe}$ = 667.1 Hz). **^{77}Se NMR** (76 MHz, C_6D_6): -418.62 (d, $^1J_{PSe}$ = 667.1 Hz). (100 % conversion)

Table S3: TEP values of phosphines **3** and **4** calculated from their $^1J_{PSe}$ coupling constant base.

	**$^1J_{PSe}$ [Hz]**	**calc. TEP [cm^{-1}]**
**3a-Cy**	762.0	2069.82
**4a-Cy**	661.9	2044.84
**3b-Cy**	753.0	2067.57
**4b-Cy**	667.1	2046.14

1.15 Procedure for the gold-catalysed hydroamination reaction

A 2 ml glass vial with a rubber cap and a stir bar was charged in a glovebox with the indicated amount of catalyst and $NaBAr^F_4$ (if indicated). The aniline (5.25 mmol) and the phenylacetylene (5.00 mmol) were added outside the glovebox via syringe. The vial was heated in a metal block heater to the indicated temperature while stirring. Small aliquots were removed by syringe and added directly to an NMR tube to monitor reaction progress. Yields were determined by integration of the peak for the alkyne starting material with respect to the peak for the imine product in the ^{1}H NMR spectrum.

Table S3. Detailed results for the gold-catalysed hydroamination.

Catalyst/loading	1 h	3 h	5 h	24 h
PPh_3AuCl or (THT)AuCl/ 0.5 mol%	n.o.	n.o.	n.o.	n.o.
[(**4b-Cy**)·Au(PPh_3)] / 0.5 mol%	n.o.	n.o.	n.o.	n.o.
**4b-Cy + (THT)AuCl**/ 0.5 mol%	68 %	86 %	87 %	99 %
**4b-Cy + (THT)AuCl**/ 0.1mol%	25 %	49 %	63 %	74 %
[(**3b**-Cy)AuCl)] / 0.5 mol%	30 %	53 %	64 %	89 %
[(**3b**-Cy)AuCl)] / 0.1mol%	12 %	26 %	36 %	66 %
PPh_3AuCl + $NaBAr^F$/ 0.1 mol%				39 %

1 NMR Spectra

2.1 NMR Spectra of the isolated compounds

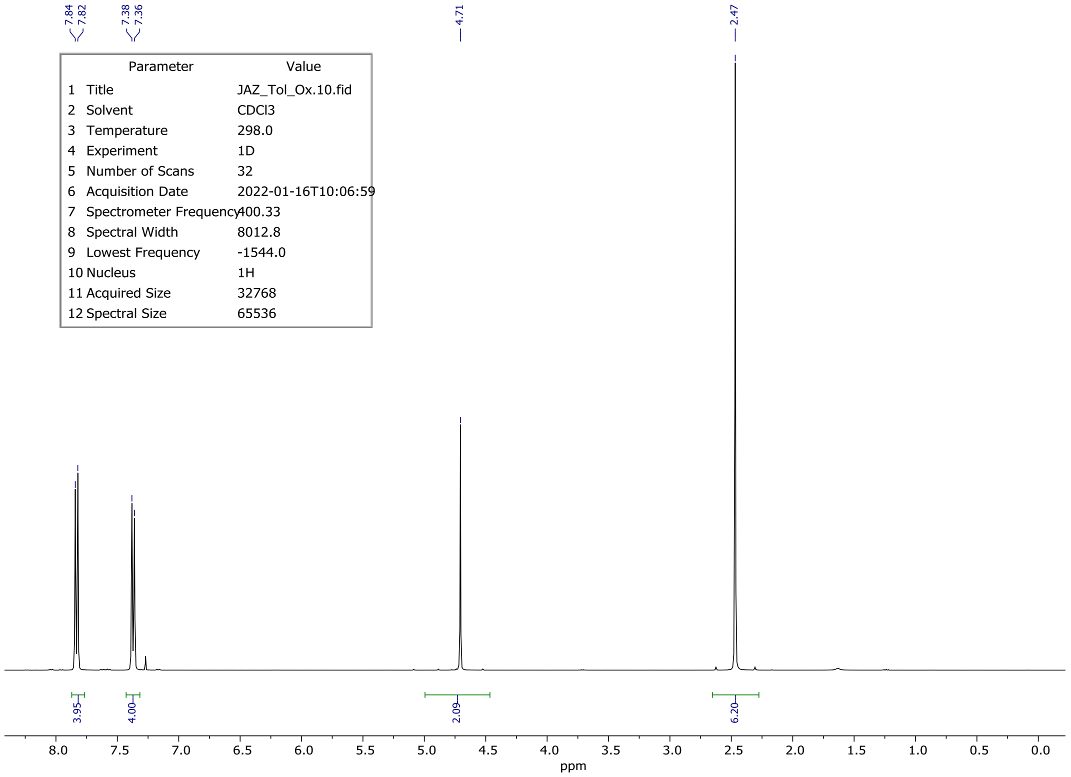

Figure S2. ^{1}H NMR spectrum of **1a** in $CDCl_3$.

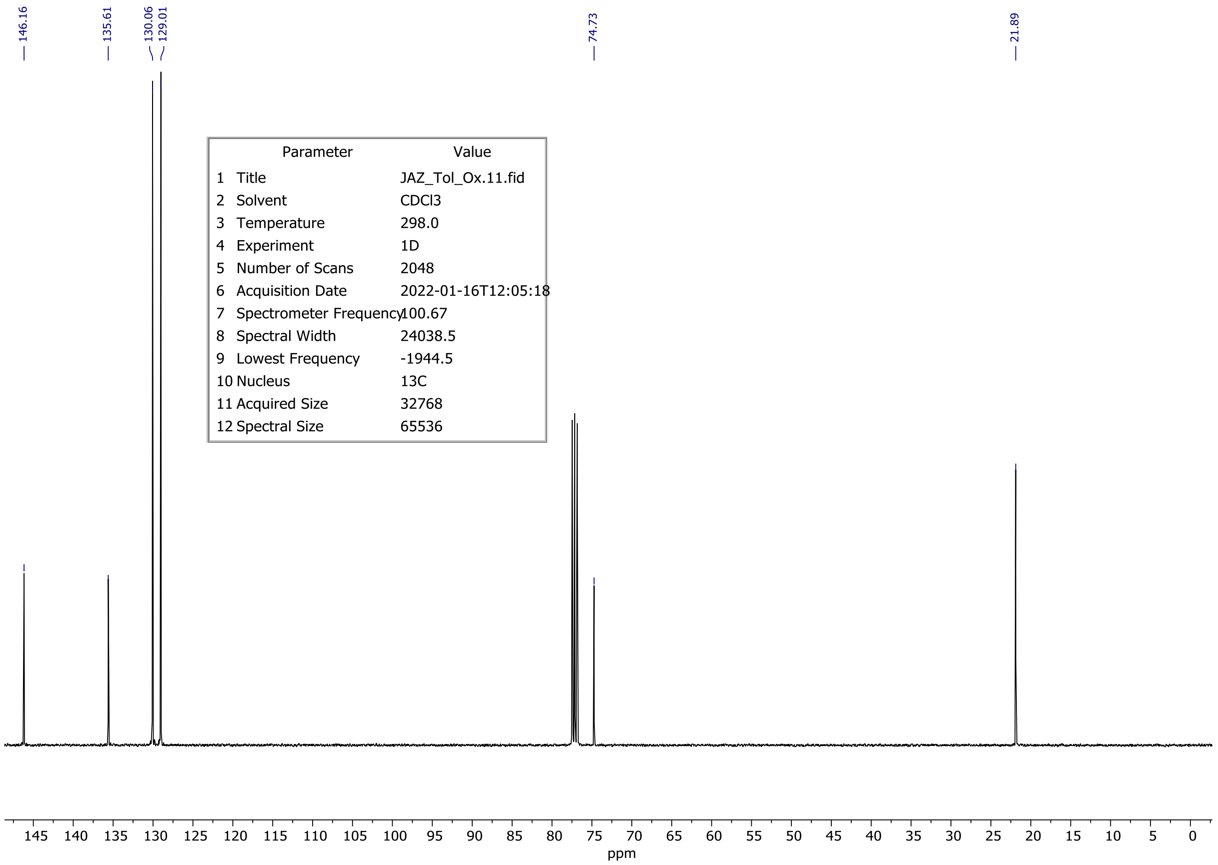

Figure S3. ^{13}C{^{1}H} NMR spectrum of **1a** in $CDCl_3$.

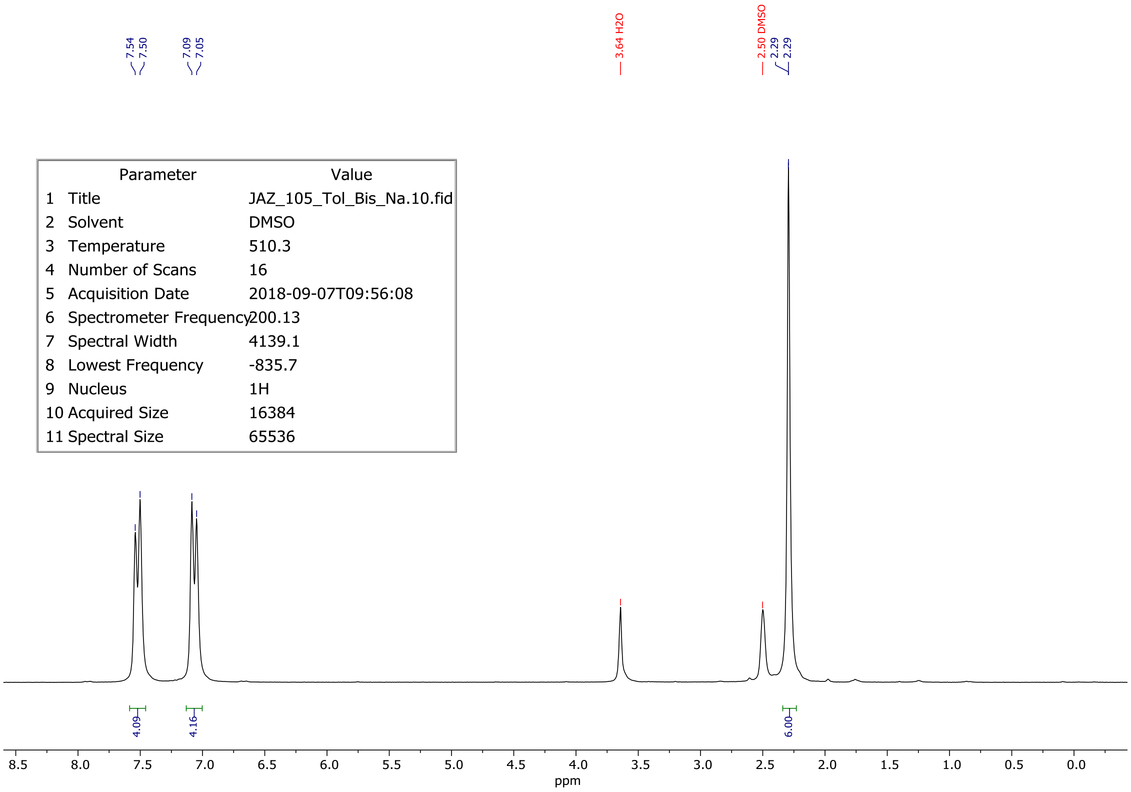

Figure S4. ^{1}H NMR spectrum of **2a** in DMSO-d^{6}.

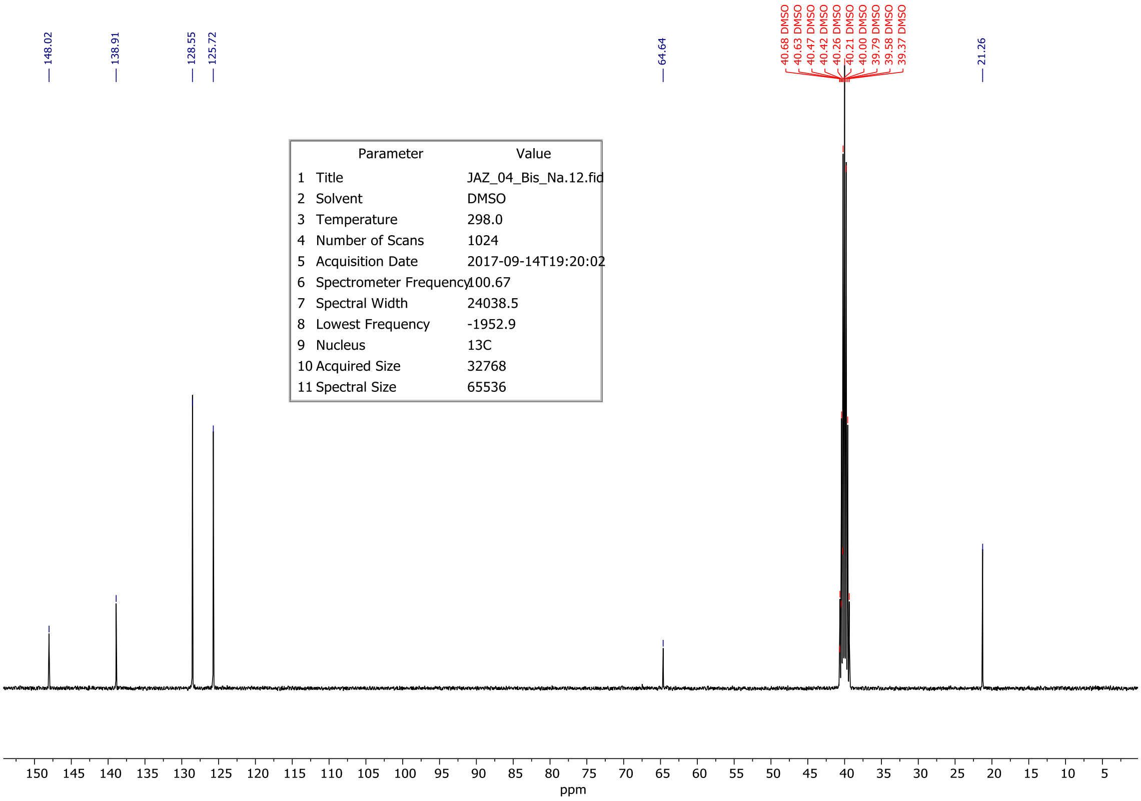

Figure S5. $^{13}C\{^{1}H\}$ NMR spectrum of **2a** in DMSO-d^{6}.

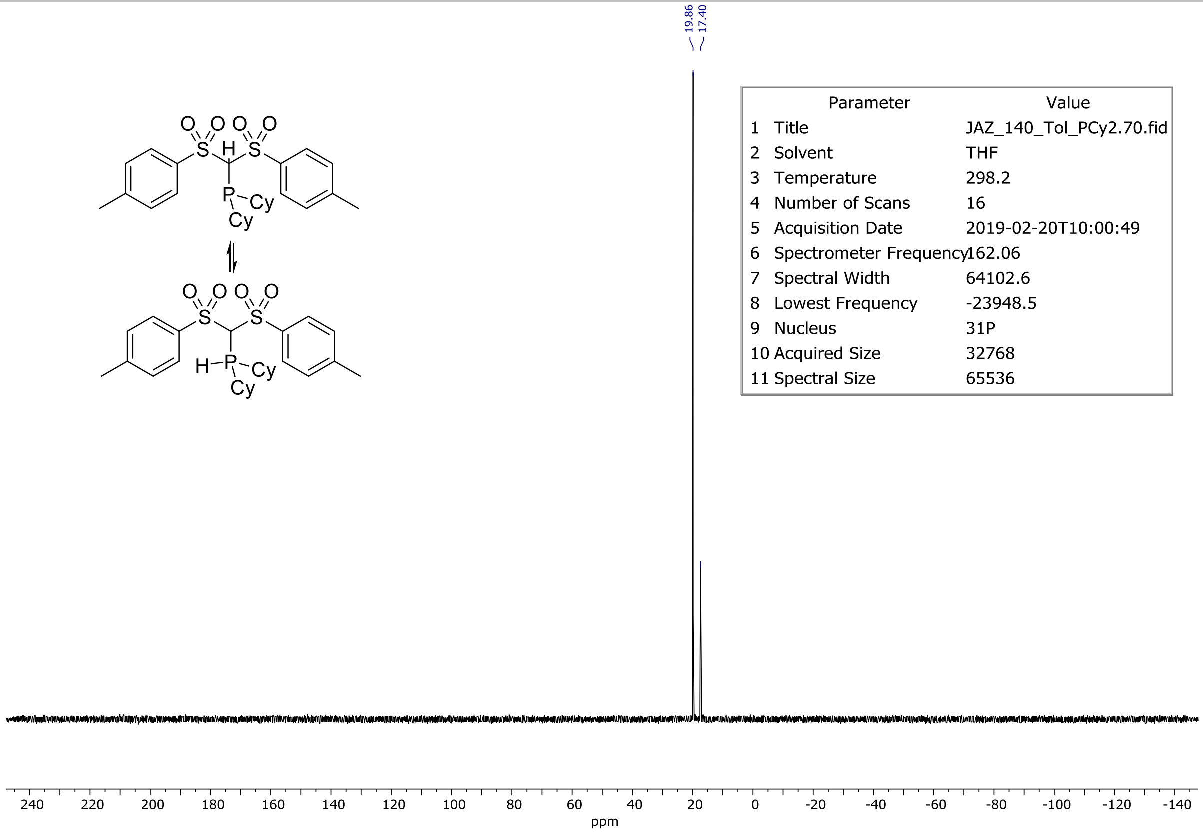

Figure S6. $^{31}P\{^{1}H\}$ NMR of **3a-Cy/ 3a'-Cy** in THF-d[8].

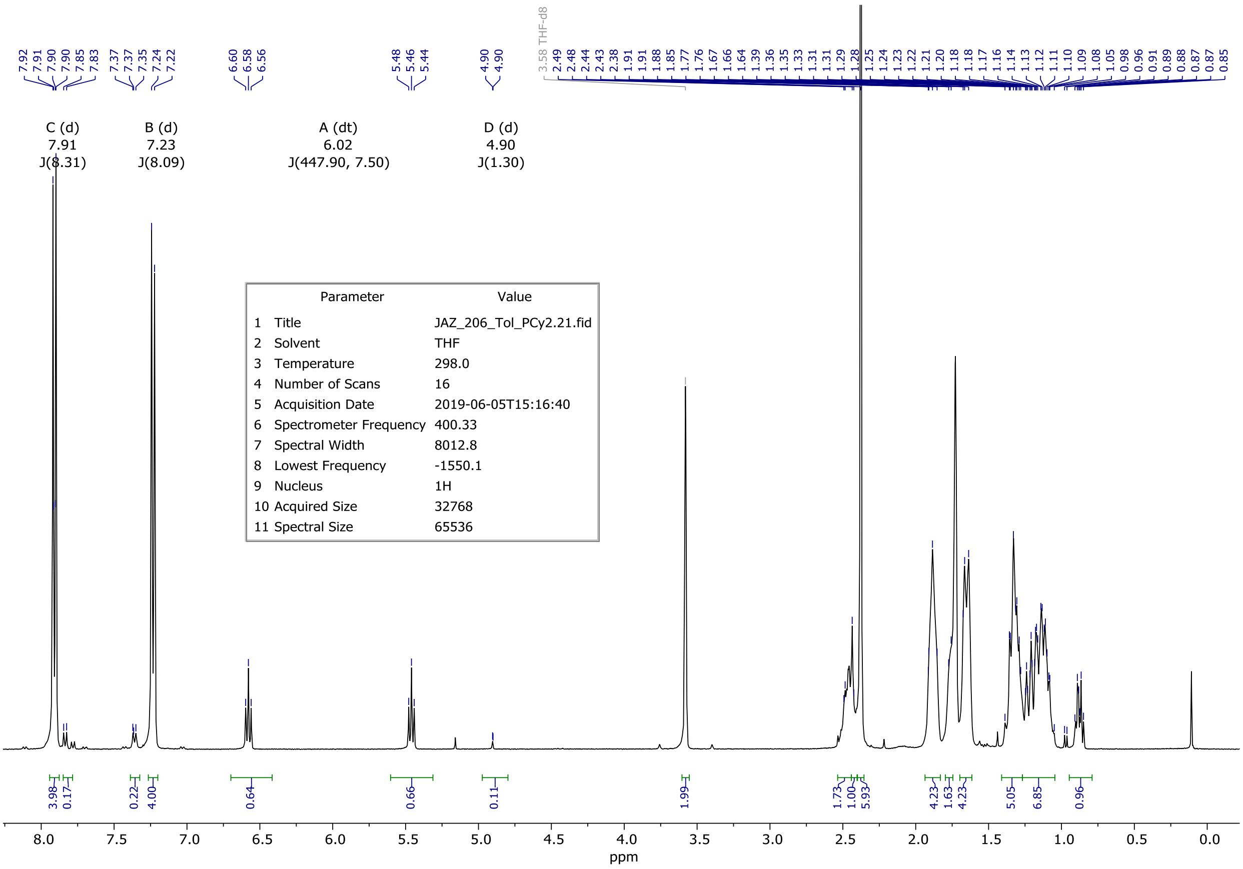

Figure S7. ^{1}H NMR of **3-Cy** / **3a'-Cy** in THF-d[8].

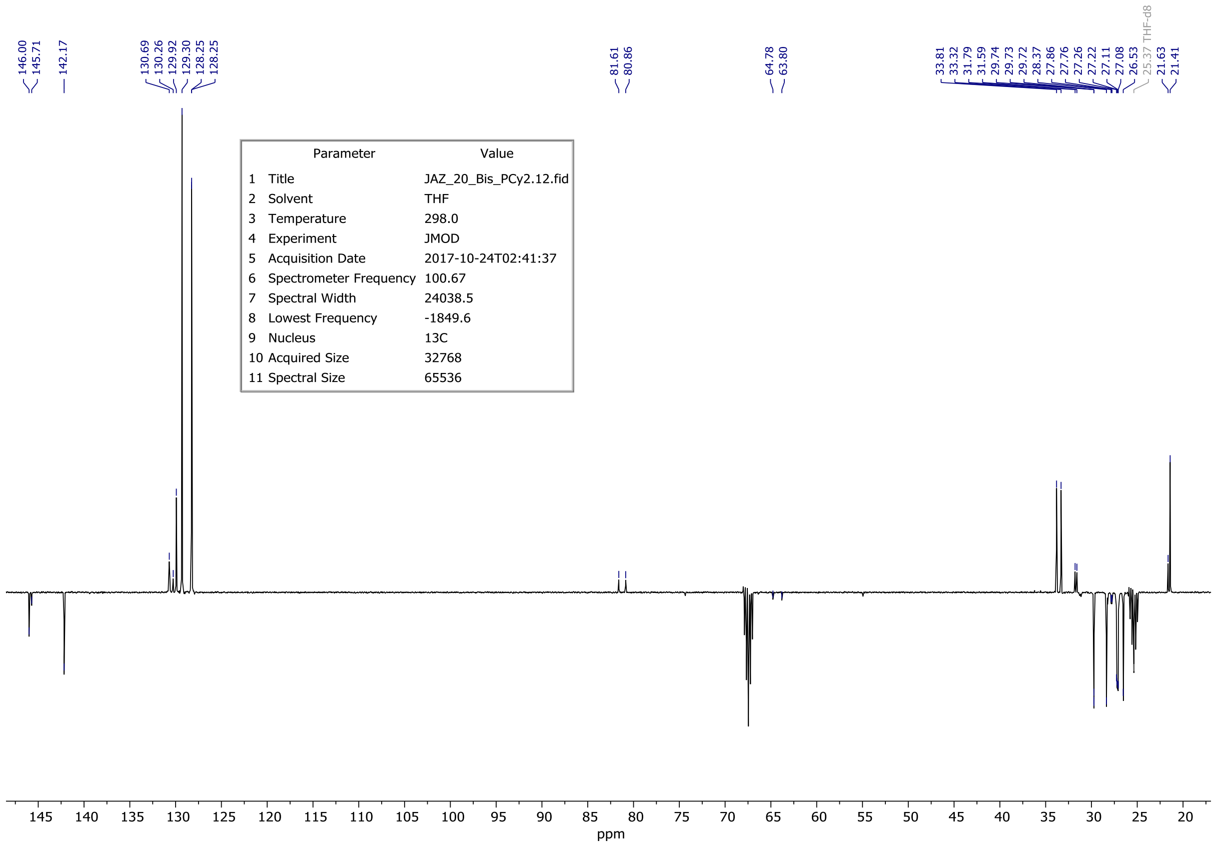

Figure S8. $^{13}C\{^1H\}$ NMR spectrum of **3a-Cy/ 3a'-Cy** in THF

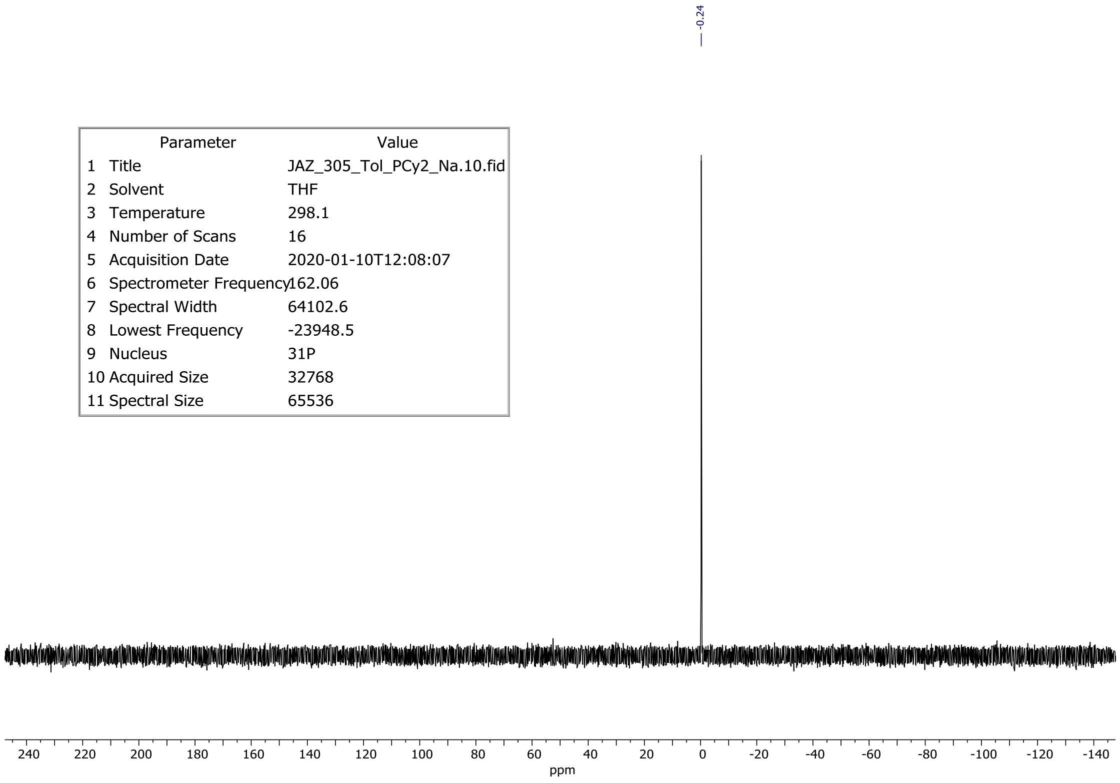

.

Figure S9. $^{31}P\{^1H\}$ NMR of **4a-PCy** in THF-d^8.

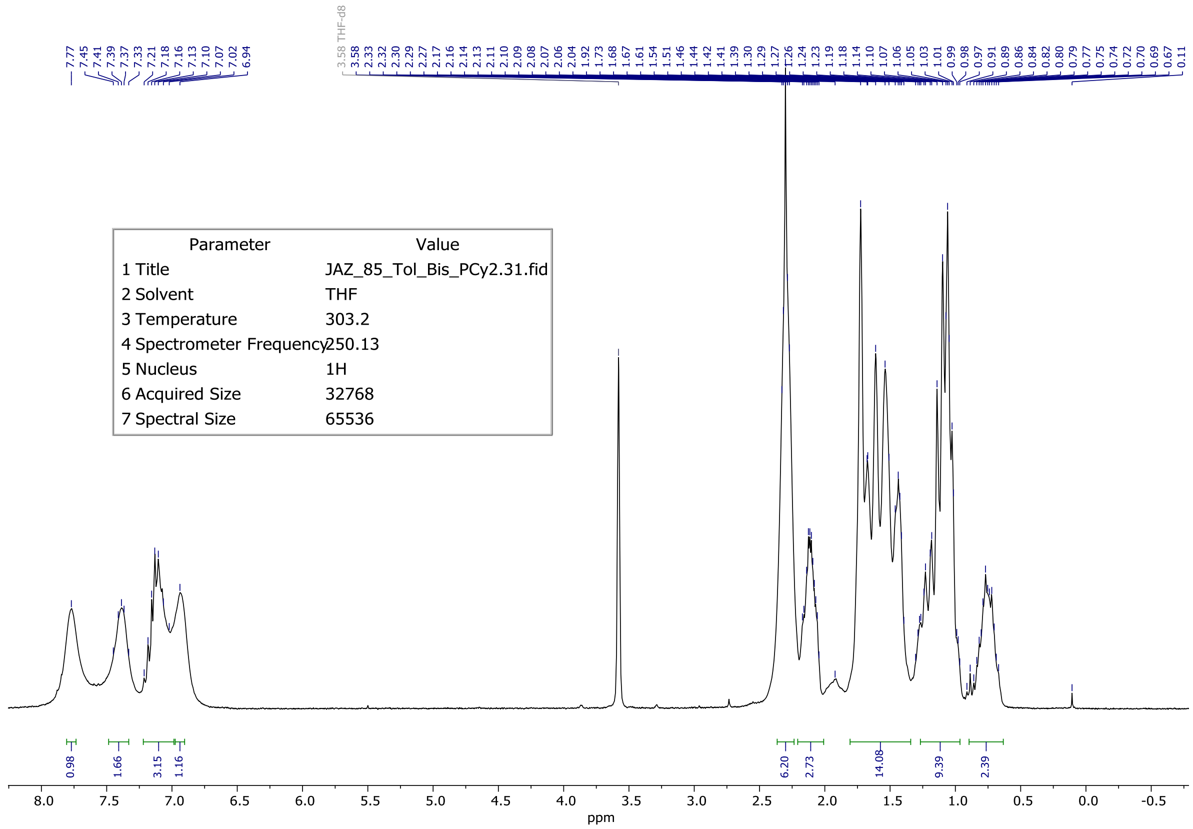

Figure S10. ^{1}H NMR of **4a-Cy** in THF-d^{8}.

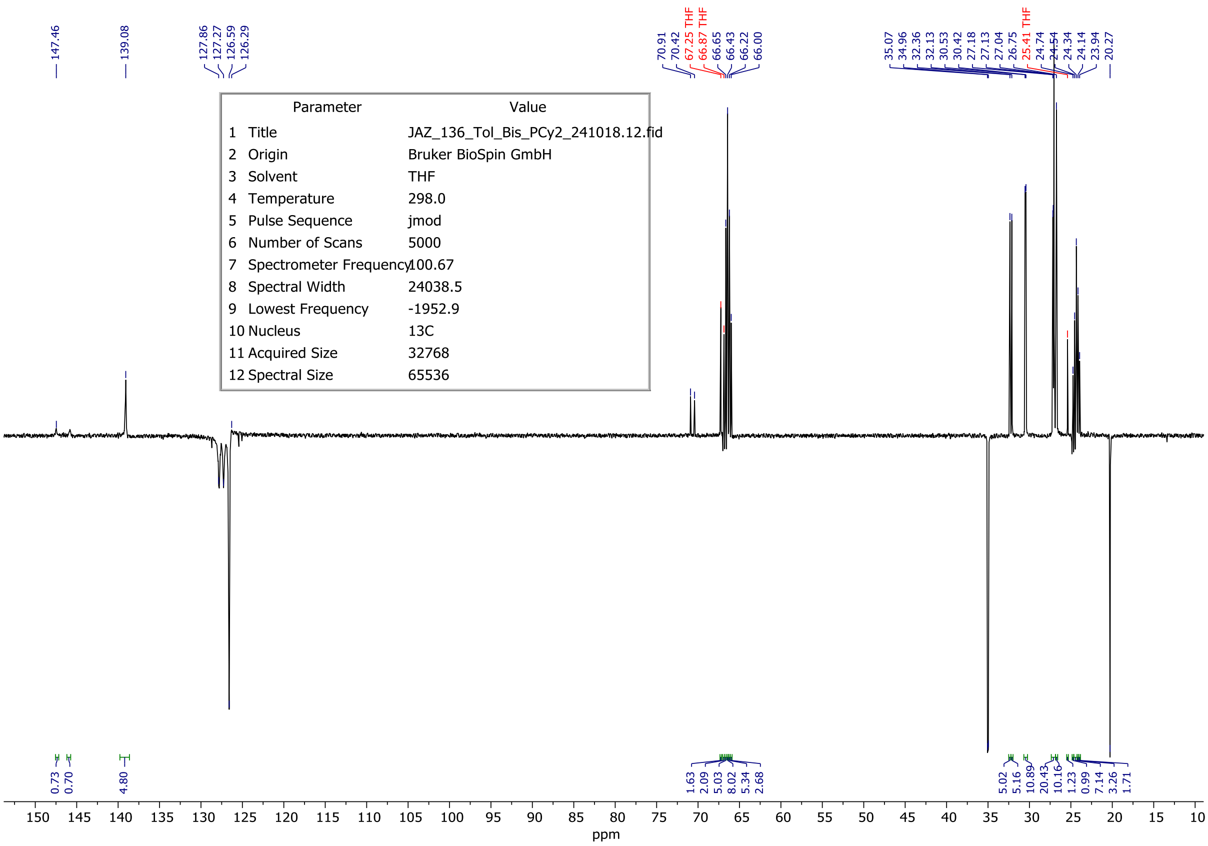

Figure S11. $^{13}C\{^{1}H\}$ NMR spectrum of **4a-Cy** in THF.

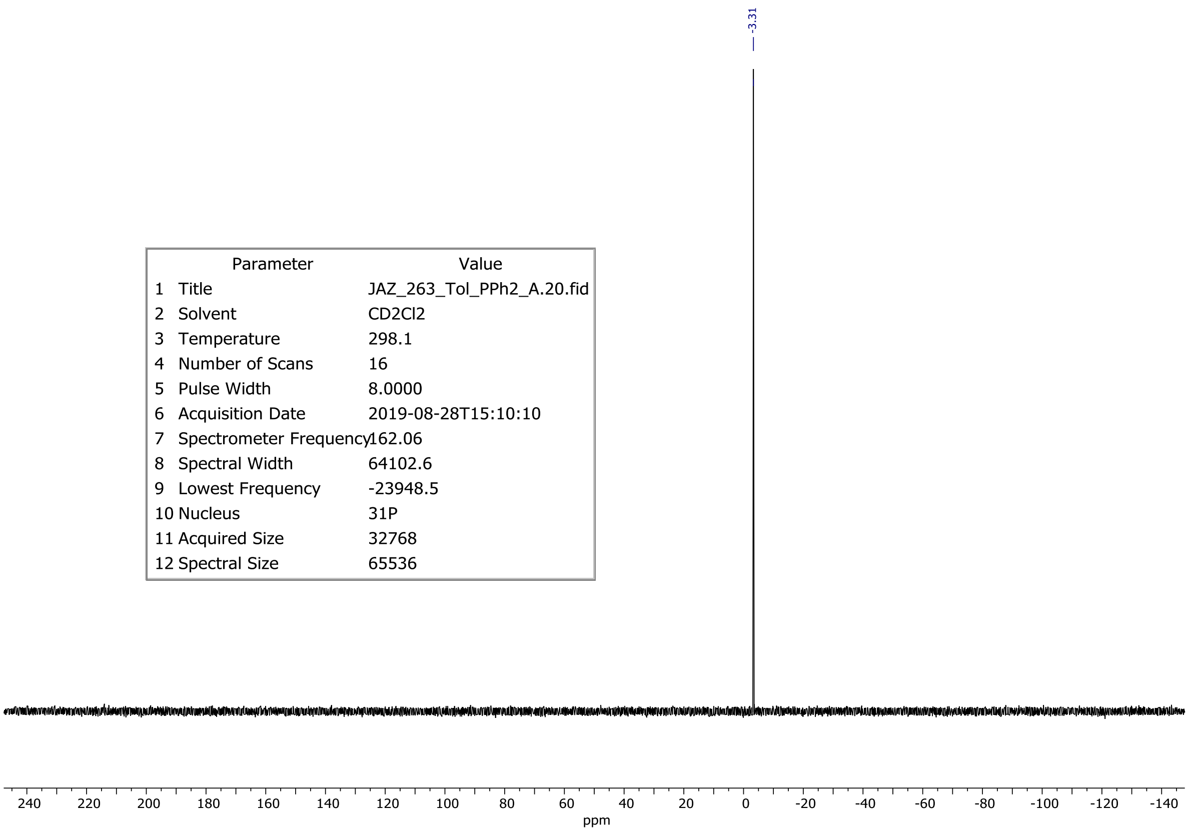

Figure S12. $^{31}P\{^1H\}$ NMR of **3a-Ph** in CD_2Cl_2.

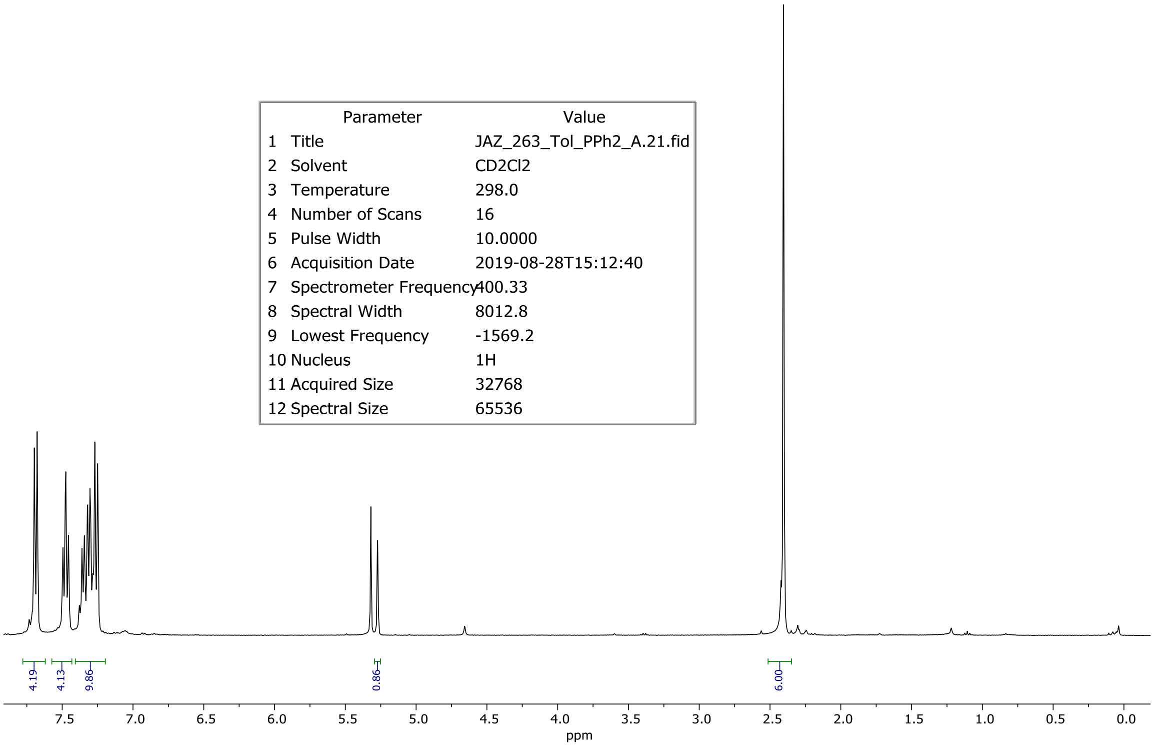

Figure S13. ^1H NMR spectrum of **3a-Ph** in CD_2Cl_2.

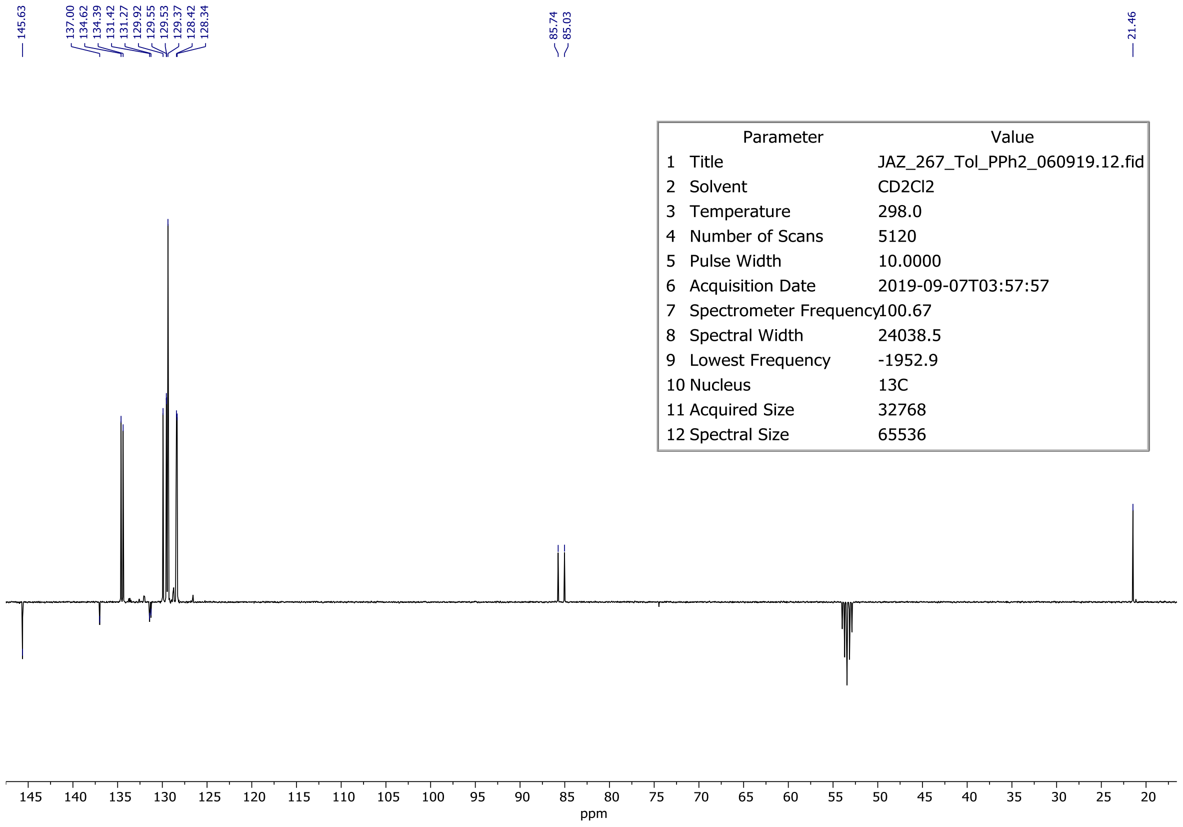

Figure S14. $^{13}C\{^{1}H\}$ NMR spectrum of **3a-Ph** in CD_2Cl_2.

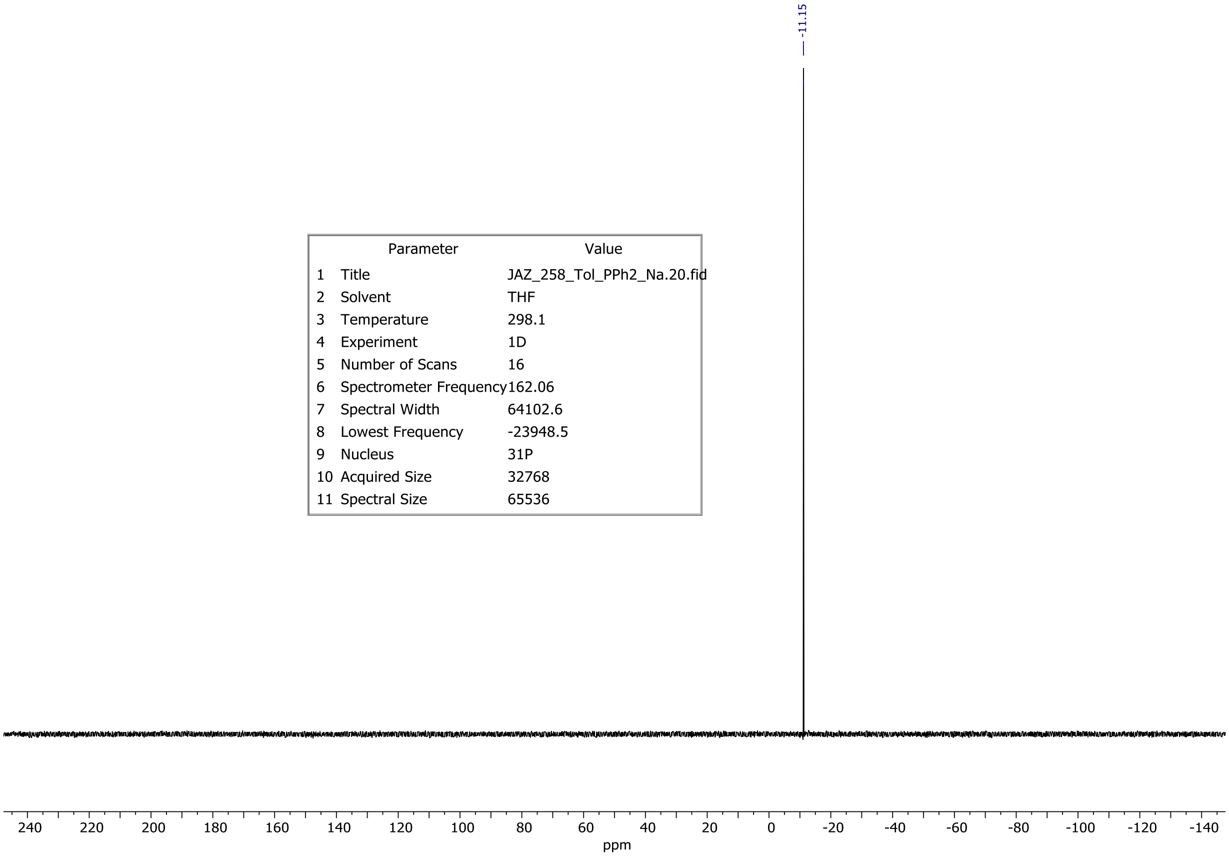

Figure S15. $^{31}P\{^{1}H\}$ NMR of **4a-Ph** in THF-d^8.

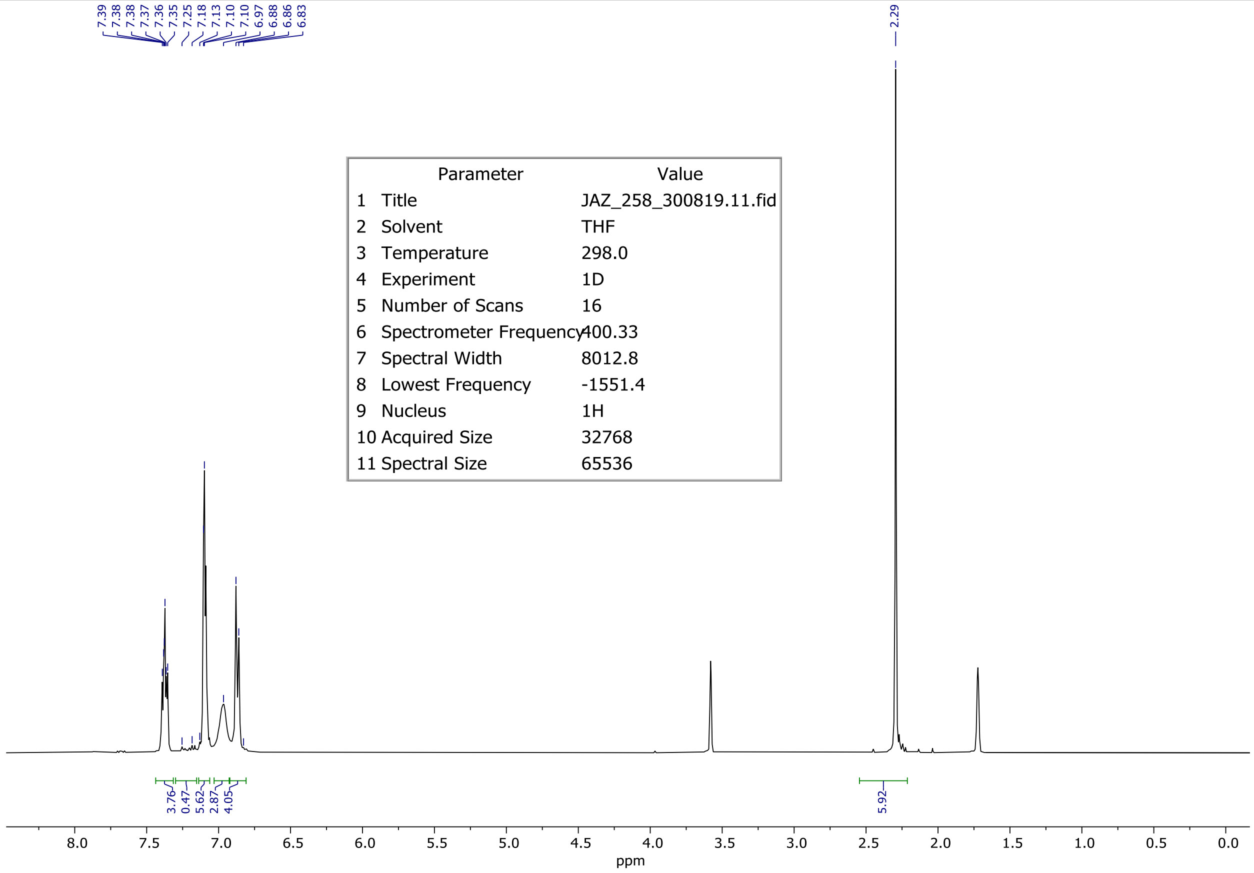

Figure S16. ^{1}H NMR spectrum of **4a-Ph** in THF-d^{8}.

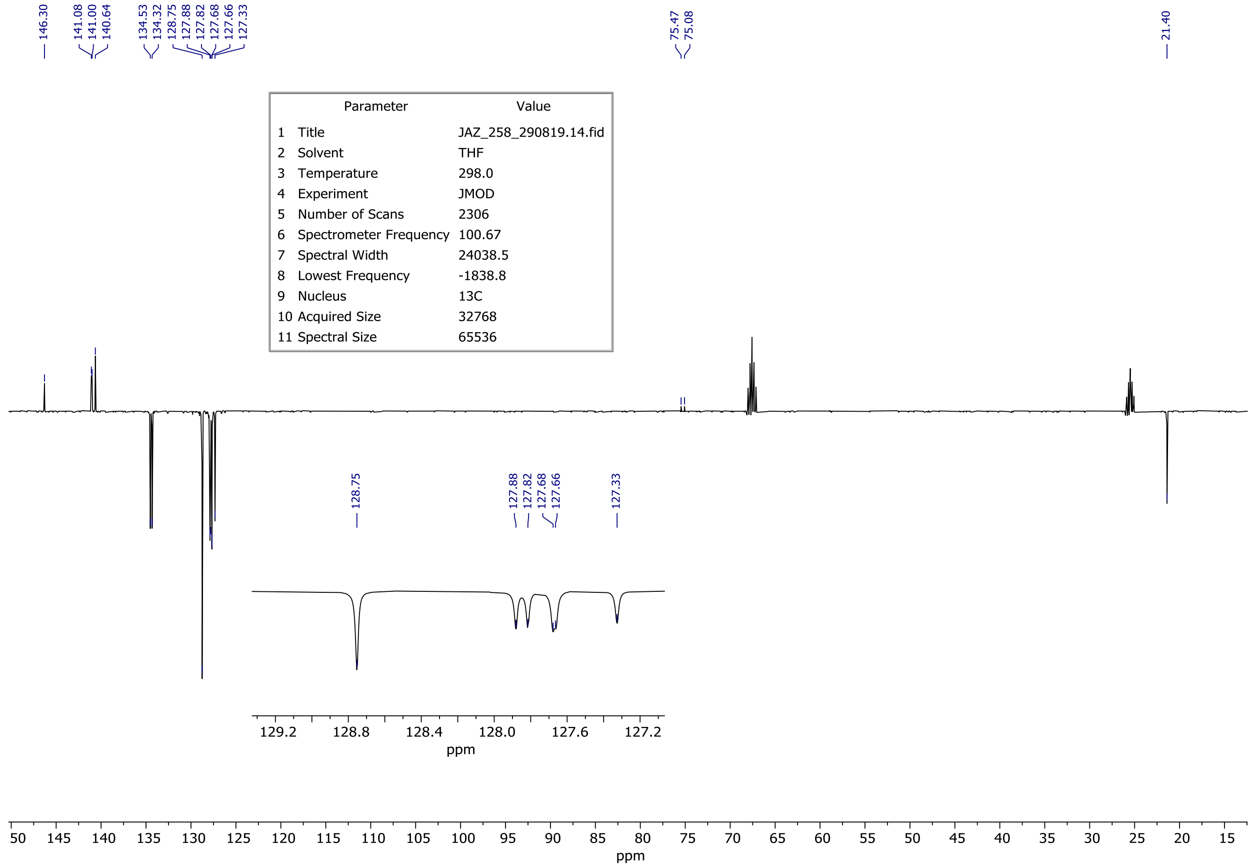

Figure S17. $^{13}C\{^{1}H\}$ NMR spectrum of **4a-Ph** in THF-d^{8}.

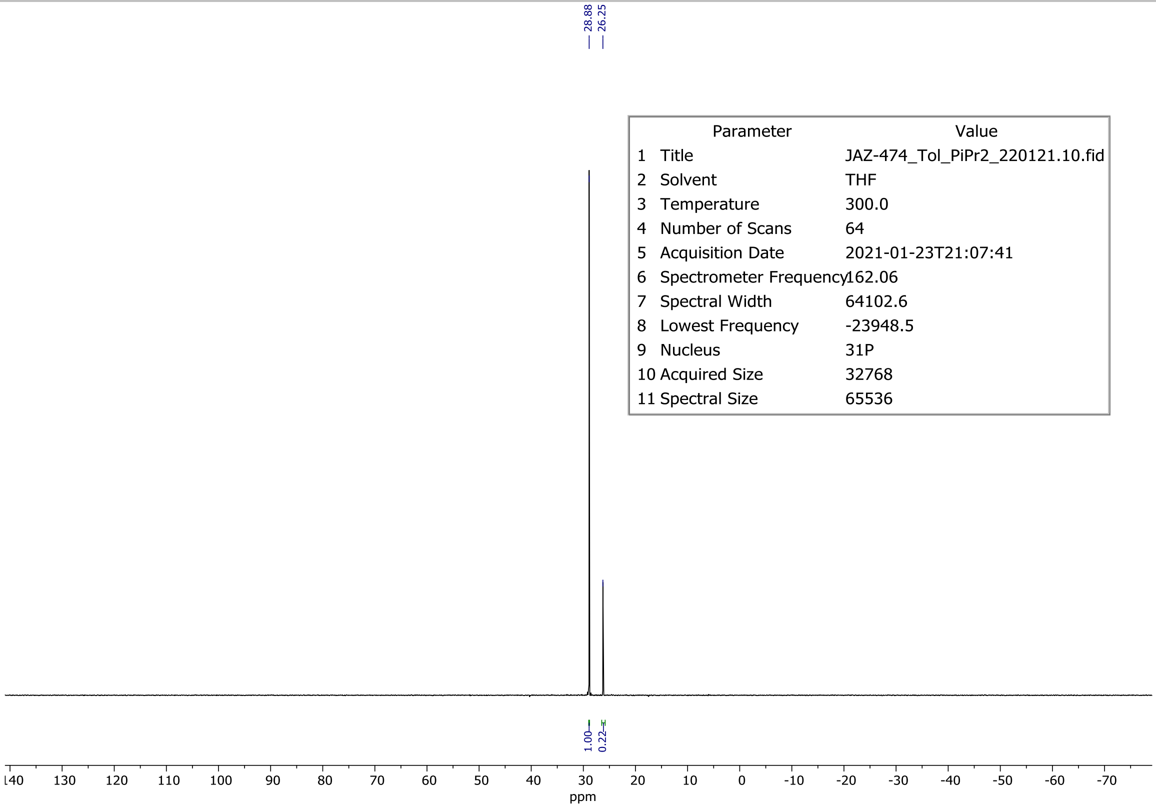

Figure S18. $^{31}P\{^1H\}$ NMR of **3a-iPr** in THF-d[8].

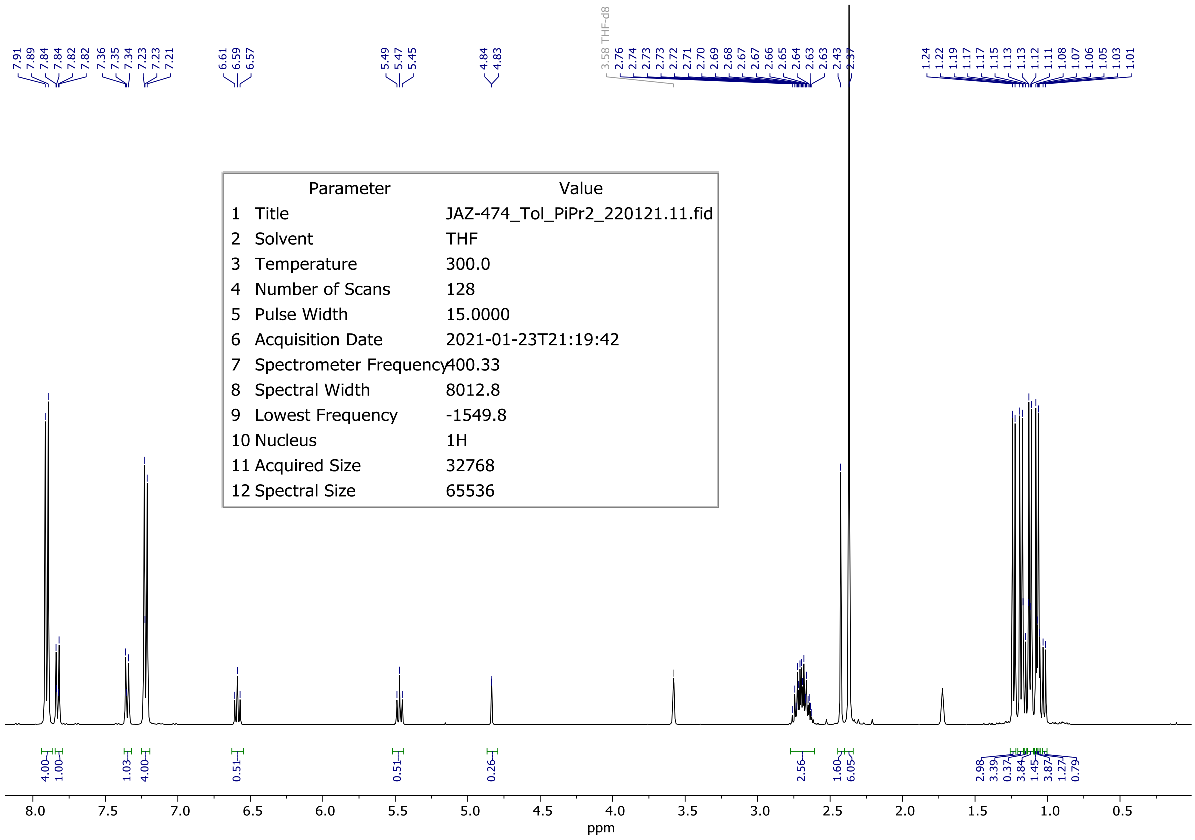

Figure S19. ^1H NMR spectrum of **3a-iPr** in THF-d[8].

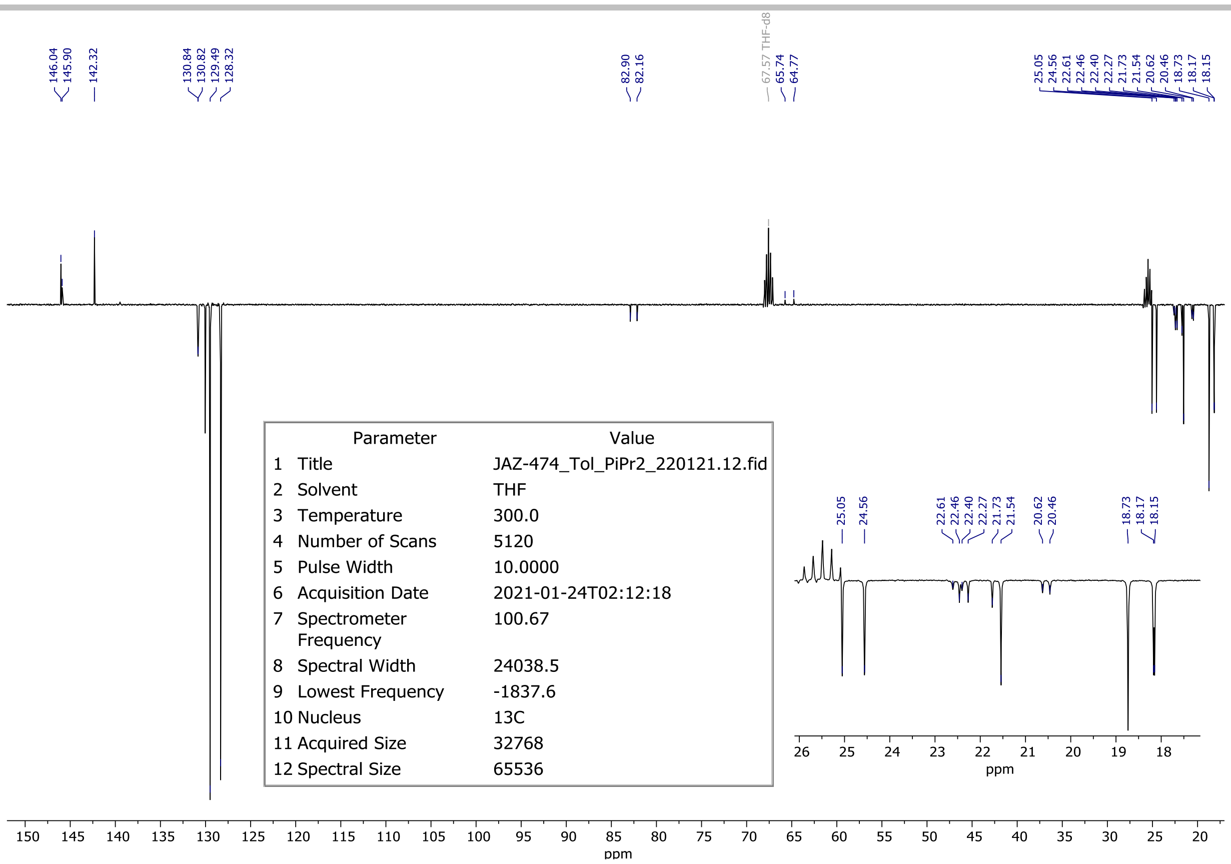

Figure S20. $^{13}C\{^1H\}$ NMR spectrum of **3a-iPr** in THF-d[8].

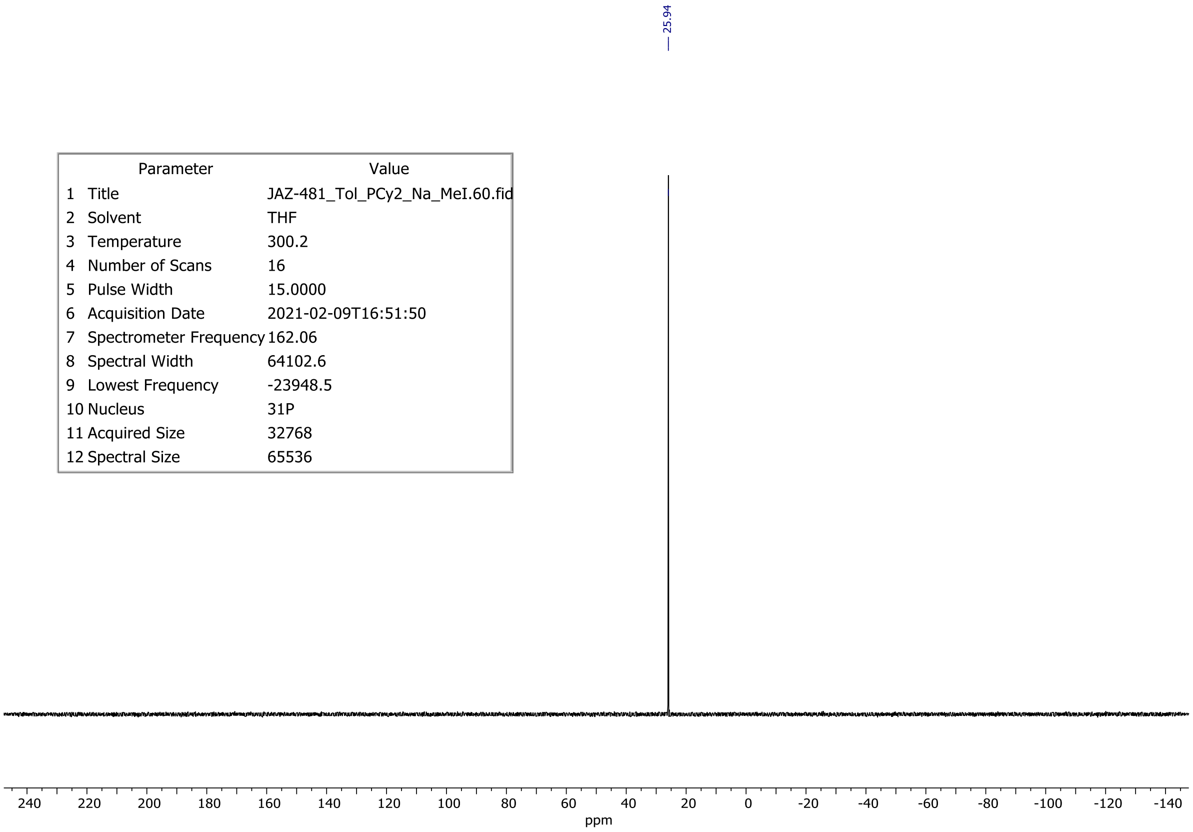

Figure S21. $^{31}P\{^1H\}$ NMR of **5a** in CD_3CN.

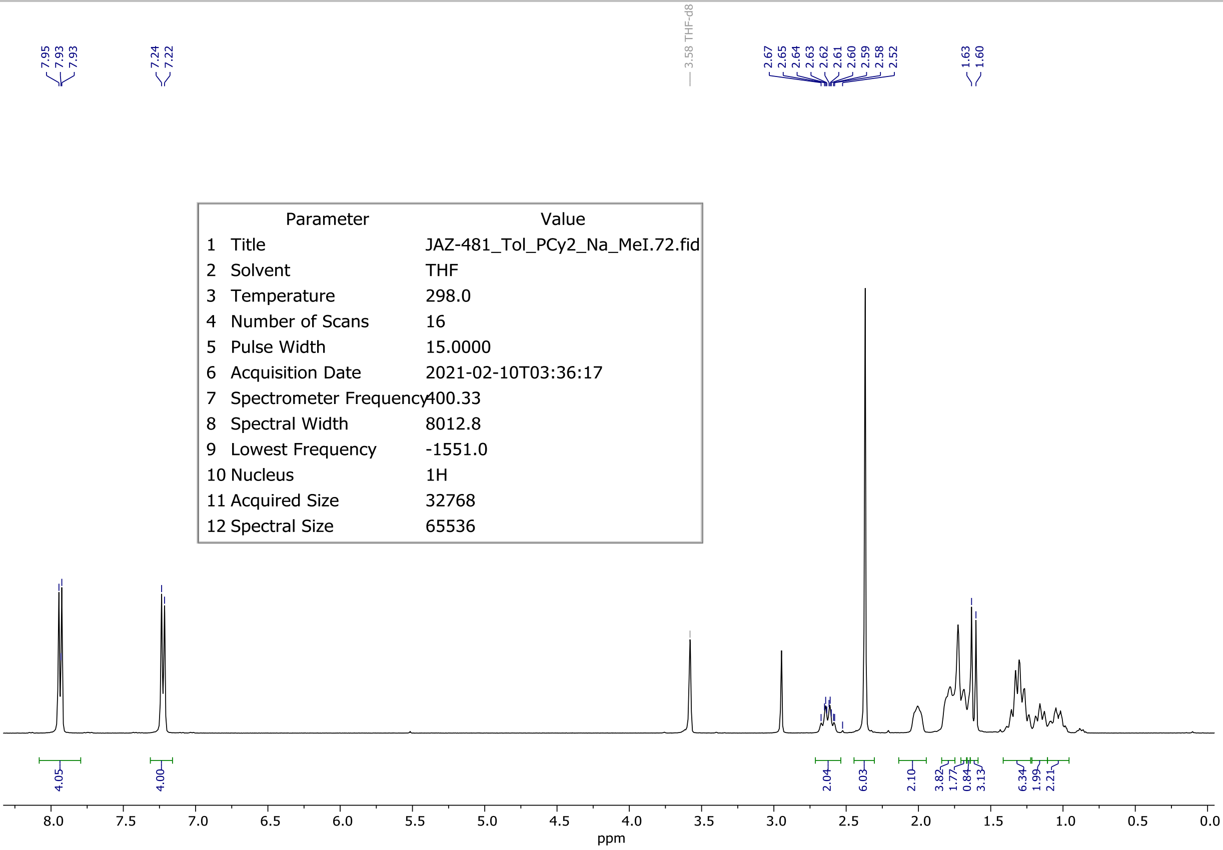

Figure S22. ^{1}H NMR spectrum of **5a** in CD_3CN.

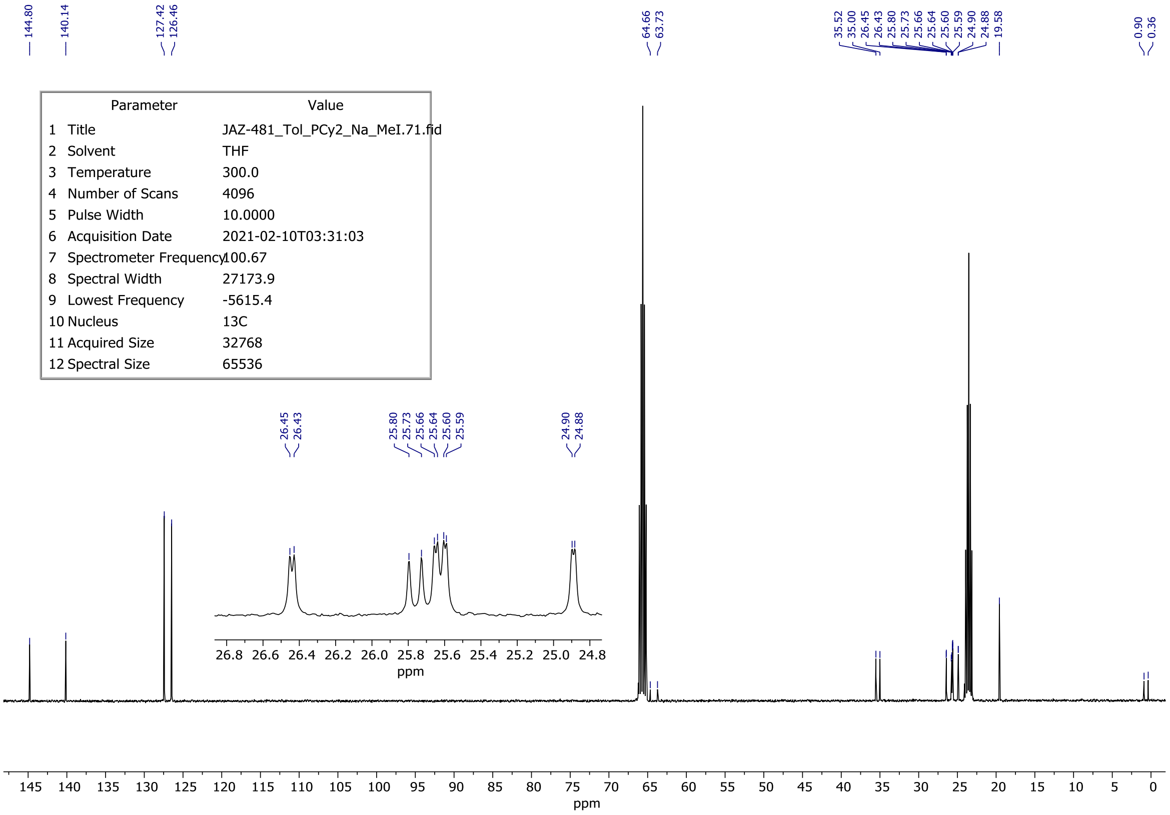

Figure S23. $^{13}C\{^{1}H\}$ NMR spectrum of **5a** in THF-d^{8}.

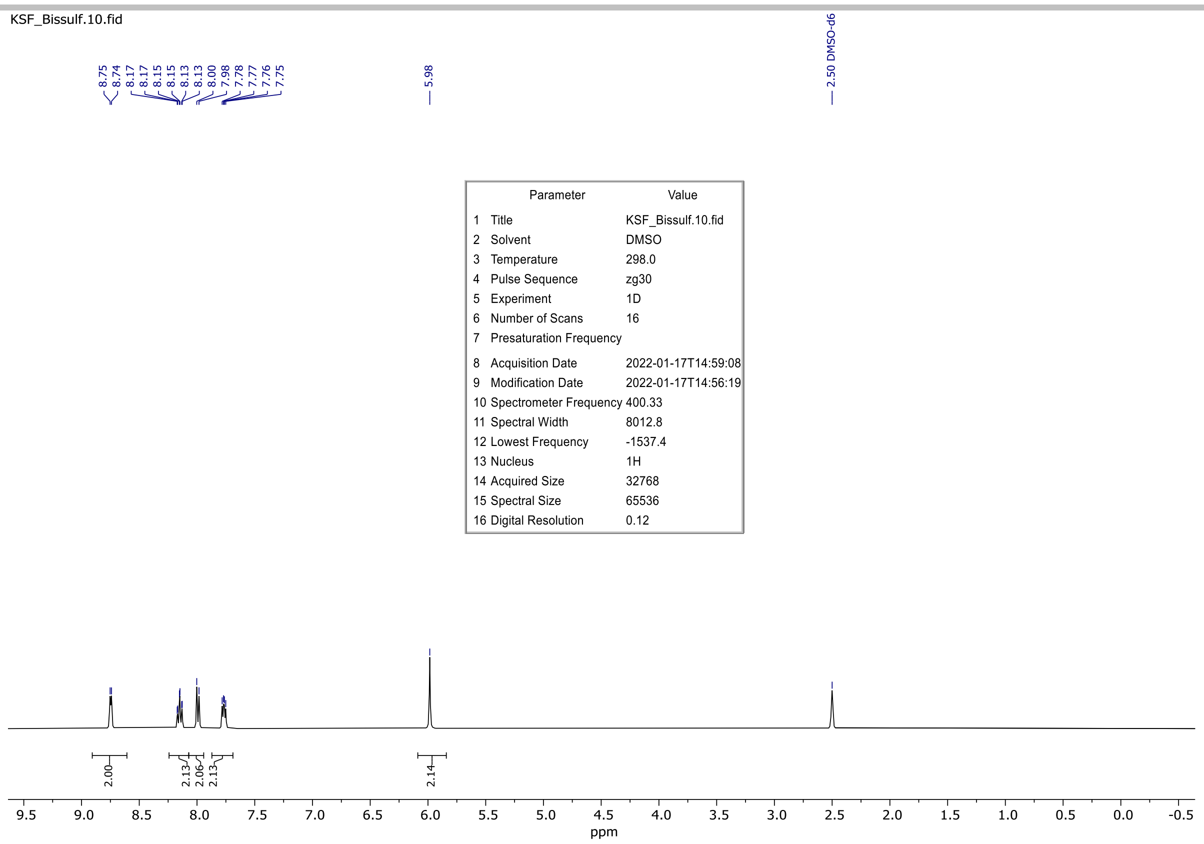

Figure S24. ^{1}H NMR of **1b** in DMSO-d^{6}.

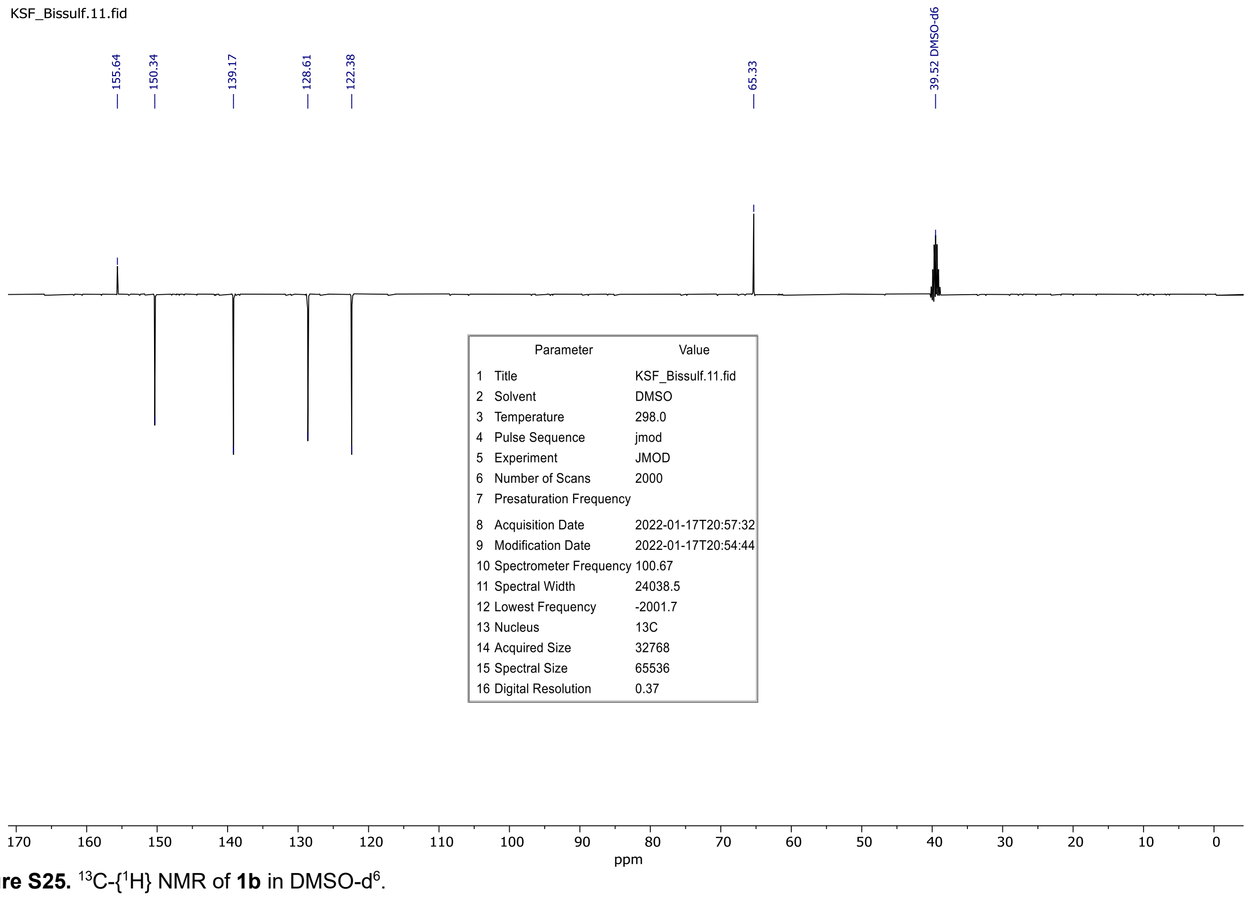

Figure S25. ^{13}C-{^{1}H} NMR of **1b** in DMSO-d^{6}.

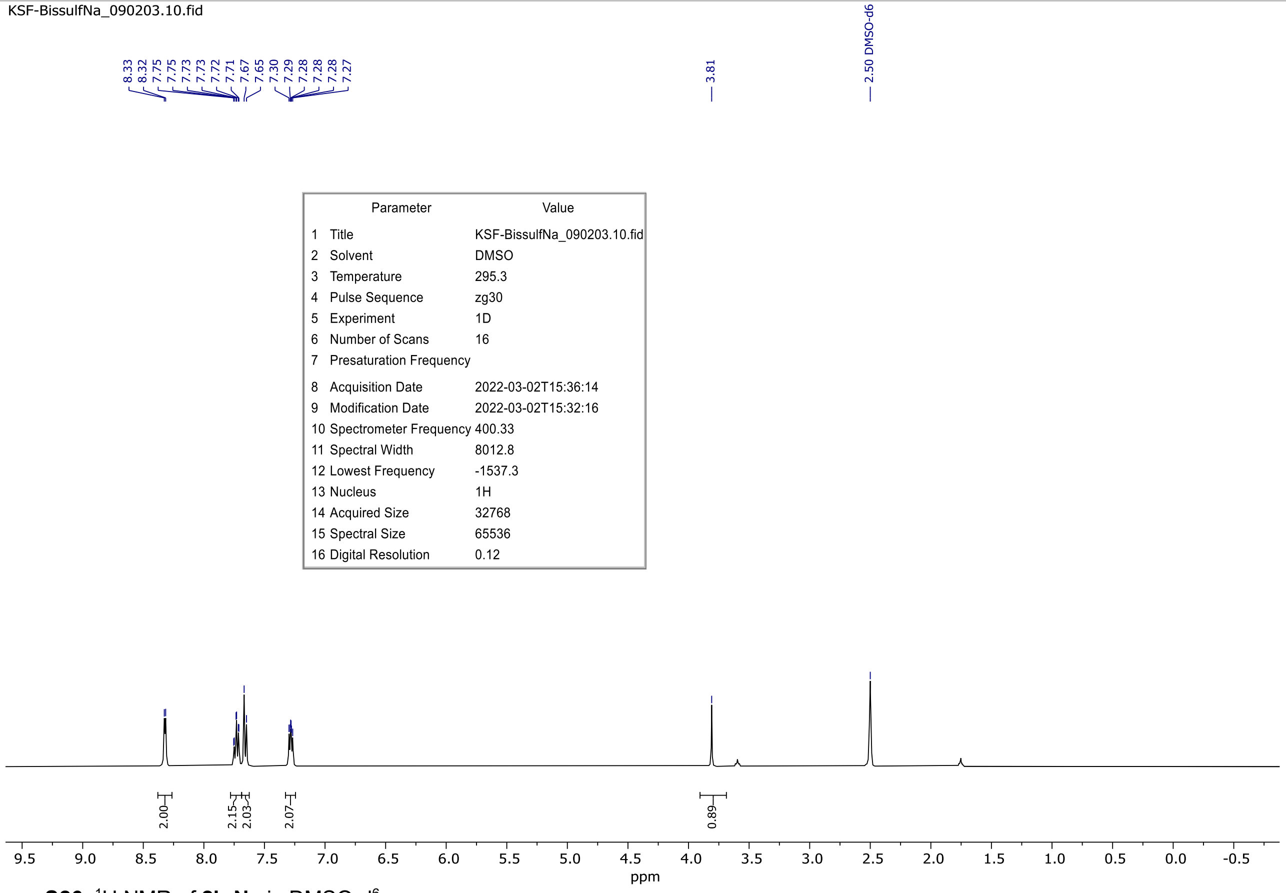

Figure S26. ^{1}H NMR of **2b-Na** in DMSO-d^6.

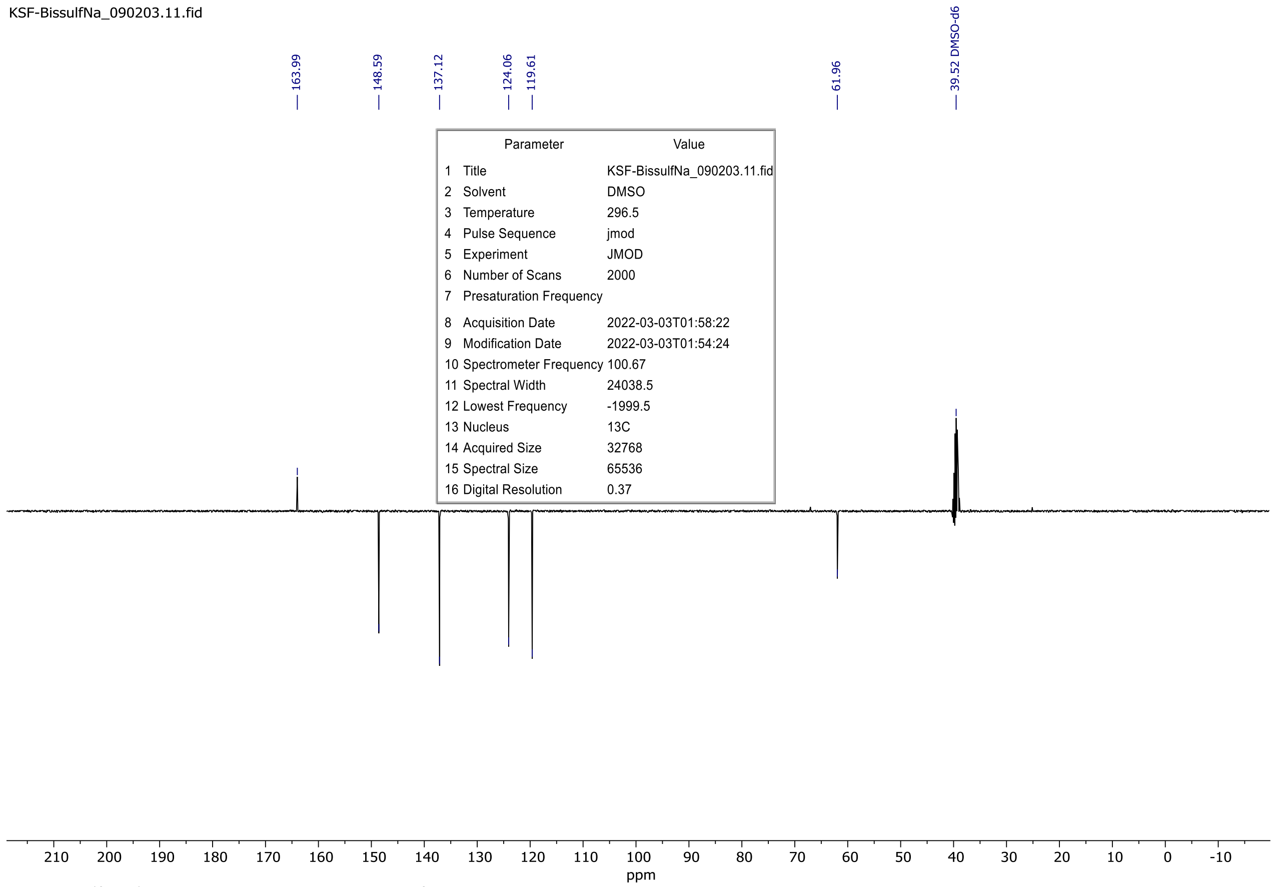

Figure S27. ^{13}C-{^{1}H} NMR of **2b-Na** in DMSO-d^6.

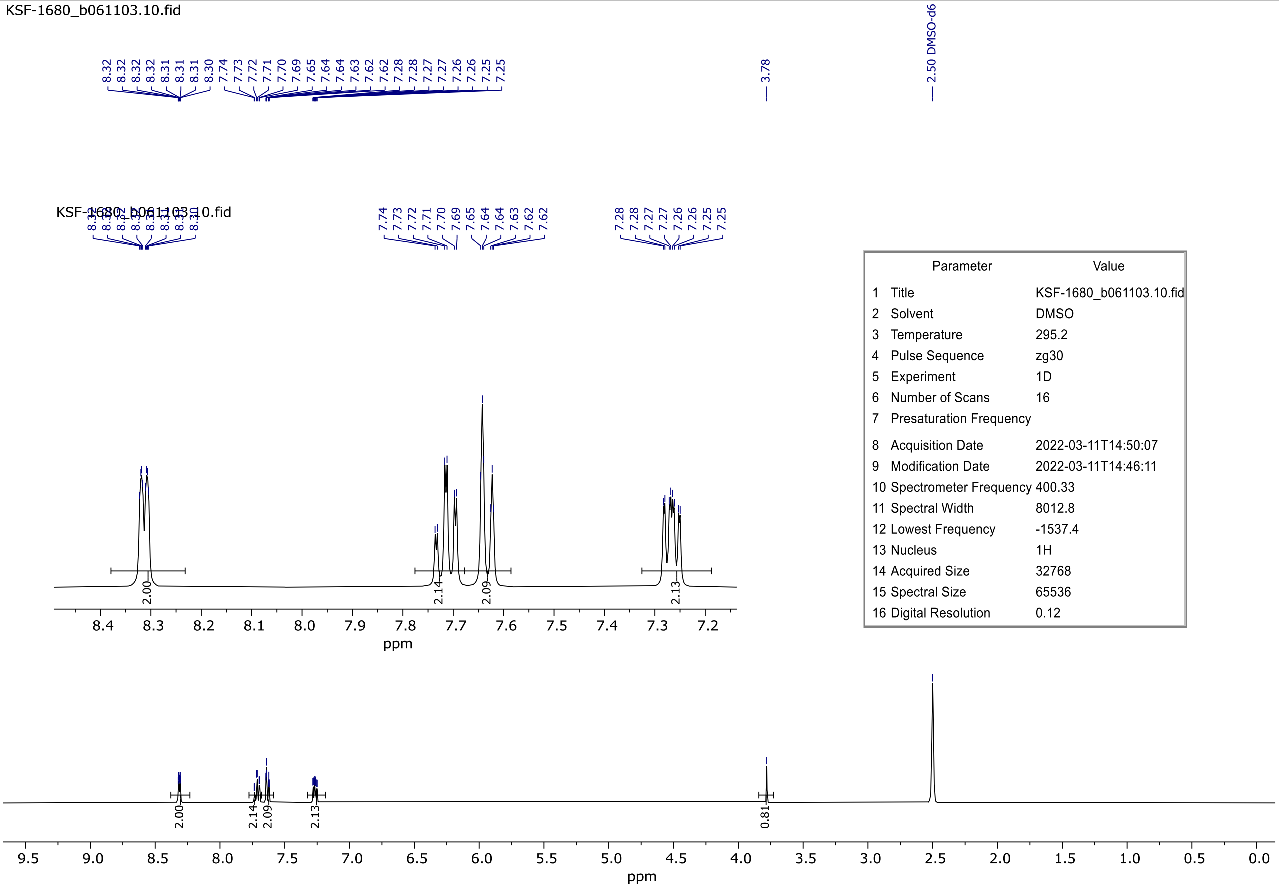

Figure S28. ^{1}H NMR of **2b-K** in DMSO-d^{6}.

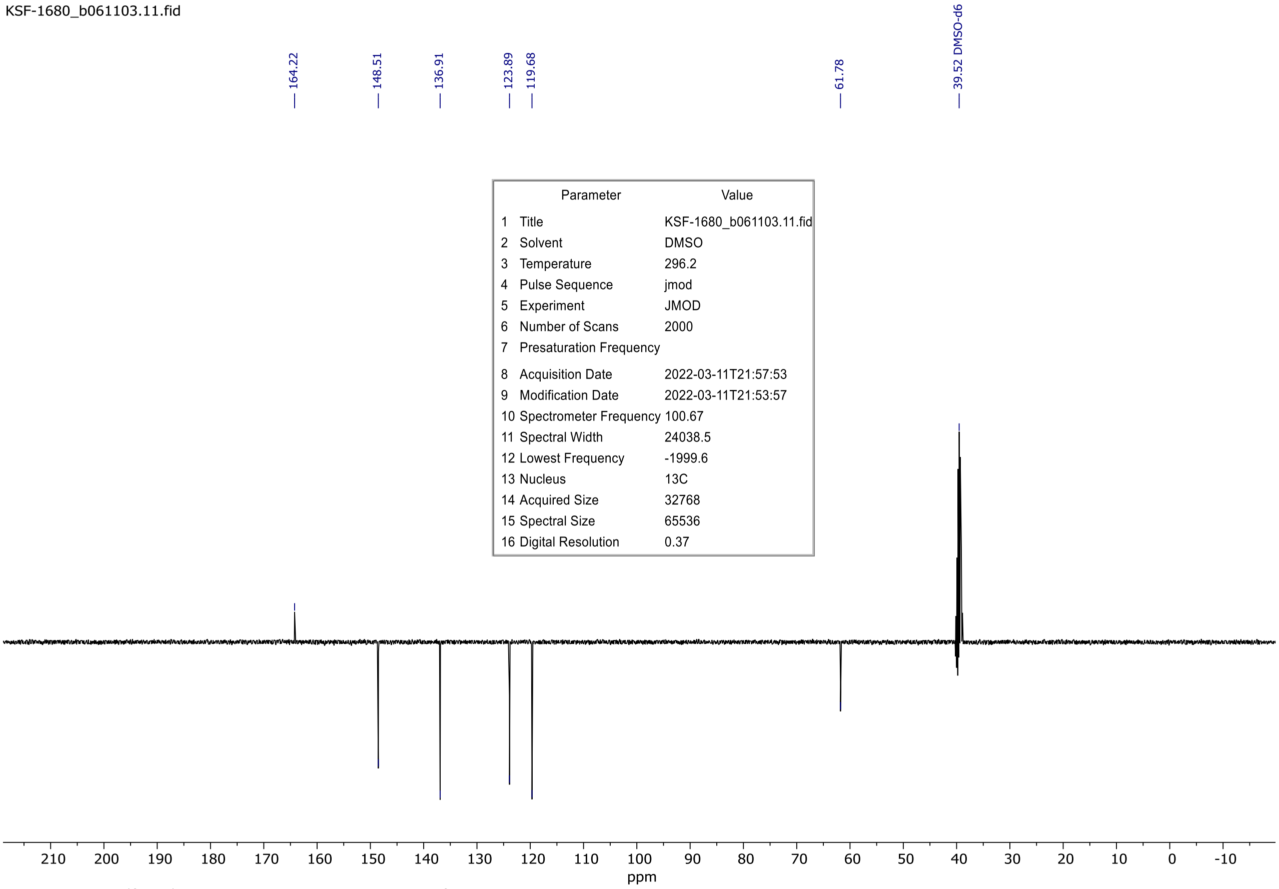

Figure S29. ^{13}C-{^{1}H} NMR of **2b-K** in DMSO-d^{6}.

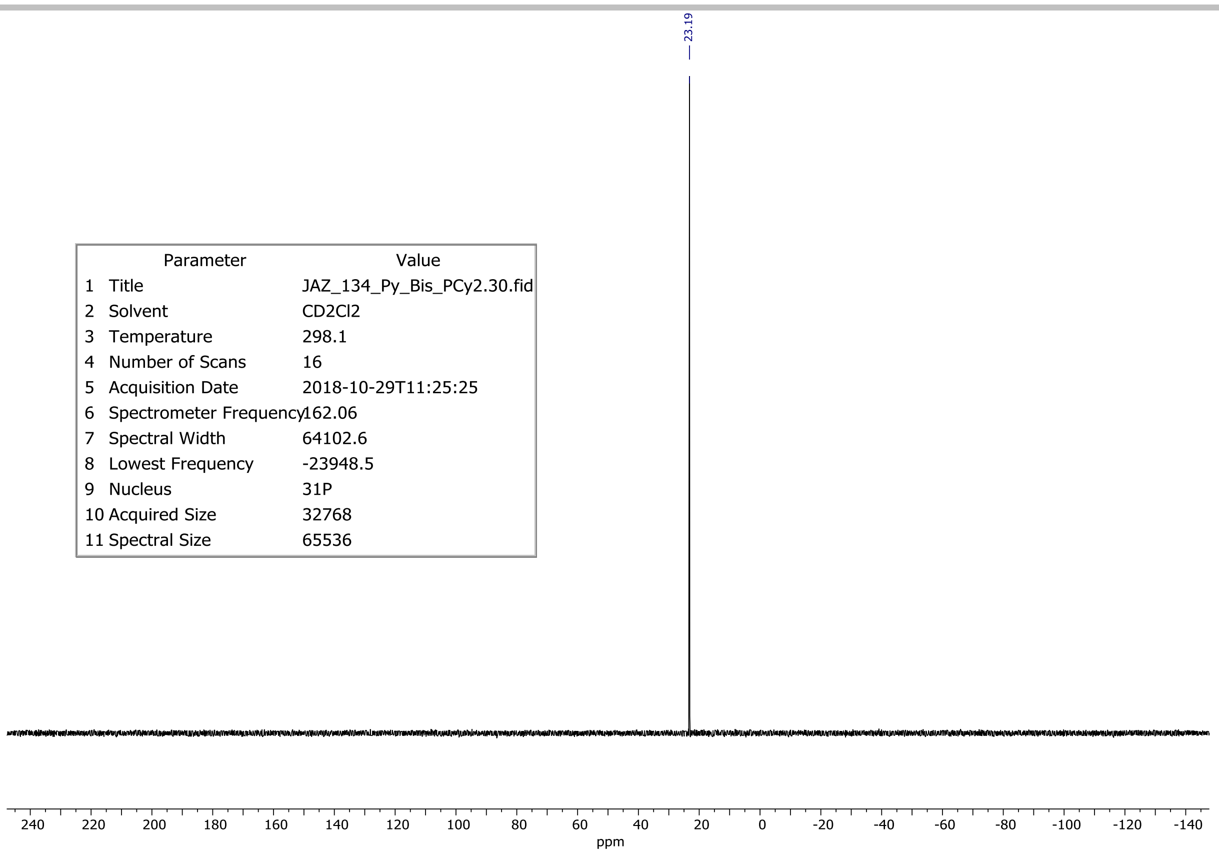

Figure S30. $^{31}P\{^{1}H\}$ NMR of **3b-PCy$_2$** in CD_2Cl_2.

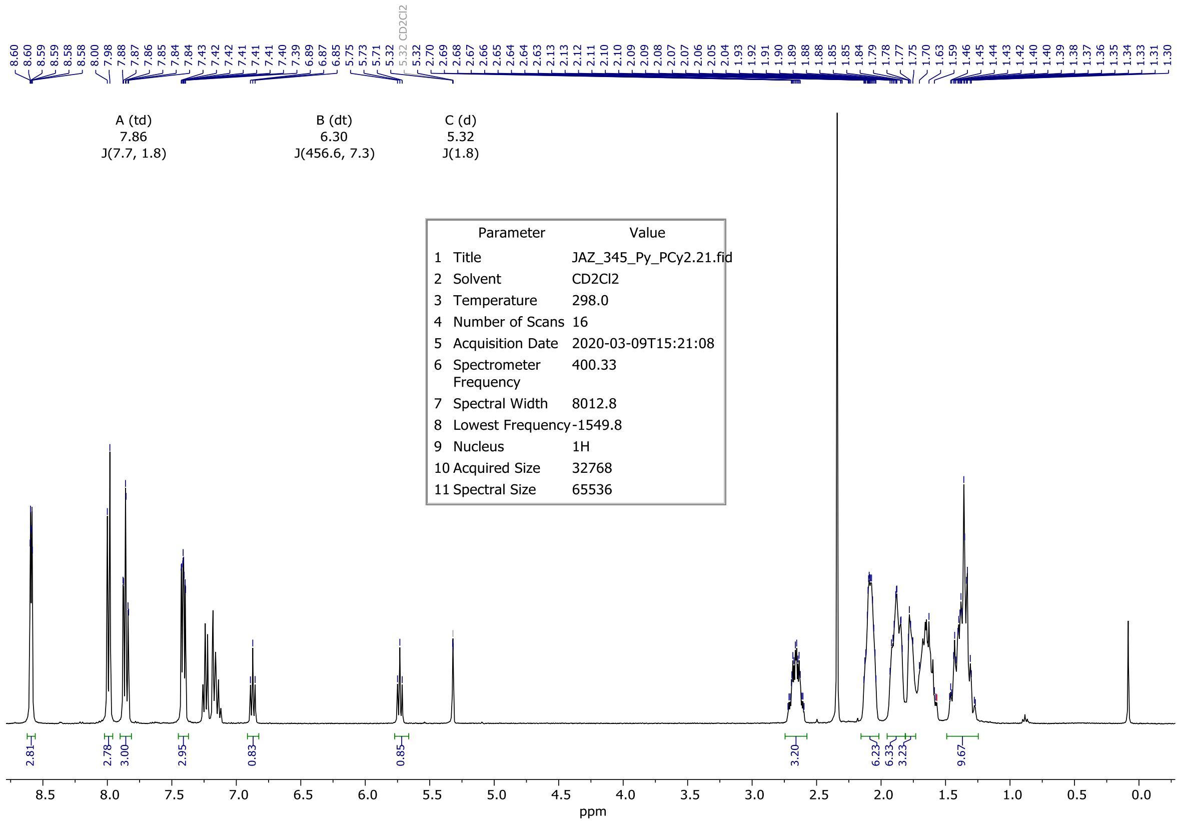

Figure S31. ^{1}H NMR of **3b-PCy$_2$** in CD_2Cl_2.

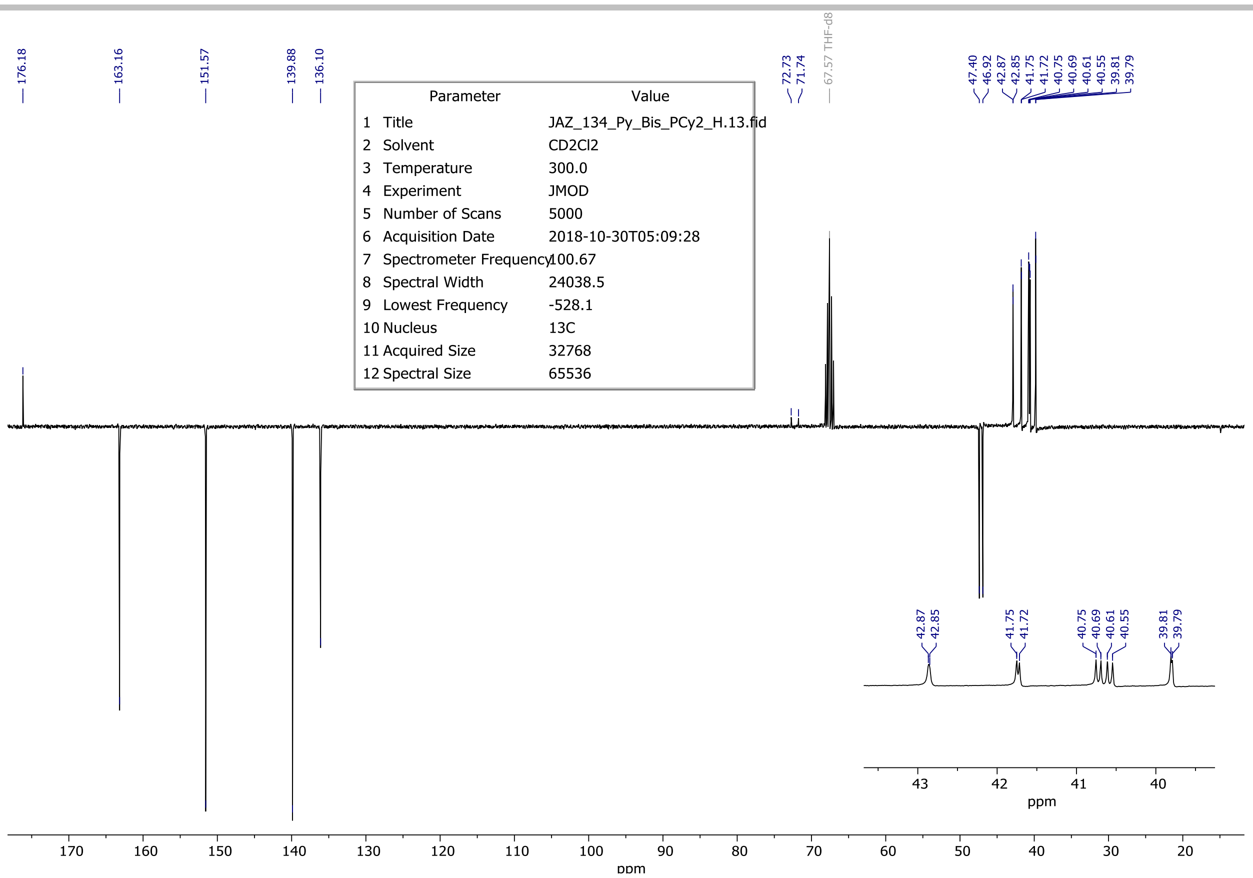

Figure S32. $^{13}C\{^1H\}$ NMR spectrum of **3b-PCy$_2$** in THF-d^8.

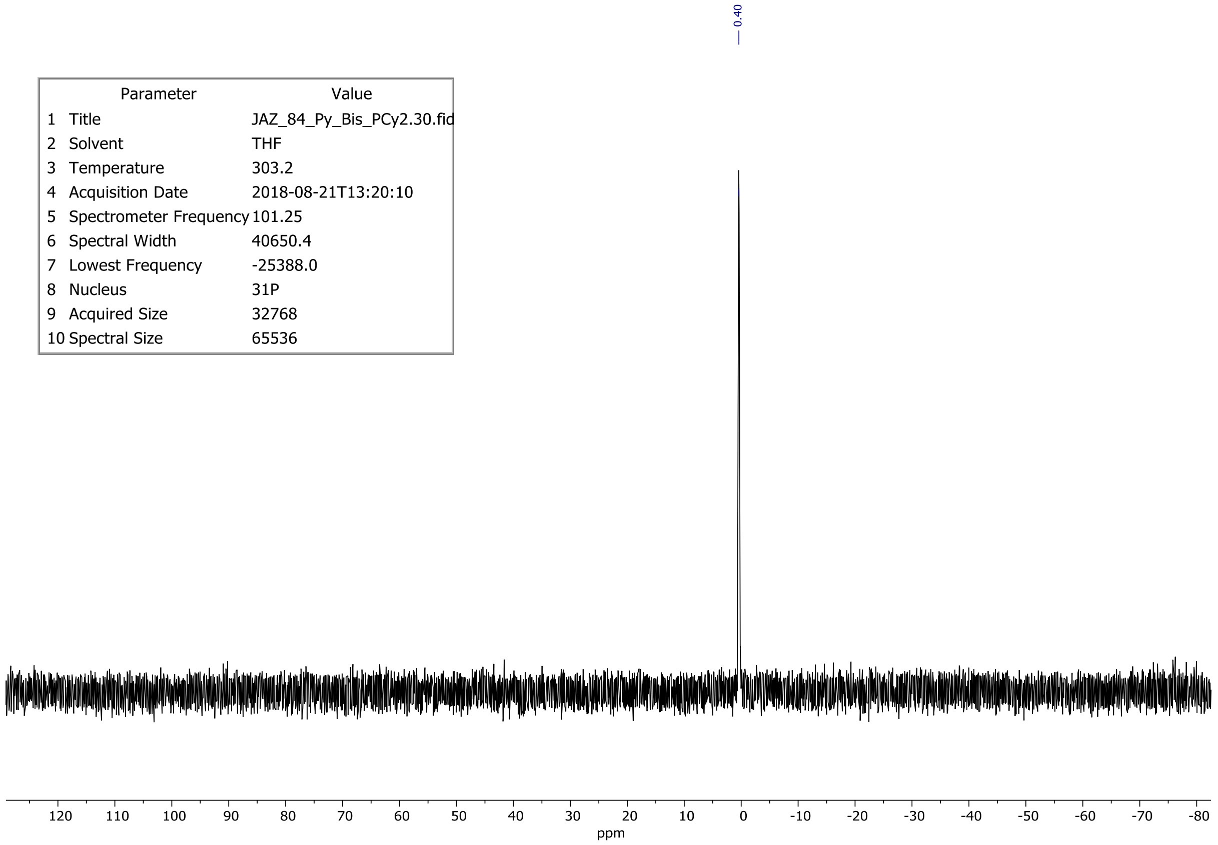

Figure S33. $^{31}P\{^1H\}$ NMR of **4b-PCy$_2$** in THF-d^8.

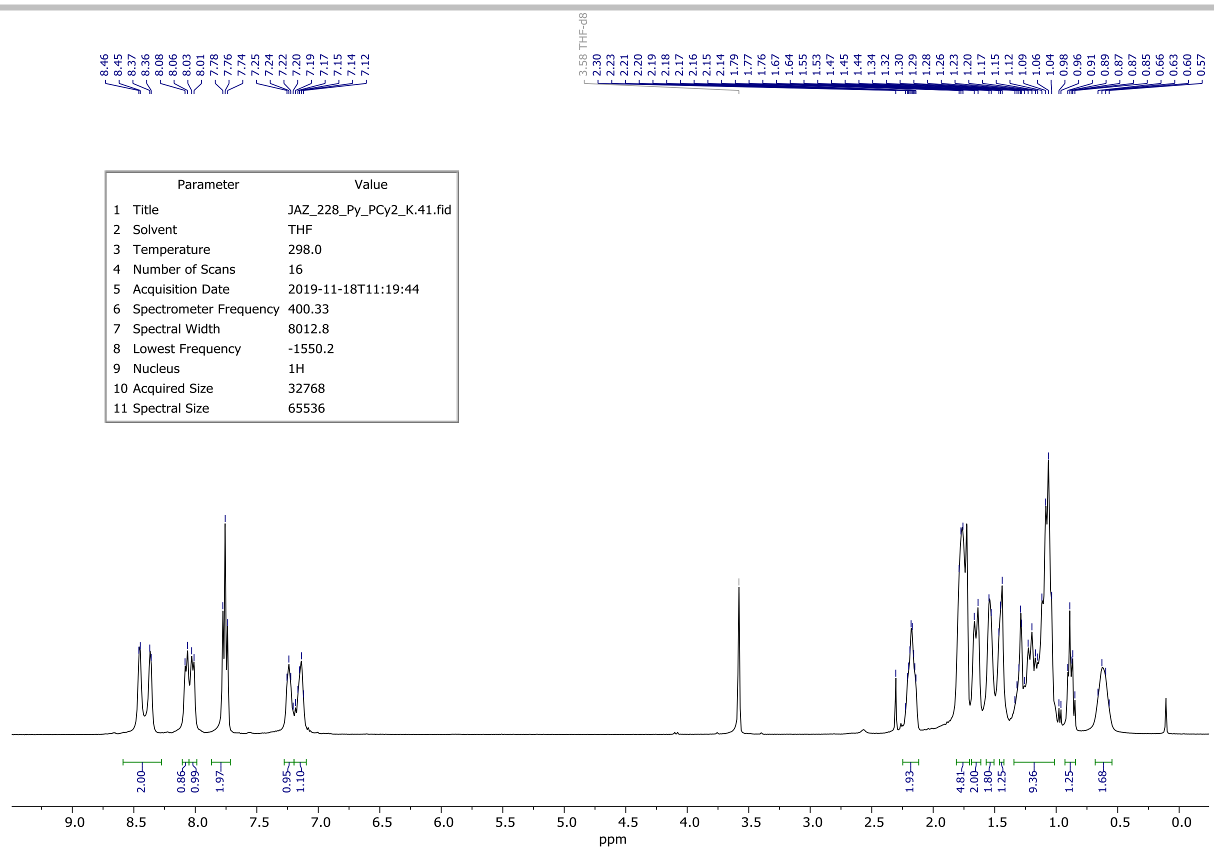

	Parameter	Value
1	Title	JAZ_228_Py_PCy2_K.41.fid
2	Solvent	THF
3	Temperature	298.0
4	Number of Scans	16
5	Acquisition Date	2019-11-18T11:19:44
6	Spectrometer Frequency	400.33
7	Spectral Width	8012.8
8	Lowest Frequency	-1550.2
9	Nucleus	1H
10	Acquired Size	32768
11	Spectral Size	65536

Figure S34. ^{1}H NMR of **4b-PCy$_2$** in THF-d^8.

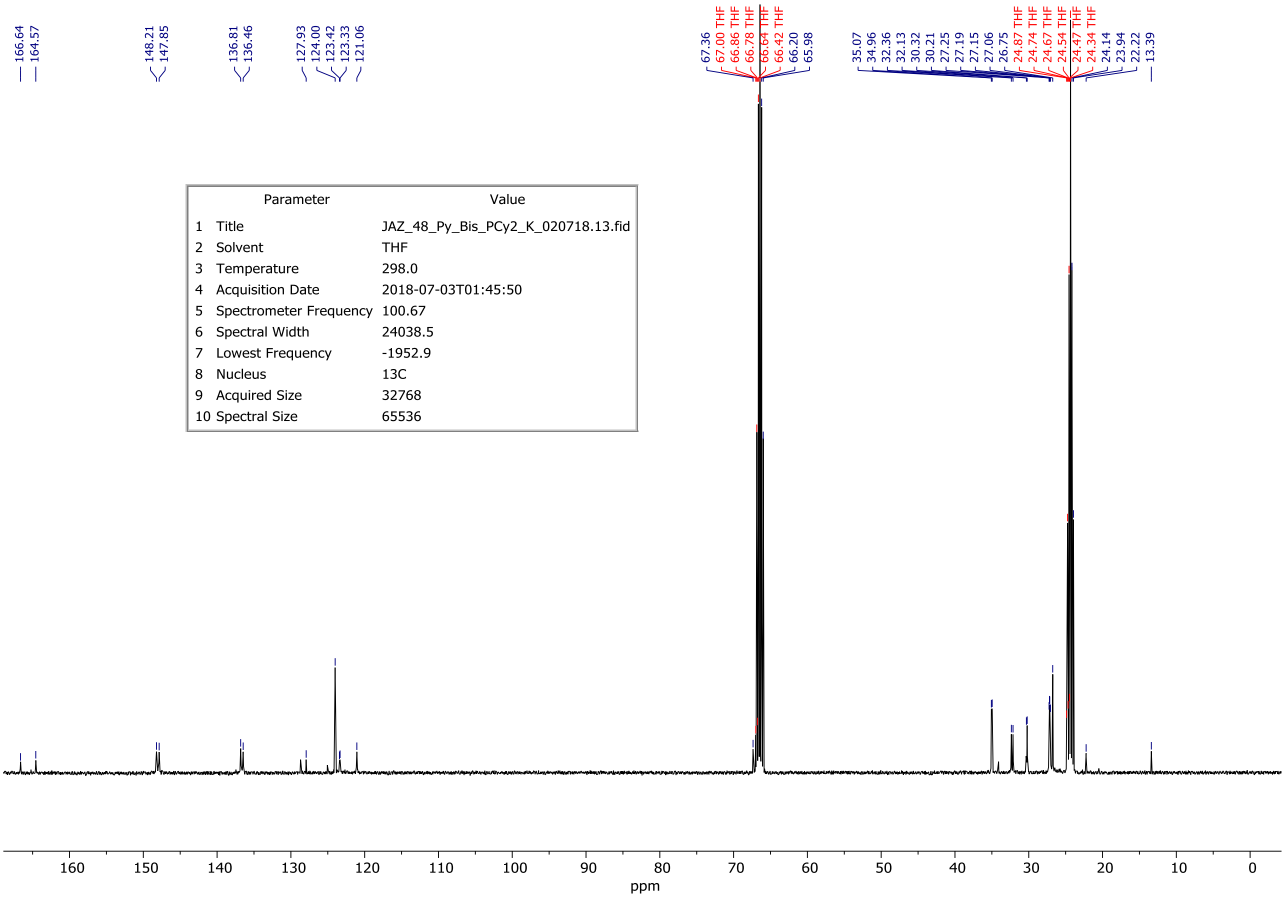

	Parameter	Value
1	Title	JAZ_48_Py_Bis_PCy2_K_020718.13.fid
2	Solvent	THF
3	Temperature	298.0
4	Acquisition Date	2018-07-03T01:45:50
5	Spectrometer Frequency	100.67
6	Spectral Width	24038.5
7	Lowest Frequency	-1952.9
8	Nucleus	13C
9	Acquired Size	32768
10	Spectral Size	65536

Figure S35. ^{13}C{^{1}H} NMR spectrum of **4b-PCy$_2$** in THF-d^8.

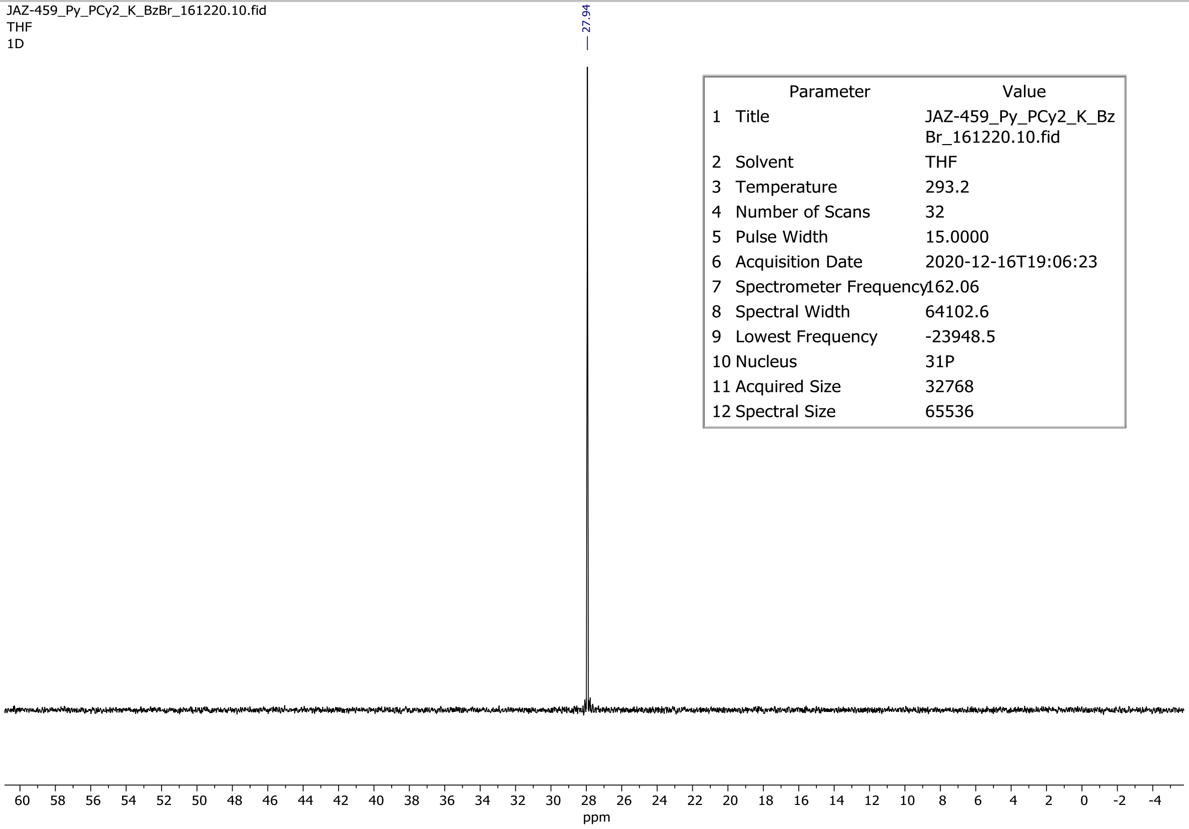

Figure S36. $^{31}P\{^1H\}$ NMR of **5b** in THF-d[8].

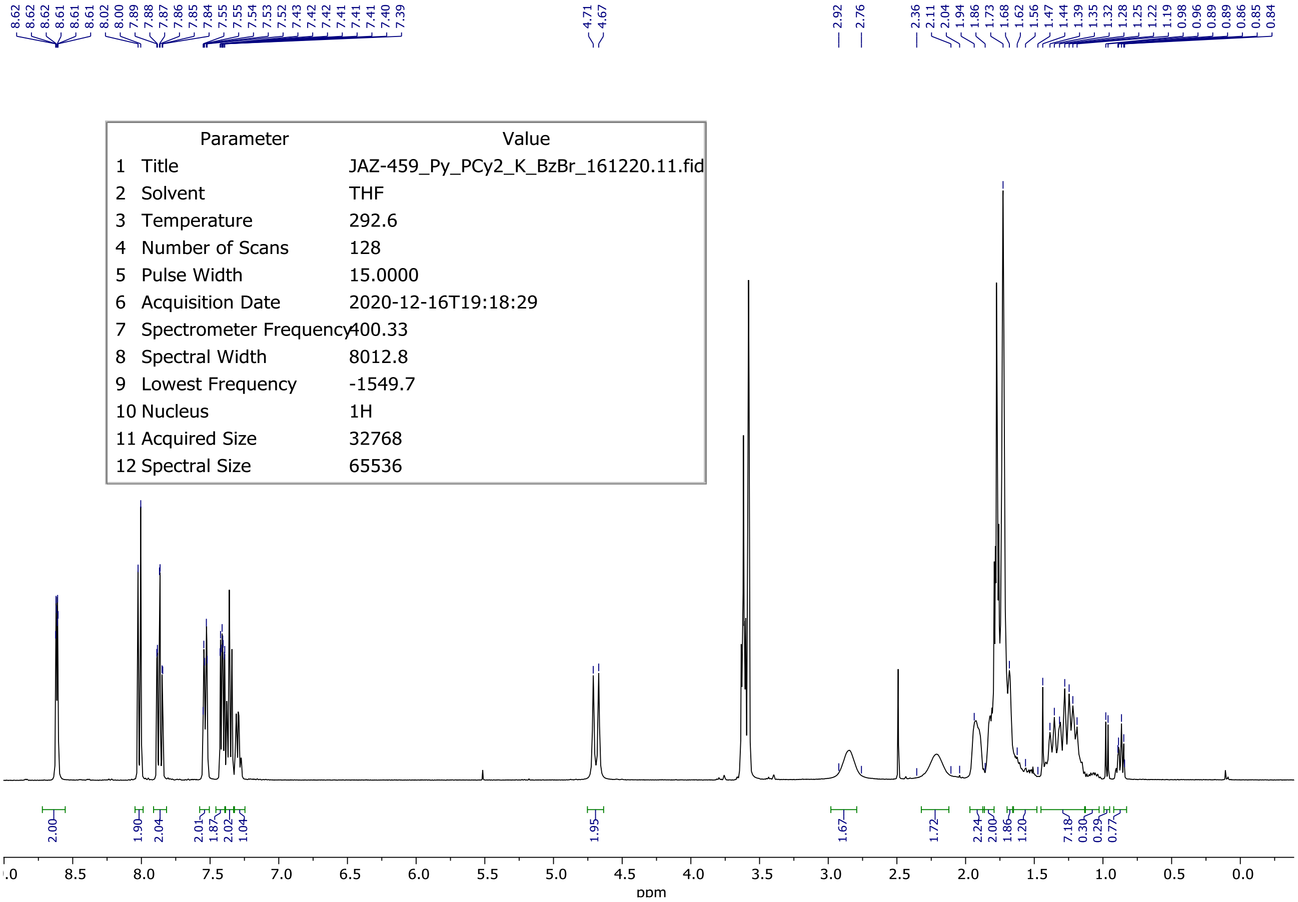

Figure S37. ^1H NMR spectrum of **5b** in THF-d[8].

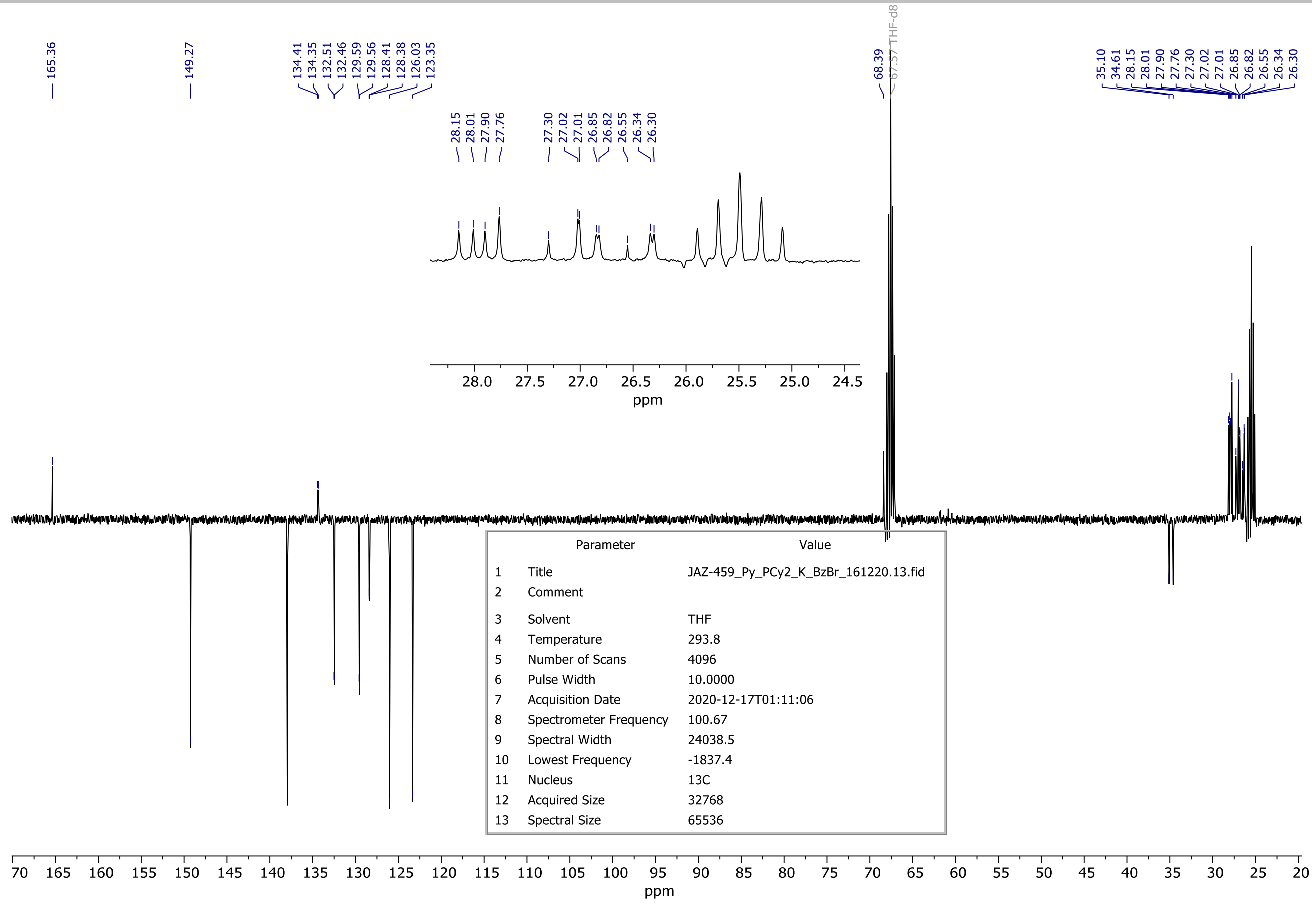

Figure S38. $^{13}C\{^{1}H\}$ NMR spectrum of **5b** in THF-d^{8}.

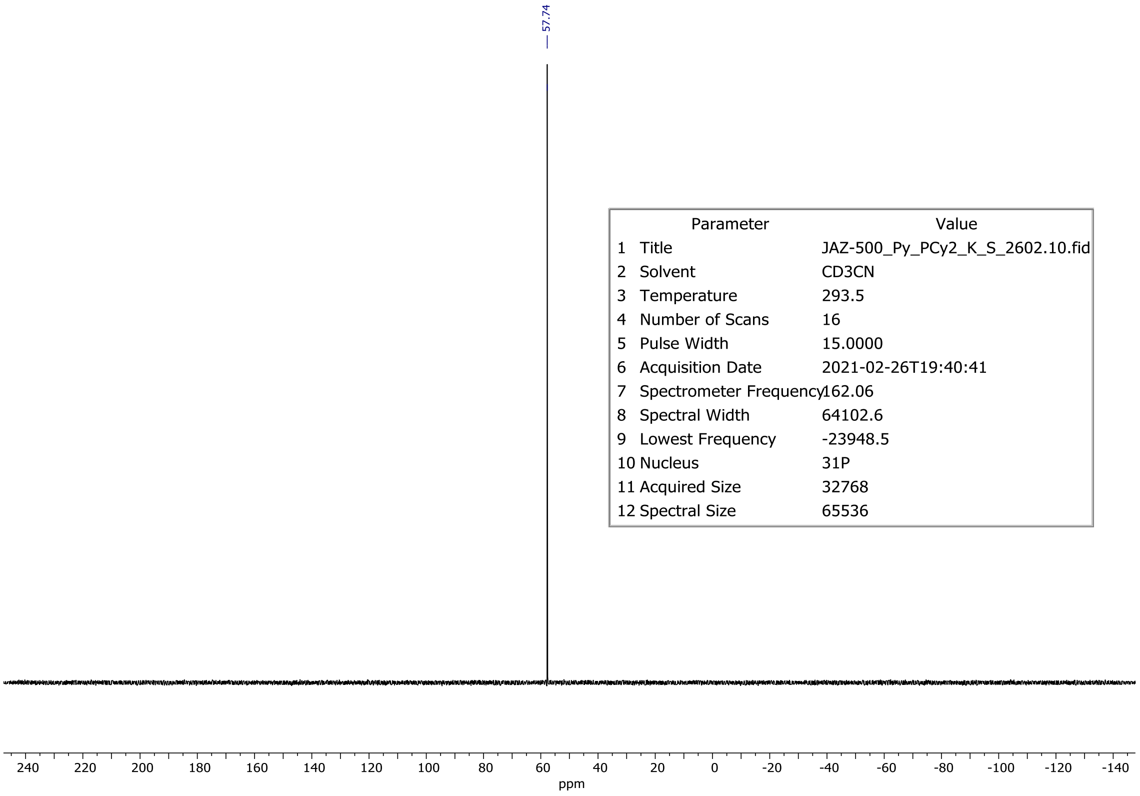

Figure S39. $^{31}P\{^{1}H\}$ NMR of **6b** in CD_3CN.

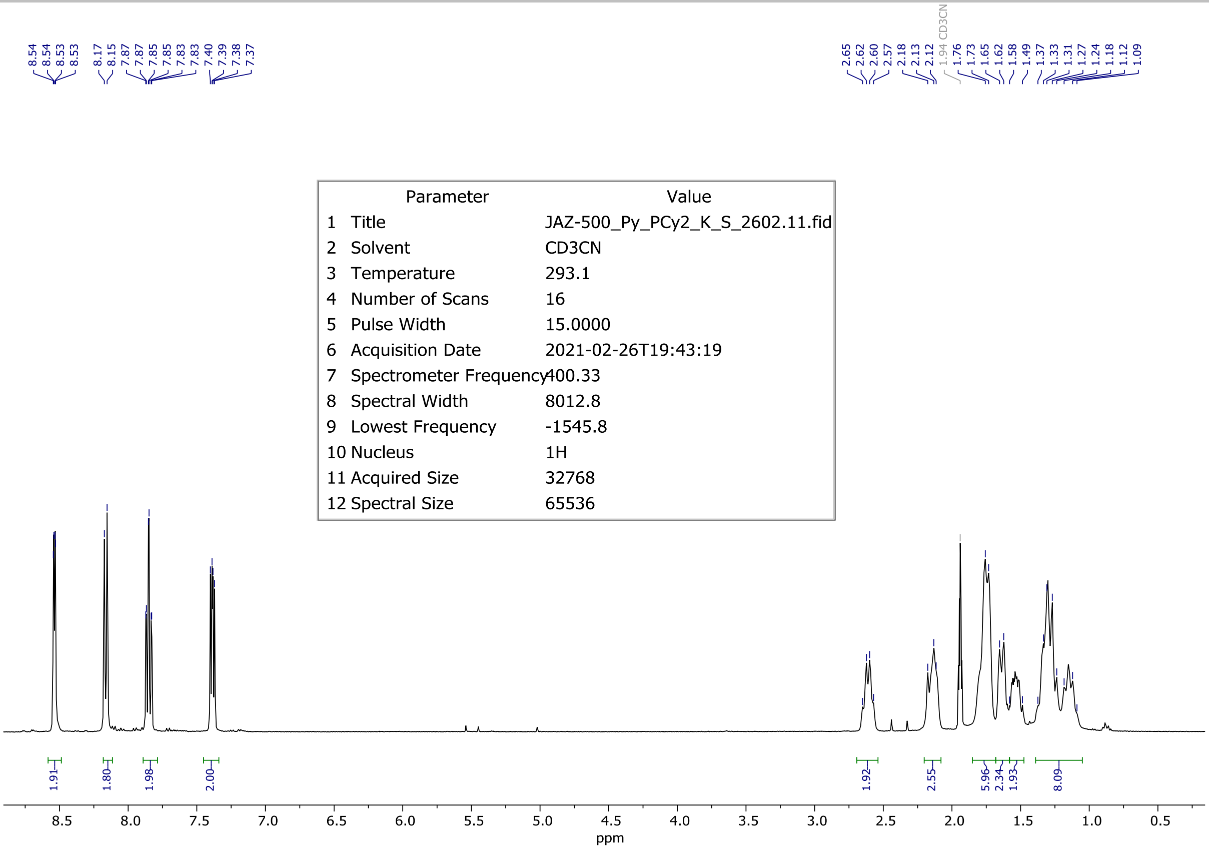

	Parameter	Value
1	Title	JAZ-500_Py_PCy2_K_S_2602.11.fid
2	Solvent	CD3CN
3	Temperature	293.1
4	Number of Scans	16
5	Pulse Width	15.0000
6	Acquisition Date	2021-02-26T19:43:19
7	Spectrometer Frequency	400.33
8	Spectral Width	8012.8
9	Lowest Frequency	-1545.8
10	Nucleus	1H
11	Acquired Size	32768
12	Spectral Size	65536

Figure S40. ^{1}H NMR spectrum of **6b** in CD_3CN.

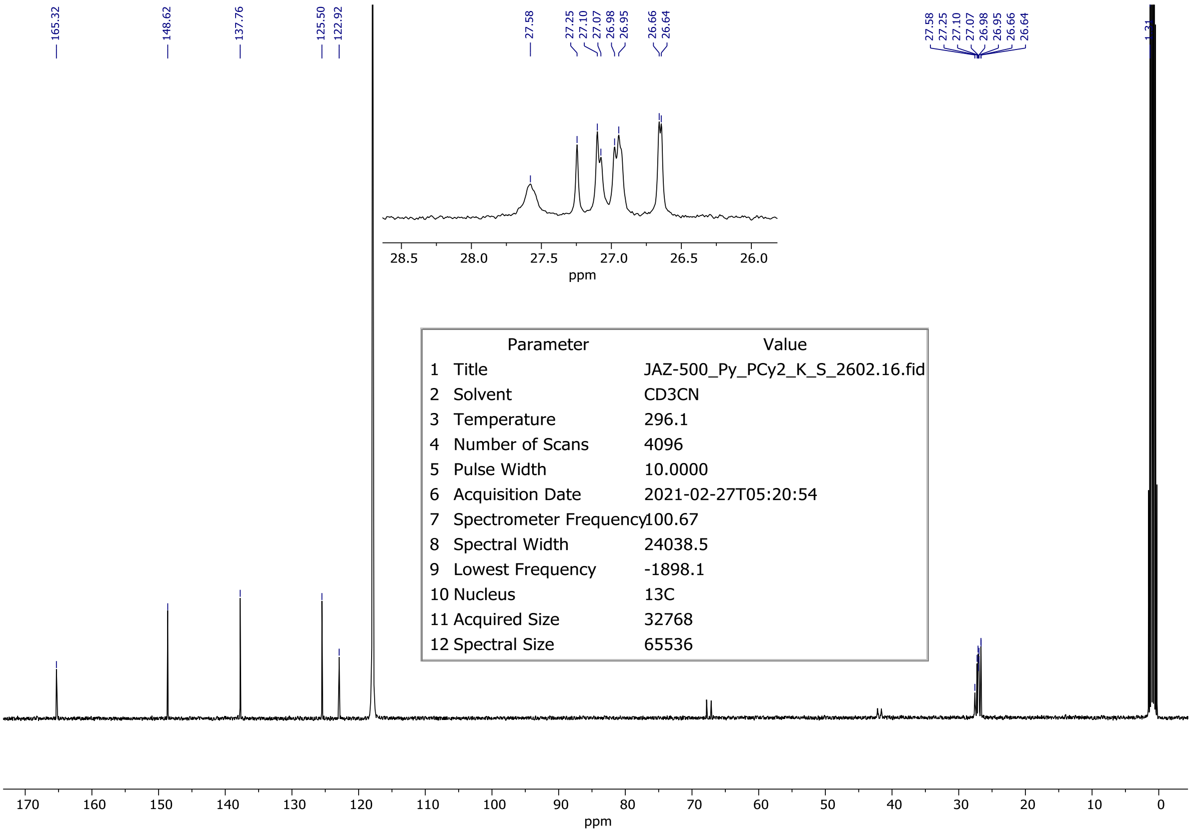

	Parameter	Value
1	Title	JAZ-500_Py_PCy2_K_S_2602.16.fid
2	Solvent	CD3CN
3	Temperature	296.1
4	Number of Scans	4096
5	Pulse Width	10.0000
6	Acquisition Date	2021-02-27T05:20:54
7	Spectrometer Frequency	100.67
8	Spectral Width	24038.5
9	Lowest Frequency	-1898.1
10	Nucleus	13C
11	Acquired Size	32768
12	Spectral Size	65536

Figure S41. $^{13}C\{^{1}H\}$ NMR spectrum of **6b** in CD_3CN.

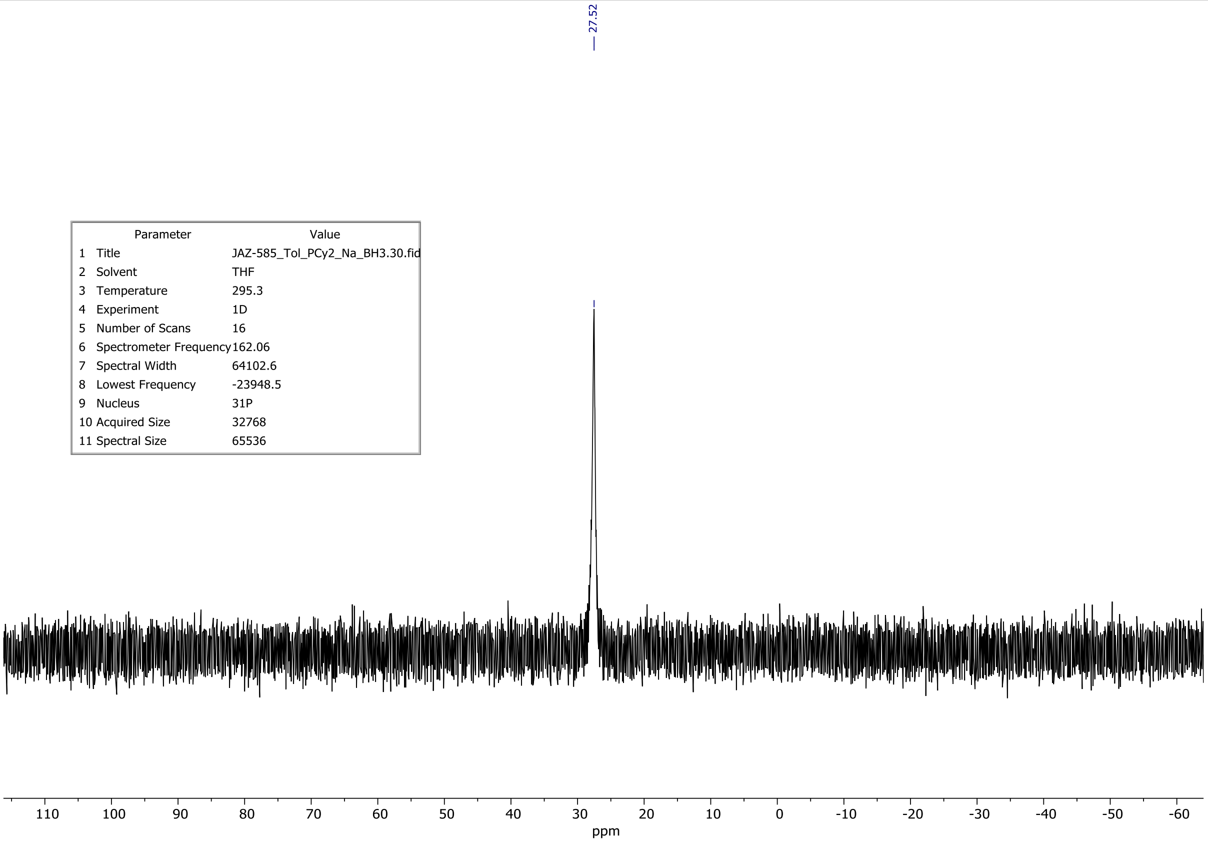

Figure S42. $^{31}P\{^{1}H\}$ NMR of **6a** in THF-d^{8}.

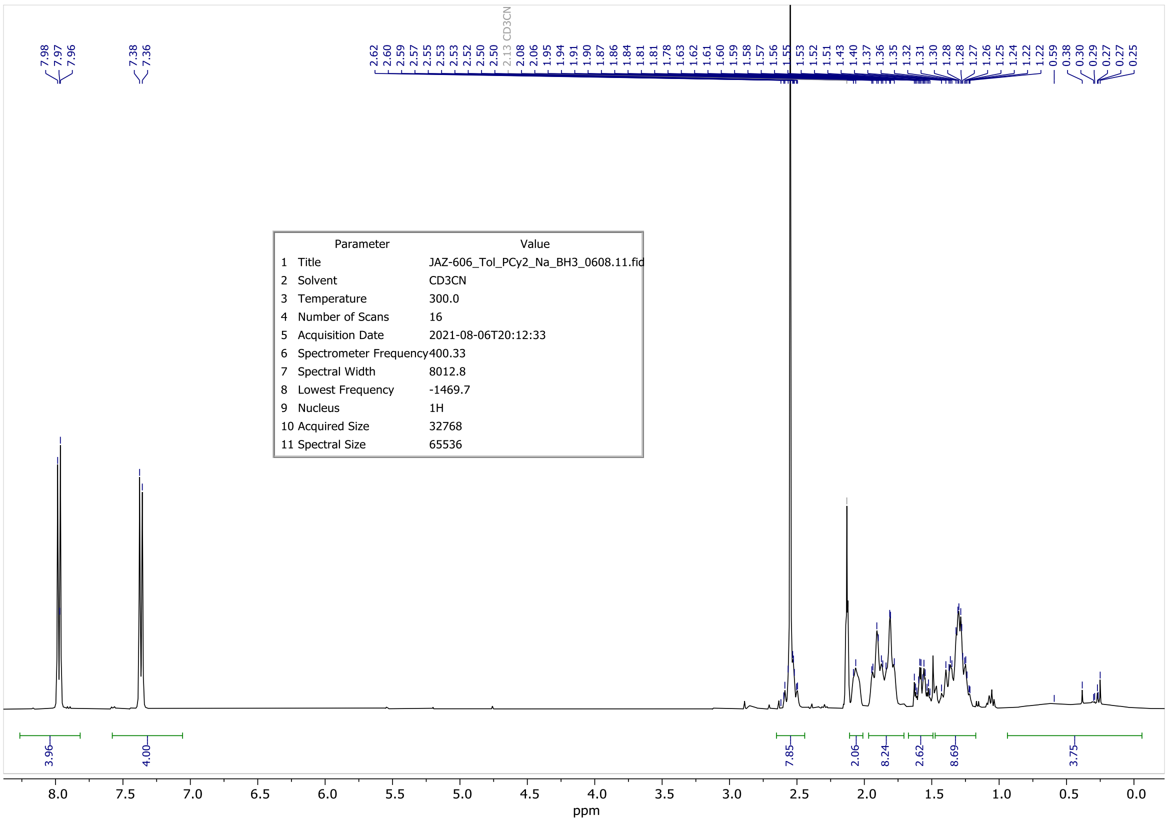

Figure S43. ^{1}H NMR spectrum of **6a** in CD_3CN.

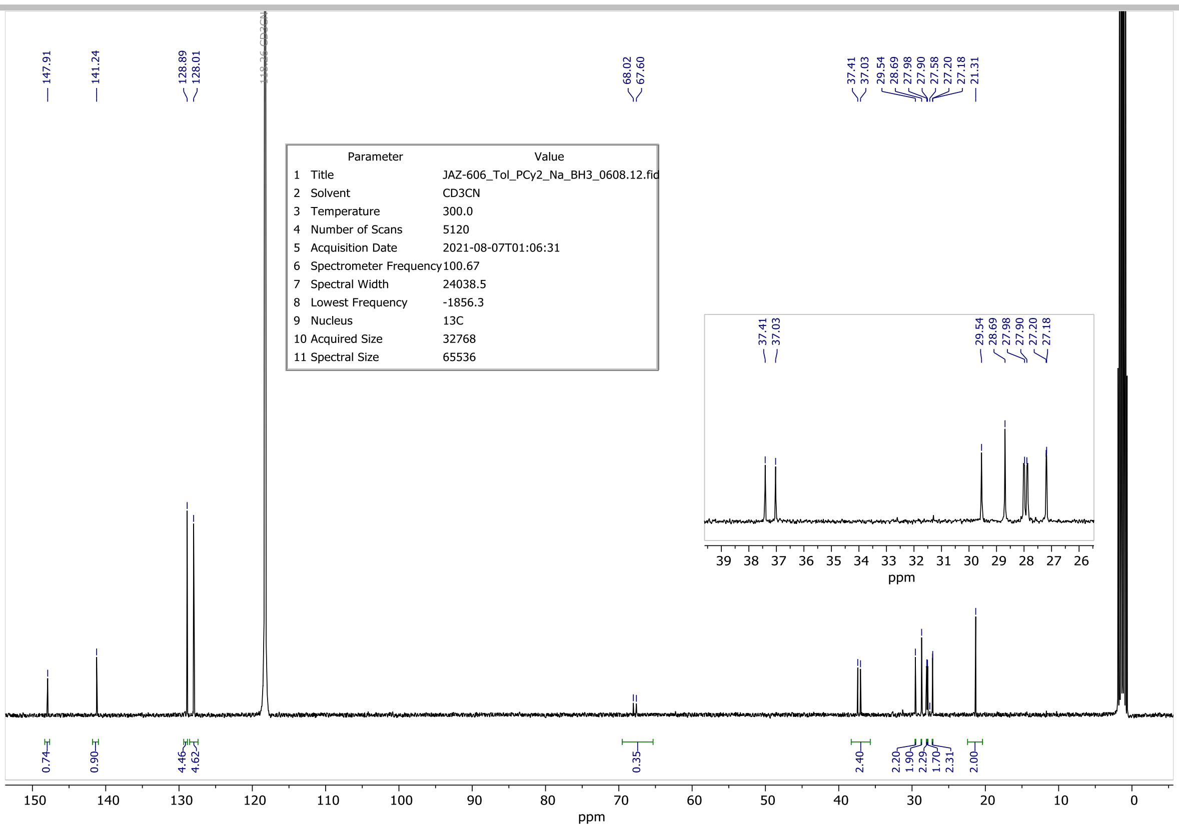

Figure S44. $^{13}C\{^{1}H\}$ NMR spectrum of **6a** in CD_3CN.

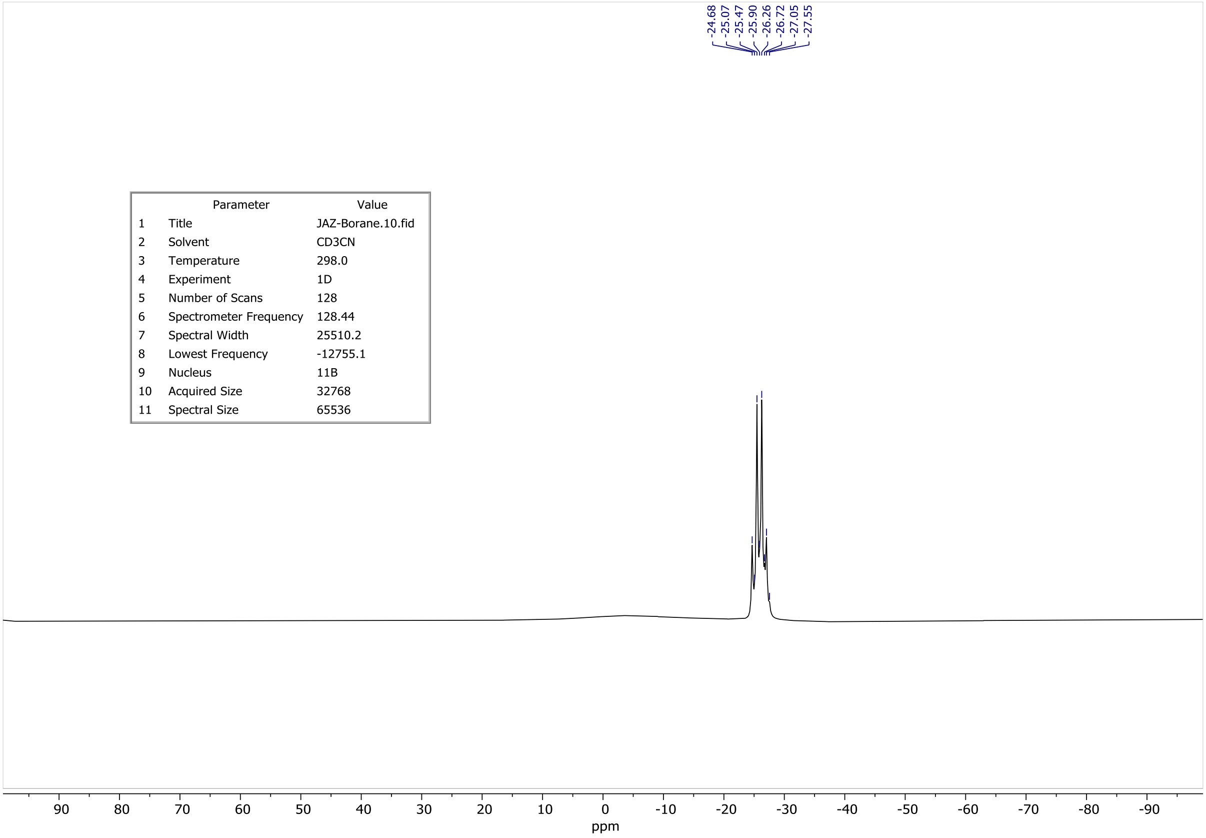

Figure S45. ^{11}B NMR spectrum of **6a** in CD_3CN.

2.2 NMR spectra for determination of the ylide/phosphine equilibria

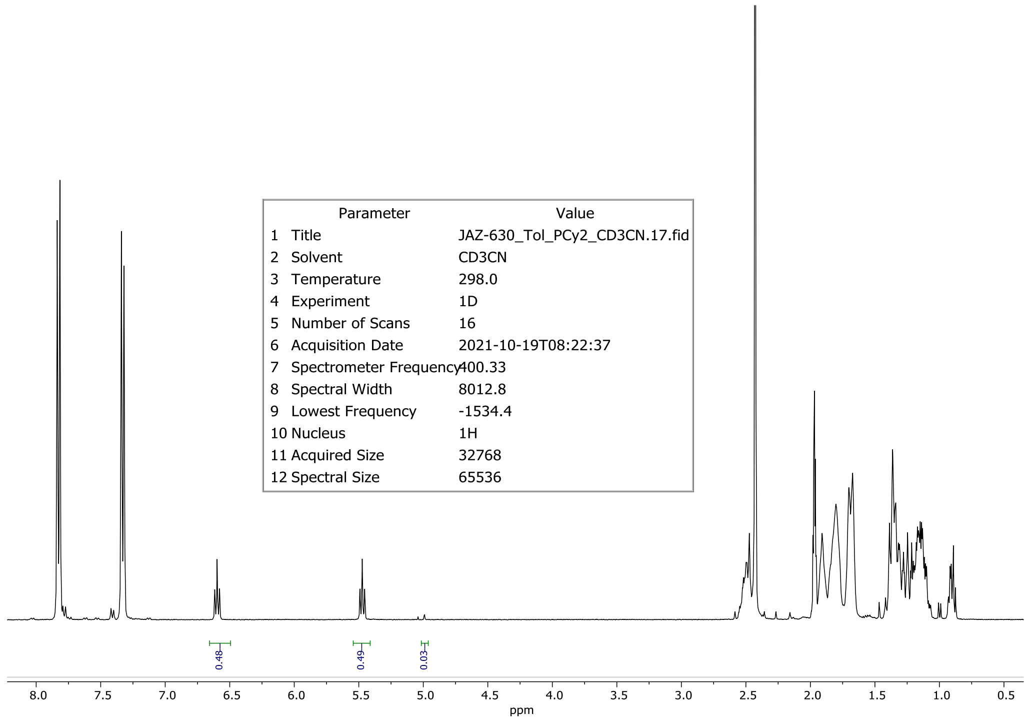

Figure S46. ^{1}H NMR spectrum of **3a-Cy/3a'-Cy** in CD_3CN.

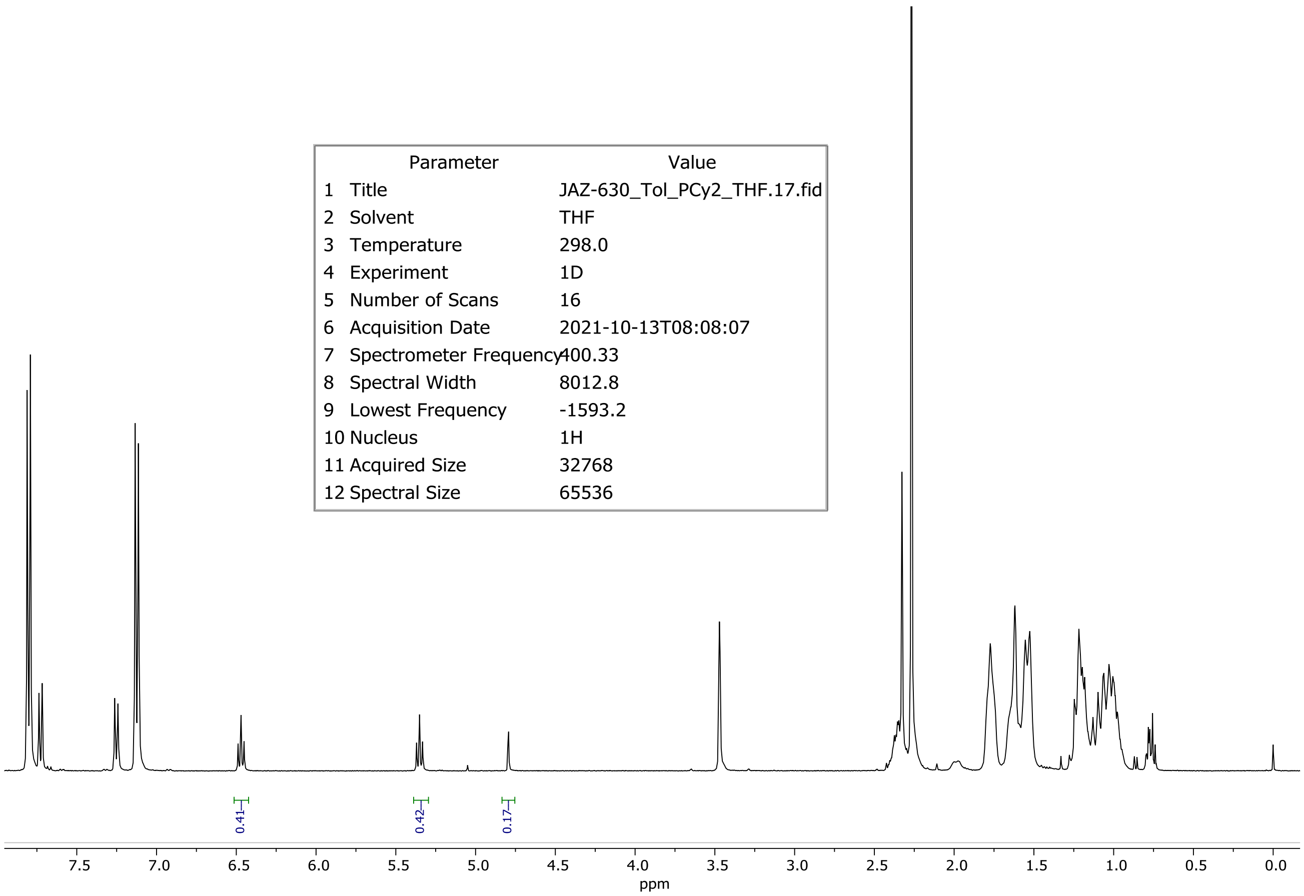

Figure S47. ^{1}H NMR spectrum of **3a-Cy/3a'-Cy** in THF-d^{8}.

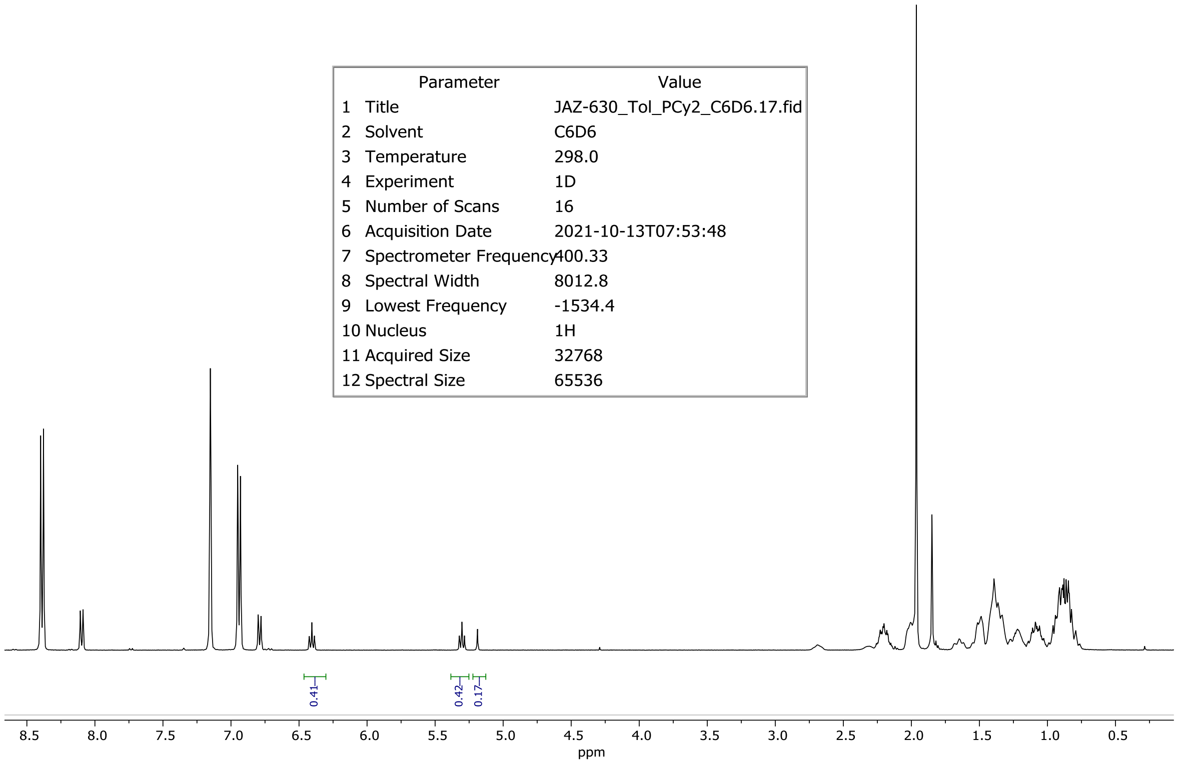

Figure S48. ^{1}H NMR spectrum of **3a-Cy/3a'-Cy** in C_6D_6.

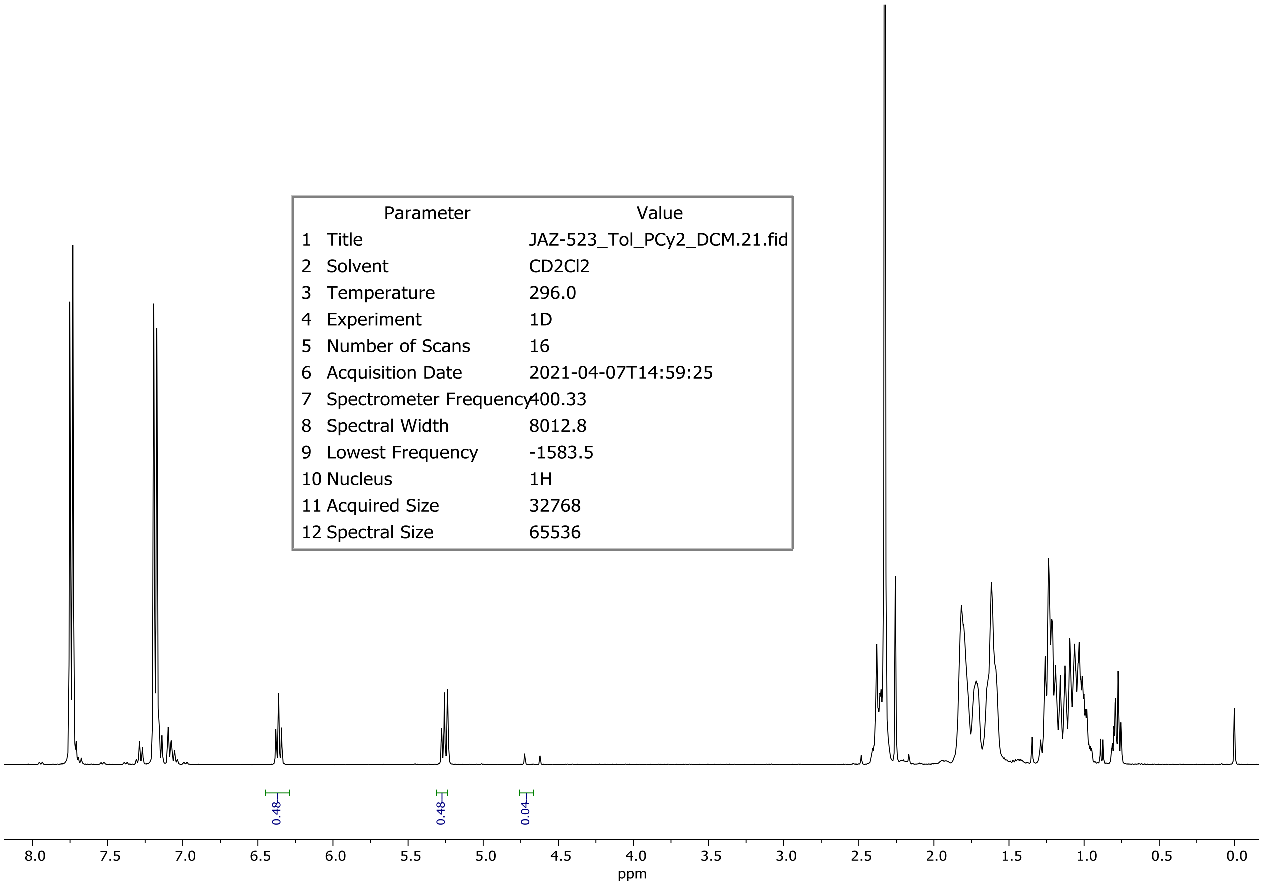

Figure S49. ^{1}H NMR spectrum of **3a-Cy/3a'-Cy** in CD_2Cl_2.

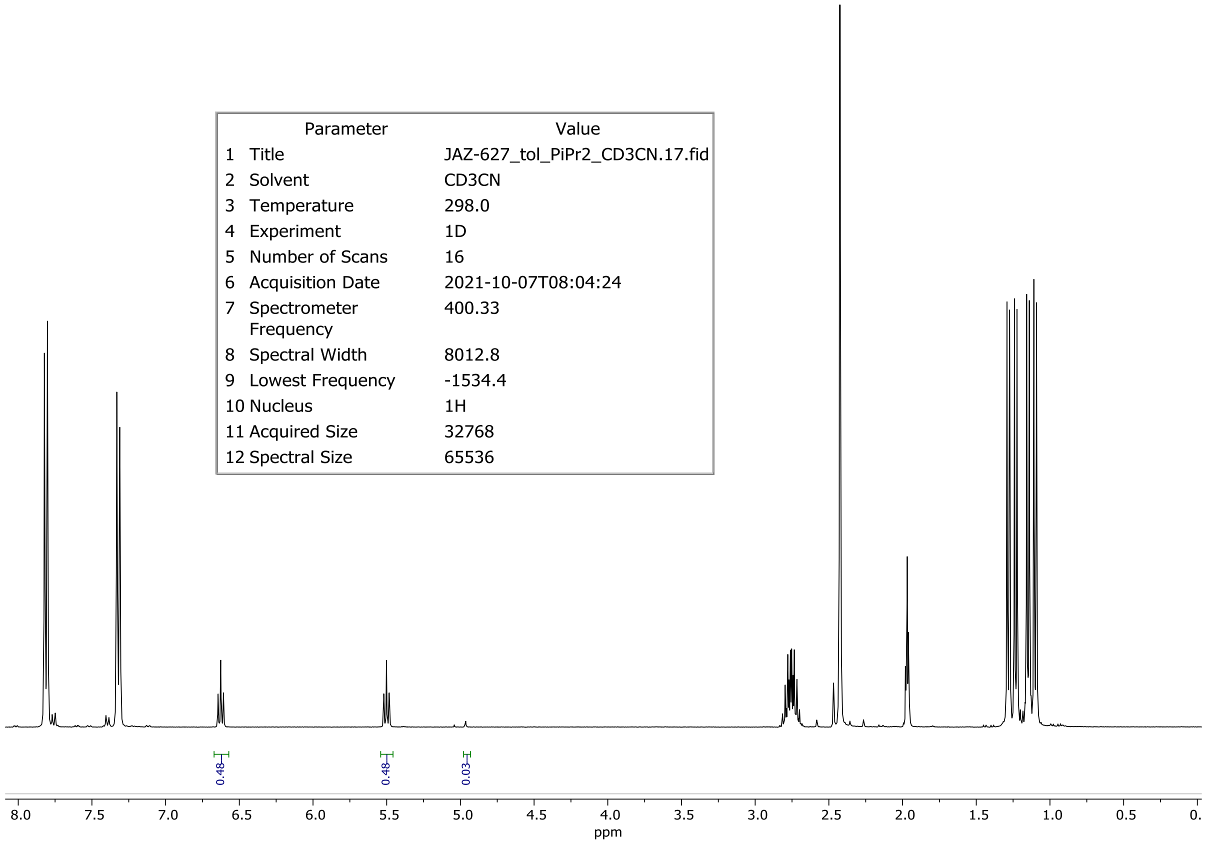

Figure S50. ^{1}H NMR spectrum of **3a-*i*Pr/3a'-*i*Pr** in CD_3CN.

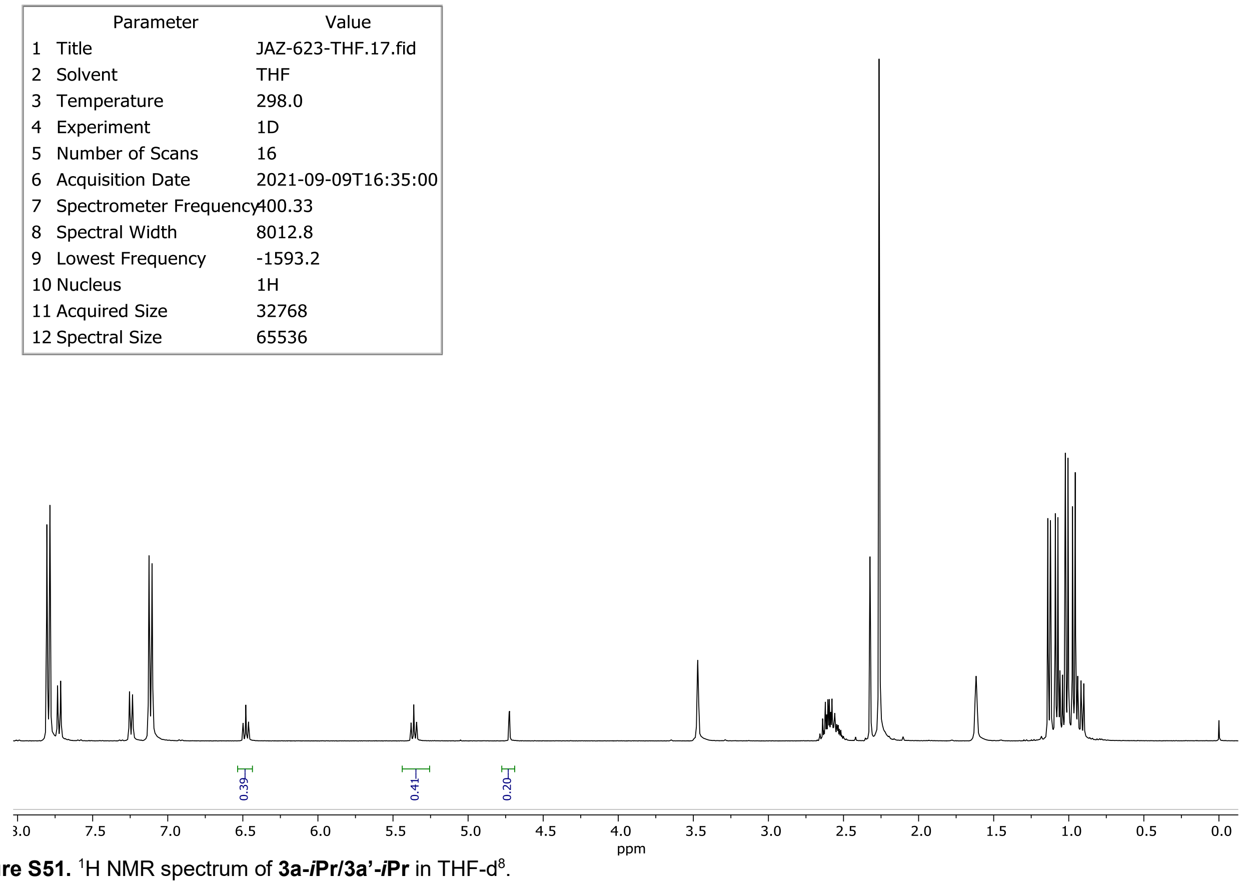

Figure S51. ^{1}H NMR spectrum of **3a-*i*Pr/3a'-*i*Pr** in THF-d^8.

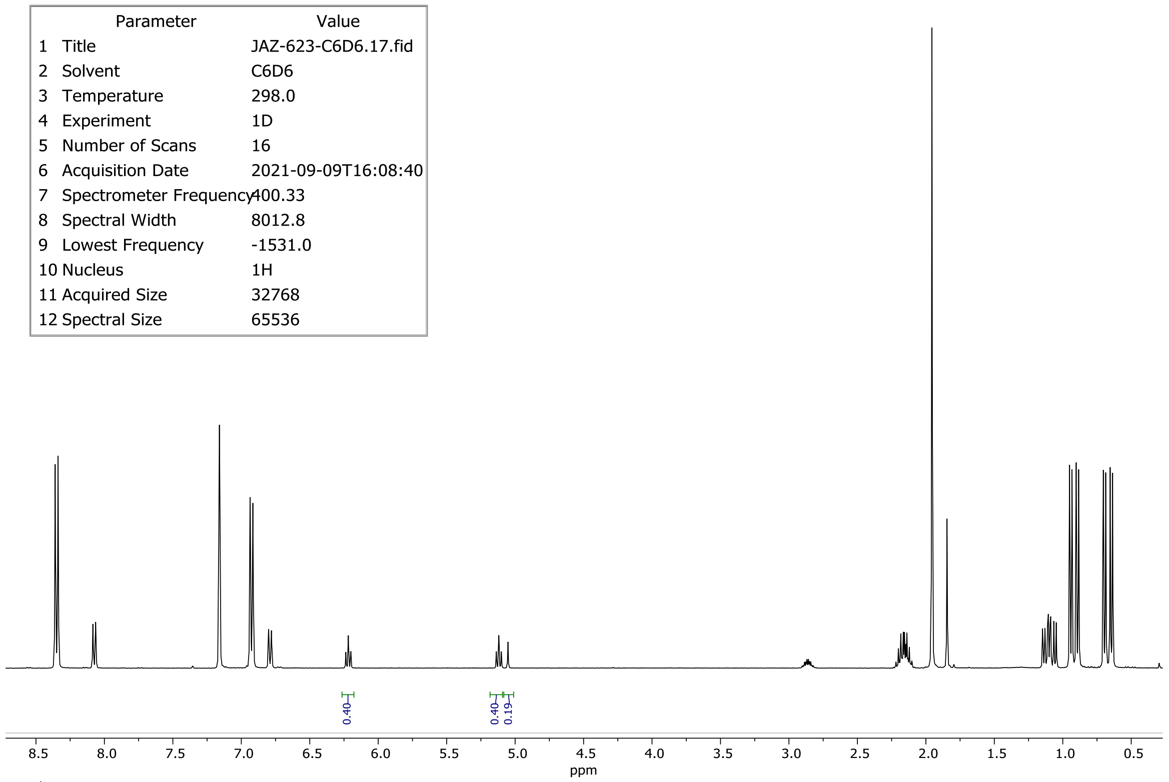

Figure S52. ^{1}H NMR spectrum of **3a-*i*Pr/3a’-*i*Pr** in C_6D_6.

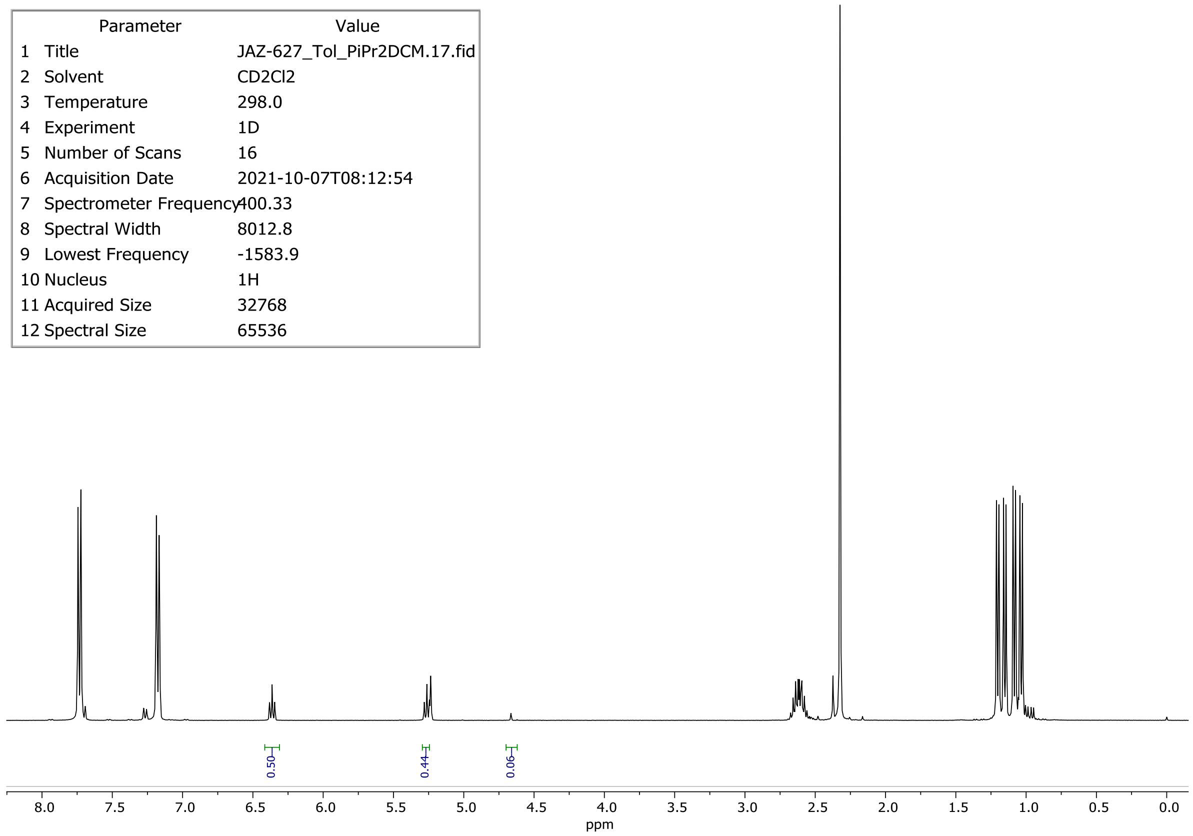

Figure S53. ^{1}H NMR spectrum of **3a-*i*Pr/3a’-*i*Pr** in CD_2Cl_2.

2.3 NMR spectra of the gold complexes

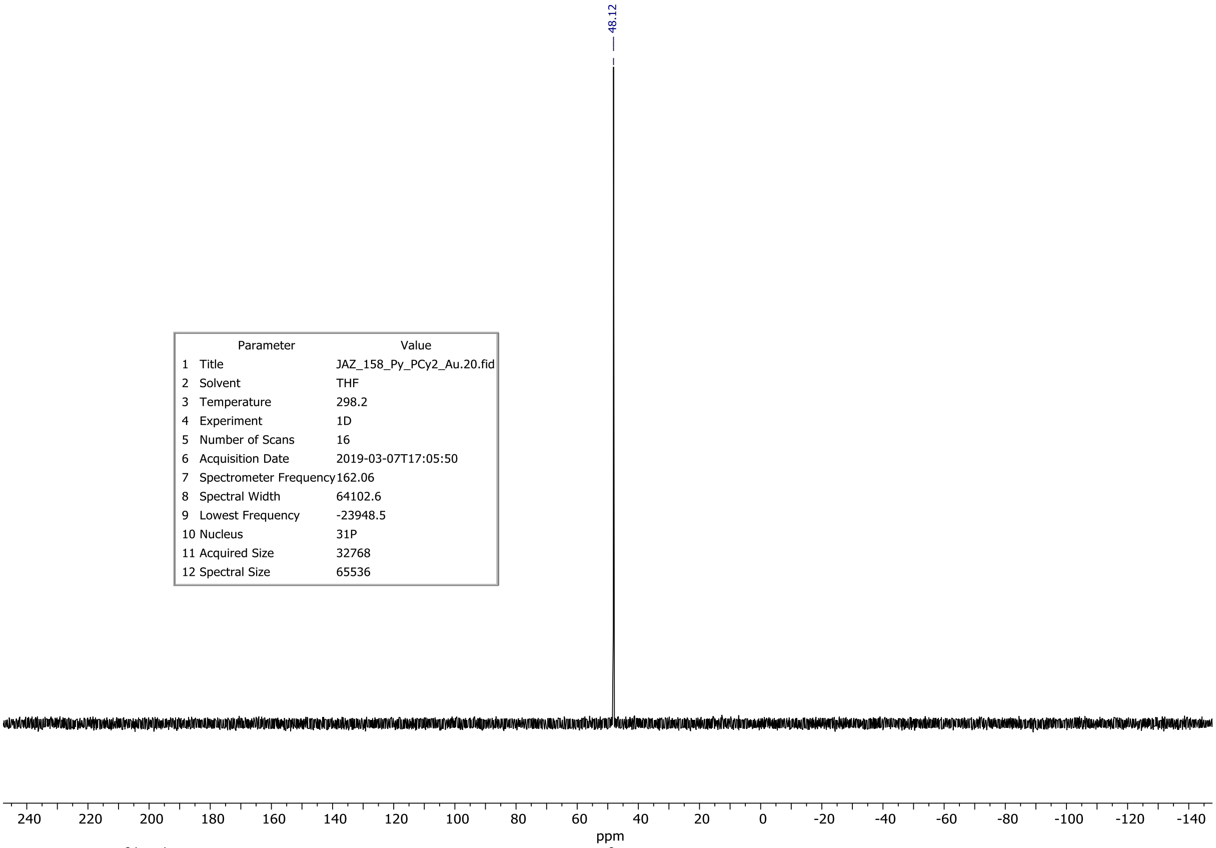

Figure S54. $^{31}P\{^1H\}$ NMR spectrum of **[(3b-Cy)AuCl)]** in THF-d^8.

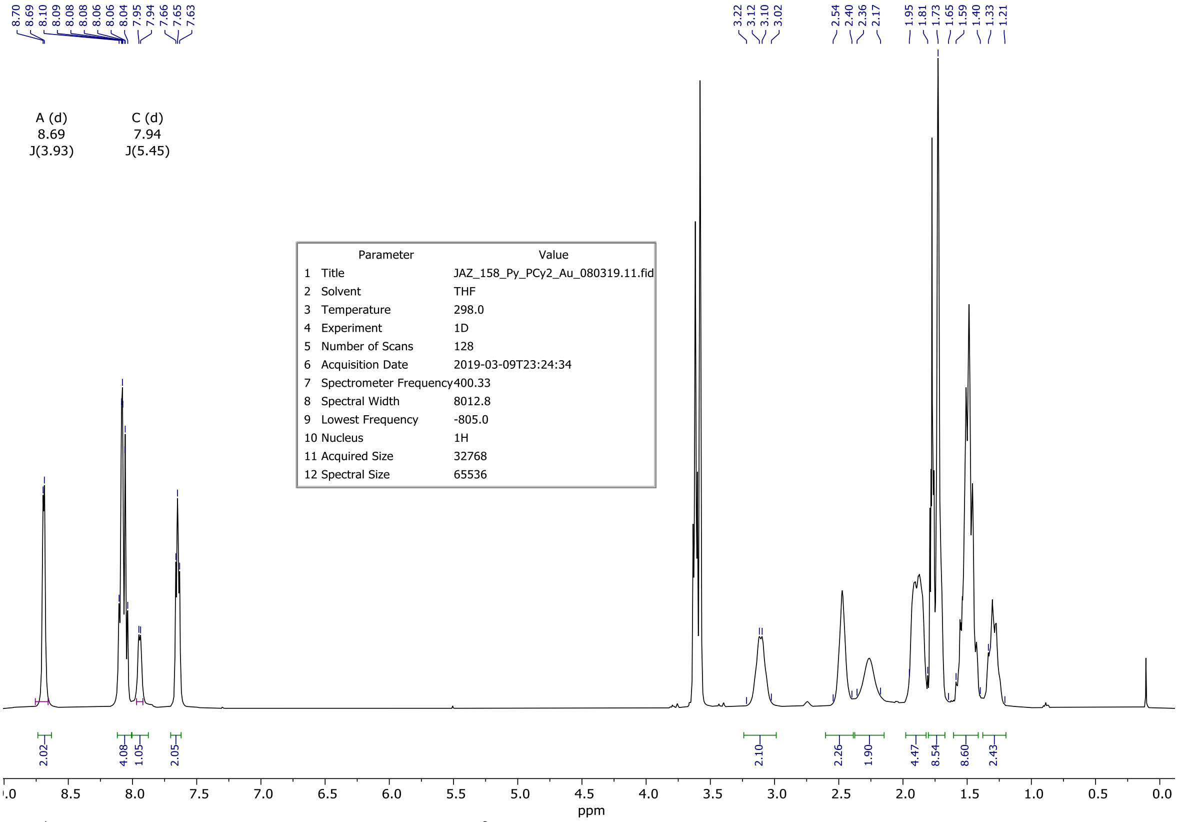

Figure S55. ^1H NMR spectrum of **[(3b-Cy)AuCl)]** in THF-d^8.

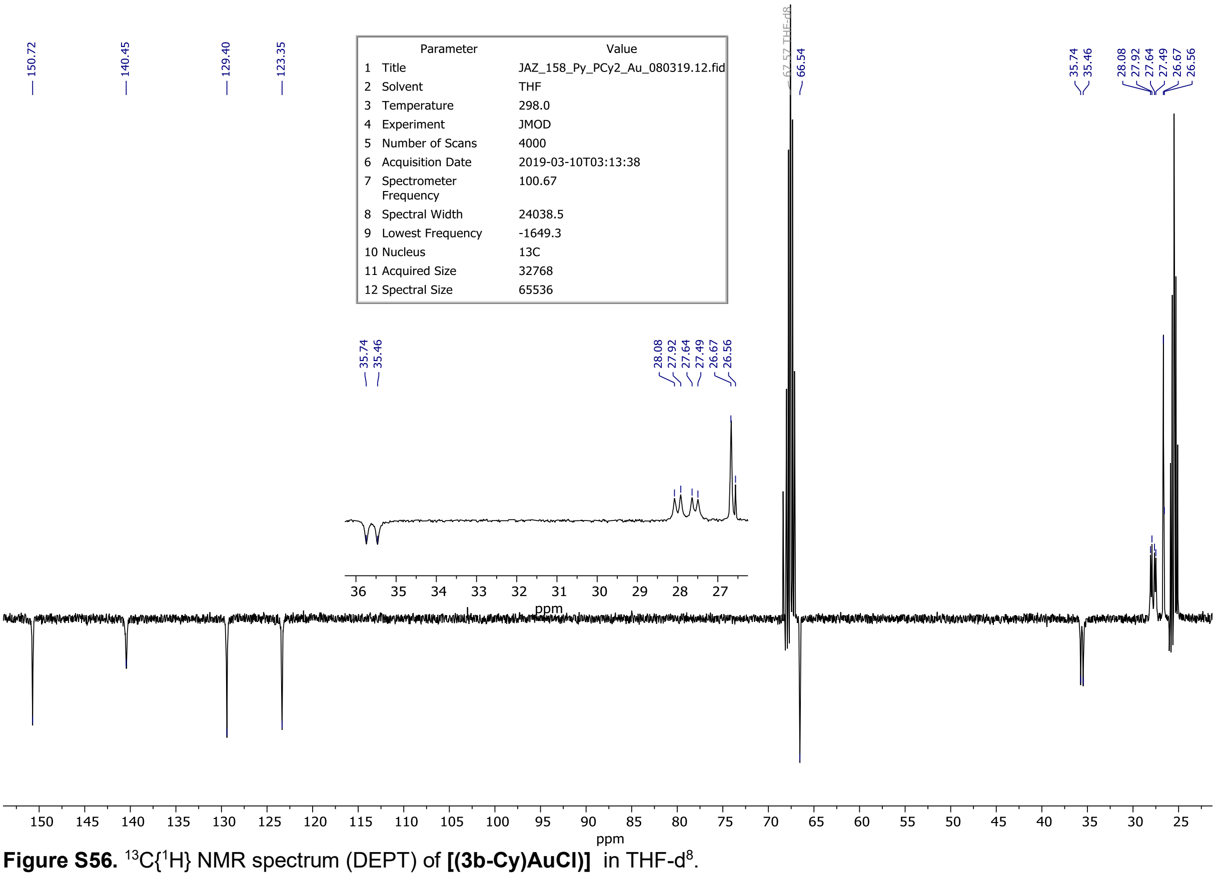

Figure S56. $^{13}C\{^1H\}$ NMR spectrum (DEPT) of **[(3b-Cy)AuCl)]** in THF-d^8.

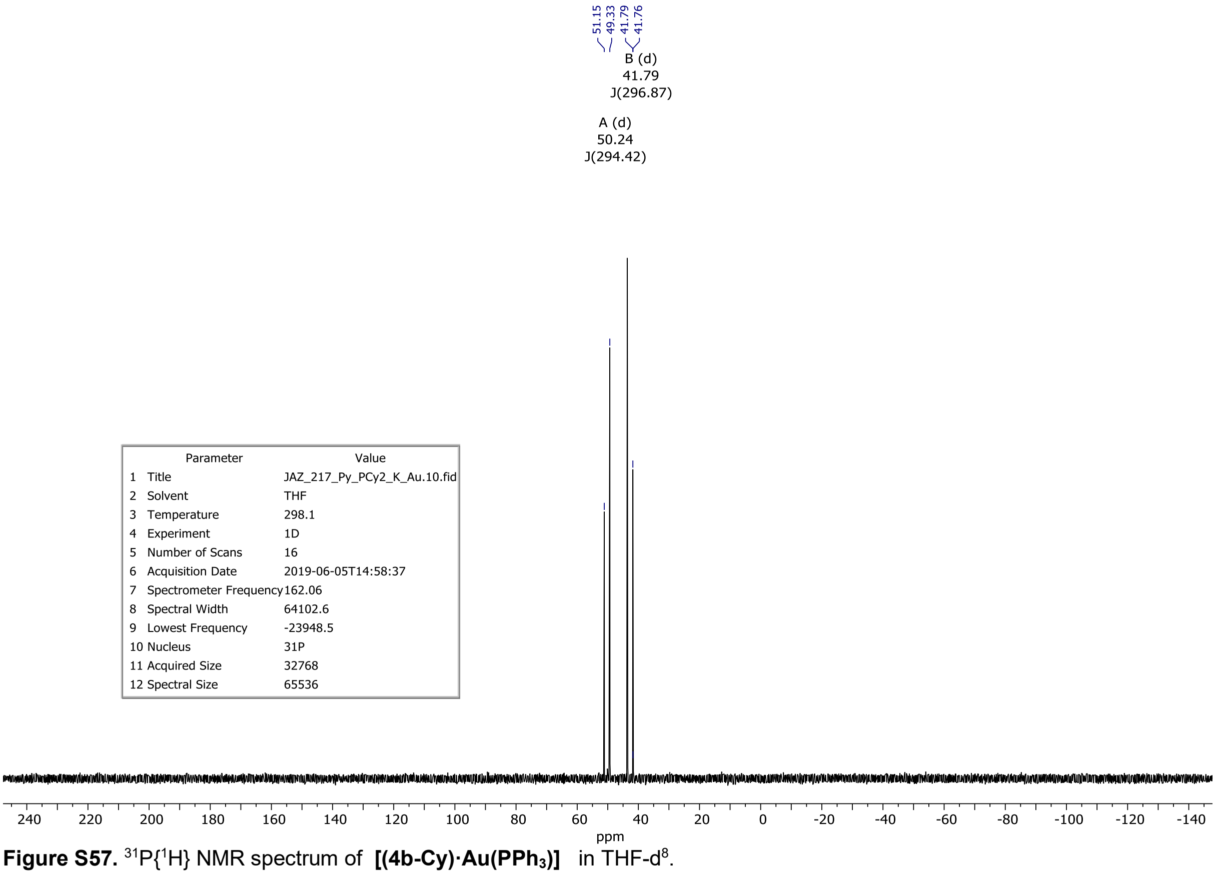

Figure S57. $^{31}P\{^1H\}$ NMR spectrum of **[(4b-Cy)·Au(PPh$_3$)]** in THF-d^8.

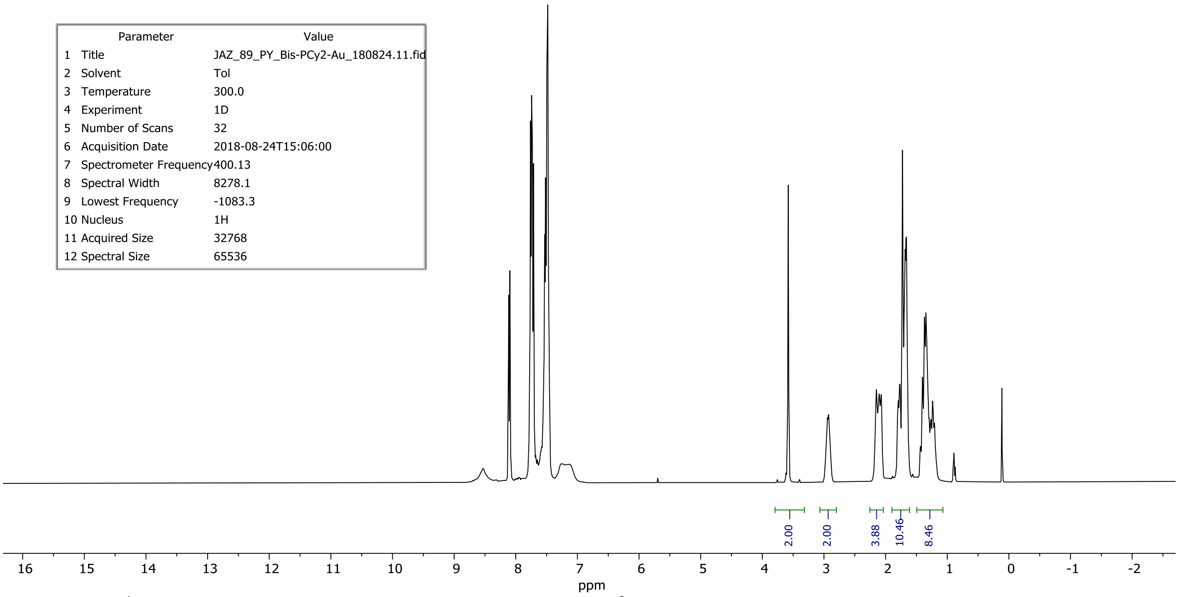

Figure S58. ^{1}H NMR spectrum of **[(4b-Cy)·Au(PPh$_3$)]** in THF-d^8.

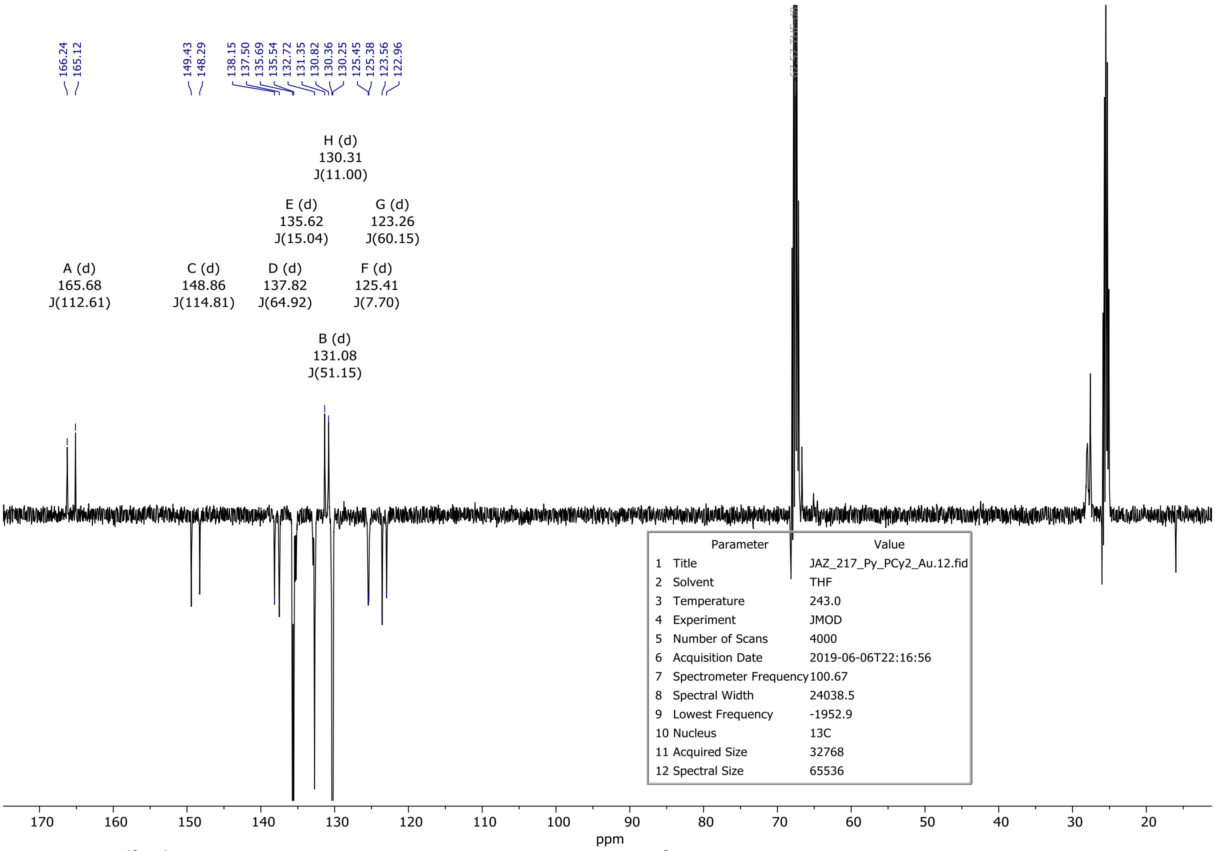

Figure S59. ^{13}C{^{1}H} NMR spectrum of **[(4b-Cy)·Au(PPh$_3$)]** in THF-d^8.

2.4 NMR spectra of the phosphine selenides for TEP determination

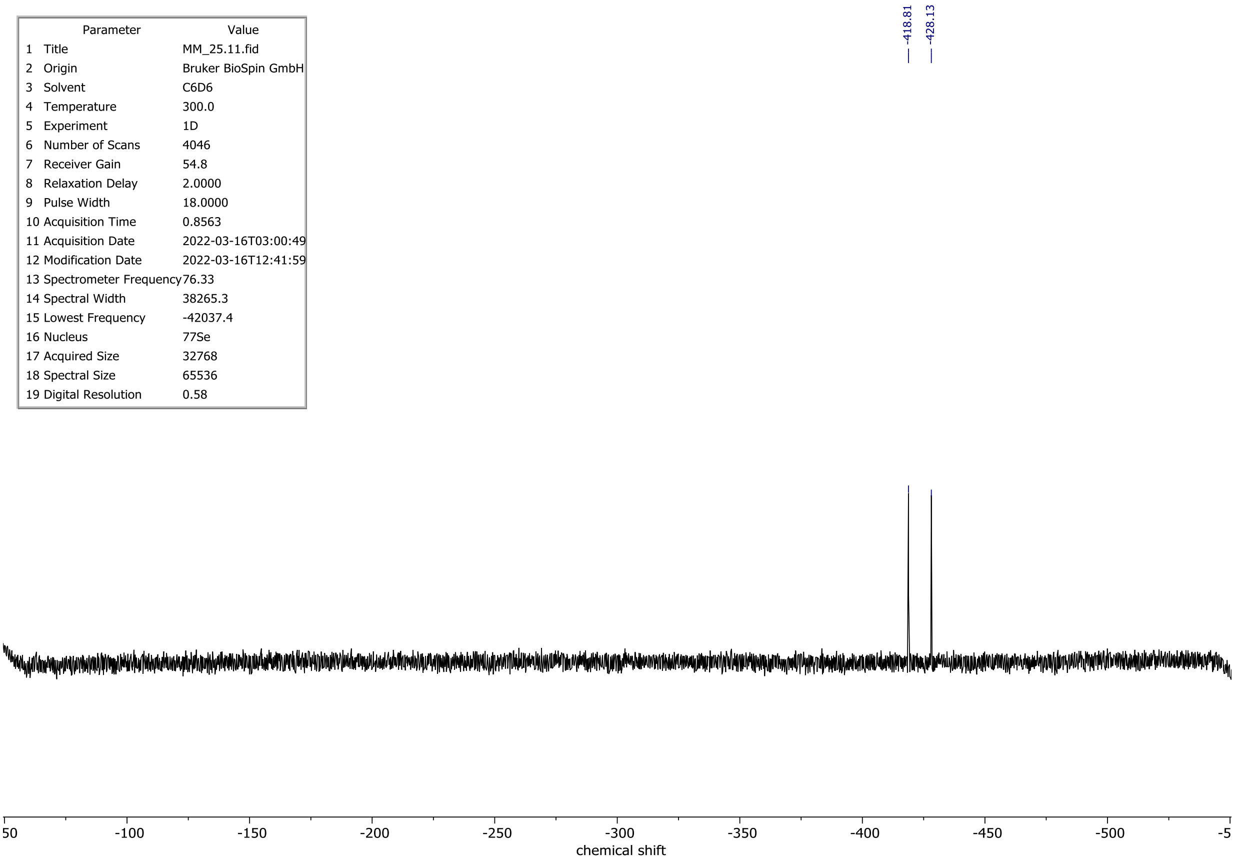

	Parameter	Value
1	Title	MM_25.11.fid
2	Origin	Bruker BioSpin GmbH
3	Solvent	C6D6
4	Temperature	300.0
5	Experiment	1D
6	Number of Scans	4046
7	Receiver Gain	54.8
8	Relaxation Delay	2.0000
9	Pulse Width	18.0000
10	Acquisition Time	0.8563
11	Acquisition Date	2022-03-16T03:00:49
12	Modification Date	2022-03-16T12:41:59
13	Spectrometer Frequency	76.33
14	Spectral Width	38265.3
15	Lowest Frequency	-42037.4
16	Nucleus	77Se
17	Acquired Size	32768
18	Spectral Size	65536
19	Digital Resolution	0.58

Figure S60. ^{77}Se{^{1}H} NMR spectrum of **tri-tert-butylphosphine selenide** in C_6D_6.

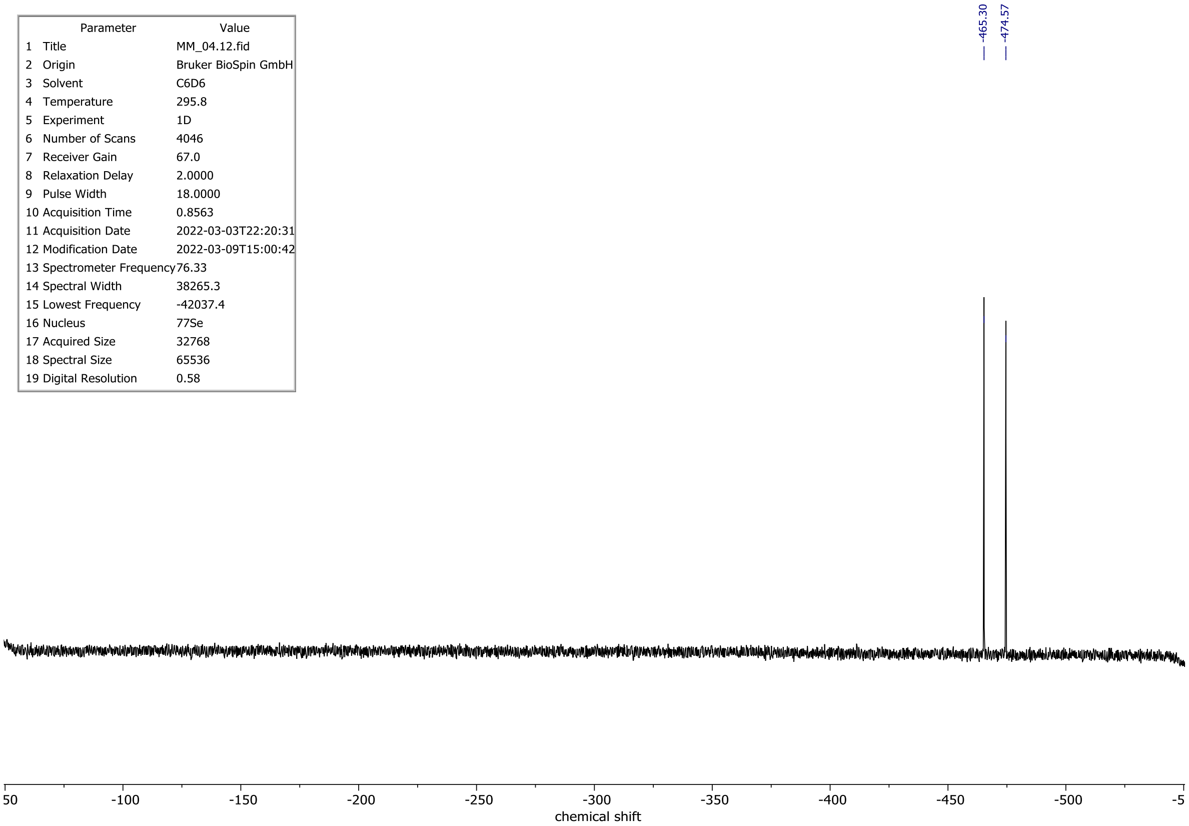

	Parameter	Value
1	Title	MM_04.12.fid
2	Origin	Bruker BioSpin GmbH
3	Solvent	C6D6
4	Temperature	295.8
5	Experiment	1D
6	Number of Scans	4046
7	Receiver Gain	67.0
8	Relaxation Delay	2.0000
9	Pulse Width	18.0000
10	Acquisition Time	0.8563
11	Acquisition Date	2022-03-03T22:20:31
12	Modification Date	2022-03-09T15:00:42
13	Spectrometer Frequency	76.33
14	Spectral Width	38265.3
15	Lowest Frequency	-42037.4
16	Nucleus	77Se
17	Acquired Size	32768
18	Spectral Size	65536
19	Digital Resolution	0.58

Figure S61. ^{77}Se{^{1}H} NMR spectrum of **tri-cyclohexylphosphine selenide** in C_6D_6.

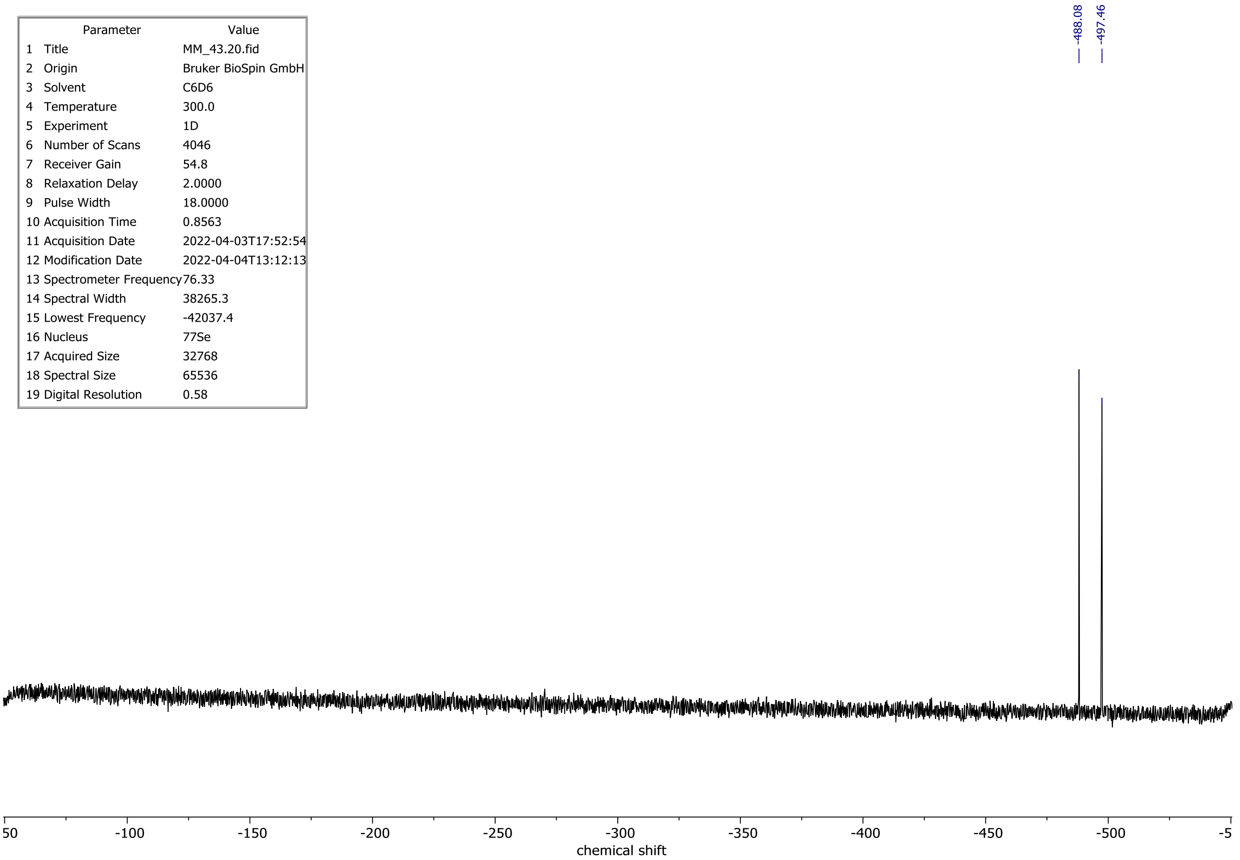

Figure S62. $^{77}Se\{^1H\}$ NMR spectrum of **tri-isopropylphosphine selenide** in C_6D_6.

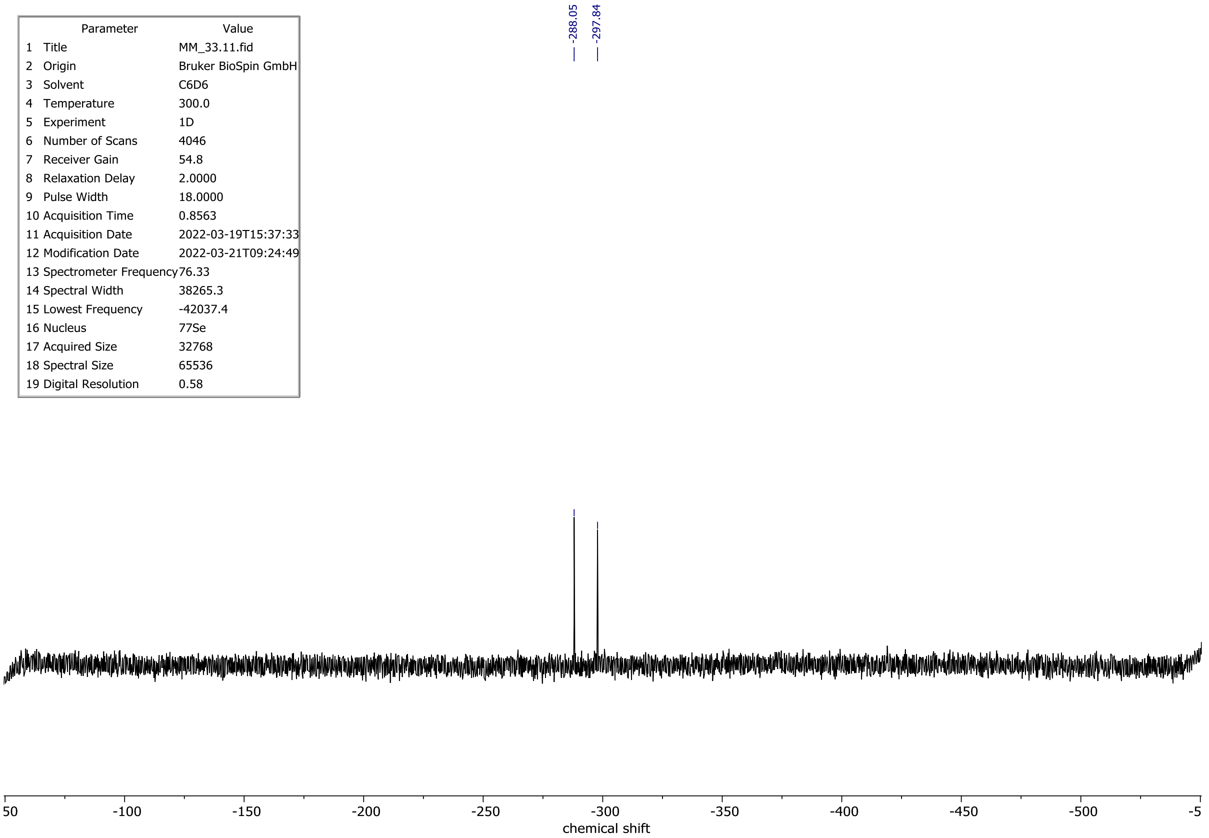

Figure S63. $^{77}Se\{^1H\}$ NMR spectrum of **methyldiphenylphosphine selenide** in C_6D_6.

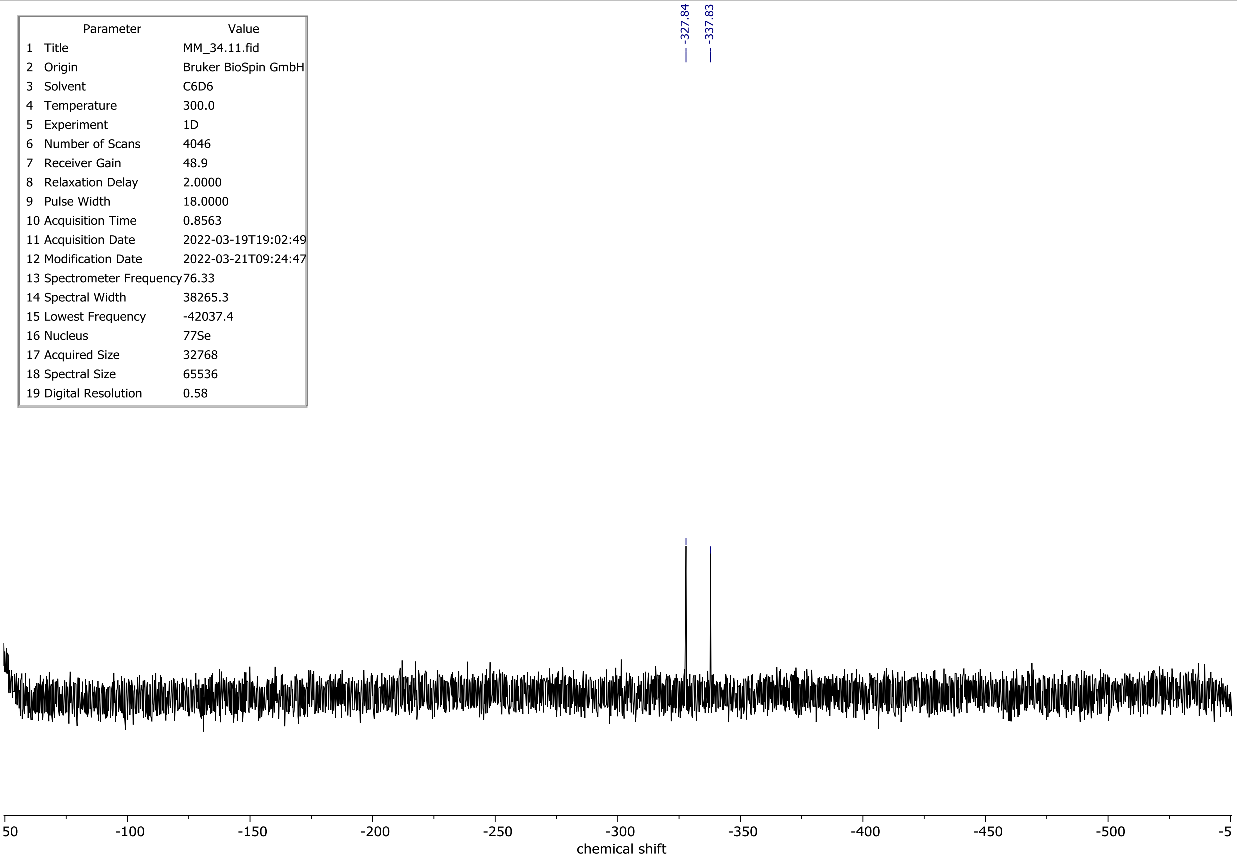

Figure S64. $^{77}Se\{^1H\}$ NMR spectrum of **benzyldiphenylphosphine selenide** in C_6D_6.

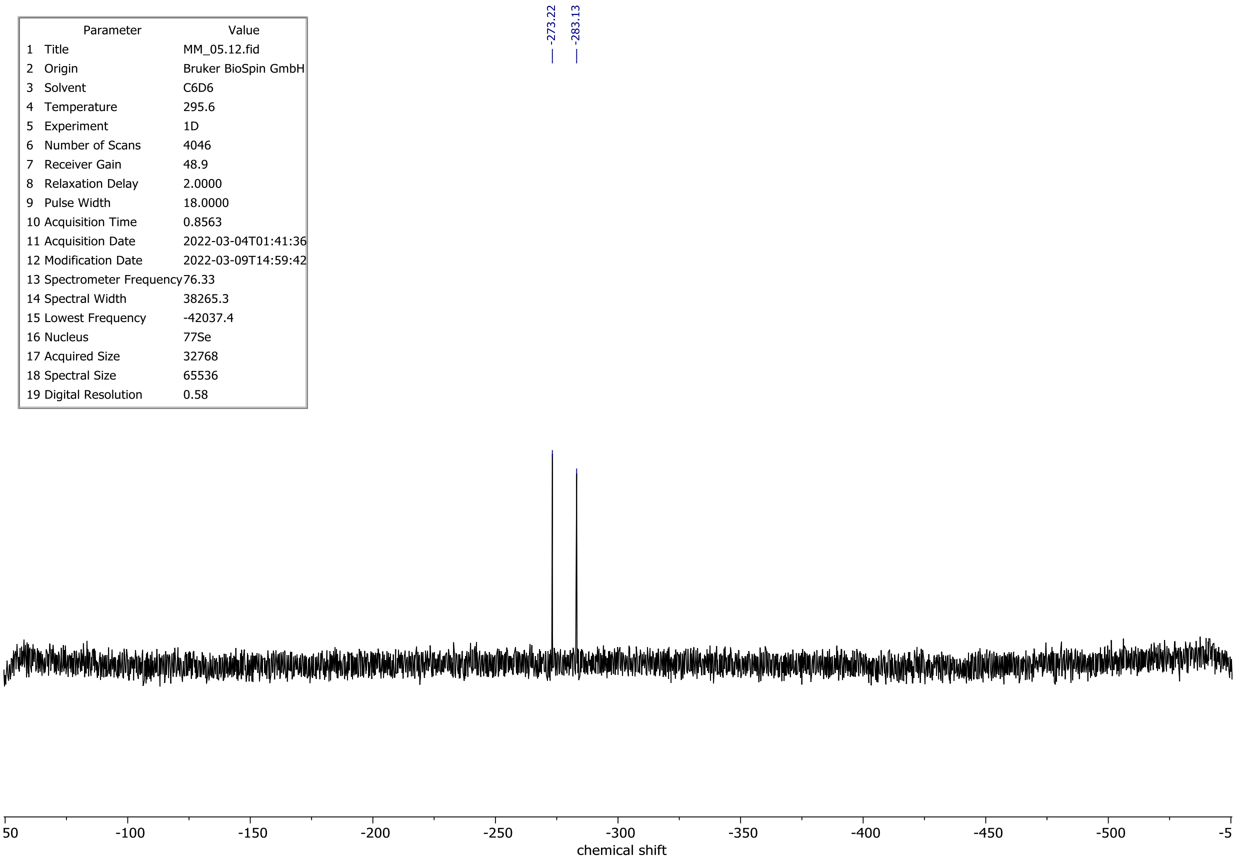

Figure S65. $^{77}Se\{^1H\}$ NMR spectrum of **triphenylphosphine selenide** in C_6D_6.

	Parameter	Value
1	Title	MM_13.20.fid
2	Origin	Bruker BioSpin GmbH
3	Solvent	C6D6
4	Temperature	296.1
5	Experiment	1D
6	Number of Scans	4046
7	Receiver Gain	54.8
8	Relaxation Delay	2.0000
9	Pulse Width	18.0000
10	Acquisition Time	0.8563
11	Acquisition Date	2022-03-05T11:21:28
12	Modification Date	2022-03-09T14:56:04
13	Spectrometer Frequency	76.33
14	Spectral Width	38265.3
15	Lowest Frequency	-42037.4
16	Nucleus	77Se
17	Acquired Size	32768
18	Spectral Size	65536
19	Digital Resolution	0.58

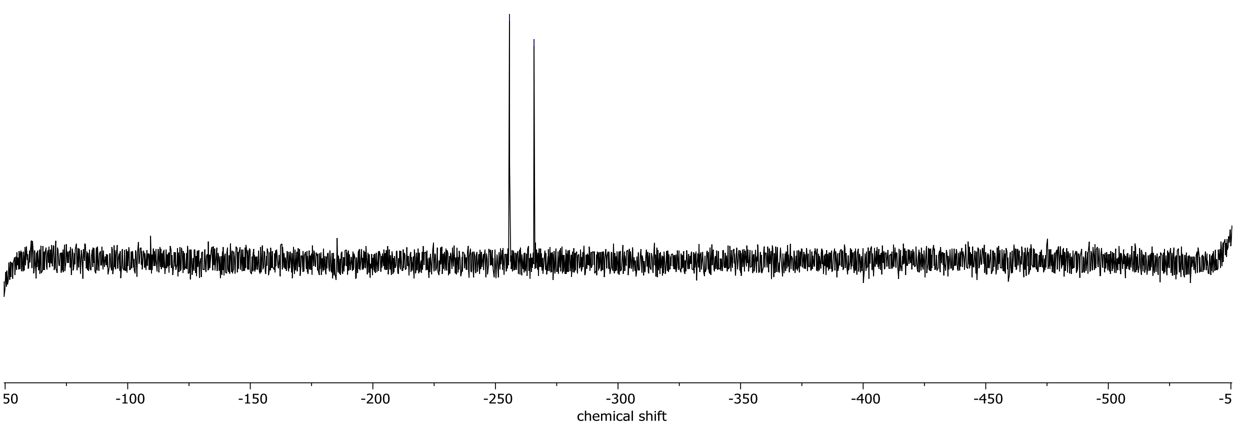

Figure S66. $^{77}Se\{^{1}H\}$ NMR spectrum of **tris(4-fluorophenyl)phosphine selenide** in C_6D_6.

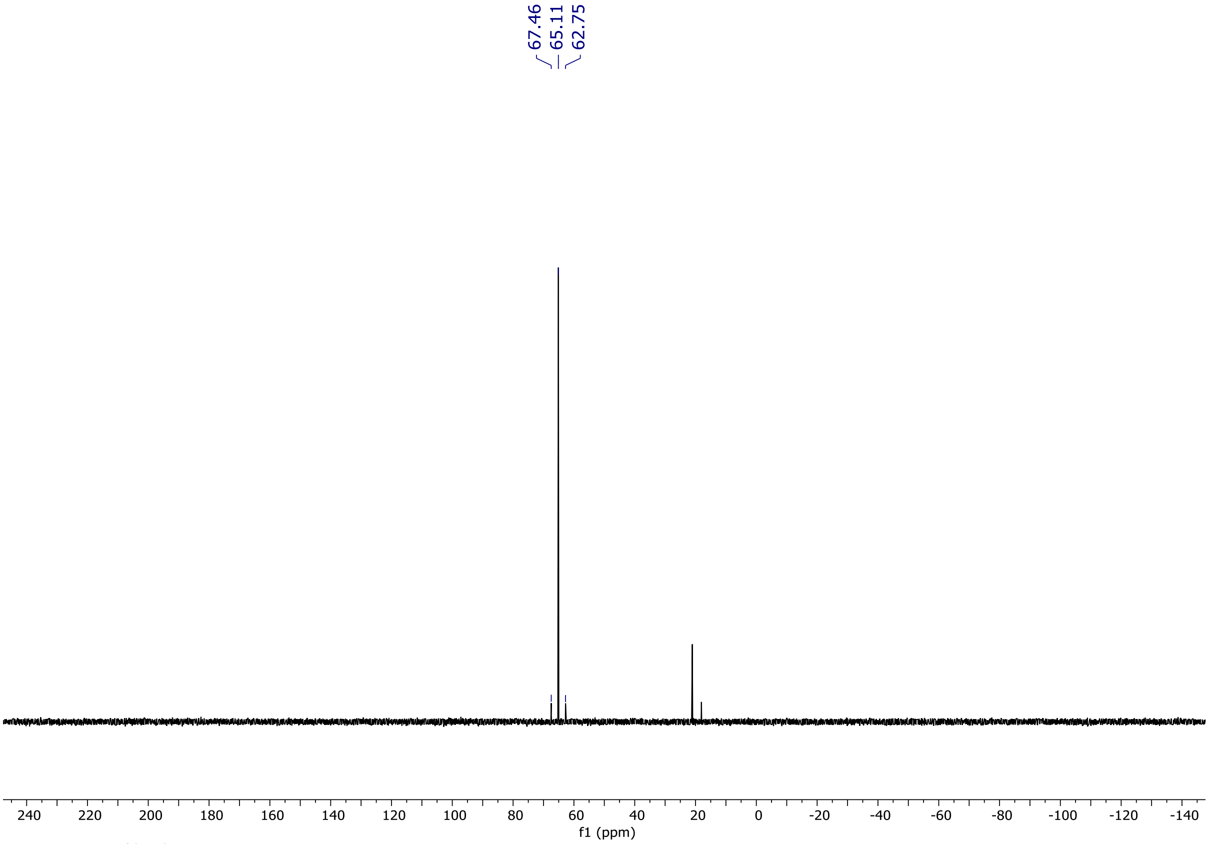

Figure S67. $^{31}P\{^{1}H\}$ NMR spectrum of the reaction mixture of **3a-Cy** with Se in C_6D_6

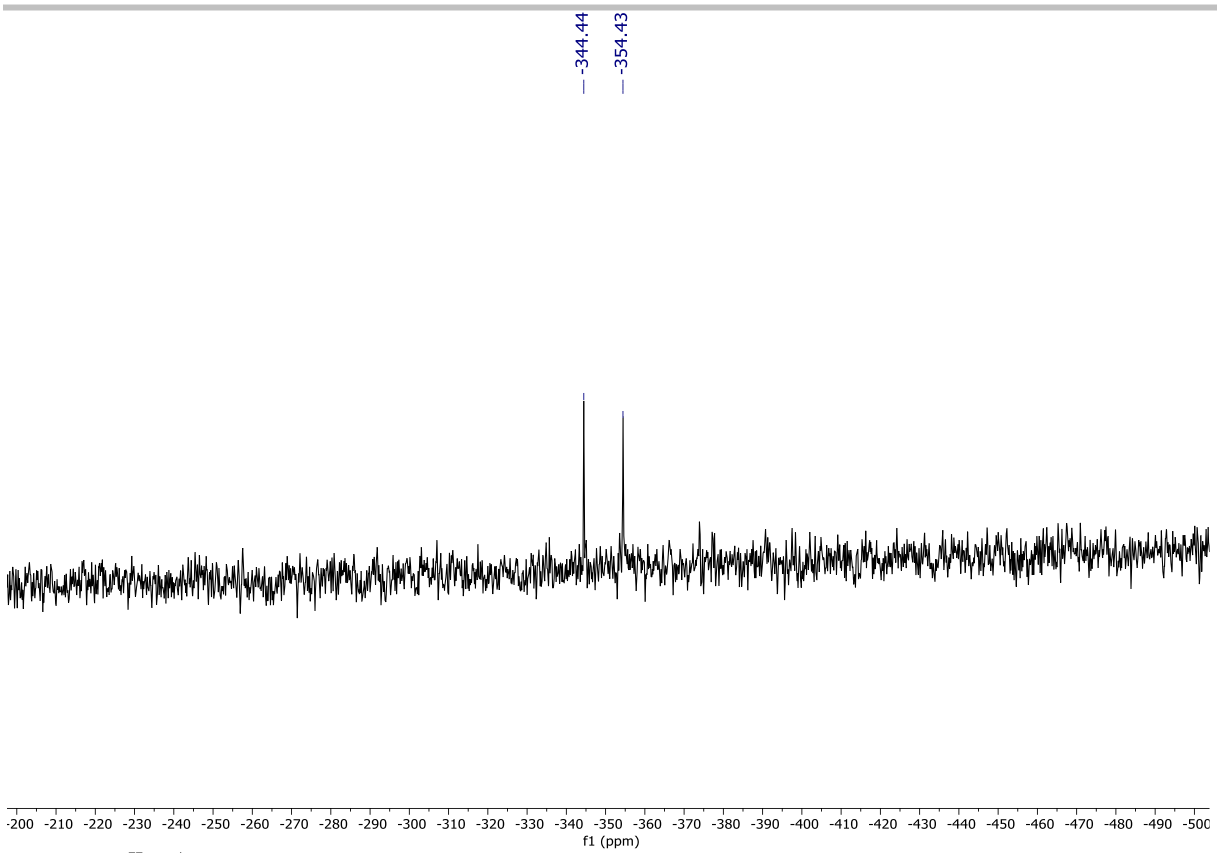

Figure S68. $^{77}Se\{^{1}H\}$ NMR spectrum of the reaction mixture of **3a-Cy** with Se in C_6D_6

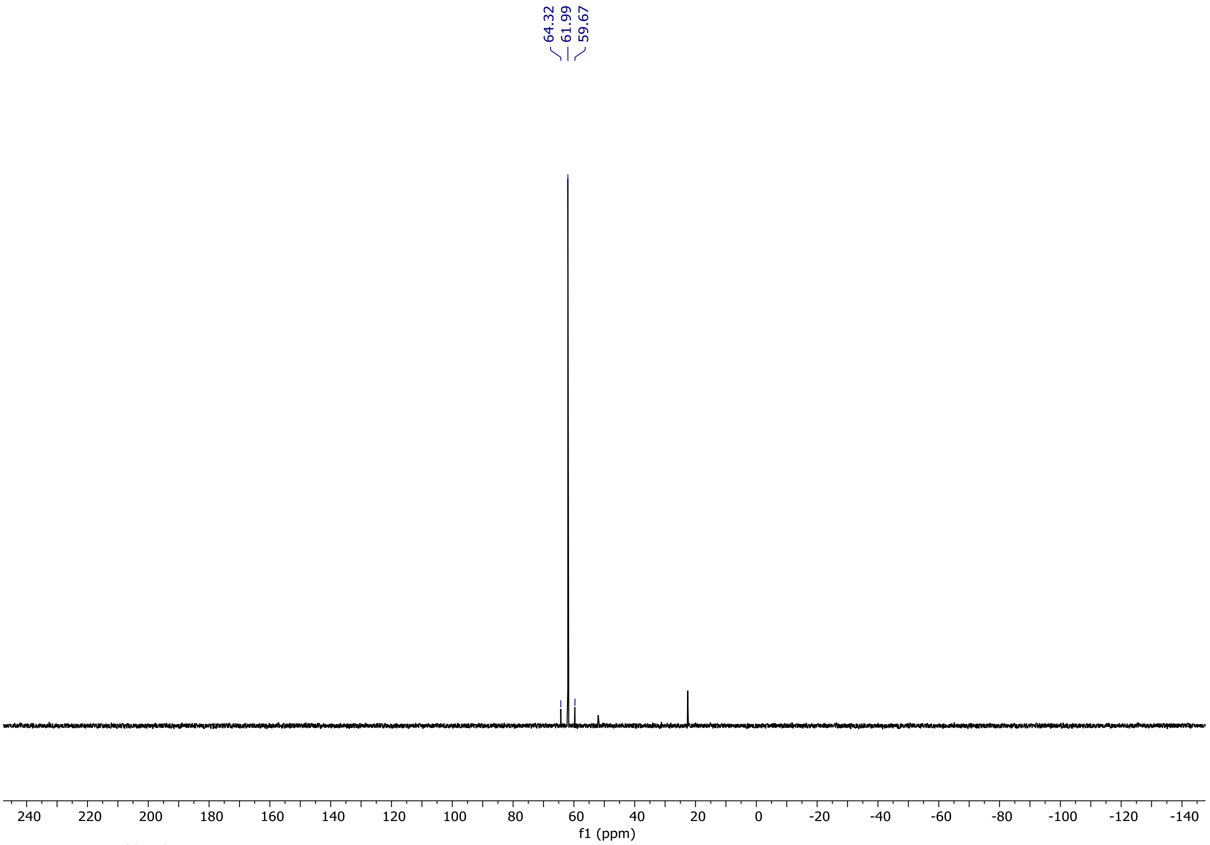

Figure S69. $^{31}P\{^{1}H\}$ NMR spectrum of the reaction mixture of **3b-Cy** with Se in C_6D_6

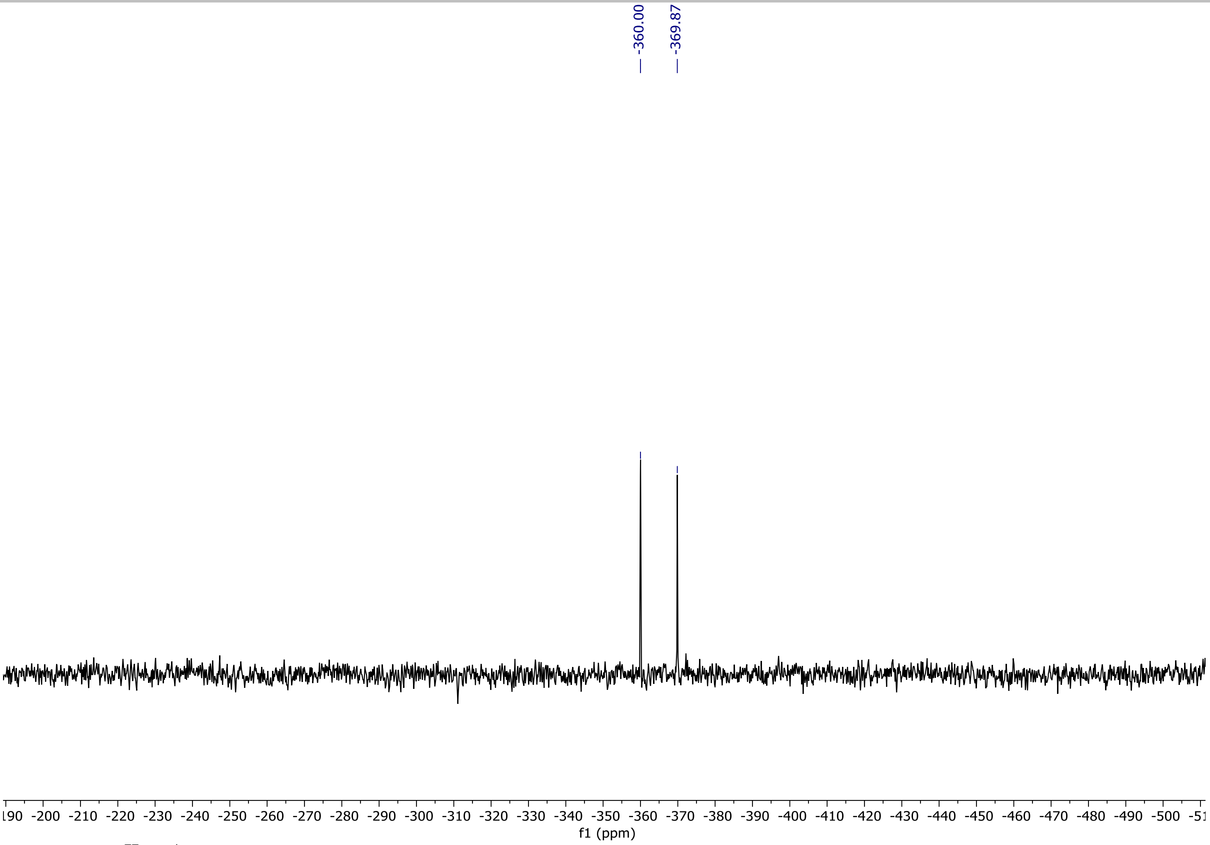

Figure S70. $^{77}Se\{^1H\}$ NMR spectrum of the reaction mixture of **3b-Cy** with Se in C_6D_6

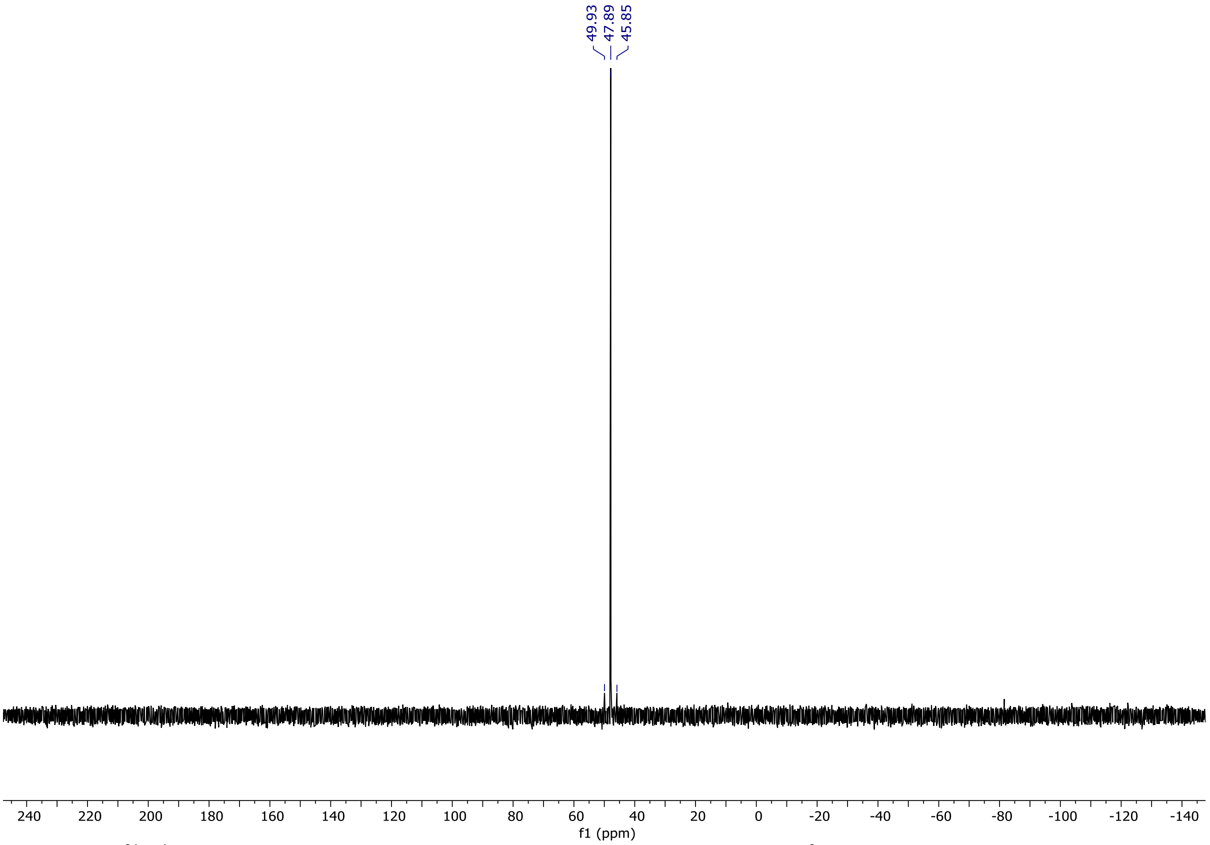

Figure S71. $^{31}P\{^1H\}$ NMR spectrum of the reaction mixture of **4a-Cy** with Se in THF-d[8]

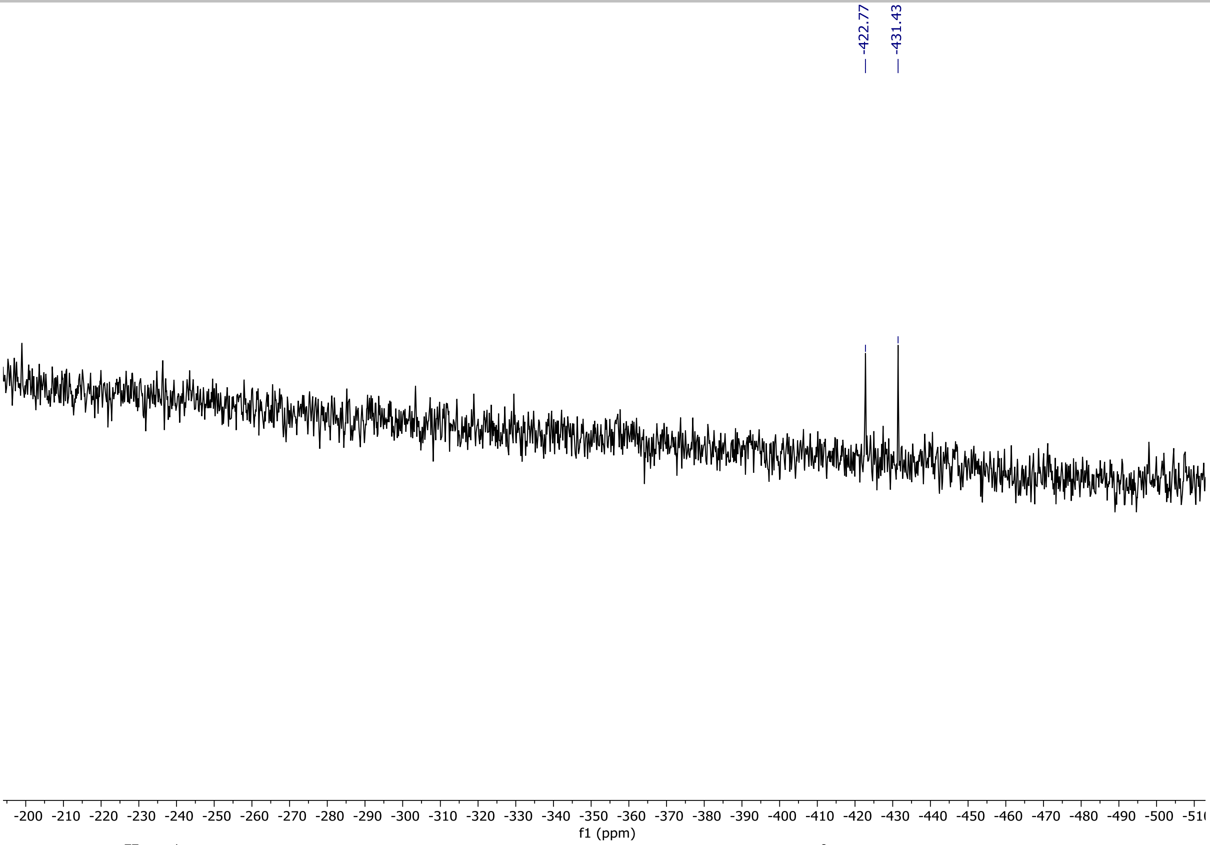

Figure S72. $^{77}Se\{^{1}H\}$ NMR spectrum of the reaction mixture of **4a-Cy** with Se in THF-d8

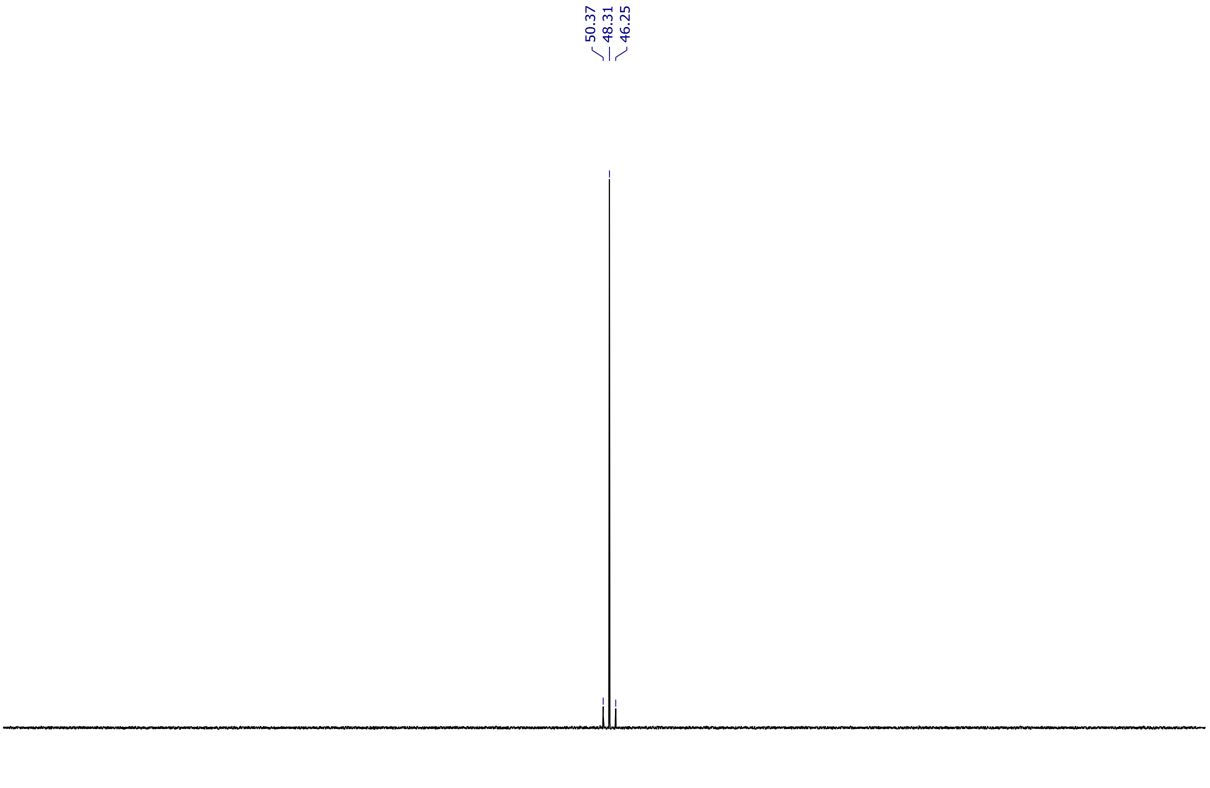

Figure S73. $^{31}P\{^{1}H\}$ NMR spectrum of the reaction mixture of **4b-Cy** with Se in THF-d8

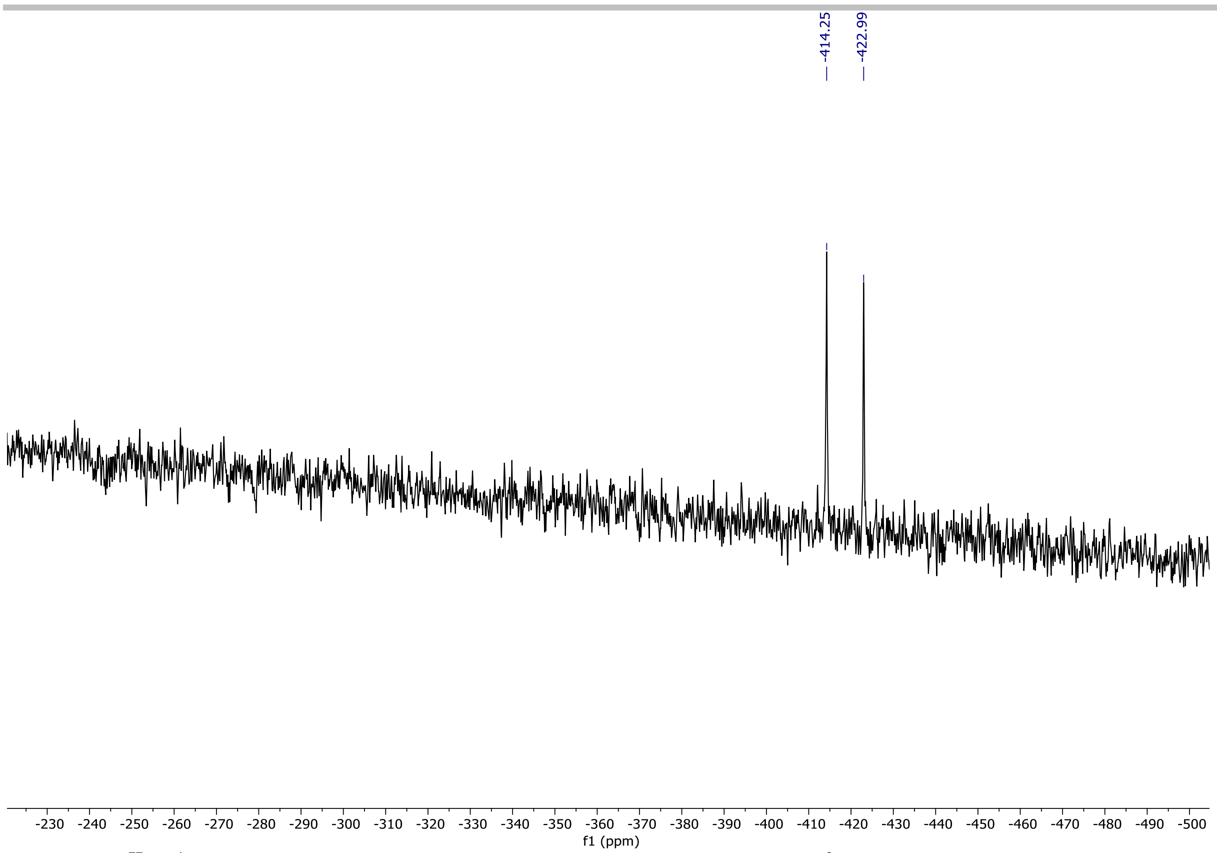

Figure S74. ^{77}Se{^{1}H} NMR spectrum of the reaction mixture of **4b-Cy** with Se in THF-d^{8}

3. Crystal Data

Data collection of all compounds was conducted with a Bruker APEX-CCD or Rigaku Synergy. The structures were solved using dual space FT and direct methods, refined with the Shelx software package and expanded using Fourier techniques.[5,6,7,8] The crystals of all compounds were mounted in an inert oil (perfluoropolyalkylether). Crystal structure determinations were measured at 100 K. Crystallographic data (excluding structure factors) have been deposited with the Cambridge Crystallographic Data Centre as supplementary publication Numbers 2154851-2154864 as well as 2169766 and 2169767 for the gold complexes. Copies of the data can be gained free of charge on application to Cambridge Crystallographic Data Centre, 12 Union Road, Cambridge CB2 1EZ, UK; [fax: (+44) 1223-336- 033; email: deposit@ccdc.cam.ac.uk].

3.1 Crystal data and structure refinement details for all compounds

Table S4. Crystal data and structure refinement for **1a**,**1b** and **2a.**

Compound	**1a**	**1b**	**2a**
CCDC-Number	2154853	2154851	2154854
Empirical formula	C_{15} H_{16} O_4 S_2	C_{11} H_{10} N_2 O_4 S_2	$C_{25}H_{35}NaO_9S_2$
Formula weight [g/mol]	324.40	298.33	566.64
Temperature [K]	100(2)	100(2)	100(2)
Wavelength [Å]	1.54178	0.71073	1.54184
Crystal system	Monoclinic	Monoclinic	Triclinic
Space group	*C*2/c	*C*2	P1
Unit cell dimensions			
a (Å)	15.5871(7)	15.6736(9)	7.90900(10)

b (Å)	11.6942(5)	5.3657(3)	11.24370(10)
c (Å)	8.1780(3)	7.5242(4)	15.98510(10)
α (°)	90	90	96.9940(10)
β (°)	91.832(4)	108.3370(10)	102.4240(10)
γ (°)	90	90	91.5210(10)
Volume [Å^3]	1489.90(11)	600.65(6)	1375.84(2)
Z	4	2	2
Density (calculated) [Mg/m^3]	1.446	1.649	1.36
Absorption coefficient [mm^{-1}]	3.360	0.455	2.338
F(000)	680	308	600
Crystal size [mm^3]	0.155 x 0.147 x 0.074	0.360 x 0.230 x 0.160	0.120 x 0.080 x 0.040
θ range [°]	4.728 to 74.461°	2.738 to 24.991	2.855 to 67.073
Index ranges	-19 ≤ h ≤19<br>-14 ≤ k ≤ 12<br>-9 ≤ l ≤ 1	-18 ≤ h ≤ 18<br>-6 ≤ k ≤ 6<br>-8 ≤ l ≤ 8	-9 ≤ h ≤ 9<br>-13 ≤ k ≤ 13<br>-19 ≤ l ≤ 19
Reflections collected	5852	3472	76073
Independent reflections	1524 [*R*(int) = 0.0244]	1052 [*R*(int) = 0.0133]	9587 [*R*(int) = 0.0375]
Data / restraints / parameters	1524/0/97	1052 / 1 / 87	9587 / 3 / 698
Goodness-of-fit on F^2	1.069	1.105	1.034
Final R indices [I>2σ(I)]	*R*1 = 0.0316, w*R*2 = 0.0905	*R*1 = 0.0166, w*R*2 = 0.0439	*R*1 = 0.0244, w*R*2 = 0.0628
R indices (all data)	*R*1 = 0.0337, w*R*2 = 0.0931	*R*1 = 0.0168, w*R*2 = 0.0441	*R*1 = 0.0253, w*R*2 = 0.0631
Largest diff. peak and hole [e.Å^{-3}]	0.312 and -0.424	0.211 and -0.150	0.357 and -0.251 e.Å^{-3}

Table S5. Crystal data and structure refinement for **2b**, **3a-PCy** and **4a-Cy**.

Compound	**2b**	**3a-Cy**	**4a-Cy**
CCDC-Number	2154852	2154855	2154859
Empirical formula	$C_{21}H_{29}N_2NaO_9S_2$	$C_{27}H_{37}O_4PS_2$	$C_{28}H_{42}Na_1O_5P_1S_2$
Formula weight [g/mol]	540.57	520.65	549.47
Temperature [K]	100(2)	99.9(2)	100(2)
Wavelength [Å]	0.71073	1.54178	0.71073
Crystal system	Monoclinic	Monoclinic	Monoclininc
Space group	*C*2/c	*P*2$_1$/n	*P*21/c
Unit cell dimensions			
a (Å)	28.7858(17)	11.0689(15)	12.6701(3)
b (Å)	9.9519(6)	11.8156(6)	14.4364(2)
c (Å)	18.6156(11)	20.5881(14)	19.2951(4)
α (°)	90	90	90
β (°)	113.5476(16)	103.469(9)	104.570(2)
γ (°)	90	90	90
Volume [Å^3]	4888.8(5)	2618(4)	3415.78(12)
Z	8	4	5
Density (calculated)[Mg/m^3]	1.469	1.321	1.336

Absorption coefficient [mm^{-1}]	0.290	2.671	0.260
F(000)	2272	1112	1472
Crystal size [mm^3]	0.350 x 0.320 x 0.150	0.155 x 0.147 x 0.074	0.204 x 0.120 x 0.036
θ range [°]	1.543 to 24.998	4.728 to 74.461	2.772 to 27.997
Index ranges	-34 ≤ h ≤ 34<br>-11 ≤ k ≤ 11<br>-22 ≤ l ≤ 22	-12 ≤ h ≤ 13<br>-14 ≤ k ≤ 14<br>-25 ≤ l ≤ 21	-15 ≤ h ≤ 15<br>-17 ≤ k ≤ 17<br>-22 ≤ l ≤ 2
Reflections collected	37310	27087	12944
Independent reflections	4300 [*R*(int) = 0.0217]	5363 [*R*(int)=0.0247]	12225 [R(int) = 0.0287]
Data / restraints / parameters	4300 / 0 / 339	5363/0/418	12944/0/409
Goodness-of-fit on F^2	1.024	1.070	1.069
Final R indices [I>2σ(I)]	*R*1 = 0.0281,<br>w*R*2 = 0.0702	*R*1 = 0.0288,<br>w*R*2 = 0.0757	*R*1 = 0.0770,<br>w*R*2 = 0.1800
R indices (all data)	*R*1 = 0.0304,<br>w*R*2 = 0.0719	*R*1 = 0.0309,<br>w*R*2 = 0.0774	*R*1 = 0.0961,<br>w*R*2 = 0.1892
Largest diff. peak and hole [e.Å^{-3}]	0.445 and -0.394	0.336 and -0.46	0.899 and -0.450

Table S6. Crystal data and structure refinement for **3a-iPr**, **3a-Ph** and **3b-Cy**.

Compound	**3a-iPr**	**3a-Ph**	**3b-Cy**
CCDC-Number	2154856	2154857	2154858
Empirical formula	$C_{21} H_{29} O_4 P S_2$	$C_{27} H_{25} O_4 P S_2$	$C_{27} H_{41} N_2 O_4 P S_2$
Formula weight [g/mol]	440.53	508.56	566.70
Temperature [K]	100(2) K	100(2)	100.00(10)
Wavelength [Å]	1.54184 Å	1.54184	0.71073
Crystal system	Monoclinic	Monoclinic	Triclinic
Space group	$P2_1/n$	P 21/n	*P*-1
Unit cell dimensions			
a (Å)	9.8069(2)	13.4340(3)	11.3732(3)
b (Å)	16.2526(3)	9.3182(2)	11.6904(2)
c (Å)	13.8974(3)	18.9262(5)	12.9393(7)
α (°)	90	90	84.4544(18)
β (°)	95.198(2)	93.188(2)	65.7713(3)
γ (°)	90	90	70.783(2)
Volume [$Å^3$]	2205.96(8)	2365.53(10)	1374.95(8
Z	4	4	2
Density (calculated)[Mg/m^3]	1.326	1.428	1.369
Absorption coefficient [mm^{-1}]	3.072	2.956	2.340
F(000)	936	1064	600
Crystal size [mm^3]	0.259 x 0.057 x 0.038	0.162 × 0.131 × 0.092	0.269 x 0.134 x 0.049
θ range [°]	4.196 to 77.856	3.934 to 77.545	3.966 to 76.099
Index ranges	-12 ≤ h ≤ 12<br>-19 ≤ k ≤ 20<br>-17 ≤ l ≤ 16	−16 ≤ h ≤ 16<br>−11 ≤ k ≤ 11<br>−23 ≤ l ≤ 17	-9 ≤ h ≤ 9<br>-14 ≤ k ≤ 13<br>-19 ≤ l ≤ 19
Reflections collected	51921	17028	12389
Independent reflections	4680 [R(int) = 0.0987]	4879 [R(int) = 0.0227]	7381 [*R*(int) = 0.0283]
Data / restraints / parameters	4680 / 0 / 263	4879 / 0 / 309	7381/3/688
Goodness-of-fit on F^2	1.076	1.073	1.045
Final R indices [I>2σ(I)]	*R*1 = 0.0555, wR2 = 0.1433	R_1 = 0.0380, wR_2 = 0.1102	*R*1 = 0.0309, w*R*2 = 0.0782
R indices (all data)	*R*1 = 0.0598, w*R*2 = 0.1465	R_1 = 0.0385, wR_2 = 0.1107	*R*1 = 0.0335, w*R*2 = 0.0800
Largest diff. peak and hole [e.$Å^{-3}$]	0.627 and -0.517	0.472 and −0.609	0.257 and -0.337

Table S7. Crystal data and structure refinement for **4a-Ph**, **5a** and **6a**.

Compound	**4a-Ph**	**5a**	**6a**
CCDC-Number	2154860	2154861	2154863
Empirical formula	$C_{62}H_{64}Na_2O_{10}P_2S_4$	$C_{28}H_{39}O_4PS_2$	C_{70} $H_{110}B_2Na_2O_{12}P_2S_4$
Formula weight [g/mol]	1205.29	534.68	1401.35
Temperature [K]	100(2)	100(2)	100(2)
Wavelength [Å]	1.54184	1.54184	1.54184
Crystal system	Triclinic	Monoclinic	Monoclinic
Space group	P-1	$P2_1/n$	$P2_1/c$
Unit cell dimensions			
a (Å)	12.49910(10)	12.33900(10)	13.37392(6)
b (Å)	15.04390(10)	15.0623(2)	20.13807(8)
c (Å)	15.86250(10)	14.9335(2)	27.48704(13)
α (°)	88.8710(10)	90	90
β (°)	88.2860(10)	102.5920(10)	98.9050(4)
γ (°)	85.8620(10)	90	90
Volume [Å^3]	2973.14(4)	2708.69(6)	7313.71(5)
Z	2	4	4
Density (calculated)[Mg/m^3]	1.346	1.311	1.273
Absorption coefficient [mm^{-1}]	2.595	2-596	2.189
F(000)	1264	1144	3008
Crystal size [mm^3]	0.162 × 0.128 × 0.085	0.092 × 0.087 × 0.053	0.273 × 0.184 ×0.152
θ range [°]	2.787 to 77.574	4.221 to 77.771	2.732 to 77.092
Index ranges	−15 ≤ h ≤ 15<br>−19 ≤ k ≤ 1,<br>−11 ≤ l ≤ 20	−15 ≤ h ≤ 15<br>−18 ≤ k ≤ 19<br>−14 ≤ l ≤ 18	−16 ≤ h ≤ 15<br>−25 ≤ k ≤ 23<br>−34 ≤ l ≤ 34
Reflections collected	40141	64871	100016
Independent reflections	12225 [*R*(int) = 0.0287]	5720 [*R*(int) = 0.0612]	15230 [*R*(int) = 0.0368]
Data / restraints / parameters	12225 / 0 / 725	5720 / 0 / 319	15230 / 0 / 863
Goodness-of-fit on F^2	1.062	1.091	1.040
Final R indices [I>2σ(I)]	R_1 = 0.0351,<br>wR_2 = 0.0938	R_1 = 0.0400,<br>wR_2 = 0.0992	R_1 = 0.0378,<br>wR_2 = 0.0994
R indices (all data)	R_1 = 0.0367,<br>wR_2 = 0.0947	R_1 = 0.0436,<br>wR_2 = 0.1014	R_1 = 0.0401,<br>wR_2 = 0.1010
Largest diff. peak and hole [e.Å^{-3}]	0.566 and −0.457	0.347 and −0.385	1.069 and −0.652

Table S8. Crystal data and structure refinement for **5b** and **6b**.

Compound	**6b**	**5b**
CCDC-Number	2154864	2154862
Empirical formula	$C_{35} H_{52} K N_2 O_{10} P S_3$	$C_{34}H_{45}N_2O_5PS_2$
Formula weight [g/mol]	827.03	656.81
Temperature [K]	100(2)	100(2)
Wavelength [Å]	1.54184	1.54184
Crystal system	Orthorhombic	Monoclinic
Space group	$Pna2_1$	$P2_1/c$
Unit cell dimensions		
a (Å)	20.62430(10)	14.92140(10)
b (Å)	12.14860(10)	10.49870(10)
c (Å)	32.5383(2)	21.2849(2)
α (°)	90	90
β (°)	90	98.4060(10)
γ (°)	90	90
Volume [$Å^3$]	8152.68(9)	3298.57(5)
Z	8	4
Density (calculated)[Mg/m^3]	1.348	1.323
Absorption coefficient [mm^{-1}]	3.411	2.276
F(000)	3504	1400
Crystal size [mm^3]	0.193 × 0.180 × 0.08	0.117 x 0.067 x 0.043
θ range [°]	3.884 to 77.297	2.994 to 74.996
Index ranges	−26 ≤ h ≤ 25<br>−15 ≤ k ≤ 14<br>−38 ≤ l ≤ 40	-16 ≤ h ≤ 18<br>-13 ≤ k ≤ 13<br>-26 ≤ l ≤ 15
Reflections collected	107636	25181
Independent reflections	16118 [*R*(int) = 0.0447]	6649 [*R*(int) = 0.0362]
Data / restraints / parameters	16118 / 1 / 938	6649 / 3 / 398
Goodness-of-fit on F^2	1.024	1.033
Final R indices [I>2σ(I)]	R_1 = 0.0423,<br>wR_2 = 0.1109	*R*1 = 0.0403,<br>w*R*2 = 0.1049
R indices (all data)	R_1 = 0.0438,<br>wR_2 = 0.1121	*R*1 = 0.0458,<br>w*R*2 = 0.1083
Largest diff. peak and hole [e.$Å^{-3}$]	1.097 and -0.455	0.643 d -0.500

Table S9. Crystal data and structure refinement for the gold complexes.

Compound	**[(3b-Cy)AuCl]**	**(bb-Cy)Au)PPh3)]** xTHF
CCDC-Number	2169766	2169767
Empirical formula	$C_{23}H_{31}AuN_2O_4PS_2$	$C_{45}H_{53}AuN_2O_5P_2S_2$
Formula weight [g/mol]	727.00	1024.92
Temperature [K]	100(2)	100(2)
Wavelength [Å]	0.71073	1.54184
Crystal system	triclinic	Monoclinic
Space group	$P\bar{1}$	$P2_1/c$
Unit cell dimensions		
a (Å)	11.3838(2)	12.9810(2)
b (Å)	11.7213(3)	14.0310(2)
c (Å)	12.1209(3)	27.2465(4)
α (°)	111.936(2)	90
β (°)	96.909(2)	90.8820(10)
γ (°)	109.050(2)	90
Volume [$Å^3$]	1362.89(6)	4961.99(13)
Z	2	4
Density (calculated)[Mg/m^3]	1.772	1.372
Absorption coefficient [mm^{-1}]	5.739	7.299
F(000)	716	2072
Crystal size [mm^3]	0.280 × 0.160 × 0.11	0.117 x 0.067 x 0.043
θ range [°]	3.071 to 30.824	3.244 to 67.080
Index ranges	−15 ≤ h ≤ 16<br>−15 ≤ k ≤ 16<br>−16 ≤ l ≤ 16	-15 ≤ h ≤ 15<br>-16 ≤ k ≤ 16<br>-32 ≤ l ≤ 30
Reflections collected	22591	32571
Independent reflections	6970 [*R*(int) = 0.0511]	8799 [*R*(int) = 0.0283]
Data / restraints / parameters	6970 / 0 / 322	8799 / 80 / 569
Goodness-of-fit on F^2	1.046	1.061
Final R indices [I>2σ(I)]	R_1 = 0.0267, wR_2 = 0.0587	*R*1 = 0.0223, w*R*2 = 0.0527
R indices (all data)	R_1 = 0.0319, wR_2 = 0.0603	*R*1 = 0.0234, w*R*2 = 0.0533
Largest diff. peak and hole [e.$Å^{-3}$]	0.965 and -1.983	0.514 and -0.537

3.2 Crystal Strucuture Determination of 1a and 1b

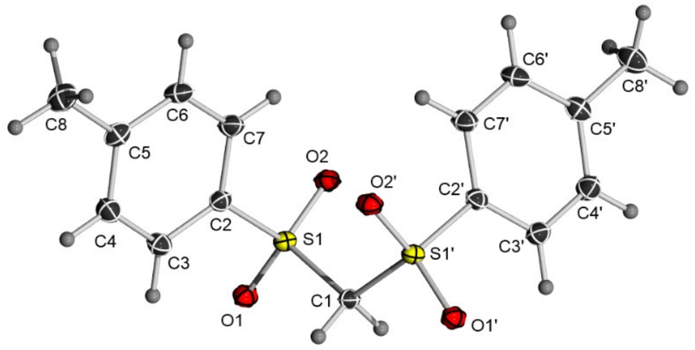

Figure S75. ORTEP plot of **1a**. Ellipsoids are drawn at 50 % probability level.

Table S10. Atomic coordinates (x 10^4) and equivalent isotropic displacement parameters ($Å^2$ x 10^3) for **1a**. U(eq) is defined as one third of the trace of the orthogonalized U^{ij} tensor.

	x	y	z	U(eq)
S(1)	4578(1)	3121(1)	762(1)	18(1)
O(1)	4286(1)	4012(1)	-341(1)	22(1)
C(1)	5000	3893(2)	2500	18(1)
C(2)	5454(1)	2403(1)	-76(2)	19(1)
C(3)	6016(1)	2986(1)	-1066(2)	23(1)
C(4)	6690(1)	2390(1)	-1738(2)	25(1)
C(5)	6804(1)	1223(1)	-1443(2)	23(1)
C(6)	6232(1)	662(1)	-442(2)	26(1)
C(7)	5561(1)	1244(1)	255(2)	24(1)
O(2)	3973(1)	2287(1)	1311(1)	23(1)
C(8)	7539(1)	575(1)	-2163(2)	31(1)

Table S11. Anisotropic displacement parameters ($Å^2$ x 10^3) for **1a**. The anisotropic displacement factor exponent takes the form: $-2\pi^2[h^2 a^{*2}U^{11} + ... + 2 h k a^* b^* U^{12}]$

	U^{11}	U^{22}	U^{33}	U^{23}	U^{13}	U^{12}
S(1)	19(1)	14(1)	20(1)	-1(1)	0(1)	0(1)
O(1)	25(1)	20(1)	22(1)	0(1)	-2(1)	2(1)
C(1)	22(1)	14(1)	18(1)	0	-1(1)	0
C(2)	22(1)	17(1)	19(1)	-3(1)	-1(1)	0(1)
C(3)	28(1)	19(1)	23(1)	2(1)	-1(1)	2(1)
C(4)	25(1)	25(1)	25(1)	3(1)	3(1)	0(1)
C(5)	20(1)	24(1)	25(1)	-5(1)	-3(1)	1(1)
C(6)	26(1)	15(1)	36(1)	-4(1)	1(1)	0(1)

C(7)	25(1)	17(1)	31(1)	-2(1)	5(1)	-3(1)
O(2)	22(1)	18(1)	28(1)	-1(1)	2(1)	-3(1)
C(8)	24(1)	31(1)	38(1)	-5(1)	4(1)	5(1)

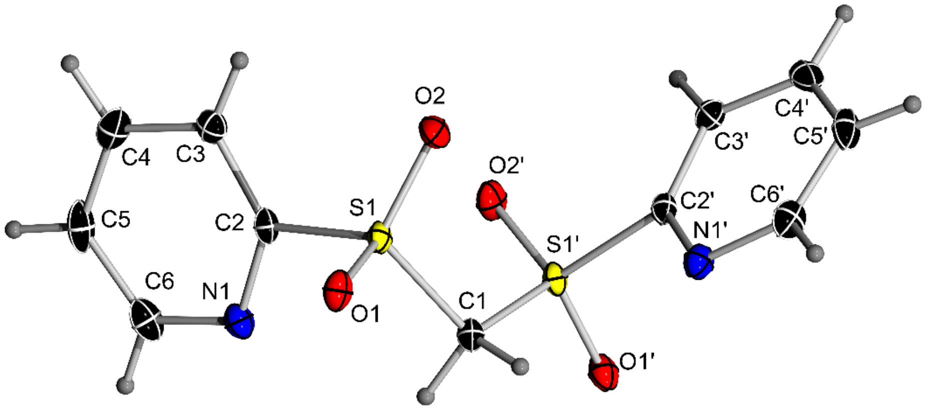

Figure S76. ORTEP plot of **1b**. Ellipsoids are drawn at 50 % probability level.

Table S12. Atomic coordinates (x 10^4) and equivalent isotropic displacement parameters (Å^2 x 10^3) for **1b**. U(eq) is defined as one third of the trace of the orthogonalized U^{ij} tensor.

	x	y	z	U(eq)
C(1)	10000	7663(6)	10000	14(1)
N(1)	11434(1)	8684(4)	8328(3)	16(1)
O(1)	9350(1)	7745(3)	6394(2)	17(1)
S(1)	9730(1)	9469(1)	7902(1)	12(1)
C(2)	10802(1)	10432(4)	7770(3)	13(1)
O(2)	9215(1)	11629(3)	8073(2)	18(1)
C(3)	10881(2)	12697(4)	6985(3)	15(1)
C(4)	11690(2)	13132(4)	6639(3)	20(1)
C(5)	12362(2)	11356(5)	7171(3)	21(1)
C(6)	12218(1)	9196(5)	8046(3)	19(1)

Table S13. Anisotropic displacement parameters (Å^{2}x 10^3) for **1b**. The anisotropic displacement factor exponent takes the form: $-2\pi^2[h^2 a^{*2}U^{11} + ... + 2 h k a^* b^* U^{12}]$

	U^{11}	U^{22}	U^{33}	U^{23}	U^{13}	U^{12}
C(1)	14(2)	17(2)	13(2)	0	7(1)	0
N(1)	13(1)	19(1)	16(1)	-2(1)	5(1)	0(1)
O(1)	13(1)	23(1)	14(1)	-1(1)	4(1)	-4(1)
S(1)	9(1)	16(1)	13(1)	1(1)	4(1)	1(1)
C(2)	11(1)	18(1)	10(1)	-3(1)	4(1)	-2(1)
O(2)	14(1)	21(1)	21(1)	4(1)	8(1)	5(1)
C(3)	17(1)	16(1)	12(1)	-2(1)	4(1)	-1(1)
C(4)	24(1)	20(1)	16(1)	-5(1)	8(1)	-11(1)

C(5)	14(1)	34(1)	18(1)	-9(1)	8(1)	-10(1)
C(6)	11(1)	26(1)	21(1)	-6(1)	4(1)	-1(1)

3.3 Crystal Structure Determination of 2a and 2b

Crystal structure analysis of 2a.

Additional information concerning the structure refinement: A disorded 15-crown-5 moiety (occupancy 0.7:0.3) was solved using the PART instructions. All hydrogen atoms were placed on ideal positions except for H1 on C1 and H26 on C26 which were found in the difference Fourier map and refined independently.

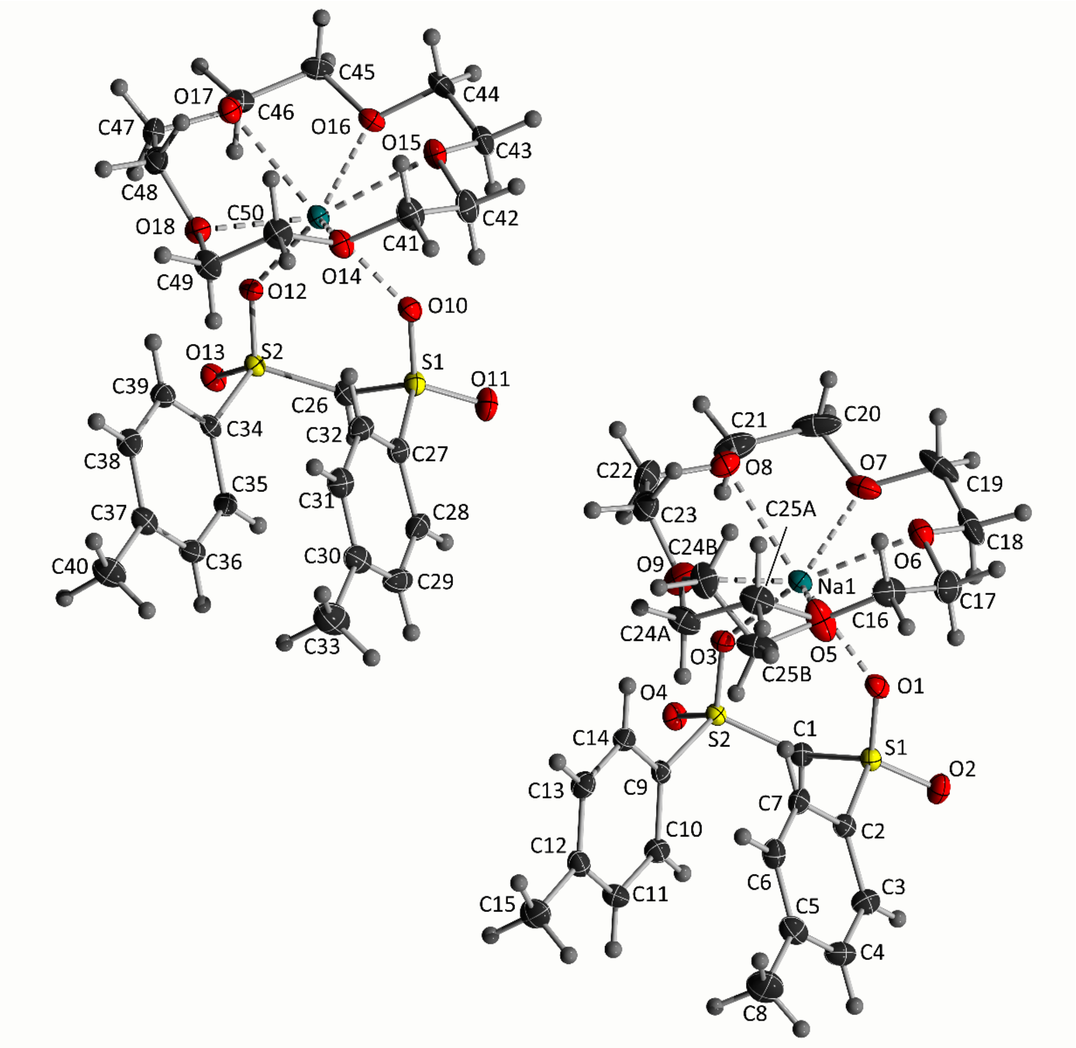

Figure S77. ORTEP plot of **2a**. Ellipsoids are drawn at 50 % probability level.

Table S14. Atomic coordinates (x 10^4) and equivalent isotropic displacement parameters (Å^2 x 10^3) for **2a**. U(eq) is defined as one third of the trace of the orthogonalized U^{ij} tensor.

	x	y	z	U(eq)
S(1)	-2330(1)	1472(1)	1540(1)	16(1)
Na(1)	155(1)	4276(1)	2388(1)	18(1)

O(1)	-1615(2)	2661(2)	1494(1)	20(1)
C(1)	-2750(3)	1405(2)	2524(2)	17(1)
C(2)	-673(3)	485(2)	1381(2)	18(1)
S(2)	-1375(1)	1973(1)	3449(1)	14(1)
Na(2)	9347(1)	9726(1)	7636(1)	17(1)
O(2)	-3872(2)	1027(2)	905(1)	24(1)
C(3)	-1092(4)	-698(2)	1001(2)	22(1)
S(3)	6645(1)	7124(1)	6530(1)	18(1)
O(3)	-698(2)	3171(1)	3405(1)	18(1)
C(4)	239(4)	-1469(2)	937(2)	26(1)
S(4)	7638(1)	7209(1)	8429(1)	15(1)
O(4)	-2243(2)	1831(2)	4150(1)	20(1)
O(5)	2469(3)	3838(2)	1562(1)	36(1)
C(5)	1969(4)	-1075(2)	1246(2)	26(1)
O(6)	-323(2)	5191(2)	1045(1)	26(1)
C(6)	2358(4)	112(2)	1611(2)	23(1)
C(7)	1047(3)	892(2)	1677(2)	19(1)
O(7)	-2192(3)	5711(2)	2273(1)	28(1)
O(8)	753(2)	6019(2)	3524(1)	25(1)
C(8)	3410(4)	-1919(3)	1185(2)	41(1)
O(9)	3160(3)	4301(2)	3326(1)	34(1)
C(9)	493(3)	1098(2)	3640(2)	16(1)
C(10)	270(3)	-151(2)	3539(2)	20(1)
O(10)	7443(2)	8322(2)	6613(1)	21(1)
C(11)	1715(3)	-837(2)	3666(2)	22(1)
O(11)	5076(2)	6824(2)	5872(1)	27(1)
C(12)	3386(4)	-299(2)	3888(2)	21(1)
O(12)	8361(2)	8427(1)	8518(1)	18(1)
C(13)	3581(3)	949(2)	3988(2)	21(1)
O(13)	6792(2)	6902(2)	9104(1)	21(1)
C(14)	2142(3)	1644(2)	3859(2)	18(1)
O(14)	11674(2)	9477(2)	6809(1)	22(1)
C(15)	4947(4)	-1060(3)	4009(2)	30(1)
O(15)	8904(2)	10874(2)	6379(1)	22(1)
C(17)	374(4)	4444(3)	432(2)	34(1)
O(17)	10001(2)	11289(2)	8906(1)	21(1)
C(16)	2266(4)	4374(3)	796(2)	30(1)
O(16)	7086(2)	11196(2)	7645(1)	20(1)
C(18)	-2151(4)	5272(3)	787(2)	35(1)
O(18)	12256(2)	9493(2)	8567(1)	21(1)
C(19)	-2686(4)	6136(3)	1454(2)	38(1)
C(21)	-858(4)	6215(3)	3762(2)	37(1)
C(20)	-2039(4)	6617(3)	2996(3)	42(1)
C(22)	2096(5)	5764(3)	4221(2)	38(1)

C(23)	3621(4)	5403(3)	3862(2)	30(1)
C(26)	6221(3)	6841(2)	7477(2)	19(1)
C(27)	8229(4)	6141(2)	6254(2)	20(1)
C(28)	7734(4)	4952(2)	5916(2)	24(1)
C(29)	8988(4)	4154(2)	5791(2)	28(1)
C(30)	10745(4)	4523(2)	6006(2)	27(1)
C(31)	11211(4)	5720(2)	6321(2)	24(1)
C(32)	9960(4)	6529(2)	6443(2)	21(1)
C(33)	12114(5)	3634(3)	5916(2)	41(1)
C(34)	9472(3)	6295(2)	8480(2)	16(1)
C(35)	9221(3)	5083(2)	8146(2)	18(1)
C(36)	10643(4)	4381(2)	8173(2)	21(1)
C(37)	12319(4)	4869(2)	8525(2)	22(1)
C(38)	12545(4)	6084(2)	8864(2)	23(1)
C(39)	11125(3)	6793(2)	8837(2)	20(1)
C(40)	13862(4)	4104(3)	8540(2)	30(1)
C(41)	11419(4)	10044(3)	6043(2)	28(1)
C(42)	9512(4)	10143(3)	5715(2)	26(1)
C(43)	7091(4)	11052(2)	6156(2)	24(1)
C(44)	6668(4)	11828(2)	6913(2)	24(1)
C(45)	7182(4)	11922(2)	8451(2)	23(1)
C(46)	8356(4)	11350(2)	9139(2)	22(1)
C(47)	11324(4)	10906(2)	9563(2)	24(1)
C(48)	12822(4)	10551(2)	9165(2)	24(1)
C(49)	13507(3)	9042(2)	8098(2)	25(1)
C(50)	13418(4)	9638(3)	7294(2)	25(1)
C(24A)	4407(6)	3727(5)	2928(3)	28(1)
C(25A)	4266(6)	4158(6)	2064(3)	28(2)
C(24B)	4543(12)	4393(15)	2739(7)	29(4)
C(25B)	4009(14)	3424(14)	2000(8)	34(3)

Table S15. Anisotropic displacement parameters (Å^{2}x 10^3) for **2a**. The anisotropic displacement factor exponent takes the form: $-2\pi^2[h^2 a^{*2}U^{11} + ... + 2 h k a^* b^* U^{12}]$

	U^{11}	U^{22}	U^{33}	U^{23}	U^{13}	U^{12}
S(1)	16(1)	19(1)	14(1)	3(1)	3(1)	2(1)
Na(1)	19(1)	17(1)	20(1)	5(1)	5(1)	2(1)
O(1)	24(1)	19(1)	19(1)	7(1)	6(1)	3(1)
C(1)	15(1)	19(1)	18(1)	4(1)	5(1)	-2(1)
C(2)	21(1)	19(1)	15(1)	4(1)	8(1)	3(1)
S(2)	14(1)	15(1)	14(1)	3(1)	5(1)	2(1)
Na(2)	17(1)	19(1)	17(1)	5(1)	4(1)	2(1)
O(2)	18(1)	31(1)	20(1)	1(1)	0(1)	2(1)
C(3)	22(1)	22(1)	24(1)	3(1)	9(1)	-3(1)

S(3)	17(1)	21(1)	16(1)	4(1)	1(1)	0(1)
O(3)	21(1)	15(1)	20(1)	3(1)	4(1)	0(1)
C(4)	32(2)	19(1)	29(1)	2(1)	12(1)	2(1)
S(4)	15(1)	16(1)	16(1)	4(1)	5(1)	2(1)
O(4)	20(1)	24(1)	17(1)	5(1)	9(1)	2(1)
O(5)	28(1)	56(1)	34(1)	22(1)	16(1)	14(1)
C(5)	28(2)	27(1)	25(1)	7(1)	12(1)	9(1)
O(6)	23(1)	28(1)	26(1)	11(1)	4(1)	1(1)
C(6)	19(1)	30(1)	19(1)	4(1)	3(1)	4(1)
C(7)	20(1)	22(1)	15(1)	2(1)	3(1)	0(1)
O(7)	23(1)	17(1)	46(1)	9(1)	10(1)	3(1)
O(8)	25(1)	24(1)	26(1)	-2(1)	9(1)	-1(1)
C(8)	38(2)	36(2)	47(2)	1(1)	10(2)	17(2)
O(9)	21(1)	36(1)	42(1)	-6(1)	4(1)	3(1)
C(9)	18(1)	17(1)	12(1)	3(1)	4(1)	3(1)
C(10)	18(1)	20(1)	21(1)	4(1)	5(1)	-1(1)
O(10)	24(1)	19(1)	20(1)	6(1)	5(1)	2(1)
C(11)	23(1)	17(1)	25(1)	5(1)	6(1)	3(1)
O(11)	22(1)	36(1)	21(1)	4(1)	-3(1)	0(1)
C(12)	23(1)	24(1)	18(1)	4(1)	4(1)	7(1)
O(12)	21(1)	15(1)	19(1)	3(1)	4(1)	1(1)
C(13)	16(1)	24(1)	22(1)	2(1)	2(1)	-1(1)
O(13)	22(1)	24(1)	21(1)	8(1)	10(1)	3(1)
C(14)	19(1)	19(1)	17(1)	2(1)	3(1)	0(1)
O(14)	20(1)	26(1)	22(1)	8(1)	7(1)	1(1)
C(15)	22(2)	29(1)	38(2)	2(1)	3(1)	9(1)
O(15)	21(1)	28(1)	19(1)	8(1)	5(1)	4(1)
C(17)	47(2)	35(2)	20(1)	6(1)	10(1)	2(1)
O(17)	21(1)	23(1)	19(1)	5(1)	6(1)	2(1)
C(16)	38(2)	29(1)	28(1)	6(1)	19(1)	-1(1)
O(16)	23(1)	17(1)	23(1)	7(1)	6(1)	4(1)
C(18)	26(2)	40(2)	40(2)	28(1)	-2(1)	0(1)
O(18)	18(1)	22(1)	24(1)	2(1)	5(1)	1(1)
C(19)	23(2)	32(2)	68(2)	32(2)	12(2)	10(1)
C(21)	35(2)	29(2)	47(2)	-11(1)	21(2)	-2(1)
C(20)	25(2)	20(1)	79(3)	-9(1)	20(2)	2(1)
C(22)	49(2)	43(2)	18(1)	-2(1)	2(1)	5(2)
C(23)	27(2)	30(1)	29(1)	5(1)	-3(1)	-5(1)
C(26)	14(1)	21(1)	21(1)	5(1)	4(1)	1(1)
C(27)	25(2)	21(1)	15(1)	4(1)	5(1)	0(1)
C(28)	30(2)	24(1)	17(1)	2(1)	4(1)	-7(1)
C(29)	47(2)	19(1)	21(1)	1(1)	13(1)	1(1)
C(30)	40(2)	26(1)	19(1)	5(1)	14(1)	9(1)
C(31)	25(2)	29(1)	21(1)	5(1)	9(1)	2(1)

C(32)	25(1)	20(1)	19(1)	1(1)	7(1)	-2(1)
C(33)	58(2)	37(2)	35(2)	6(1)	22(2)	19(2)
C(34)	18(1)	18(1)	15(1)	7(1)	6(1)	4(1)
C(35)	19(1)	19(1)	18(1)	5(1)	4(1)	-2(1)
C(36)	26(2)	17(1)	22(1)	4(1)	8(1)	2(1)
C(37)	22(1)	24(1)	22(1)	8(1)	11(1)	6(1)
C(38)	17(1)	25(1)	25(1)	5(1)	3(1)	-1(1)
C(39)	19(1)	19(1)	22(1)	4(1)	4(1)	1(1)
C(40)	26(2)	30(1)	38(2)	9(1)	13(1)	9(1)
C(41)	29(2)	36(2)	24(1)	12(1)	13(1)	3(1)
C(42)	30(2)	36(2)	16(1)	9(1)	8(1)	5(1)
C(43)	22(1)	28(1)	22(1)	12(1)	1(1)	2(1)
C(44)	20(1)	21(1)	31(1)	14(1)	4(1)	5(1)
C(45)	24(1)	18(1)	32(1)	3(1)	13(1)	4(1)
C(46)	27(2)	19(1)	24(1)	2(1)	13(1)	1(1)
C(47)	27(2)	25(1)	16(1)	2(1)	-1(1)	1(1)
C(48)	20(1)	25(1)	24(1)	4(1)	-2(1)	-3(1)
C(49)	16(1)	29(1)	31(1)	7(1)	6(1)	5(1)
C(50)	19(1)	30(1)	29(1)	6(1)	8(1)	1(1)
C(24A)	21(2)	27(3)	41(3)	11(2)	11(2)	6(2)
C(25A)	21(2)	29(4)	40(3)	8(2)	14(2)	2(2)
C(24B)	13(5)	41(9)	33(7)	11(5)	4(4)	0(4)
C(25B)	22(6)	19(7)	63(8)	8(5)	16(5)	2(5)

Crystal structure analysis of 2b.

Additional information concerning the structure refinement: A disorded 15-crown-5 moiety (occupancy 0.71:0.29) was solved using the PART instructions. All hydrogen atoms were placed on ideal positions except for H1 on C1 which was found in the difference Fourier map and refined independently.

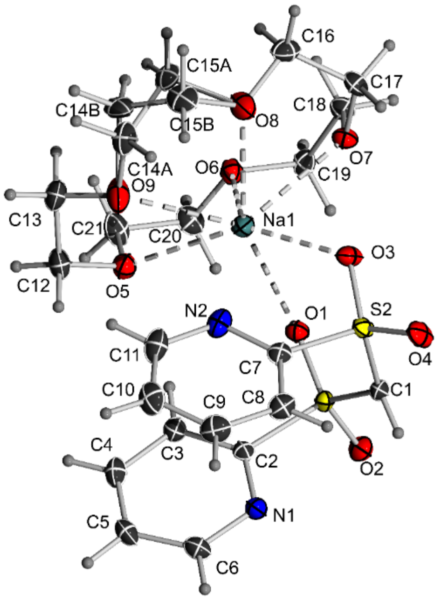

Figure S78. ORTEP plot of **2b**. Ellipsoids are drawn at 50 % probability level.

Table S16. Atomic coordinates (x 10^4) and equivalent isotropic displacement parameters (Å^2 x 10^3) for **2b**. U(eq) is defined as one third of the trace of the orthogonalized U^{ij} tensor.

	x	y	z	U(eq)
S(1)	1091(1)	7047(1)	4722(1)	14(1)
Na(1)	954(1)	7935(1)	6495(1)	18(1)
N(1)	1890(1)	5490(1)	4988(1)	18(1)
C(1)	1456(1)	8408(2)	4817(1)	16(1)
O(1)	769(1)	7195(1)	5151(1)	18(1)
S(2)	1726(1)	9241(1)	5669(1)	16(1)
C(2)	1485(1)	5611(1)	5158(1)	14(1)
N(2)	2001(1)	7430(1)	6808(1)	22(1)
O(2)	846(1)	6724(1)	3897(1)	22(1)
C(3)	1351(1)	4718(2)	5614(1)	19(1)
O(4)	2020(1)	10327(1)	5546(1)	24(1)
C(4)	1656(1)	3597(2)	5896(1)	23(1)
O(5)	707(1)	5366(1)	6648(1)	22(1)
C(5)	2079(1)	3442(2)	5729(1)	21(1)
O(6)	-24(1)	7420(1)	6110(1)	19(1)
C(6)	2183(1)	4411(2)	5283(1)	21(1)
O(7)	347(1)	9950(1)	6047(1)	18(1)
C(7)	2177(1)	8140(2)	6364(1)	17(1)
O(8)	1073(1)	9592(1)	7579(1)	31(1)
C(8)	2658(1)	8003(2)	6363(1)	22(1)
C(9)	2976(1)	7056(2)	6863(1)	27(1)
C(13)	1159(1)	5721(2)	8042(1)	29(1)
C(12)	1050(1)	4786(2)	7366(1)	25(1)
C(11)	2321(1)	6532(2)	7293(1)	28(1)
C(10)	2807(1)	6305(2)	7336(1)	28(1)
C(21)	189(1)	5171(2)	6519(1)	29(1)
C(20)	-129(1)	6064(2)	5861(1)	24(1)
C(19)	-301(1)	8334(2)	5496(1)	20(1)
C(18)	-186(1)	9730(2)	5812(1)	20(1)
C(17)	538(1)	11021(2)	6592(1)	22(1)
C(16)	679(1)	10537(2)	7415(1)	25(1)
O(3)	1363(1)	9571(1)	5997(1)	21(1)
O(9A)	1381(1)	6898(1)	7876(1)	37(1)
C(14A)	1555(1)	7913(3)	8454(1)	25(1)
C(15A)	1130(1)	8894(3)	8311(1)	22(1)
C(14B)	1238(4)	7894(7)	8449(4)	29(2)
C(15B)	1436(3)	9168(7)	8323(3)	30(2)

Table S17. Anisotropic displacement parameters ($Å^2$x 10^3) for **2b**. The anisotropic displacement factor exponent takes the form: $-2\pi^2[h^2 a^{*2}U^{11} + ... + 2 h k a^* b^* U^{12}]$

	U^{11}	U^{22}	U^{33}	U^{23}	U^{13}	U^{12}
S(1)	16(1)	14(1)	12(1)	1(1)	5(1)	2(1)
Na(1)	20(1)	18(1)	16(1)	0(1)	9(1)	0(1)
N(1)	18(1)	16(1)	19(1)	0(1)	8(1)	1(1)
C(1)	21(1)	15(1)	17(1)	3(1)	11(1)	2(1)
O(1)	16(1)	18(1)	19(1)	0(1)	8(1)	2(1)
S(2)	19(1)	12(1)	21(1)	-1(1)	11(1)	-1(1)
C(2)	16(1)	13(1)	12(1)	-2(1)	3(1)	0(1)
N(2)	22(1)	25(1)	18(1)	1(1)	7(1)	-4(1)
O(2)	24(1)	24(1)	14(1)	0(1)	4(1)	3(1)
C(3)	19(1)	19(1)	18(1)	1(1)	8(1)	0(1)
O(4)	27(1)	14(1)	38(1)	-1(1)	20(1)	-3(1)
C(4)	27(1)	18(1)	23(1)	5(1)	9(1)	-2(1)
O(5)	20(1)	25(1)	22(1)	5(1)	10(1)	2(1)
C(5)	22(1)	14(1)	23(1)	0(1)	4(1)	3(1)
O(6)	21(1)	18(1)	16(1)	1(1)	5(1)	0(1)
C(6)	19(1)	19(1)	24(1)	-2(1)	9(1)	2(1)
O(7)	16(1)	19(1)	20(1)	-2(1)	7(1)	1(1)
C(7)	19(1)	16(1)	15(1)	-4(1)	5(1)	-4(1)
O(8)	43(1)	26(1)	17(1)	-2(1)	3(1)	10(1)
C(8)	20(1)	25(1)	21(1)	-2(1)	8(1)	-3(1)
C(9)	18(1)	35(1)	24(1)	-3(1)	5(1)	1(1)
C(13)	38(1)	28(1)	23(1)	10(1)	14(1)	4(1)
C(12)	28(1)	20(1)	26(1)	7(1)	9(1)	4(1)
C(11)	30(1)	31(1)	20(1)	5(1)	6(1)	-5(1)
C(10)	25(1)	29(1)	20(1)	2(1)	-1(1)	1(1)
C(21)	24(1)	22(1)	42(1)	7(1)	14(1)	-2(1)
C(20)	21(1)	20(1)	30(1)	-3(1)	7(1)	-4(1)
C(19)	17(1)	24(1)	16(1)	3(1)	3(1)	2(1)
C(18)	16(1)	23(1)	22(1)	2(1)	8(1)	4(1)
C(17)	24(1)	15(1)	26(1)	-2(1)	9(1)	1(1)
C(16)	30(1)	24(1)	24(1)	-7(1)	14(1)	-2(1)
O(3)	22(1)	19(1)	26(1)	-5(1)	14(1)	-1(1)
O(9A)	59(1)	21(1)	22(1)	4(1)	8(1)	-4(1)
C(14A)	21(2)	29(2)	19(1)	2(1)	0(1)	-3(1)
C(15A)	28(2)	23(2)	14(1)	-3(1)	7(1)	-5(1)
C(14B)	41(5)	31(4)	14(3)	-5(3)	9(3)	10(3)
C(15B)	31(5)	33(4)	16(3)	-7(3)	-2(3)	3(3)

3.4 Crystal Structure Determination of 3a-Cy

Additional information concerning the structure refinement: All hydrogen atoms were placed on ideal positions except for H1 on P1 which was found in the difference Fourier map and refined independently.

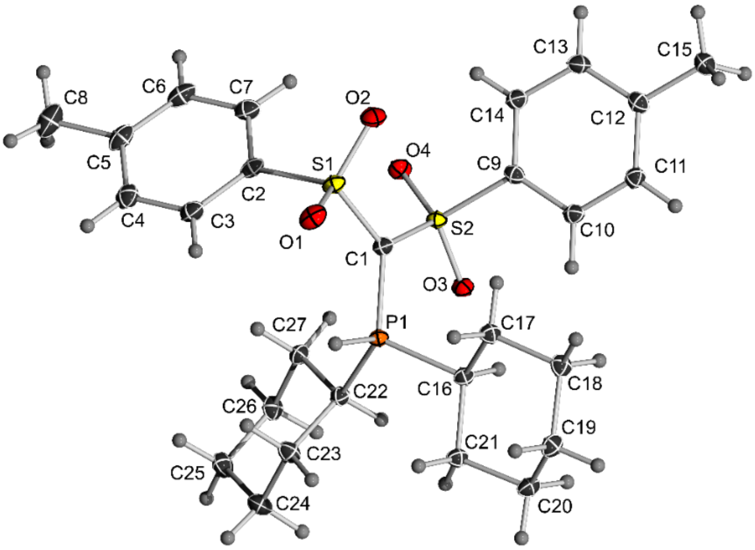

Figure S79. ORTEP plot of **3a-Cy**. Ellipsoids are drawn at 50 % probability level.

Table S18. Atomic coordinates (x 10^4) and equivalent isotropic displacement parameters (Å^2 x 10^3) for **3a-Cy**. U(eq) is defined as one third of the trace of the orthogonalized U^{ij} tensor.

	x	y	z	U(eq)
P(1)	7740(1)	6509(1)	2740(1)	14(1)
S(1)	5645(1)	7312(1)	3334(1)	18(1)
S(2)	5840(1)	4910(1)	2948(1)	14(1)
O(1)	6049(1)	8392(1)	3128(1)	24(1)
O(2)	4337(1)	7056(1)	3189(1)	24(1)
O(3)	6774(1)	4269(1)	2711(1)	18(1)
O(4)	5521(1)	4556(1)	3558(1)	20(1)
C(1)	6382(1)	6266(1)	2999(1)	16(1)
C(2)	6179(1)	7267(1)	4216(1)	19(1)
C(3)	7238(1)	7864(1)	4523(1)	23(1)
C(4)	7619(1)	7872(1)	5214(1)	26(1)
C(5)	6945(1)	7298(1)	5605(1)	25(1)
C(6)	5897(1)	6690(1)	5286(1)	26(1)
C(7)	5510(1)	6667(1)	4595(1)	23(1)
C(8)	7350(2)	7337(1)	6356(1)	34(1)
C(9)	4460(1)	4801(1)	2312(1)	16(1)
C(10)	4530(1)	4675(1)	1651(1)	18(1)

C(11)	3445(1)	4555(1)	1161(1)	19(1)
C(12)	2286(1)	4546(1)	1322(1)	19(1)
C(13)	2242(1)	4658(1)	1988(1)	22(1)
C(14)	3320(1)	4798(1)	2482(1)	21(1)
C(15)	1114(1)	4426(1)	785(1)	24(1)
C(16)	7570(1)	6285(1)	1848(1)	16(1)
C(17)	6493(1)	7019(1)	1464(1)	18(1)
C(18)	6338(1)	6889(1)	709(1)	21(1)
C(19)	7550(1)	7168(1)	509(1)	21(1)
C(20)	8591(1)	6399(1)	876(1)	20(1)
C(21)	8788(1)	6521(1)	1632(1)	18(1)
C(22)	9153(1)	5800(1)	3198(1)	16(1)
C(23)	10250(1)	6640(1)	3309(1)	21(1)
C(24)	11462(1)	6051(1)	3648(1)	25(1)
C(25)	11366(1)	5490(1)	4300(1)	24(1)
C(26)	10257(1)	4689(1)	4190(1)	20(1)
C(27)	9045(1)	5299(1)	3869(1)	18(1)

Table S19. Anisotropic displacement parameters (Å^{2}x 10^3) for **3a-Cy**. The anisotropic displacement factor exponent takes the form: $-2\pi^2[h^2 a^{*2}U^{11} + ... + 2 h k a^* b^* U^{12}]$

	U^{11}	U^{22}	U^{33}	U^{23}	U^{13}	U^{12}
P(1)	15(1)	14(1)	15(1)	1(1)	5(1)	0(1)
S(1)	19(1)	15(1)	21(1)	0(1)	8(1)	3(1)
S(2)	14(1)	13(1)	17(1)	1(1)	5(1)	1(1)
O(1)	32(1)	15(1)	28(1)	2(1)	13(1)	4(1)
O(2)	18(1)	26(1)	30(1)	-2(1)	7(1)	5(1)
O(3)	16(1)	16(1)	22(1)	-1(1)	5(1)	2(1)
O(4)	21(1)	21(1)	18(1)	4(1)	6(1)	-2(1)
C(1)	17(1)	15(1)	18(1)	0(1)	7(1)	0(1)
C(2)	24(1)	15(1)	22(1)	-1(1)	11(1)	4(1)
C(3)	28(1)	20(1)	25(1)	-2(1)	13(1)	-1(1)
C(4)	29(1)	22(1)	26(1)	-5(1)	8(1)	1(1)
C(5)	37(1)	18(1)	23(1)	0(1)	12(1)	9(1)
C(6)	36(1)	20(1)	28(1)	4(1)	18(1)	5(1)
C(7)	25(1)	19(1)	28(1)	0(1)	12(1)	1(1)
C(8)	54(1)	26(1)	24(1)	0(1)	12(1)	8(1)
C(9)	15(1)	14(1)	19(1)	1(1)	4(1)	0(1)

C(10)	17(1)	16(1)	22(1)	-1(1)	7(1)	-1(1)
C(11)	21(1)	18(1)	19(1)	-1(1)	6(1)	-1(1)
C(12)	18(1)	15(1)	23(1)	0(1)	4(1)	-1(1)
C(13)	16(1)	26(1)	25(1)	0(1)	7(1)	0(1)
C(14)	19(1)	25(1)	20(1)	0(1)	7(1)	0(1)
C(15)	18(1)	28(1)	24(1)	-1(1)	2(1)	-2(1)
C(16)	17(1)	16(1)	16(1)	1(1)	5(1)	0(1)
C(17)	17(1)	19(1)	18(1)	1(1)	4(1)	2(1)
C(18)	20(1)	22(1)	19(1)	1(1)	2(1)	2(1)
C(19)	24(1)	22(1)	16(1)	2(1)	5(1)	2(1)
C(20)	21(1)	22(1)	18(1)	1(1)	8(1)	2(1)
C(21)	17(1)	20(1)	17(1)	3(1)	5(1)	1(1)
C(22)	16(1)	17(1)	16(1)	-1(1)	4(1)	1(1)
C(23)	18(1)	22(1)	23(1)	3(1)	4(1)	-3(1)
C(24)	16(1)	30(1)	29(1)	-1(1)	5(1)	-2(1)
C(25)	19(1)	24(1)	24(1)	-1(1)	-2(1)	2(1)
C(26)	22(1)	19(1)	18(1)	1(1)	2(1)	2(1)
C(27)	18(1)	19(1)	16(1)	0(1)	4(1)	0(1)

3.5 Crystal Structure Determination of 4a-Cy

Additional information concerning the structure refinement: The structure contained a twin (ratio 0.84:0.16) which was solved using the BASF and MERG instructions.

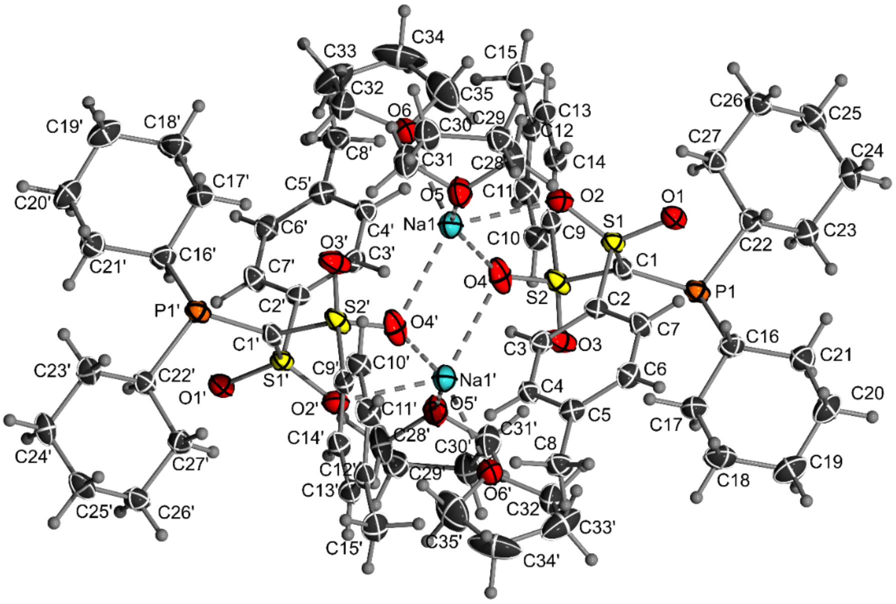

Figure S80. ORTEP plot of **4a-Cy**. Ellipsoids are drawn at 50 % probability level.

Table S20. Atomic coordinates (x 10^4) and equivalent isotropic displacement parameters (Å^2 x 10^3) for **4a-Cy**. U(eq) is defined as one third of the trace of the orthogonalized U^{ij} tensor.

	x	y	z	U(eq)
S(1)	3157(1)	5484(1)	3926(1)	29(1)
C(1)	3118(5)	4993(4)	3119(3)	26(1)
Na(1)	6289(2)	5214(2)	4481(1)	35(1)
O(1)	4248(4)	5405(3)	4408(2)	39(1)
P(1)	1755(1)	4826(1)	2546(1)	26(1)
C(2)	2942(5)	6700(4)	3809(3)	25(1)
S(2)	4300(1)	4613(1)	2951(1)	24(1)
O(2)	2313(4)	5152(3)	4243(2)	41(1)
O(4)	4094(4)	4485(3)	2180(2)	30(1)
C(4)	3436(5)	8169(4)	3401(3)	32(1)
C(3)	3534(5)	7210(4)	3438(3)	30(1)
O(3)	5220(4)	5194(3)	3282(2)	34(1)
C(5)	2732(5)	8620(4)	3737(3)	30(1)
O(5)	7696(4)	4551(3)	4034(2)	39(1)
C(6)	2138(6)	8089(4)	4114(3)	34(2)
O(6)	7025(4)	6682(3)	4546(2)	40(1)
C(7)	2236(5)	7135(4)	4158(3)	30(1)
C(8)	2599(6)	9658(5)	3696(4)	40(2)
C(9)	4630(5)	3483(4)	3335(3)	23(1)
C(00N)	5409(6)	756(4)	4229(4)	33(1)
C(11)	5167(5)	2478(4)	4353(3)	28(1)
C(10)	4901(5)	3354(4)	4073(3)	26(1)
C(13)	4863(5)	1849(4)	3175(3)	28(1)
C(12)	5143(5)	1711(4)	3914(3)	25(1)
C(14)	4612(5)	2727(4)	2885(3)	28(1)
C(15)	1723(5)	5389(4)	1671(3)	26(1)
C(16)	2256(5)	6344(4)	1778(3)	29(1)
C(17)	2099(5)	6893(4)	1077(3)	33(1)
C(18)	923(5)	6961(5)	680(3)	34(2)
C(20)	535(5)	5485(4)	1239(3)	32(1)
C(21)	1751(5)	3558(4)	2344(3)	30(1)
C(22)	830(6)	3258(4)	1699(4)	34(1)
C(23)	777(6)	2206(5)	1609(4)	43(2)
C(24)	666(6)	1726(5)	2286(4)	45(2)
C(25)	1608(6)	1984(5)	2909(4)	42(2)

C(26)	1683(6)	3026(4)	3021(3)	35(2)
C(27)	7629(6)	4541(6)	3281(4)	44(2)
C(28)	8711(6)	4167(5)	3211(4)	37(2)
C(29)	9509(6)	4458(5)	3913(4)	40(2)
C(30)	8776(6)	4820(6)	4361(4)	46(2)
C(31)	6441(8)	7333(7)	4062(5)	64(2)
C(32)	6619(9)	8264(7)	4405(6)	78(3)
C(33)	7367(7)	8072(6)	5142(5)	56(2)
C(34)	7878(6)	7153(5)	5048(4)	40(2)
C(19)	428(6)	6012(5)	532(3)	34(2)

Table S21. Anisotropic displacement parameters ($Å^2$x 10^3) for **4a-Cy**. The anisotropic displacement factor exponent takes the form: $-2\pi^2$[h^2 $a^{*2}U^{11}$ + ... + 2 h k a* b* U^{12}]

	U^{11}	U^{22}	U^{33}	U^{23}	U^{13}	U^{12}
S(1)	47(1)	21(1)	21(1)	2(1)	10(1)	7(1)
C(1)	38(3)	20(3)	18(3)	1(2)	6(2)	2(2)
Na(1)	45(2)	33(1)	27(1)	-4(1)	10(1)	-6(1)
O(1)	59(3)	31(2)	23(2)	-6(2)	4(2)	15(2)
P(1)	33(1)	23(1)	24(1)	1(1)	10(1)	2(1)
C(2)	33(3)	18(3)	21(3)	2(2)	2(2)	5(2)
S(2)	34(1)	19(1)	21(1)	1(1)	9(1)	1(1)
O(2)	73(4)	27(2)	32(2)	1(2)	27(2)	-1(2)
O(4)	42(3)	29(2)	20(2)	2(2)	10(2)	-1(2)
C(4)	35(3)	32(3)	27(3)	5(3)	5(3)	-2(3)
C(3)	36(3)	28(3)	24(3)	-1(2)	6(3)	4(3)
O(3)	37(2)	27(2)	38(3)	5(2)	7(2)	-1(2)
C(5)	38(4)	24(3)	22(3)	-1(2)	-1(3)	5(3)
O(5)	40(3)	48(3)	27(2)	-7(2)	4(2)	-2(2)
C(6)	43(4)	29(3)	30(3)	-2(3)	9(3)	9(3)
O(6)	48(3)	33(3)	33(2)	3(2)	-1(2)	-9(2)
C(7)	37(3)	30(3)	26(3)	-2(3)	10(3)	3(3)
C(8)	56(4)	28(3)	34(4)	-3(3)	4(3)	2(3)
C(9)	28(3)	18(3)	25(3)	2(2)	7(2)	1(2)
C(00N)	42(4)	21(3)	37(4)	5(3)	13(3)	4(3)
C(11)	34(3)	26(3)	22(3)	2(2)	7(2)	-1(3)
C(10)	33(3)	23(3)	23(3)	-3(2)	11(2)	-4(2)
C(13)	31(3)	23(3)	30(3)	-7(2)	8(3)	2(2)
C(12)	26(3)	19(3)	31(3)	2(2)	10(2)	-1(2)

C(14)	36(3)	27(3)	22(3)	-2(2)	9(2)	4(3)
C(15)	35(3)	24(3)	20(3)	2(2)	9(2)	1(2)
C(16)	31(3)	27(3)	27(3)	3(2)	6(3)	0(3)
C(17)	41(4)	25(3)	35(3)	7(3)	11(3)	2(3)
C(18)	40(4)	34(3)	27(3)	7(3)	6(3)	7(3)
C(20)	36(3)	31(3)	27(3)	-3(3)	8(3)	-2(3)
C(21)	37(3)	23(3)	31(3)	-1(2)	7(3)	-3(3)
C(22)	39(4)	27(3)	35(3)	0(3)	10(3)	-3(3)
C(23)	49(4)	31(4)	47(4)	-11(3)	10(3)	-8(3)
C(24)	51(4)	25(3)	61(5)	-5(3)	20(4)	-10(3)
C(25)	52(4)	28(4)	47(4)	9(3)	15(3)	-1(3)
C(26)	47(4)	29(3)	30(3)	4(3)	10(3)	-4(3)
C(27)	48(4)	55(5)	28(3)	-6(3)	6(3)	7(4)
C(28)	45(4)	34(4)	32(3)	-1(3)	11(3)	1(3)
C(29)	41(4)	41(4)	39(4)	-5(3)	9(3)	2(3)
C(30)	49(4)	53(5)	37(4)	-14(3)	13(3)	-12(4)
C(31)	55(5)	79(7)	52(5)	17(5)	4(4)	18(5)
C(32)	95(8)	53(6)	101(8)	47(6)	56(7)	25(5)
C(33)	51(5)	42(4)	84(6)	-21(4)	33(5)	-18(4)
C(34)	39(4)	45(4)	35(4)	-9(3)	9(3)	-8(3)
C(19)	37(4)	37(4)	25(3)	-1(3)	1(3)	6(3)

3.6 Crystal Structure Determination of 3a-iPr

Additional information concerning the structure refinement: All hydrogen atoms were placed on ideal positions except for H1 on P1 which was found in the difference Fourier map and refined independently.

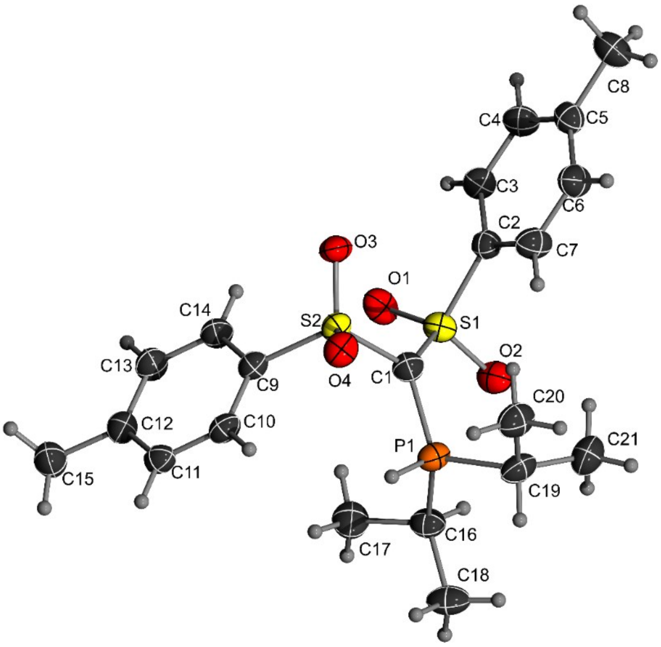

Figure S81. ORTEP plot of **3a-iPr**. Ellipsoids are drawn at 50 % probability level.

Table S22. Atomic coordinates (× 10^4) and equivalent isotropic displacement parameters (Å^2× 10^3) for **3a-iPr**. U(eq) is defined as one third of the trace of the orthogonalized U^{ij} tensor.

	x	y	z	U(eq)
S(1)	7261(1)	3572(1)	3744(1)	31(1)
O(1)	6236(2)	2938(1)	3724(2)	39(1)
C(1)	7033(3)	4211(2)	2755(2)	32(1)
P(1)	6754(1)	5259(1)	2902(1)	32(1)
S(2)	6702(1)	3788(1)	1625(1)	29(1)
O(2)	7393(2)	4114(1)	4573(1)	39(1)
C(2)	8867(3)	3071(2)	3701(2)	30(1)
C(4)	10269(3)	1885(2)	4027(2)	36(1)
O(4)	7122(2)	4376(1)	929(1)	36(1)
C(3)	8992(3)	2252(2)	3981(2)	33(1)
O(3)	7267(2)	2968(1)	1641(1)	34(1)
C(5)	11425(3)	2306(2)	3796(2)	33(1)
C(6)	11261(3)	3130(2)	3513(2)	39(1)
C(7)	9995(3)	3507(2)	3460(2)	41(1)
C(9)	4903(3)	3698(2)	1386(2)	29(1)

C(8)	12793(3)	1890(2)	3842(2)	47(1)
C(10)	4169(3)	4350(2)	940(2)	32(1)
C(11)	2762(3)	4290(2)	752(2)	33(1)
C(12)	2068(3)	3579(2)	990(2)	36(1)
C(13)	2829(3)	2934(2)	1432(2)	38(1)
C(14)	4232(3)	2988(2)	1637(2)	34(1)
C(15)	551(3)	3506(2)	751(2)	46(1)
C(16)	5514(3)	5513(2)	3763(2)	35(1)
C(17)	4180(3)	5056(2)	3479(2)	47(1)
C(18)	5309(4)	6447(2)	3809(2)	46(1)
C(19)	8258(3)	5918(2)	3067(2)	39(1)
C(20)	9170(3)	5776(2)	2240(2)	44(1)
C(21)	9031(4)	5827(2)	4072(2)	49(1)

Table S23. Anisotropic displacement parameters (Å^{2}x 10^3) for **3a-iPr**. The anisotropic displacement factor exponent takes the form: $-2p^2[h^2 a^{*2}U^{11} + ... + 2 h k a^* b^* U^{12}]$

	U^{11}	U^{22}	U^{33}	U^{23}	U^{13}	U^{12}
S(1)	32(1)	31(1)	30(1)	3(1)	7(1)	2(1)
O(1)	32(1)	41(1)	46(1)	11(1)	8(1)	-2(1)
C(1)	33(1)	32(1)	31(1)	0(1)	5(1)	0(1)
P(1)	35(1)	28(1)	35(1)	-3(1)	9(1)	0(1)
S(2)	31(1)	27(1)	28(1)	-1(1)	6(1)	-2(1)
O(2)	47(1)	43(1)	28(1)	1(1)	6(1)	9(1)
C(2)	31(1)	30(1)	29(1)	-1(1)	3(1)	1(1)
C(4)	42(2)	33(1)	34(1)	7(1)	5(1)	5(1)
O(4)	40(1)	37(1)	31(1)	2(1)	9(1)	-6(1)
C(3)	35(1)	32(1)	33(1)	5(1)	6(1)	-2(1)
O(3)	36(1)	26(1)	41(1)	-4(1)	5(1)	3(1)
C(5)	33(1)	39(1)	28(1)	1(1)	2(1)	3(1)
C(6)	33(1)	35(1)	51(2)	1(1)	7(1)	-3(1)
C(7)	39(2)	30(1)	54(2)	3(1)	11(1)	-2(1)
C(9)	34(1)	27(1)	27(1)	-2(1)	4(1)	-2(1)
C(8)	38(2)	53(2)	48(2)	8(1)	2(1)	10(1)
C(10)	41(1)	26(1)	30(1)	0(1)	4(1)	-3(1)
C(11)	41(1)	28(1)	29(1)	1(1)	0(1)	3(1)
C(12)	37(1)	35(1)	35(1)	-3(1)	1(1)	-2(1)
C(13)	39(1)	30(1)	44(2)	4(1)	1(1)	-5(1)
C(14)	37(1)	26(1)	39(1)	3(1)	2(1)	0(1)

C(15)	40(2)	42(2)	54(2)	2(1)	-5(1)	-2(1)
C(16)	39(1)	37(1)	31(1)	-1(1)	9(1)	5(1)
C(17)	38(2)	60(2)	43(2)	-10(1)	12(1)	0(1)
C(18)	56(2)	40(2)	44(2)	-1(1)	16(1)	13(1)
C(19)	45(2)	27(1)	46(2)	-6(1)	11(1)	-4(1)
C(20)	43(2)	35(1)	54(2)	-4(1)	14(1)	-9(1)
C(21)	50(2)	47(2)	49(2)	-8(1)	0(1)	-11(1)

3.7 Crystal Structure Determination of 3a-Ph

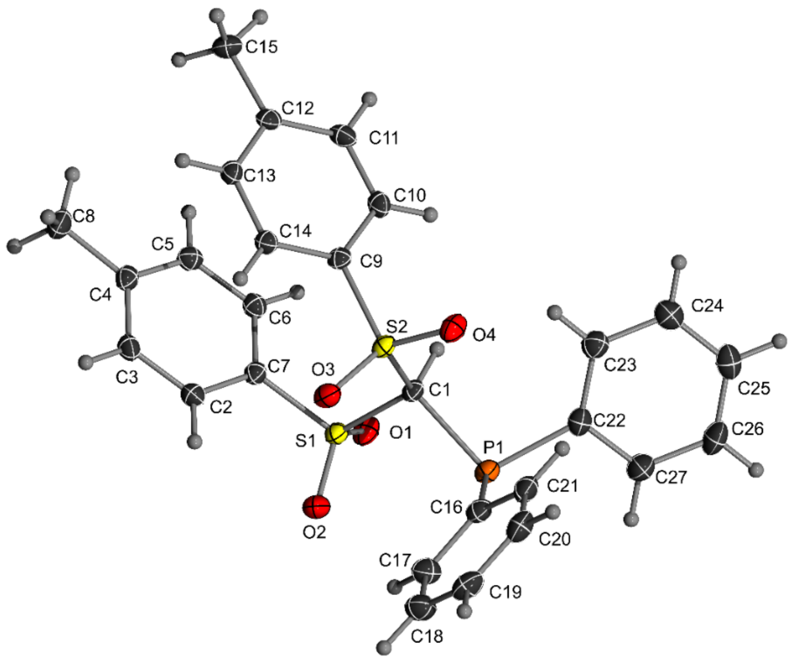

Figure S82. ORTEP plot of **3a-Ph**. Ellipsoids are drawn at 50 % probability level.

Table S24. Atomic coordinates (x 10^4) and equivalent isotropic displacement parameters (Å^{2}x 10^3) for **3a-Ph** U(eq) is defined as one third of the trace of the orthogonalized U^{ij} tensor.

	x	y	z	U(eq)
S(1)	7253(1)	4477(1)	6858(1)	18(1)
O(1)	8143(1)	4893(2)	7268(1)	23(1)
P(1)	6030(1)	4645(1)	8082(1)	20(1)
C(1)	6274(1)	5582(2)	7217(1)	16(1)
C(2)	7385(1)	4988(2)	5977(1)	17(1)
S(2)	5205(1)	5956(1)	6621(1)	17(1)
O(2)	6937(1)	3001(1)	6867(1)	26(1)
C(3)	7006(1)	4106(2)	5435(1)	19(1)
O(3)	4947(1)	4659(1)	6242(1)	22(1)
C(4)	7128(1)	4507(2)	4740(1)	20(1)

O(4)	4473(1)	6627(2)	7039(1)	23(1)
C(5)	7606(1)	5791(2)	4582(1)	20(1)
C(6)	7996(1)	6641(2)	5140(1)	20(1)
C(7)	7893(1)	6252(2)	5840(1)	19(1)
C(8)	7662(2)	6270(2)	3826(1)	27(1)
C(9)	5602(1)	7256(2)	6017(1)	17(1)
C(10)	6019(1)	8548(2)	6263(1)	20(1)
C(11)	6318(1)	9550(2)	5777(1)	21(1)
C(12)	6189(1)	9296(2)	5050(1)	19(1)
C(13)	5725(1)	8027(2)	4817(1)	18(1)
C(14)	5440(1)	6997(2)	5296(1)	17(1)
C(15)	6558(2)	10343(2)	4517(1)	25(1)
C(16)	4841(1)	3688(2)	7928(1)	20(1)
C(17)	4900(1)	2279(2)	7674(1)	26(1)
C(18)	4042(2)	1446(2)	7588(1)	28(1)
C(19)	3130(2)	1995(2)	7768(1)	29(1)
C(20)	3073(1)	3378(2)	8023(1)	27(1)
C(21)	3918(1)	4226(2)	8103(1)	24(1)
C(22)	5749(1)	6113(2)	8680(1)	22(1)
C(23)	5435(1)	7502(2)	8506(1)	24(1)
C(24)	5308(1)	8515(2)	9035(1)	27(1)
C(25)	5478(1)	8146(2)	9739(1)	28(1)
C(26)	5785(2)	6777(2)	9922(1)	32(1)
C(27)	5931(2)	5773(2)	9394(1)	28(1)

Table S25. Anisotropic displacement parameters ($Å^2 \times 10^3$) **3a-Ph**. The anisotropic displacement factor exponent takes the form: $-2p^2[h^2a^{*2}U^{11} + ... + 2hka^* b^* U^{12}]$

	U^{11}	U^{22}	U^{33}	U^{23}	U^{13}	U^{12}
S(1)	20(1)	18(1)	15(1)	3(1)	1(1)	3(1)
O(1)	20(1)	32(1)	18(1)	4(1)	-3(1)	5(1)
P(1)	22(1)	21(1)	16(1)	2(1)	0(1)	1(1)
C(1)	18(1)	18(1)	12(1)	2(1)	0(1)	1(1)
C(2)	18(1)	18(1)	15(1)	1(1)	1(1)	2(1)
S(2)	17(1)	19(1)	14(1)	2(1)	-2(1)	-1(1)
O(2)	34(1)	18(1)	25(1)	4(1)	6(1)	2(1)
C(3)	20(1)	16(1)	20(1)	-2(1)	1(1)	1(1)
O(3)	25(1)	21(1)	19(1)	2(1)	-4(1)	-6(1)
C(4)	23(1)	20(1)	17(1)	-4(1)	-1(1)	2(1)

O(4)	20(1)	30(1)	20(1)	4(1)	2(1)	4(1)
C(5)	20(1)	22(1)	18(1)	1(1)	3(1)	4(1)
C(6)	19(1)	18(1)	22(1)	1(1)	2(1)	0(1)
C(7)	19(1)	19(1)	19(1)	-2(1)	-1(1)	1(1)
C(8)	32(1)	31(1)	18(1)	2(1)	4(1)	1(1)
C(9)	17(1)	17(1)	16(1)	2(1)	-2(1)	1(1)
C(10)	23(1)	20(1)	16(1)	-3(1)	-3(1)	1(1)
C(11)	24(1)	14(1)	24(1)	-2(1)	-4(1)	0(1)
C(12)	21(1)	15(1)	22(1)	2(1)	2(1)	4(1)
C(13)	21(1)	19(1)	14(1)	-1(1)	0(1)	2(1)
C(14)	18(1)	16(1)	16(1)	-1(1)	-2(1)	0(1)
C(15)	31(1)	20(1)	26(1)	5(1)	3(1)	-1(1)
C(16)	22(1)	22(1)	17(1)	4(1)	-1(1)	-2(1)
C(17)	26(1)	23(1)	28(1)	2(1)	2(1)	1(1)
C(18)	34(1)	22(1)	29(1)	2(1)	0(1)	-4(1)
C(19)	28(1)	33(1)	25(1)	8(1)	0(1)	-7(1)
C(20)	25(1)	37(1)	19(1)	4(1)	3(1)	-1(1)
C(21)	27(1)	27(1)	17(1)	3(1)	2(1)	2(1)
C(22)	22(1)	25(1)	17(1)	-1(1)	2(1)	-1(1)
C(23)	27(1)	25(1)	22(1)	0(1)	-1(1)	-1(1)
C(24)	27(1)	26(1)	29(1)	-2(1)	2(1)	0(1)
C(25)	24(1)	34(1)	26(1)	-9(1)	4(1)	-4(1)
C(26)	38(1)	40(1)	16(1)	0(1)	1(1)	-1(1)
C(27)	36(1)	27(1)	22(1)	2(1)	-1(1)	4(1)

3.8 Crystal Structure Determination of 3b-Cy

Additional information concerning the structure refinement: A disordered pentane and a disorded THF on a symmetry centre were solved using the PART, RIGU and SAME instructions. All hydrogen atoms were placed on ideal positions except for H1 on P1 which was found in the difference Fourier map and refined independently.

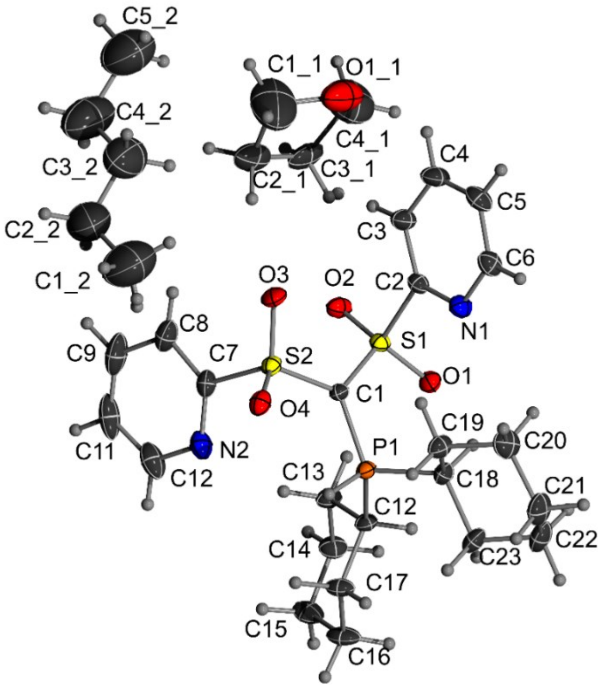

Figure S83. ORTEP plot of **3b-Cy$_2$**. Ellipsoids are drawn at 50 % probability level.

Table S26. Atomic coordinates (x 10^4) and equivalent isotropic displacement parameters (Å^2 x 10^3) for **3b-Cy$_2$**. U(eq) is defined as one third of the trace of the orthogonalized U^{ij} tensor.

	x	y	z	U(eq)
S(1)	2899(1)	2931(1)	3421(1)	18(1)
S(2)	5500(1)	3252(1)	3042(1)	20(1)
P(1)	2889(1)	4859(1)	4759(1)	18(1)
O(1)	1479(1)	3585(1)	4079(1)	23(1)
O(2)	3350(1)	2742(1)	2217(1)	25(1)
O(3)	6023(1)	2004(1)	2637(1)	29(1)
O(4)	6058(1)	3608(1)	3732(1)	24(1)
N(1)	2841(2)	1428(2)	5091(1)	25(1)
N(2)	5334(2)	5336(2)	2043(2)	29(1)
C(1)	3780(2)	3660(2)	3749(2)	18(1)
C(2)	3241(2)	1444(2)	3973(2)	20(1)
C(3)	3864(2)	432(2)	3244(2)	27(1)
C(4)	4089(2)	-690(2)	3727(2)	32(1)

C(5)	3697(2)	-740(2)	4884(2)	30(1)
C(6)	3083(2)	333(2)	5530(2)	28(1)
C(7)	5856(2)	4148(2)	1814(2)	23(1)
C(8)	6632(2)	3594(2)	746(2)	35(1)
C(9)	6881(3)	4361(3)	-158(2)	47(1)
C(10)	6358(3)	5585(3)	45(2)	46(1)
C(11)	5591(2)	6040(2)	1152(2)	40(1)
C(12)	1724(2)	6201(2)	4447(2)	20(1)
C(13)	1860(2)	6278(2)	3217(2)	24(1)
C(14)	821(2)	7451(2)	3087(2)	28(1)
C(15)	1001(2)	8565(2)	3446(2)	34(1)
C(16)	904(2)	8482(2)	4656(2)	34(1)
C(17)	1929(2)	7315(2)	4801(2)	27(1)
C(18)	2005(2)	4378(2)	6164(2)	22(1)
C(19)	3029(2)	3509(2)	6597(2)	26(1)
C(20)	2277(3)	3015(2)	7726(2)	37(1)
C(21)	1250(2)	4035(2)	8606(2)	38(1)
C(22)	270(2)	4920(2)	8156(2)	35(1)
C(23)	1020(2)	5430(2)	7042(2)	27(1)
O11	4914(6)	-1021(4)	801(4)	71(1)
C11	6156(15)	-696(12)	128(15)	135(7)
C21	5628(11)	584(8)	-210(7)	53(2)
C31	4098(9)	702(7)	-102(5)	47(2)
C41	3945(17)	-533(12)	240(15)	100(4)
C12	9830(50)	1380(30)	1552(17)	136(6)
C22	10221(11)	1114(9)	218(9)	108(3)
C32	9990(50)	-110(20)	149(16)	105(5)
C42	10324(12)	-350(9)	-1117(8)	115(3)
C52	10000(50)	-1590(30)	-1194(19)	136(6)

Table S27. Anisotropic displacement parameters ($Å^2$x 10^3) for 3b-Cy_2. The anisotropic displacement factor exponent takes the form: $-2\pi^2[h^2 a^{*2}U^{11} + ... + 2 h k a^* b^* U^{12}]$

	U^{11}	U^{22}	U^{33}	U^{23}	U^{13}	U^{12}
S(1)	20(1)	14(1)	18(1)	1(1)	-7(1)	-3(1)
S(2)	17(1)	18(1)	19(1)	2(1)	-5(1)	-2(1)
P(1)	18(1)	15(1)	17(1)	-1(1)	-5(1)	-2(1)
O(1)	18(1)	21(1)	27(1)	1(1)	-8(1)	-4(1)
O(2)	36(1)	20(1)	20(1)	1(1)	-12(1)	-7(1)

O(3)	22(1)	19(1)	32(1)	-4(1)	-4(1)	2(1)
O(4)	20(1)	27(1)	23(1)	3(1)	-8(1)	-4(1)
N(1)	31(1)	21(1)	22(1)	4(1)	-10(1)	-8(1)
N(2)	28(1)	29(1)	31(1)	11(1)	-12(1)	-11(1)
C(1)	17(1)	15(1)	17(1)	-2(1)	-4(1)	-3(1)
C(2)	22(1)	16(1)	24(1)	3(1)	-10(1)	-6(1)
C(3)	35(1)	20(1)	25(1)	0(1)	-11(1)	-7(1)
C(4)	41(1)	17(1)	36(1)	-1(1)	-16(1)	-6(1)
C(5)	36(1)	21(1)	39(1)	10(1)	-21(1)	-12(1)
C(6)	36(1)	26(1)	26(1)	8(1)	-15(1)	-13(1)
C(7)	19(1)	31(1)	20(1)	6(1)	-7(1)	-10(1)
C(8)	28(1)	51(1)	23(1)	-1(1)	-6(1)	-14(1)
C(9)	37(1)	87(2)	19(1)	9(1)	-7(1)	-29(1)
C(10)	38(1)	78(2)	36(1)	35(1)	-23(1)	-35(1)
C(11)	36(1)	42(1)	49(1)	26(1)	-24(1)	-20(1)
C(12)	18(1)	15(1)	22(1)	-2(1)	-7(1)	-1(1)
C(13)	28(1)	18(1)	23(1)	0(1)	-11(1)	-1(1)
C(14)	31(1)	21(1)	32(1)	3(1)	-16(1)	-2(1)
C(15)	34(1)	16(1)	51(1)	5(1)	-22(1)	-2(1)
C(16)	36(1)	15(1)	50(1)	-6(1)	-23(1)	2(1)
C(17)	28(1)	16(1)	37(1)	-4(1)	-17(1)	0(1)
C(18)	23(1)	24(1)	17(1)	-1(1)	-4(1)	-7(1)
C(19)	30(1)	24(1)	18(1)	2(1)	-8(1)	-4(1)
C(20)	49(1)	37(1)	24(1)	9(1)	-15(1)	-16(1)
C(21)	40(1)	53(2)	17(1)	4(1)	-7(1)	-20(1)
C(22)	26(1)	54(1)	20(1)	-6(1)	-3(1)	-14(1)
C(23)	21(1)	33(1)	20(1)	-7(1)	-4(1)	-3(1)
O11	107(4)	43(2)	48(2)	1(2)	-23(3)	-13(2)
C11	93(6)	67(7)	189(15)	46(8)	-27(7)	-5(5)
C21	87(5)	44(4)	25(3)	-2(3)	-21(3)	-19(3)
C31	64(4)	41(4)	19(2)	-8(2)	-10(3)	-1(3)
C41	136(10)	71(6)	101(9)	32(6)	-58(8)	-36(6)
C12	118(10)	116(10)	136(11)	-32(10)	-28(12)	-11(6)
C22	90(6)	80(5)	121(7)	-17(5)	-10(6)	-21(5)
C32	124(6)	77(8)	87(9)	6(8)	-19(9)	-28(7)
C42	134(9)	85(6)	86(6)	-1(5)	-34(6)	2(5)
C52	118(10)	116(10)	136(11)	-32(10)	-28(12)	-11(6)

3.9 Crystal Structure Determination of 4a-Ph

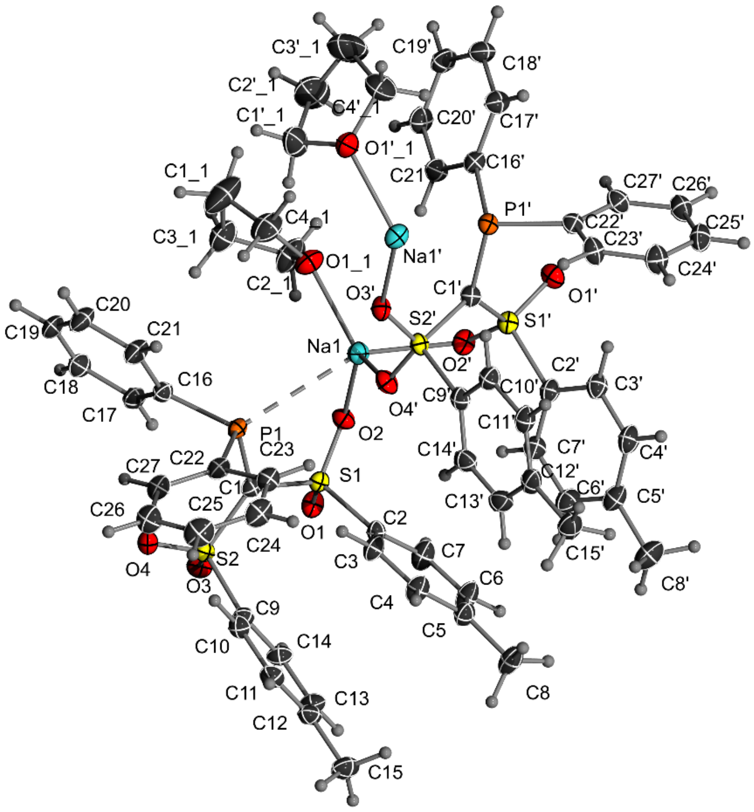

Figure S84. ORTEP plot of **4a-Ph**. Ellipsoids are drawn at 50 % probability level.

Table S28. Atomic coordinates (x 10^4) and equivalent isotropic displacement parameters (Å^{2}x 10^3) for **4a-Ph**. U(eq) is defined as one third of the trace of the orthogonalized U^{ij} tensor.

	x	y	z	U(eq)
S(1)	1811(1)	7588(1)	2622(1)	17(1)
Na(1')	9161(1)	8361(1)	2928(1)	24(1)
O(1')	6379(1)	7718(1)	-138(1)	25(1)
S(1')	6022(1)	7446(1)	694(1)	19(1)
P(1')	8137(1)	8293(1)	1112(1)	18(1)
C(1')	6944(1)	7761(1)	1397(1)	19(1)
O(1)	695(1)	7640(1)	2379(1)	23(1)
Na(1)	4268(1)	8402(1)	2152(1)	25(1)
P(1)	3124(1)	8409(1)	3884(1)	17(1)
C(1)	1942(1)	7868(1)	3646(1)	17(1)
C(2')	6059(1)	6264(1)	727(1)	21(1)
O(2)	2515(1)	8098(1)	2091(1)	23(1)
S(2)	1016(1)	7523(1)	4385(1)	17(1)

O(2')	4930(1)	7748(1)	944(1)	23(1)
S(2')	6783(1)	7447(1)	2427(1)	18(1)
C(2)	2254(1)	6446(1)	2484(1)	19(1)
C(3)	3005(1)	6023(1)	3006(1)	23(1)
O(3)	-70(1)	7667(1)	4080(1)	22(1)
O(3')	7433(1)	7968(1)	2944(1)	22(1)
C(3')	7010(1)	5780(1)	492(1)	25(1)
C(4')	7058(1)	4855(1)	537(1)	29(1)
O(4)	1228(1)	7919(1)	5177(1)	23(1)
O(4')	5649(1)	7448(1)	2657(1)	25(1)
C(4)	3304(1)	5126(1)	2900(1)	27(1)
C(5)	2870(1)	4640(1)	2276(1)	25(1)
C(5')	6175(2)	4402(1)	806(1)	29(1)
C(6')	5229(1)	4900(1)	1023(1)	30(1)
C(6)	2138(2)	5085(1)	1743(1)	31(1)
C(7')	5167(1)	5825(1)	997(1)	25(1)
C(7)	1825(1)	5980(1)	1846(1)	28(1)
C(8)	3166(2)	3655(1)	2183(1)	34(1)
C(8')	6247(2)	3397(1)	862(1)	40(1)
C(9)	1236(1)	6351(1)	4518(1)	19(1)
C(9')	7295(1)	6321(1)	2573(1)	21(1)
C(10)	2049(1)	6003(1)	5037(1)	22(1)
C(10')	8396(1)	6115(1)	2517(1)	24(1)
C(11)	2246(1)	5083(1)	5104(1)	24(1)
C(11')	8800(1)	5232(1)	2578(1)	26(1)
C(12)	1660(1)	4510(1)	4654(1)	25(1)
C(12')	8121(2)	4545(1)	2701(1)	28(1)
C(13)	850(1)	4877(1)	4145(1)	26(1)
C(13')	7022(2)	4767(1)	2768(1)	30(1)
C(17')	8480(1)	9802(1)	121(1)	27(1)
C(17)	3440(1)	9945(1)	4748(1)	28(1)
C(16)	2668(1)	9467(1)	4371(1)	21(1)
C(16')	7708(1)	9338(1)	567(1)	21(1)
C(15)	1888(2)	3514(1)	4731(1)	32(1)
C(15')	8560(2)	3587(1)	2762(1)	38(1)
C(14)	631(1)	5792(1)	4075(1)	23(1)
C(14')	6604(1)	5645(1)	2707(1)	26(1)
C(19)	2121(2)	11148(1)	5042(1)	30(1)

C(19')	7190(2)	11046(1)	-108(1)	29(1)
C(18)	3167(2)	10779(1)	5085(1)	33(1)
C(18')	8221(2)	10647(1)	-216(1)	30(1)
C(20)	1354(1)	10691(1)	4657(1)	28(1)
C(20')	6418(1)	10595(1)	335(1)	28(1)
C(21)	1624(1)	9853(1)	4320(1)	23(1)
C(21')	6674(1)	9748(1)	673(1)	25(1)
C(22)	3757(1)	7788(1)	4771(1)	20(1)
C(22')	8826(1)	7610(1)	284(1)	21(1)
C(23)	4550(1)	7117(1)	4569(1)	24(1)
C(23')	9533(1)	6916(1)	573(1)	27(1)
C(24)	5031(1)	6589(1)	5202(1)	32(1)
C(24')	10024(1)	6294(1)	22(1)	30(1)
C(25)	4752(2)	6746(1)	6037(1)	34(1)
C(25')	9856(1)	6382(1)	-833(1)	30(1)
C(26)	3996(1)	7432(1)	6247(1)	30(1)
C(26')	9190(1)	7086(1)	-1131(1)	29(1)
C(27')	8670(1)	7695(1)	-581(1)	25(1)
C(28)	3498(1)	7949(1)	5620(1)	24(1)
O11	4312(1)	9916(1)	2065(1)	33(1)
O1'1	9361(1)	9834(1)	2914(1)	33(1)
C1'1	8473(2)	10434(1)	3175(1)	47(1)
C11	3096(2)	11121(1)	2436(1)	40(1)
C21	3332(2)	10396(1)	1802(1)	43(1)
C2'1	8278(2)	11064(2)	2438(2)	61(1)
C31	4211(2)	11361(2)	2617(2)	64(1)
C3'1	9377(3)	11147(2)	2094(2)	83(1)
C41	4864(2)	10473(1)	2600(2)	43(1)
C4'1	9948(2)	10254(2)	2246(2)	50(1)

Table S29. Anisotropic displacement parameters ($Å^2×10^3$) for **4a-Ph** The anisotropic displacement factor exponent takes the form: $-2p^2[h^2a^{*2}U^{11} + ... + 2 h k a^{*}b^{*}U^{12}]$

	U^{11}	U^{22}	U^{33}	U^{23}	U^{13}	U^{12}
S(1)	16(1)	16(1)	18(1)	0(1)	-2(1)	1(1)
Na(1')	19(1)	21(1)	30(1)	-1(1)	-5(1)	3(1)
O(1')	24(1)	31(1)	20(1)	2(1)	-4(1)	-3(1)
S(1')	16(1)	21(1)	19(1)	-2(1)	-3(1)	0(1)
P(1')	16(1)	19(1)	20(1)	-1(1)	-1(1)	0(1)

C(1')	17(1)	20(1)	19(1)	-1(1)	-2(1)	1(1)
O(1)	17(1)	25(1)	25(1)	-4(1)	-6(1)	4(1)
Na(1)	21(1)	21(1)	30(1)	-1(1)	4(1)	1(1)
P(1)	16(1)	16(1)	21(1)	-2(1)	-1(1)	0(1)
C(1)	16(1)	16(1)	20(1)	0(1)	-2(1)	0(1)
C(2')	20(1)	23(1)	21(1)	-4(1)	-4(1)	0(1)
O(2)	25(1)	24(1)	20(1)	3(1)	-1(1)	-2(1)
S(2)	16(1)	17(1)	19(1)	1(1)	0(1)	1(1)
O(2')	17(1)	25(1)	28(1)	-3(1)	-4(1)	3(1)
S(2')	17(1)	21(1)	18(1)	-1(1)	0(1)	1(1)
C(2)	18(1)	19(1)	19(1)	-2(1)	0(1)	2(1)
C(3)	22(1)	22(1)	25(1)	-5(1)	-6(1)	1(1)
O(3)	16(1)	23(1)	27(1)	4(1)	-2(1)	1(1)
O(3')	22(1)	25(1)	20(1)	-4(1)	-2(1)	-1(1)
C(3')	21(1)	27(1)	26(1)	-5(1)	-1(1)	0(1)
C(4')	26(1)	29(1)	31(1)	-8(1)	-2(1)	5(1)
O(4)	24(1)	23(1)	21(1)	-3(1)	1(1)	0(1)
O(4')	18(1)	32(1)	25(1)	1(1)	3(1)	1(1)
C(4)	28(1)	24(1)	29(1)	-2(1)	-8(1)	6(1)
C(5)	29(1)	20(1)	25(1)	-2(1)	-1(1)	2(1)
C(5')	33(1)	24(1)	30(1)	-6(1)	-8(1)	-1(1)
C(6')	27(1)	27(1)	35(1)	-4(1)	-3(1)	-5(1)
C(6)	38(1)	25(1)	28(1)	-8(1)	-11(1)	3(1)
C(7')	21(1)	27(1)	28(1)	-4(1)	-2(1)	-1(1)
C(7)	32(1)	26(1)	26(1)	-4(1)	-12(1)	6(1)
C(8)	45(1)	21(1)	37(1)	-4(1)	-8(1)	4(1)
C(8')	46(1)	25(1)	50(1)	-5(1)	-8(1)	0(1)
C(9)	20(1)	18(1)	20(1)	3(1)	1(1)	0(1)
C(9')	24(1)	23(1)	16(1)	0(1)	-3(1)	1(1)
C(10)	23(1)	24(1)	20(1)	0(1)	-2(1)	0(1)
C(10')	26(1)	22(1)	22(1)	-1(1)	-3(1)	0(1)
C(11)	25(1)	24(1)	23(1)	6(1)	-2(1)	4(1)
C(11')	31(1)	26(1)	22(1)	-3(1)	-4(1)	5(1)
C(12)	27(1)	20(1)	26(1)	5(1)	4(1)	-1(1)
C(12')	46(1)	23(1)	16(1)	0(1)	-2(1)	2(1)
C(13)	27(1)	21(1)	31(1)	1(1)	-3(1)	-6(1)
C(13')	43(1)	26(1)	23(1)	2(1)	0(1)	-8(1)
C(17')	22(1)	24(1)	34(1)	2(1)	0(1)	-1(1)
C(17)	23(1)	22(1)	40(1)	-7(1)	-4(1)	-1(1)
C(16)	22(1)	17(1)	23(1)	-1(1)	0(1)	-1(1)

C(16')	22(1)	20(1)	23(1)	-1(1)	-2(1)	-1(1)
C(15)	34(1)	20(1)	42(1)	5(1)	1(1)	0(1)
C(15')	60(1)	24(1)	30(1)	1(1)	3(1)	5(1)
C(14)	23(1)	21(1)	26(1)	4(1)	-4(1)	-3(1)
C(14')	29(1)	28(1)	20(1)	2(1)	0(1)	-5(1)
C(19)	38(1)	16(1)	34(1)	-4(1)	5(1)	0(1)
C(19')	37(1)	20(1)	31(1)	2(1)	-5(1)	2(1)
C(18)	36(1)	22(1)	43(1)	-9(1)	-3(1)	-6(1)
C(18')	32(1)	24(1)	35(1)	4(1)	1(1)	-6(1)
C(20)	29(1)	21(1)	32(1)	1(1)	4(1)	5(1)
C(20')	28(1)	26(1)	31(1)	-1(1)	-2(1)	6(1)
C(21)	22(1)	19(1)	27(1)	-1(1)	-1(1)	1(1)
C(21')	24(1)	24(1)	27(1)	2(1)	1(1)	1(1)
C(22)	16(1)	20(1)	26(1)	-2(1)	-4(1)	-1(1)
C(22')	17(1)	22(1)	23(1)	-1(1)	0(1)	-2(1)
C(23)	19(1)	24(1)	30(1)	-4(1)	-5(1)	1(1)
C(23')	23(1)	34(1)	23(1)	-1(1)	-1(1)	6(1)
C(24)	24(1)	28(1)	43(1)	-2(1)	-9(1)	7(1)
C(24')	26(1)	33(1)	30(1)	-3(1)	-1(1)	8(1)
C(25)	33(1)	34(1)	35(1)	4(1)	-16(1)	4(1)
C(25')	28(1)	33(1)	29(1)	-9(1)	4(1)	2(1)
C(26)	31(1)	34(1)	26(1)	-1(1)	-10(1)	0(1)
C(26')	31(1)	36(1)	21(1)	-3(1)	1(1)	-2(1)
C(27')	23(1)	28(1)	22(1)	2(1)	0(1)	0(1)
C(28)	22(1)	24(1)	26(1)	-3(1)	-6(1)	1(1)
O11	35(1)	23(1)	42(1)	-2(1)	-4(1)	0(1)
O1'1	37(1)	24(1)	37(1)	-2(1)	8(1)	2(1)
C1'1	58(1)	31(1)	47(1)	2(1)	19(1)	14(1)
C11	45(1)	31(1)	45(1)	-5(1)	-7(1)	4(1)
C21	52(1)	30(1)	48(1)	-7(1)	-20(1)	10(1)
C2'1	70(2)	40(1)	70(2)	12(1)	8(1)	12(1)
C31	60(2)	36(1)	99(2)	-24(1)	-17(1)	-6(1)
C3'1	110(3)	46(2)	88(2)	23(2)	37(2)	-4(2)
C41	34(1)	40(1)	55(1)	-8(1)	-7(1)	-8(1)
C4'1	43(1)	51(1)	54(1)	1(1)	16(1)	-13(1)

3.10 Crystal Structure Determination of 5a and 5b

Crystal Structure Deterimination of 5a.

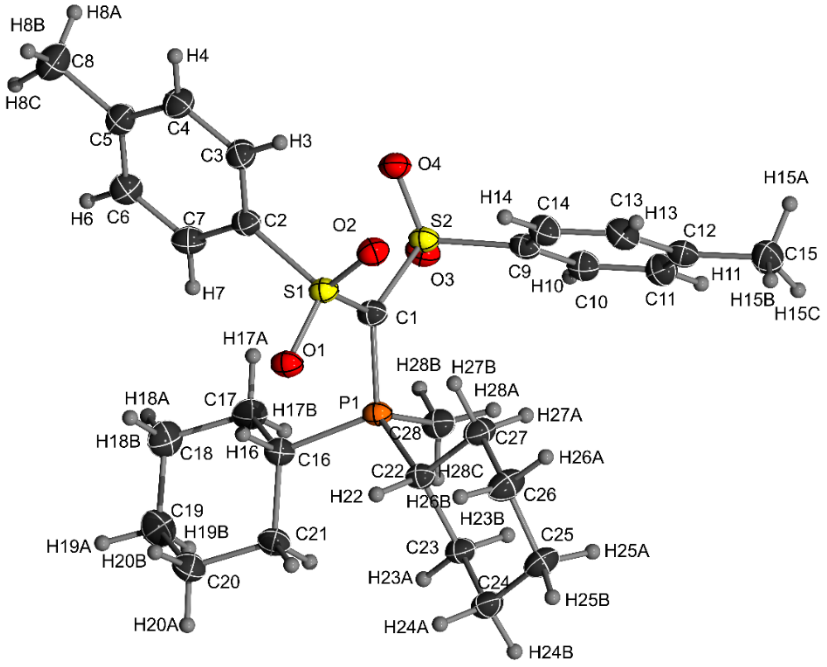

Figure S85. ORTEP plot of **5a**. Ellipsoids are drawn at 50 % probability level.

Table S30. Atomic coordinates (x 10^4) and equivalent isotropic displacement parameters ($Å^2$x 10^3) for **5a**. U(eq) is defined as one third of the trace of the orthogonalized Uij tensor

	x	y	z	U(eq)
S(1)	2836(1)	6001(1)	3882(1)	22(1)
O(1)	3799(1)	5691(1)	4552(1)	25(1)
C(1)	2139(2)	5048(1)	3473(1)	24(1)
P(1)	2831(1)	4042(1)	3810(1)	21(1)
S(2)	932(1)	5142(1)	2650(1)	23(1)
O(2)	3051(1)	6578(1)	3169(1)	27(1)
C(2)	2008(1)	6632(1)	4486(1)	24(1)
C(4)	1236(2)	8036(1)	4765(1)	31(1)
O(4)	562(1)	6046(1)	2676(1)	28(1)
C(3)	1822(2)	7519(1)	4267(1)	28(1)
O(3)	174(1)	4436(1)	2749(1)	28(1)
C(5)	834(2)	7680(1)	5487(1)	28(1)
C(6)	1036(2)	6786(1)	5706(1)	29(1)
C(7)	1617(2)	6261(1)	5208(1)	28(1)

C(9)	1251(2)	4984(1)	1553(1)	24(1)
C(8)	191(2)	8259(2)	6011(2)	37(1)
C(20)	3870(2)	3040(1)	6477(1)	30(1)
C(21)	3780(2)	3108(1)	5442(1)	27(1)
C(22)	4207(1)	3961(1)	3537(1)	23(1)
C(23)	4545(2)	2994(1)	3392(1)	26(1)
C(24)	5749(2)	2963(1)	3278(1)	29(1)
C(25)	5916(2)	3548(1)	2491(1)	33(1)
C(26)	5499(2)	4492(1)	2575(2)	36(1)
C(27)	4301(2)	4518(1)	2695(1)	28(1)
C(28)	2007(2)	3149(1)	3214(1)	28(1)
C(19)	2737(2)	2904(2)	6707(2)	36(1)
C(11)	1106(2)	4160(1)	156(1)	31(1)
C(10)	849(2)	4256(1)	1010(1)	28(1)
C(14)	1903(2)	5616(1)	1240(1)	29(1)
C(13)	2143(2)	5513(1)	384(1)	30(1)
C(12)	1755(2)	4784(1)	-175(1)	29(1)
C(16)	2963(1)	3849(1)	5038(1)	24(1)
C(15)	2028(2)	4687(2)	-1106(1)	37(1)
C(18)	1914(2)	3627(2)	6292(1)	35(1)
C(17)	1817(2)	3684(1)	5258(1)	30(1)

Table S31. Anisotropic displacement parameters (Å^2×10^3) for **5a**. The anisotropic displacement factor exponent takes the form: −2p^2[h^{2}a·^{2}U^{11} + ... + 2 h k a·b·U^{12}]

	U^{11}	U^{22}	U^{33}	U^{23}	U^{13}	U^{12}
S(1)	19(1)	19(1)	26(1)	0(1)	3(1)	0(1)
O(1)	19(1)	23(1)	31(1)	-1(1)	0(1)	0(1)
C(1)	21(1)	22(1)	27(1)	0(1)	2(1)	1(1)
P(1)	18(1)	19(1)	26(1)	0(1)	3(1)	0(1)
S(2)	18(1)	22(1)	26(1)	0(1)	1(1)	1(1)
O(2)	27(1)	23(1)	31(1)	1(1)	7(1)	-1(1)
C(2)	20(1)	23(1)	26(1)	-2(1)	1(1)	0(1)
C(4)	35(1)	23(1)	33(1)	-4(1)	5(1)	1(1)
O(4)	24(1)	26(1)	32(1)	0(1)	2(1)	6(1)
C(3)	34(1)	22(1)	27(1)	-1(1)	7(1)	0(1)
O(3)	20(1)	30(1)	34(1)	2(1)	2(1)	-2(1)
C(5)	20(1)	31(1)	31(1)	-9(1)	2(1)	-3(1)
C(6)	22(1)	32(1)	32(1)	0(1)	7(1)	-3(1)

C(7)	23(1)	24(1)	34(1)	2(1)	5(1)	0(1)
C(9)	20(1)	24(1)	25(1)	1(1)	-1(1)	0(1)
C(8)	33(1)	37(1)	43(1)	-11(1)	12(1)	-3(1)
C(20)	31(1)	28(1)	30(1)	5(1)	6(1)	1(1)
C(21)	26(1)	23(1)	30(1)	4(1)	5(1)	0(1)
C(22)	20(1)	21(1)	27(1)	0(1)	3(1)	1(1)
C(23)	25(1)	21(1)	30(1)	-1(1)	5(1)	3(1)
C(24)	24(1)	28(1)	35(1)	-4(1)	4(1)	5(1)
C(25)	30(1)	31(1)	41(1)	-3(1)	14(1)	5(1)
C(26)	37(1)	27(1)	47(1)	3(1)	21(1)	2(1)
C(27)	31(1)	23(1)	34(1)	2(1)	11(1)	5(1)
C(28)	24(1)	23(1)	35(1)	-3(1)	3(1)	-2(1)
C(19)	39(1)	36(1)	35(1)	7(1)	11(1)	-6(1)
C(11)	27(1)	31(1)	32(1)	-5(1)	0(1)	-2(1)
C(10)	23(1)	28(1)	31(1)	-1(1)	2(1)	-3(1)
C(14)	28(1)	26(1)	30(1)	0(1)	1(1)	-3(1)
C(13)	27(1)	31(1)	31(1)	4(1)	4(1)	-4(1)
C(12)	22(1)	34(1)	29(1)	1(1)	1(1)	5(1)
C(16)	21(1)	23(1)	27(1)	2(1)	5(1)	-2(1)
C(15)	31(1)	47(1)	31(1)	-2(1)	4(1)	3(1)
C(18)	29(1)	41(1)	38(1)	2(1)	14(1)	-4(1)
C(17)	22(1)	31(1)	36(1)	2(1)	8(1)	-2(1)

Crystal Structure Deterimination of 5b.

Additional information concerning the structure refinement: A slightly disorded THF molecule was solved using the DFIX instruction.

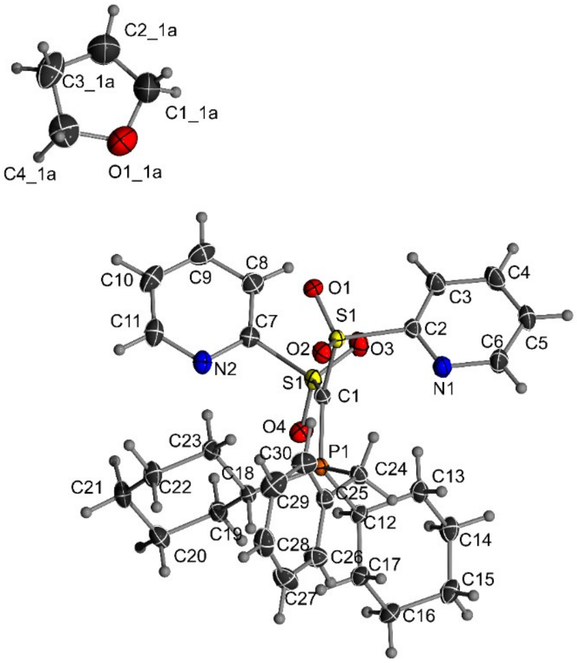

Figure S86. ORTEP plot of **5b**. Ellipsoids are drawn at 50 % probability level.

Table S32. Atomic coordinates (x 10^4) and equivalent isotropic displacement parameters ($Å^2$x 10^3) for **5b**. U(eq) is defined as one third of the trace of the orthogonalized U^{ij} tensor.

	x	y	z	U(eq)
S(1)	5929(1)	6941(1)	3345(1)	17(1)
P(1)	7008(1)	4876(1)	2761(1)	16(1)
O(1)	6168(1)	7951(1)	3797(1)	23(1)
N(1)	4614(1)	5235(2)	3229(1)	28(1)
C(1)	6824(1)	5906(2)	3383(1)	18(1)
S(2)	7522(1)	5868(1)	4099(1)	19(1)
O(2)	5615(1)	7270(1)	2693(1)	22(1)
N(2)	8592(1)	7697(2)	3791(1)	25(1)
C(2)	5020(1)	6097(2)	3628(1)	19(1)
O(3)	7011(1)	5719(1)	4620(1)	27(1)
C(3)	4800(1)	6349(2)	4221(1)	31(1)
C(4)	4100(2)	5649(2)	4416(1)	33(1)
O(4)	8230(1)	4957(1)	4044(1)	25(1)
C(5)	3665(1)	4745(2)	4013(1)	29(1)
C(6)	3942(2)	4568(2)	3426(1)	33(1)
C(7)	8079(1)	7384(2)	4228(1)	21(1)
C(8)	7992(1)	8088(2)	4765(1)	28(1)
C(9)	8478(2)	9218(2)	4852(1)	34(1)
C(10)	9014(1)	9575(2)	4408(1)	34(1)

C(13)	6602(1)	2766(2)	3459(1)	24(1)
C(12)	7306(1)	3270(2)	3061(1)	20(1)
C(11)	9052(1)	8792(2)	3887(1)	32(1)
C(14)	6895(1)	1454(2)	3735(1)	27(1)
C(15)	7052(1)	517(2)	3218(1)	29(1)
C(16)	7740(1)	1037(2)	2818(1)	30(1)
C(17)	7441(1)	2331(2)	2528(1)	26(1)
C(18)	7897(1)	5447(2)	2325(1)	19(1)
C(19)	8869(1)	5198(2)	2651(1)	25(1)
C(20)	9539(1)	5649(2)	2216(1)	33(1)
C(21)	9413(2)	7059(2)	2050(1)	35(1)
C(22)	8443(1)	7330(2)	1747(1)	32(1)
C(23)	7763(1)	6876(2)	2170(1)	23(1)
C(24)	5959(1)	4658(2)	2192(1)	21(1)
C(25)	5995(1)	5013(2)	1507(1)	21(1)
C(26)	6367(1)	4189(2)	1103(1)	28(1)
C(27)	6388(2)	4514(2)	473(1)	33(1)
C(28)	6030(2)	5668(2)	236(1)	34(1)
C(29)	5652(2)	6479(2)	635(1)	36(1)
C(30)	5632(1)	6159(2)	1265(1)	29(1)
O11	8913(2)	2094(2)	5406(1)	69(1)
C11	8446(3)	2560(3)	5882(2)	90(1)
C21	8924(3)	3707(4)	6157(2)	84(1)
C31	9336(2)	4255(3)	5599(1)	56(1)
C41	9362(2)	3133(3)	5152(1)	50(1)

Table S33. Anisotropic displacement parameters ($Å^2 \times 10^3$) for **5b**. The anisotropic displacement factor exponent takes the form: $-2p^2[h^2 a^{*2} U^{11} + ... + 2 h k a^* b^* U^{12}]$

	U^{11}	U^{22}	U^{33}	U^{23}	U^{13}	U^{12}
S(1)	19(1)	16(1)	17(1)	2(1)	5(1)	0(1)
P(1)	18(1)	16(1)	16(1)	1(1)	5(1)	0(1)
O(1)	26(1)	18(1)	25(1)	-3(1)	7(1)	-2(1)
N(1)	31(1)	30(1)	24(1)	-5(1)	10(1)	-10(1)
C(1)	18(1)	19(1)	17(1)	1(1)	5(1)	-1(1)
S(2)	22(1)	20(1)	15(1)	2(1)	2(1)	-2(1)
O(2)	25(1)	23(1)	19(1)	6(1)	4(1)	2(1)
N(2)	24(1)	25(1)	25(1)	4(1)	4(1)	-2(1)
C(2)	19(1)	18(1)	20(1)	3(1)	5(1)	1(1)
O(3)	33(1)	33(1)	16(1)	3(1)	6(1)	-7(1)
C(3)	34(1)	38(1)	23(1)	-6(1)	10(1)	-11(1)
C(4)	35(1)	42(1)	24(1)	0(1)	15(1)	-7(1)
O(4)	26(1)	23(1)	25(1)	2(1)	-2(1)	3(1)
C(5)	26(1)	32(1)	31(1)	7(1)	10(1)	-5(1)
C(6)	34(1)	33(1)	33(1)	-5(1)	11(1)	-14(1)

C(7)	20(1)	22(1)	19(1)	2(1)	0(1)	0(1)
C(8)	27(1)	31(1)	25(1)	-4(1)	2(1)	-3(1)
C(9)	36(1)	29(1)	34(1)	-8(1)	-3(1)	-1(1)
C(10)	31(1)	24(1)	42(1)	4(1)	-8(1)	-5(1)
C(13)	27(1)	22(1)	23(1)	5(1)	7(1)	-1(1)
C(12)	24(1)	16(1)	22(1)	3(1)	6(1)	1(1)
C(11)	29(1)	29(1)	36(1)	9(1)	3(1)	-6(1)
C(14)	31(1)	24(1)	27(1)	10(1)	5(1)	-2(1)
C(15)	32(1)	20(1)	34(1)	6(1)	4(1)	-2(1)
C(16)	36(1)	17(1)	38(1)	2(1)	11(1)	1(1)
C(17)	33(1)	18(1)	28(1)	2(1)	11(1)	0(1)
C(18)	20(1)	18(1)	20(1)	0(1)	7(1)	-1(1)
C(19)	20(1)	23(1)	31(1)	3(1)	7(1)	2(1)
C(20)	25(1)	37(1)	40(1)	1(1)	14(1)	0(1)
C(21)	30(1)	36(1)	43(1)	4(1)	18(1)	-10(1)
C(22)	36(1)	31(1)	30(1)	8(1)	12(1)	-6(1)
C(23)	25(1)	20(1)	24(1)	4(1)	7(1)	1(1)
C(24)	22(1)	23(1)	18(1)	0(1)	5(1)	-2(1)
C(25)	21(1)	26(1)	16(1)	0(1)	3(1)	-4(1)
C(26)	32(1)	28(1)	22(1)	-1(1)	5(1)	2(1)
C(27)	35(1)	46(1)	20(1)	-5(1)	7(1)	0(1)
C(28)	38(1)	44(1)	18(1)	5(1)	3(1)	-11(1)
C(29)	50(1)	31(1)	23(1)	5(1)	-4(1)	-1(1)
C(30)	34(1)	29(1)	21(1)	1(1)	1(1)	4(1)
O11	72(1)	50(1)	89(2)	-21(1)	28(1)	-15(1)
C11	95(3)	55(2)	138(4)	-21(2)	74(3)	-22(2)
C21	101(3)	70(2)	91(3)	-28(2)	49(2)	-23(2)
C31	53(2)	43(2)	66(2)	15(1)	-9(1)	-7(1)
C41	50(2)	55(2)	43(1)	15(1)	0(1)	6(1)

3.11 Crystal Structure Determination of 6a and 6b

Crystal Structure Determination of 6a

Additional information concerning the structure refinement: All hydrogen atoms were placed on ideal positions except for H2, H78 and H79 on B1 and H5, H80 and H81 on B2 which were found in the difference Fourier map and refined independently.

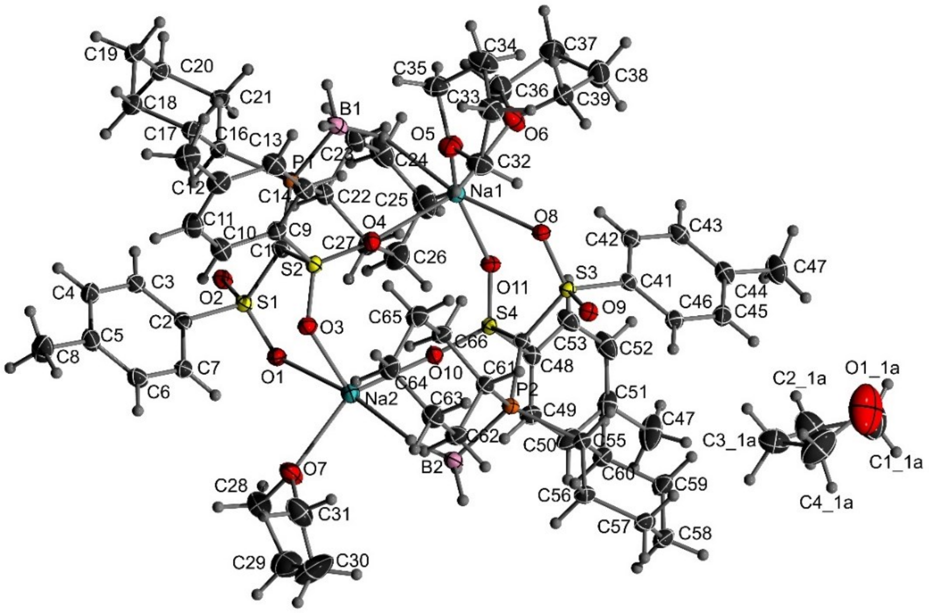

Figure S87. ORTEP plot of **6a**. Ellipsoids are drawn at 50 % probability level.

Table S34. Atomic coordinates (× 10^4) and equivalent isotropic displacement parameters ($Å^2$× 10^3)for 6a U(eq) is defined as one third of the trace of the orthogonalized U^{ij} tensor.

	x	y	z	U(eq)
S(4)	3724(1)	7136(1)	2026(1)	15(1)
O(4)	4913(1)	7884(1)	984(1)	21(1)
S(1)	7544(1)	6897(1)	1343(1)	17(1)
P(1)	7219(1)	8378(1)	1469(1)	17(1)
Na(1)	4009(1)	8593(1)	1398(1)	20(1)
O(1)	6971(1)	6356(1)	1516(1)	22(1)
C(1)	6839(1)	7599(1)	1185(1)	18(1)
B(1)	6300(1)	9110(1)	1269(1)	23(1)
C(61)	1924(1)	6525(1)	647(1)	20(1)
C(62)	1882(1)	5908(1)	312(1)	24(1)
C(63)	1712(1)	6102(1)	-233(1)	27(1)
C(2)	7966(1)	6595(1)	802(1)	20(1)
B(2)	3155(1)	5573(1)	1367(1)	22(1)
C(3)	8757(1)	6925(1)	631(1)	21(1)
C(4)	9026(1)	6736(1)	183(1)	25(1)
C(64)	2544(1)	6565(1)	-349(1)	28(1)
S(3)	1818(1)	7739(1)	1560(1)	15(1)

O(3)	5524(1)	6806(1)	722(1)	21(1)
C(5)	8517(1)	6224(1)	-97(1)	26(1)
C(6)	7757(1)	5886(1)	94(1)	26(1)
C(7)	7474(1)	6066(1)	541(1)	23(1)
C(8)	8781(2)	6045(1)	-594(1)	40(1)
C(9)	5784(1)	7839(1)	211(1)	20(1)
C(10)	6394(1)	7526(1)	-87(1)	26(1)
C(11)	6421(1)	7759(1)	-558(1)	29(1)
C(12)	5850(1)	8308(1)	-743(1)	27(1)
C(13)	5254(1)	8617(1)	-437(1)	28(1)
C(14)	5207(1)	8387(1)	36(1)	24(1)
S(2)	5707(1)	7513(1)	805(1)	17(1)
P(2)	2196(1)	6308(1)	1313(1)	16(1)
Na(2)	5276(1)	6108(1)	1354(1)	23(1)
O(2)	8430(1)	7108(1)	1679(1)	21(1)
C(48)	3565(1)	6865(1)	2625(1)	18(1)
C(49)	3507(1)	6189(1)	2718(1)	22(1)
C(15)	5875(2)	8552(1)	-1259(1)	35(1)
C(16)	8478(1)	8584(1)	1310(1)	18(1)
C(17)	8361(1)	8764(1)	760(1)	22(1)
C(50)	3273(1)	5975(1)	3168(1)	28(1)
C(18)	9383(1)	8942(1)	608(1)	27(1)
C(19)	9876(1)	9518(1)	921(1)	28(1)
C(51)	3098(1)	6427(1)	3528(1)	31(1)
C(20)	10015(1)	9339(1)	1465(1)	24(1)
C(21)	9015(1)	9145(1)	1631(1)	21(1)
C(42)	1878(1)	8408(1)	2440(1)	21(1)
C(22)	7377(1)	8270(1)	2146(1)	21(1)
C(41)	1392(1)	7927(1)	2125(1)	18(1)
C(23)	7325(1)	8934(1)	2422(1)	27(1)
C(24)	7466(2)	8821(1)	2978(1)	38(1)
C(25)	6674(2)	8349(1)	3118(1)	45(1)
C(43)	1531(1)	8536(1)	2881(1)	25(1)
C(26)	6681(2)	7692(1)	2840(1)	41(1)
C(27)	6567(1)	7796(1)	2284(1)	29(1)
C(47)	355(2)	8320(1)	3500(1)	36(1)
C(28)	6456(2)	4662(1)	1227(1)	35(1)
C(36)	4558(2)	9827(1)	2236(1)	38(1)
C(29)	5902(2)	4043(1)	1349(1)	53(1)
C(37)	4054(2)	10343(1)	2523(1)	39(1)

C(30)	4919(3)	4060(2)	979(1)	73(1)
C(38)	2928(2)	10204(1)	2370(1)	39(1)
C(31)	5013(2)	4686(1)	676(1)	42(1)
C(39)	2884(1)	9921(1)	1857(1)	28(1)
C(32)	2391(2)	9023(1)	361(1)	32(1)
C(44)	710(1)	8193(1)	3014(1)	25(1)
C(45)	221(1)	7724(1)	2686(1)	24(1)
C(46)	556(1)	7590(1)	2244(1)	20(1)
O(10)	4470(1)	6696(1)	1874(1)	19(1)
C(52)	3208(2)	7099(1)	3437(1)	34(1)
O(11)	3974(1)	7836(1)	2081(1)	19(1)
C(53)	3444(1)	7324(1)	2989(1)	26(1)
C(54)	2784(2)	6187(1)	4002(1)	47(1)
C(55)	1003(1)	6056(1)	1529(1)	18(1)
O(6)	3783(1)	9534(1)	1887(1)	37(1)
C(56)	443(1)	5471(1)	1250(1)	22(1)
O(9)	932(1)	7547(1)	1216(1)	19(1)
C(57)	-492(1)	5275(1)	1477(1)	25(1)
O(8)	2336(1)	8332(1)	1433(1)	19(1)
C(58)	-224(1)	5110(1)	2022(1)	28(1)
C(59)	306(1)	5699(1)	2299(1)	28(1)
C(60)	1249(1)	5899(1)	2082(1)	22(1)
C(65)	2587(1)	7185(1)	-28(1)	27(1)
C(66)	2740(1)	7009(1)	520(1)	22(1)
C(40)	2599(1)	7056(1)	1639(1)	16(1)
O(7)	5685(1)	5092(1)	997(1)	34(1)
C(33)	2048(2)	9671(1)	103(1)	31(1)
C(35)	3687(2)	9802(1)	548(1)	35(1)
O(5)	3360(1)	9148(1)	655(1)	32(1)
C(34)	2700(2)	10183(1)	396(1)	40(1)
O11	-1458(2)	6918(2)	3754(1)	91(1)
C11	-998(2)	6804(2)	4229(1)	72(1)
C21	60(2)	6548(2)	4208(1)	61(1)
C31	39(2)	6370(1)	3670(1)	45(1)
C41	-1061(2)	6408(2)	3459(1)	64(1)

Table S35. Anisotropic displacement parameters ($Å^2 \times 10^3$) for **6a**. The anisotropic displacement factor exponent takes the form: $-2p^2[\, h^2\, a^{\cdot 2}U^{11} + \ldots + 2\, h\, k\, a\cdot b\cdot U^{12}\,]$

	U^{11}	U^{22}	U^{33}	U^{23}	U^{13}	U^{12}
S(4)	15(1)	14(1)	16(1)	0(1)	2(1)	0(1)

O(4)	17(1)	21(1)	25(1)	-2(1)	4(1)	2(1)
S(1)	16(1)	15(1)	21(1)	-1(1)	2(1)	1(1)
P(1)	16(1)	16(1)	19(1)	-2(1)	2(1)	0(1)
Na(1)	19(1)	17(1)	26(1)	1(1)	6(1)	1(1)
O(1)	21(1)	18(1)	27(1)	2(1)	5(1)	0(1)
C(1)	17(1)	16(1)	20(1)	-2(1)	1(1)	1(1)
B(1)	21(1)	19(1)	30(1)	-4(1)	0(1)	4(1)
C(61)	20(1)	21(1)	18(1)	-1(1)	3(1)	-2(1)
C(62)	32(1)	22(1)	20(1)	-4(1)	5(1)	-6(1)
C(63)	31(1)	31(1)	20(1)	-6(1)	4(1)	-4(1)
C(2)	18(1)	17(1)	24(1)	-1(1)	2(1)	3(1)
B(2)	22(1)	16(1)	26(1)	-3(1)	4(1)	1(1)
C(3)	17(1)	20(1)	27(1)	-4(1)	1(1)	-1(1)
C(4)	20(1)	26(1)	31(1)	-5(1)	7(1)	-2(1)
C(64)	34(1)	32(1)	19(1)	-3(1)	8(1)	-2(1)
S(3)	15(1)	14(1)	16(1)	0(1)	2(1)	0(1)
O(3)	19(1)	18(1)	27(1)	-3(1)	2(1)	-2(1)
C(5)	22(1)	27(1)	30(1)	-9(1)	7(1)	1(1)
C(6)	23(1)	21(1)	35(1)	-10(1)	4(1)	-2(1)
C(7)	20(1)	18(1)	31(1)	-4(1)	6(1)	-1(1)
C(8)	37(1)	46(1)	39(1)	-21(1)	16(1)	-11(1)
C(9)	17(1)	22(1)	21(1)	-3(1)	0(1)	-3(1)
C(10)	26(1)	28(1)	25(1)	-3(1)	3(1)	4(1)
C(11)	29(1)	33(1)	23(1)	-5(1)	6(1)	1(1)
C(12)	29(1)	32(1)	20(1)	-3(1)	1(1)	-6(1)
C(13)	30(1)	29(1)	24(1)	3(1)	0(1)	4(1)
C(14)	24(1)	25(1)	25(1)	-1(1)	3(1)	3(1)
S(2)	15(1)	16(1)	20(1)	-2(1)	2(1)	0(1)
P(2)	17(1)	14(1)	17(1)	-1(1)	3(1)	-1(1)
Na(2)	22(1)	20(1)	30(1)	-4(1)	9(1)	-1(1)
O(2)	18(1)	20(1)	23(1)	-1(1)	0(1)	1(1)
C(48)	16(1)	21(1)	17(1)	2(1)	1(1)	3(1)
C(49)	24(1)	22(1)	21(1)	2(1)	1(1)	0(1)
C(15)	42(1)	39(1)	23(1)	0(1)	4(1)	-1(1)
C(16)	17(1)	17(1)	21(1)	-1(1)	3(1)	1(1)
C(17)	24(1)	21(1)	22(1)	0(1)	3(1)	0(1)
C(50)	28(1)	29(1)	27(1)	9(1)	2(1)	-3(1)
C(18)	30(1)	25(1)	27(1)	2(1)	10(1)	1(1)
C(19)	26(1)	24(1)	35(1)	5(1)	9(1)	-2(1)
C(51)	26(1)	45(1)	23(1)	11(1)	7(1)	6(1)

C(20)	20(1)	21(1)	32(1)	0(1)	2(1)	-1(1)
C(21)	19(1)	19(1)	25(1)	-2(1)	2(1)	-2(1)
C(42)	20(1)	19(1)	25(1)	-2(1)	2(1)	1(1)
C(22)	18(1)	25(1)	20(1)	-3(1)	3(1)	2(1)
C(41)	17(1)	16(1)	19(1)	1(1)	2(1)	3(1)
C(23)	29(1)	30(1)	24(1)	-8(1)	6(1)	3(1)
C(24)	44(1)	46(1)	24(1)	-11(1)	2(1)	10(1)
C(25)	57(1)	57(1)	23(1)	4(1)	16(1)	15(1)
C(43)	26(1)	23(1)	24(1)	-6(1)	0(1)	3(1)
C(26)	53(1)	45(1)	29(1)	8(1)	16(1)	3(1)
C(27)	30(1)	34(1)	25(1)	2(1)	9(1)	-5(1)
C(47)	40(1)	44(1)	25(1)	-5(1)	11(1)	5(1)
C(28)	34(1)	35(1)	35(1)	-2(1)	6(1)	6(1)
C(36)	26(1)	46(1)	39(1)	-13(1)	2(1)	-4(1)
C(29)	74(2)	41(1)	47(1)	10(1)	21(1)	0(1)
C(37)	38(1)	37(1)	43(1)	-15(1)	10(1)	-10(1)
C(30)	79(2)	65(2)	76(2)	-2(2)	17(2)	-44(2)
C(38)	34(1)	44(1)	42(1)	-11(1)	14(1)	-1(1)
C(31)	41(1)	41(1)	42(1)	-13(1)	1(1)	8(1)
C(39)	25(1)	26(1)	34(1)	1(1)	7(1)	3(1)
C(32)	40(1)	28(1)	26(1)	3(1)	2(1)	2(1)
C(44)	27(1)	27(1)	21(1)	0(1)	5(1)	8(1)
C(45)	22(1)	26(1)	26(1)	3(1)	7(1)	3(1)
C(46)	18(1)	20(1)	21(1)	0(1)	3(1)	1(1)
O(10)	17(1)	19(1)	21(1)	-1(1)	4(1)	2(1)
C(52)	44(1)	37(1)	21(1)	2(1)	9(1)	17(1)
O(11)	19(1)	15(1)	21(1)	0(1)	1(1)	-2(1)
C(53)	34(1)	23(1)	22(1)	2(1)	4(1)	9(1)
C(54)	41(1)	72(2)	30(1)	20(1)	14(1)	10(1)
C(55)	18(1)	17(1)	21(1)	0(1)	4(1)	-2(1)
O(6)	23(1)	31(1)	55(1)	-18(1)	0(1)	2(1)
C(56)	22(1)	20(1)	24(1)	-3(1)	3(1)	-4(1)
O(9)	17(1)	19(1)	19(1)	0(1)	-1(1)	0(1)
C(57)	21(1)	21(1)	34(1)	0(1)	2(1)	-5(1)
O(8)	19(1)	15(1)	23(1)	2(1)	4(1)	0(1)
C(58)	26(1)	24(1)	35(1)	5(1)	9(1)	-6(1)
C(59)	30(1)	29(1)	26(1)	1(1)	10(1)	-6(1)
C(60)	22(1)	22(1)	21(1)	1(1)	4(1)	-3(1)
C(65)	34(1)	27(1)	21(1)	1(1)	7(1)	-3(1)
C(66)	26(1)	20(1)	19(1)	-1(1)	5(1)	-4(1)

C(40)	17(1)	15(1)	17(1)	-1(1)	2(1)	0(1)
O(7)	28(1)	26(1)	46(1)	-11(1)	3(1)	4(1)
C(33)	40(1)	30(1)	24(1)	4(1)	4(1)	7(1)
C(35)	39(1)	25(1)	42(1)	9(1)	7(1)	1(1)
O(5)	29(1)	28(1)	39(1)	13(1)	6(1)	4(1)
C(34)	49(1)	30(1)	38(1)	2(1)	-1(1)	7(1)
O11	51(1)	139(2)	81(2)	14(2)	1(1)	22(1)
C11	64(2)	93(2)	58(2)	4(2)	9(1)	20(2)
C21	57(2)	71(2)	51(1)	-8(1)	-2(1)	16(1)
C31	51(1)	34(1)	47(1)	4(1)	4(1)	2(1)
C41	52(2)	81(2)	57(2)	7(1)	0(1)	-26(1)

Crystal Structure Determination of 6b

Additional information concerning the structure refinement: The structure contains an inversion twin (ratio 0.47:0.53) which was solved using the TWIN and BASF instructions.

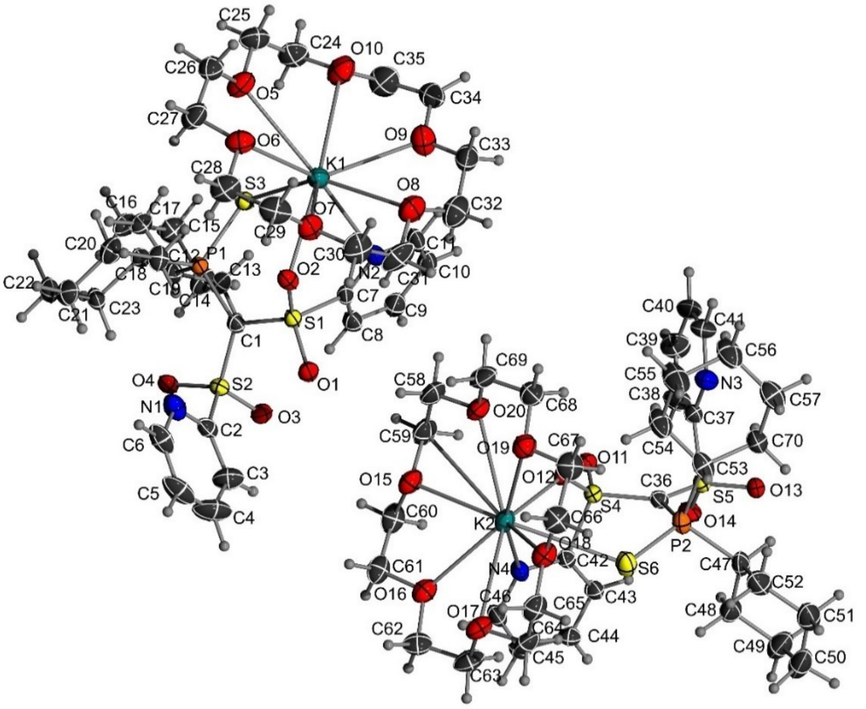

Figure S88. ORTEP plot of **6b**. Ellipsoids are drawn at 50 % probability level.

Table S36. Atomic coordinates (× 10^4) and equivalent isotropic displacement parameters (Å^2× 10^3) for **6b**. U(eq) is defined as one third of the trace of the orthogonalized U^{ij} tensor.

	x	y	z	U(eq)
K(1)	3732(1)	-1053(1)	6197(1)	34(1)
O(1)	2414(1)	1707(3)	5425(1)	31(1)

S(1)	2607(1)	1320(1)	5827(1)	24(1)
C(1)	2015(2)	1689(3)	6165(1)	23(1)
P(1)	2026(1)	1189(1)	6679(1)	22(1)
N(1)	800(2)	987(3)	5619(1)	38(1)
K(2)	3447(1)	3840(1)	3815(1)	31(1)
N(2)	3878(2)	1522(3)	5971(1)	33(1)
P(2)	5288(1)	5646(1)	3396(1)	29(1)
O(2)	2779(1)	175(2)	5860(1)	28(1)
S(2)	1452(1)	2601(1)	5982(1)	24(1)
C(2)	1028(2)	1991(4)	5555(1)	29(1)
S(3)	2905(1)	679(1)	6838(1)	28(1)
N(3)	6523(2)	5852(3)	4463(1)	36(1)
O(3)	1733(1)	3603(2)	5822(1)	32(1)
C(3)	914(3)	2609(5)	5206(2)	46(1)
C(37)	6195(2)	6792(3)	4504(1)	27(1)
O(4)	962(1)	2742(2)	6296(1)	26(1)
S(4)	4613(1)	6163(1)	4202(1)	21(1)
N(4)	3364(2)	6517(3)	4046(1)	32(1)
C(4)	513(4)	2139(6)	4912(2)	63(2)
C(5)	273(3)	1108(5)	4967(2)	52(2)
O(5)	3544(2)	-2386(3)	6981(1)	45(1)
S(5)	5822(1)	7286(1)	4038(1)	23(1)
C(6)	431(2)	559(5)	5319(2)	43(1)
O(6)	3101(2)	-3392(3)	6261(1)	43(1)
S(6)	4423(1)	5134(1)	3206(1)	38(1)
O(9)	5069(2)	-451(3)	6134(1)	50(1)
C(9)	3831(2)	3808(4)	6079(1)	37(1)
C(8)	3268(2)	3219(4)	6006(1)	32(1)
O(8)	4451(2)	-1233(4)	5436(1)	53(1)
C(23)	731(2)	410(4)	6724(2)	32(1)
C(7)	3330(2)	2091(3)	5955(1)	26(1)
O(7)	3260(2)	-2344(3)	5505(1)	44(1)
O(16)	2198(2)	4786(2)	3904(1)	36(1)
O(13)	6334(1)	7298(2)	3736(1)	27(1)
C(13)	2210(2)	3292(3)	6989(1)	30(1)
C(70)	6567(2)	4788(4)	3440(2)	38(1)
O(14)	5550(1)	8341(2)	4146(1)	29(1)
C(14)	2075(3)	4163(4)	7317(2)	39(1)

C(16)	1622(2)	2746(4)	7789(1)	34(1)
C(18)	1446(2)	43(3)	6738(1)	26(1)
O(18)	3559(2)	2605(3)	3020(1)	35(1)
C(17)	1762(2)	1843(4)	7477(1)	29(1)
O(17)	2458(2)	3961(3)	3121(1)	37(1)
O(10)	4695(2)	-1164(3)	6914(1)	50(1)
C(10)	4414(2)	3234(5)	6099(2)	40(1)
C(22)	282(2)	-586(4)	6768(2)	39(1)
C(12)	1765(2)	2284(3)	7035(1)	25(1)
O(12)	4389(1)	5037(2)	4199(1)	26(1)
C(11)	4416(2)	2110(4)	6044(2)	37(1)
O(11)	4791(1)	6615(2)	4598(1)	28(1)
C(15)	2112(3)	3679(4)	7748(2)	36(1)
O(15)	2763(2)	3755(3)	4574(1)	36(1)
C(19)	1582(2)	-835(4)	6412(2)	33(1)
C(21)	412(2)	-1433(4)	6431(2)	39(1)
C(20)	1124(2)	-1803(4)	6453(2)	38(1)
O(20)	3892(2)	2445(3)	4475(1)	37(1)
C(24)	4326(3)	-1196(5)	7268(2)	50(1)
C(26)	3313(3)	-3460(4)	6976(2)	42(1)
C(25)	3999(3)	-2245(5)	7314(2)	48(1)
C(27)	2810(3)	-3571(5)	6649(2)	48(1)
C(29)	2984(3)	-3399(5)	5538(2)	50(1)
C(30)	3592(3)	-2263(6)	5124(2)	56(2)
C(32)	4908(3)	-425(5)	5407(2)	58(2)
C(40)	6844(2)	6053(5)	5166(2)	46(1)
C(41)	6835(2)	5504(5)	4796(2)	43(1)
C(42)	3939(2)	7000(3)	4034(1)	24(1)
C(43)	4045(2)	8096(3)	3936(1)	26(1)
C(44)	3506(2)	8735(3)	3854(1)	28(1)
C(45)	2895(2)	8266(3)	3875(1)	29(1)
C(46)	2853(2)	7157(4)	3968(2)	36(1)
C(47)	5635(2)	6530(4)	3001(1)	36(1)
C(48)	5252(3)	7589(4)	2957(2)	41(1)
C(49)	5538(3)	8294(6)	2618(2)	56(2)
C(50)	5588(3)	7675(6)	2208(2)	58(2)
C(51)	5953(3)	6602(7)	2257(2)	58(2)
C(52)	5660(3)	5898(5)	2590(2)	47(1)

C(53)	5844(2)	4481(4)	3434(2)	36(1)
C(54)	5676(2)	3773(4)	3800(2)	46(1)
C(55)	6094(3)	2737(5)	3805(3)	66(2)
C(56)	6821(3)	3042(4)	3823(2)	53(2)
C(57)	6996(3)	3769(4)	3461(2)	47(1)
C(58)	3549(3)	2551(5)	4852(2)	46(1)
C(59)	3250(3)	3664(5)	4881(2)	44(1)
C(60)	2376(3)	4719(4)	4622(2)	40(1)
C(61)	1879(3)	4752(4)	4293(2)	41(1)
C(62)	1773(3)	4981(4)	3567(2)	42(1)
C(63)	2175(3)	5017(4)	3187(2)	46(1)
C(64)	2835(3)	3937(4)	2755(2)	44(1)
C(65)	3109(3)	2812(4)	2702(1)	40(1)
C(66)	3882(3)	1595(4)	2975(2)	43(1)
C(67)	4379(3)	1484(5)	3308(2)	46(1)
C(68)	4489(2)	1284(4)	4024(2)	40(1)
C(69)	4117(3)	1349(4)	4415(2)	44(1)
C(31)	3984(4)	-1243(5)	5120(2)	58(2)
C(34)	5469(3)	-331(4)	6480(2)	47(1)
O(19)	4059(2)	1473(3)	3690(1)	38(1)
C(33)	5384(3)	-522(5)	5758(2)	49(1)
C(36)	5249(2)	6365(3)	3878(1)	23(1)
C(35)	5094(3)	-250(5)	6852(2)	59(2)
C(39)	6495(3)	7018(6)	5197(2)	53(1)
C(38)	6155(3)	7402(4)	4857(2)	40(1)
C(28)	2634(3)	-3486(5)	5938(2)	51(1)

Table S37. Anisotropic displacement parameters ($Å^2$x 10^3) for **6b**. The anisotropic displacement factor exponent takes the form: $-2p^2[h^2 a^{*2}U^{11} + ... + 2 h k a^* b^* U^{12}]$

	U^{11}	U^{22}	U^{33}	U^{23}	U^{13}	U^{12}
K(1)	31(1)	44(1)	27(1)	2(1)	-1(1)	2(1)
O(1)	27(1)	45(2)	20(1)	0(1)	-1(1)	-2(1)
S(1)	19(1)	34(1)	20(1)	-1(1)	0(1)	0(1)
C(1)	18(2)	29(2)	22(2)	-1(2)	-2(1)	-1(1)
P(1)	19(1)	25(1)	21(1)	0(1)	0(1)	0(1)
N(1)	30(2)	45(2)	38(2)	-10(2)	-3(2)	-5(2)
K(2)	34(1)	34(1)	25(1)	0(1)	1(1)	-7(1)

N(2)	22(2)	49(2)	29(2)	5(2)	1(1)	0(2)
P(2)	25(1)	34(1)	27(1)	-5(1)	4(1)	-4(1)
O(2)	23(1)	34(1)	27(1)	-5(1)	-1(1)	3(1)
S(2)	21(1)	28(1)	23(1)	2(1)	-3(1)	1(1)
C(2)	22(2)	39(2)	27(2)	-4(2)	-1(2)	6(2)
S(3)	22(1)	38(1)	25(1)	2(1)	-2(1)	3(1)
N(3)	32(2)	40(2)	36(2)	6(2)	-3(2)	7(2)
O(3)	30(2)	31(1)	34(2)	9(1)	-3(1)	-1(1)
C(3)	55(3)	47(3)	38(3)	2(2)	-13(2)	4(2)
C(37)	21(2)	33(2)	28(2)	4(2)	-3(2)	-4(2)
O(4)	22(1)	31(1)	28(1)	-2(1)	-1(1)	4(1)
S(4)	18(1)	23(1)	22(1)	2(1)	1(1)	1(1)
N(4)	21(2)	31(2)	44(2)	7(2)	3(2)	1(1)
C(4)	77(4)	75(4)	38(3)	-4(3)	-25(3)	22(4)
C(5)	36(3)	71(4)	50(3)	-30(3)	-18(2)	12(2)
O(5)	58(2)	40(2)	36(2)	4(1)	-3(2)	-13(2)
S(5)	19(1)	24(1)	26(1)	2(1)	-2(1)	-1(1)
C(6)	28(2)	55(3)	46(3)	-23(2)	-5(2)	-3(2)
O(6)	52(2)	37(2)	40(2)	-2(2)	-9(2)	2(2)
S(6)	28(1)	54(1)	32(1)	-2(1)	-1(1)	-8(1)
O(9)	42(2)	63(2)	46(2)	-10(2)	5(2)	-9(2)
C(9)	35(2)	46(3)	28(2)	1(2)	-2(2)	-11(2)
C(8)	23(2)	45(2)	27(2)	1(2)	-2(2)	-2(2)
O(8)	55(2)	70(3)	34(2)	4(2)	4(2)	-1(2)
C(23)	19(2)	32(2)	43(3)	4(2)	3(2)	-1(2)
C(7)	19(2)	38(2)	22(2)	3(2)	1(2)	-4(2)
O(7)	44(2)	61(2)	27(2)	-5(2)	1(2)	-1(2)
O(16)	45(2)	29(1)	34(2)	0(1)	3(1)	-1(1)
O(13)	20(1)	32(1)	30(2)	6(1)	1(1)	-2(1)
C(13)	36(2)	30(2)	25(2)	2(2)	1(2)	-4(2)
C(70)	27(2)	43(2)	43(3)	4(2)	2(2)	1(2)
O(14)	27(1)	22(1)	39(2)	-1(1)	-4(1)	1(1)
C(14)	51(3)	27(2)	38(3)	-3(2)	3(2)	-5(2)
C(16)	43(2)	35(2)	23(2)	-2(2)	6(2)	2(2)
C(18)	23(2)	26(2)	28(2)	1(2)	0(2)	0(1)
O(18)	44(2)	32(2)	30(2)	-3(1)	1(1)	-6(1)
C(17)	35(2)	29(2)	25(2)	-2(2)	1(2)	0(2)
O(17)	51(2)	29(1)	30(2)	4(1)	1(2)	2(1)

O(10)	62(2)	45(2)	45(2)	3(2)	-4(2)	-18(2)
C(10)	28(2)	61(3)	29(2)	0(2)	-1(2)	-13(2)
C(22)	26(2)	38(2)	52(3)	7(2)	4(2)	-7(2)
C(12)	24(2)	27(2)	23(2)	-2(2)	2(2)	-2(2)
O(12)	24(1)	23(1)	30(2)	6(1)	1(1)	-2(1)
C(11)	19(2)	58(3)	33(2)	1(2)	-1(2)	-7(2)
O(11)	22(1)	37(2)	24(1)	2(1)	0(1)	0(1)
C(15)	48(3)	32(2)	29(2)	-10(2)	-1(2)	-2(2)
O(15)	47(2)	35(2)	25(2)	-1(1)	2(1)	-5(1)
C(19)	33(2)	30(2)	35(2)	-3(2)	6(2)	-4(2)
C(21)	38(2)	35(2)	45(3)	5(2)	-6(2)	-14(2)
C(20)	45(3)	29(2)	38(2)	-2(2)	4(2)	-8(2)
O(20)	46(2)	38(2)	28(2)	8(1)	1(1)	-6(1)
C(24)	55(3)	62(3)	32(3)	-4(2)	-11(2)	-1(3)
C(26)	44(3)	39(2)	44(3)	5(2)	3(2)	4(2)
C(25)	55(3)	52(3)	37(3)	3(2)	-8(2)	6(3)
C(27)	56(3)	46(3)	42(3)	10(2)	-2(2)	-16(2)
C(29)	60(3)	43(3)	46(3)	-16(2)	-9(3)	5(2)
C(30)	65(4)	70(4)	32(3)	-14(3)	5(3)	-2(3)
C(32)	67(4)	56(3)	51(3)	-2(3)	15(3)	0(3)
C(40)	33(2)	64(3)	40(3)	16(2)	-12(2)	-3(2)
C(41)	35(2)	51(3)	44(3)	15(2)	-6(2)	12(2)
C(42)	21(2)	26(2)	24(2)	-1(2)	1(2)	1(1)
C(43)	22(2)	27(2)	30(2)	1(2)	-2(2)	-2(1)
C(44)	30(2)	24(2)	30(2)	1(2)	-4(2)	3(2)
C(45)	26(2)	27(2)	35(2)	2(2)	-3(2)	6(2)
C(46)	19(2)	36(2)	51(3)	7(2)	0(2)	1(2)
C(47)	24(2)	60(3)	25(2)	0(2)	4(2)	-5(2)
C(48)	37(2)	54(3)	33(2)	9(2)	0(2)	-8(2)
C(49)	60(3)	67(4)	40(3)	16(3)	-8(3)	-27(3)
C(50)	55(3)	83(4)	35(3)	13(3)	-5(3)	-34(3)
C(51)	39(3)	106(5)	28(3)	4(3)	1(2)	-6(3)
C(52)	39(3)	73(4)	30(2)	-2(2)	-1(2)	5(2)
C(53)	27(2)	37(2)	44(3)	-16(2)	2(2)	2(2)
C(54)	31(2)	31(2)	77(4)	1(2)	8(3)	2(2)
C(55)	39(3)	35(3)	124(6)	13(3)	7(3)	5(2)
C(56)	36(3)	38(3)	86(5)	2(3)	6(3)	9(2)
C(57)	35(2)	47(3)	58(3)	-13(2)	5(2)	10(2)

C(58)	52(3)	62(3)	26(2)	14(2)	1(2)	-4(2)
C(59)	50(3)	59(3)	23(2)	1(2)	3(2)	-8(2)
C(60)	50(3)	35(2)	35(2)	-10(2)	11(2)	-6(2)
C(61)	48(3)	34(2)	40(3)	-4(2)	12(2)	-1(2)
C(62)	52(3)	29(2)	45(3)	1(2)	-6(2)	7(2)
C(63)	67(3)	27(2)	42(3)	9(2)	-4(3)	3(2)
C(64)	58(3)	46(3)	26(2)	11(2)	-4(2)	-9(2)
C(65)	50(3)	46(3)	23(2)	-5(2)	2(2)	-8(2)
C(66)	50(3)	41(2)	38(3)	-12(2)	2(2)	2(2)
C(67)	50(3)	43(3)	45(3)	-8(2)	4(2)	1(2)
C(68)	39(2)	30(2)	52(3)	5(2)	-1(2)	-3(2)
C(69)	55(3)	36(2)	41(3)	14(2)	-6(2)	-6(2)
C(31)	91(5)	60(3)	23(2)	1(2)	-1(3)	-9(3)
C(34)	38(2)	34(2)	68(4)	10(2)	-9(3)	-7(2)
O(19)	40(2)	35(2)	38(2)	1(1)	0(1)	-3(1)
C(33)	43(3)	47(3)	58(3)	13(3)	13(3)	4(2)
C(36)	20(2)	25(2)	24(2)	-1(1)	0(1)	3(1)
C(35)	64(4)	58(3)	54(3)	-5(3)	-12(3)	-18(3)
C(39)	52(3)	75(4)	31(3)	-3(3)	-9(2)	1(3)
C(38)	39(2)	46(3)	36(3)	-3(2)	-11(2)	4(2)
C(28)	63(3)	46(3)	42(3)	-7(2)	-10(3)	-10(2)

3.12 Crystal Structure Determination of the gold complexes

Crystal Structure Determination of [(3b-Cy)AuCl]

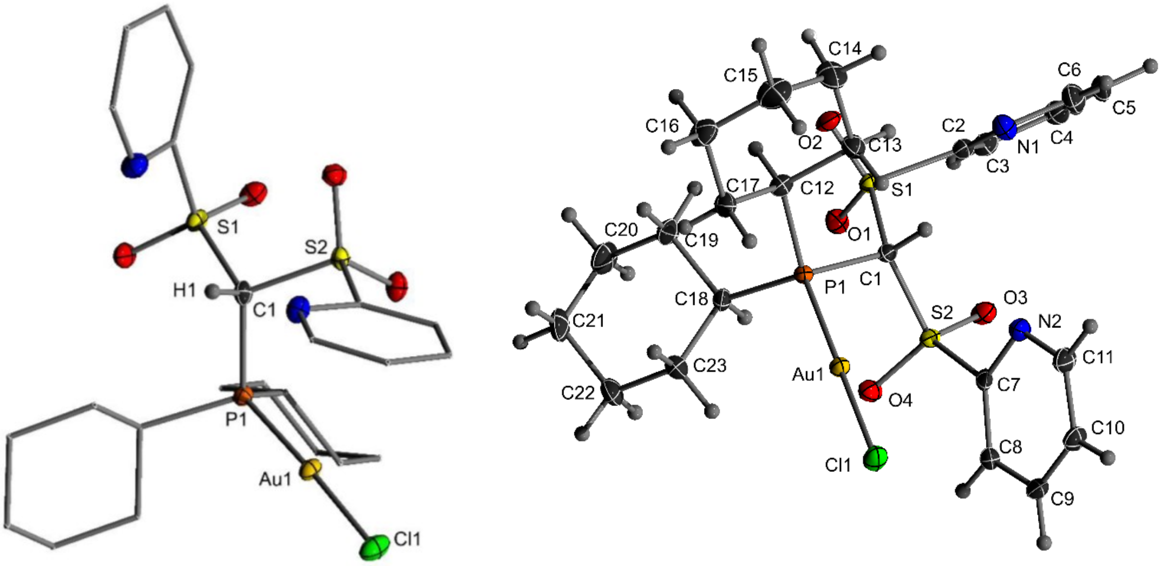

Figure S89. Figure and ORTEP Plot of **[(3b-Cy)AuCl]**. Ellipsoids are drawn at 50 % probability level. Important bond lengths and angles: S(1)–O(1) 1.435(2), S(1)–O(2) 1.436(2), S(1)–C(2) 1.780(3), S(1)– C(1) 1.816(3), P(1)–C(12) 1.836(3), P(1)–C(18) 1.857(3), P(1)–C(1) 1.865(3), P(1)–Au(1) 2.2391(7), Au(1)– Cl(1) 2.2843(7), P(1)–Au(1)–Cl(1) 177.76(3), C(2)–S(1)–C(1) 103.61(13), S(2)–C(1)–P(1) 113.17(15), S(1)– C(1)–P(1) 115.27(15), S(2)–C(1)–S(1) 109.01(16).

Table S38. Atomic coordinates (× 10^4) and equivalent isotropic displacement parameters (Å^2× 10^3) for **[(3b-Cy)AuCl]**. U(eq) is defined as one third of the trace of the orthogonalized U^{ij} tensor.

	x	y	z	U(eq)
S(1)	7671(1)	5591(1)	826(1)	14(1)
P(1)	6698(1)	5568(1)	3110(1)	12(1)
O(1)	7765(2)	4317(2)	286(2)	19(1)
N(1)	9068(3)	8078(3)	1295(2)	20(1)
C(1)	8066(3)	6211(3)	2496(3)	14(1)
S(2)	9479(1)	5955(1)	3016(1)	13(1)
O(2)	6467(2)	5678(2)	462(2)	20(1)
N(2)	10164(2)	8406(2)	4769(2)	18(1)
C(2)	8902(3)	6814(3)	600(3)	14(1)
O(4)	9074(2)	4668(2)	3034(2)	18(1)
C(4)	10435(3)	7408(3)	-440(3)	21(1)
C(5)	10630(3)	8721(3)	239(3)	21(1)
C(6)	9945(3)	9018(3)	1107(3)	23(1)
C(7)	10205(3)	7232(3)	4575(3)	13(1)
C(8)	10826(3)	6945(3)	5420(3)	16(1)
C(9)	11466(3)	7989(3)	6595(3)	18(1)
C(10)	11436(3)	9230(3)	6845(3)	19(1)
C(11)	10773(3)	9395(3)	5915(3)	21(1)
C(12)	5511(3)	6221(3)	2761(3)	16(1)
C(13)	6139(3)	7721(3)	3074(3)	21(1)

C(14)	5104(3)	8234(4)	2834(4)	31(1)
C(15)	4102(4)	7980(4)	3540(4)	33(1)
C(16)	3495(3)	6496(4)	3238(3)	25(1)
C(17)	4526(3)	5992(3)	3501(3)	18(1)
C(18)	5989(3)	3706(3)	2250(3)	16(1)
C(19)	4808(3)	3133(3)	1134(3)	22(1)
C(20)	4319(3)	1600(3)	468(3)	30(1)
C(21)	3982(3)	970(3)	1337(4)	28(1)
C(22)	5131(3)	1535(3)	2448(3)	28(1)
C(23)	5644(3)	3072(3)	3131(3)	22(1)
O(3)	10353(2)	6260(2)	2308(2)	18(1)
C(3)	9547(3)	6404(3)	-259(3)	18(1)
Au(1)	7494(1)	6388(1)	5158(1)	14(1)
Cl(1)	8250(1)	7260(1)	7257(1)	22(1)

Table S39. Anisotropic displacement parameters (Å^{2}x 10^3) for **[(3b-Cy)AuCl]**. The anisotropic displacement factor exponent takes the form: -2p^2[h^2 a$\cdot^2$U^{11} + ... + 2 h k a$\cdot$b$\cdot$U^{12}].

	U^{11}	U^{22}	U^{33}	U^{23}	U^{13}	U^{12}
S(1)	13(1)	16(1)	11(1)	6(1)	1(1)	4(1)
P(1)	11(1)	13(1)	12(1)	7(1)	2(1)	4(1)
O(1)	23(1)	16(1)	15(1)	6(1)	4(1)	5(1)
N(1)	23(1)	17(1)	20(1)	8(1)	7(1)	6(1)
C(1)	12(1)	13(1)	15(1)	6(1)	1(1)	5(1)
S(2)	13(1)	13(1)	12(1)	5(1)	1(1)	6(1)
O(2)	14(1)	29(1)	17(1)	12(1)	2(1)	9(1)
N(2)	18(1)	15(1)	17(1)	7(1)	1(1)	5(1)
C(2)	13(1)	16(1)	14(1)	7(1)	2(1)	5(1)
O(4)	21(1)	14(1)	19(1)	6(1)	1(1)	7(1)
C(4)	18(2)	29(2)	19(2)	13(1)	7(1)	9(1)
C(5)	16(1)	22(2)	21(2)	13(1)	4(1)	0(1)
C(6)	24(2)	17(2)	24(2)	9(1)	6(1)	4(1)
C(7)	9(1)	13(1)	14(1)	6(1)	3(1)	2(1)
C(8)	13(1)	15(1)	19(2)	9(1)	4(1)	4(1)
C(9)	14(1)	22(2)	18(2)	11(1)	1(1)	5(1)
C(10)	18(1)	19(2)	13(2)	4(1)	-1(1)	4(1)
C(11)	20(2)	14(1)	22(2)	5(1)	0(1)	5(1)
C(12)	14(1)	16(1)	14(2)	6(1)	2(1)	6(1)
C(13)	18(2)	18(2)	30(2)	14(1)	6(1)	7(1)
C(14)	27(2)	28(2)	49(2)	23(2)	13(2)	18(2)
C(15)	29(2)	37(2)	39(2)	15(2)	12(2)	22(2)
C(16)	18(2)	36(2)	25(2)	15(2)	8(1)	12(1)
C(17)	14(1)	22(2)	22(2)	12(1)	7(1)	7(1)
C(18)	12(1)	13(1)	18(2)	6(1)	2(1)	3(1)
C(19)	21(2)	16(2)	18(2)	4(1)	-4(1)	2(1)
C(20)	27(2)	18(2)	27(2)	0(1)	-5(1)	4(1)
C(21)	20(2)	12(1)	43(2)	9(1)	5(2)	2(1)

C(22)	23(2)	17(2)	42(2)	18(2)	5(2)	4(1)
C(23)	21(2)	19(2)	26(2)	14(1)	3(1)	4(1)
O(3)	16(1)	23(1)	15(1)	8(1)	5(1)	8(1)
C(3)	18(1)	20(2)	16(2)	7(1)	4(1)	8(1)
Au(1)	13(1)	16(1)	12(1)	7(1)	2(1)	3(1)
Cl(1)	20(1)	25(1)	14(1)	9(1)	1(1)	2(1)

Crystal Structure Determination of [(4b-Cy)Au(PPh3)]

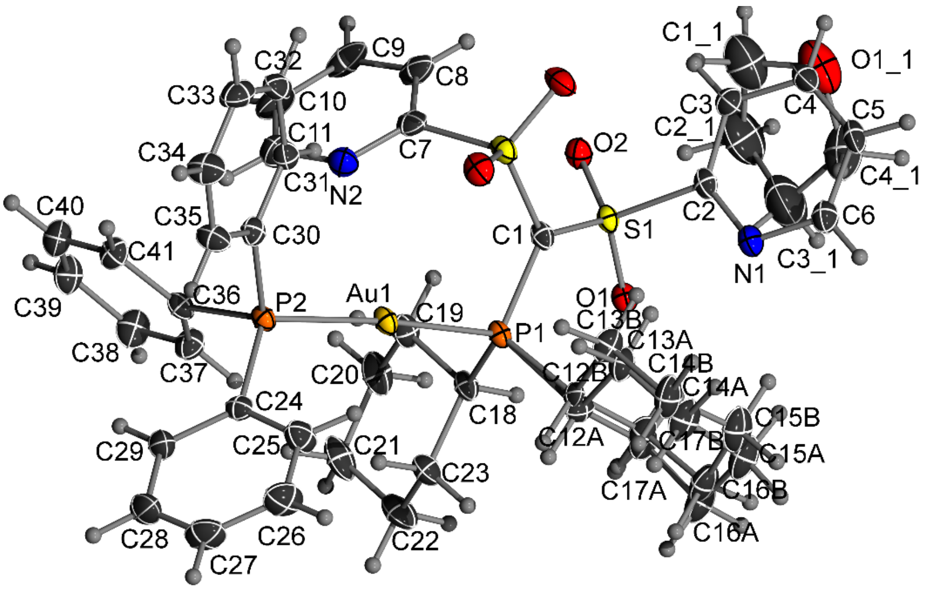

Figure S90. ORTEP plot of **[(4b-Cy)Au(PPh3)]**. Ellipsoids are drawn at 50 % probability level.

Table S40. Atomic coordinates (× 10^4) and equivalent isotropic displacement parameters ($Å^2$× 10^3) for **[(4b-Cy)Au(PPh3)]**. U(eq) is defined as one third of the trace of the orthogonalized U^{ij} tensor.

	x	y	z	U(eq)
N(1)	10037(2)	3870(1)	3053(1)	21(1)
O(1)	9022(1)	2031(1)	2980(1)	19(1)
S(1)	8501(1)	2795(1)	2724(1)	16(1)
Au(1)	5612(1)	3119(1)	3911(1)	18(1)
P(1)	7242(1)	2660(1)	3663(1)	16(1)
C(1)	7543(2)	3203(2)	3094(1)	18(1)
P(2)	3955(1)	3350(1)	4166(1)	16(1)
C(2)	9449(2)	3720(2)	2658(1)	18(1)
N(2)	4939(2)	3075(2)	2831(1)	27(1)
O(2)	8130(1)	2601(1)	2233(1)	22(1)
S(2)	6714(1)	4024(1)	2842(1)	21(1)
C(3)	9538(2)	4193(2)	2216(1)	22(1)

O(3)	7233(1)	4528(1)	2455(1)	30(1)
C(4)	10294(2)	4892(2)	2184(1)	25(1)
O(4)	6233(1)	4580(1)	3222(1)	25(1)
C(5)	10919(2)	5067(2)	2588(1)	24(1)
C(6)	10772(2)	4538(2)	3010(1)	25(1)
C(7)	5710(2)	3343(2)	2546(1)	23(1)
C(8)	5816(2)	3074(2)	2062(1)	31(1)
C(9)	5095(2)	2445(2)	1867(1)	36(1)
C(10)	4311(2)	2117(2)	2163(1)	36(1)
C(11)	4245(2)	2472(2)	2637(1)	35(1)
C(18)	7261(2)	1352(2)	3603(1)	19(1)
C(19)	6533(2)	1025(2)	3186(1)	24(1)
C(20)	6537(2)	-63(2)	3137(1)	33(1)
C(21)	6276(2)	-540(2)	3623(1)	35(1)
C(22)	6990(2)	-213(2)	4032(1)	34(1)
C(23)	6972(2)	873(2)	4088(1)	25(1)
C(24)	3737(2)	3298(2)	4822(1)	19(1)
C(25)	4384(2)	3830(2)	5127(1)	29(1)
C(26)	4193(2)	3903(2)	5623(1)	36(1)
C(27)	3365(2)	3421(2)	5819(1)	36(1)
C(28)	2744(2)	2859(2)	5525(1)	34(1)
C(29)	2916(2)	2805(2)	5023(1)	25(1)
C(30)	3396(2)	4485(2)	3977(1)	19(1)
C(31)	3642(2)	4835(2)	3514(1)	21(1)
C(32)	3213(2)	5677(2)	3348(1)	25(1)
C(33)	2550(2)	6189(2)	3642(1)	27(1)
C(34)	2311(2)	5853(2)	4103(1)	31(1)
C(35)	2730(2)	4998(2)	4273(1)	25(1)
C(36)	3157(2)	2441(2)	3873(1)	19(1)
C(38)	3054(2)	862(2)	3534(1)	30(1)
C(37)	3584(2)	1544(2)	3804(1)	25(1)
C(39)	2097(2)	1076(2)	3326(1)	28(1)
C(40)	1655(2)	1958(2)	3405(1)	27(1)
C(41)	2183(2)	2645(2)	3677(1)	23(1)
C11	10284(3)	2368(3)	1241(2)	64(1)
O11	11047(2)	3018(2)	1400(1)	58(1)
C21	10607(4)	1427(3)	1431(2)	69(1)
C31	11154(4)	1627(4)	1906(2)	78(1)
C41	11544(3)	2642(3)	1825(2)	56(1)
C(12A)	8270(16)	2900(13)	4170(8)	17(3)

C(13A)	8434(14)	3998(12)	4177(7)	24(3)
C(14A)	9198(15)	4287(12)	4581(6)	33(3)
C(15A)	10185(12)	3717(15)	4547(5)	36(3)
C(16A)	9994(11)	2645(14)	4557(6)	32(3)
C(17A)	9287(19)	2382(15)	4123(10)	22(3)
C(12B)	8121(15)	3062(15)	4115(8)	14(3)
C(13B)	8198(16)	4141(14)	4119(8)	25(3)
C(14B)	8908(17)	4498(15)	4524(6)	35(3)
C(15B)	9967(15)	4042(19)	4484(6)	39(4)
C(16B)	9900(12)	2963(19)	4510(6)	31(3)
C(17B)	9180(20)	2593(17)	4098(10)	21(3)

Table S41. Anisotropic displacement parameters (Å^{2}x 10^3) for **[(4b-Cy)Au(PPh$_3$)]**. The anisotropic displacement factor exponent takes the form: -2p^2[h^2 a^{*2}U^{11} + ... + 2 h k a*b*U^{12}].

	U^{11}	U^{22}	U^{33}	U^{23}	U^{13}	U^{12}
N(1)	20(1)	19(1)	25(1)	0(1)	3(1)	-2(1)
O(1)	18(1)	15(1)	24(1)	-2(1)	4(1)	0(1)
S(1)	14(1)	15(1)	18(1)	-1(1)	3(1)	0(1)
Au(1)	16(1)	17(1)	20(1)	0(1)	6(1)	0(1)
P(1)	15(1)	15(1)	18(1)	-1(1)	4(1)	-1(1)
C(1)	15(1)	16(1)	22(1)	1(1)	5(1)	1(1)
P(2)	16(1)	16(1)	16(1)	0(1)	4(1)	1(1)
C(2)	15(1)	15(1)	24(1)	-2(1)	6(1)	2(1)
N(2)	25(1)	38(1)	19(1)	1(1)	2(1)	-3(1)
O(2)	20(1)	25(1)	21(1)	-4(1)	2(1)	-2(1)
S(2)	18(1)	19(1)	25(1)	5(1)	7(1)	4(1)
C(3)	19(1)	22(1)	24(1)	1(1)	1(1)	3(1)
O(3)	26(1)	27(1)	36(1)	14(1)	12(1)	6(1)
C(4)	23(1)	22(1)	31(1)	9(1)	8(1)	1(1)
O(4)	23(1)	20(1)	34(1)	1(1)	8(1)	6(1)
C(5)	16(1)	16(1)	41(1)	4(1)	7(1)	-1(1)
C(6)	20(1)	22(1)	32(1)	0(1)	1(1)	-2(1)
C(7)	20(1)	26(1)	23(1)	6(1)	0(1)	8(1)
C(8)	21(1)	51(2)	20(1)	2(1)	1(1)	13(1)
C(9)	29(1)	56(2)	25(1)	-10(1)	-5(1)	19(1)
C(10)	35(2)	40(2)	32(2)	-3(1)	-13(1)	4(1)
C(11)	29(1)	48(2)	27(1)	3(1)	-3(1)	-9(1)
C(18)	19(1)	14(1)	25(1)	0(1)	4(1)	-2(1)

C(19)	24(1)	23(1)	24(1)	-5(1)	2(1)	-5(1)
C(20)	34(2)	25(1)	38(2)	-11(1)	9(1)	-10(1)
C(21)	37(2)	20(1)	50(2)	-4(1)	15(1)	-10(1)
C(22)	41(2)	21(1)	42(2)	8(1)	10(1)	-2(1)
C(23)	28(1)	21(1)	27(1)	3(1)	4(1)	-3(1)
C(24)	20(1)	19(1)	18(1)	2(1)	2(1)	4(1)
C(25)	34(1)	28(1)	26(1)	-1(1)	1(1)	-9(1)
C(26)	47(2)	38(2)	23(1)	-5(1)	-5(1)	-6(1)
C(27)	41(2)	51(2)	17(1)	0(1)	3(1)	6(1)
C(28)	26(1)	52(2)	25(1)	8(1)	6(1)	1(1)
C(29)	19(1)	32(1)	24(1)	4(1)	1(1)	-1(1)
C(30)	18(1)	17(1)	22(1)	-2(1)	-1(1)	-3(1)
C(31)	20(1)	23(1)	21(1)	-1(1)	1(1)	-3(1)
C(32)	29(1)	24(1)	21(1)	4(1)	-4(1)	-9(1)
C(33)	31(1)	21(1)	30(1)	4(1)	-7(1)	2(1)
C(34)	35(2)	27(1)	31(1)	1(1)	5(1)	10(1)
C(35)	31(1)	23(1)	23(1)	3(1)	6(1)	4(1)
C(36)	21(1)	19(1)	18(1)	0(1)	5(1)	-1(1)
C(38)	30(1)	19(1)	42(2)	-7(1)	-1(1)	2(1)
C(37)	22(1)	22(1)	30(1)	-1(1)	-2(1)	2(1)
C(39)	26(1)	26(1)	34(1)	-9(1)	0(1)	-5(1)
C(40)	21(1)	29(1)	32(1)	-5(1)	-1(1)	-1(1)
C(41)	20(1)	24(1)	25(1)	-3(1)	3(1)	5(1)
C11	65(3)	61(2)	65(3)	-10(2)	20(2)	-14(2)
O11	57(2)	49(1)	69(2)	-6(1)	24(1)	-9(1)
C21	76(3)	46(2)	86(3)	-2(2)	13(2)	-26(2)
C31	67(3)	75(3)	92(4)	14(3)	18(3)	-22(2)
C41	35(2)	40(2)	91(3)	-16(2)	11(2)	9(2)
C(12A)	21(5)	13(5)	17(4)	2(4)	-10(4)	2(4)
C(13A)	19(6)	21(5)	31(5)	-3(4)	-1(4)	1(4)
C(14A)	29(6)	37(5)	34(4)	-20(4)	-3(4)	-2(4)
C(15A)	22(5)	43(7)	44(5)	-18(4)	-7(3)	-1(4)
C(16A)	21(4)	40(6)	33(5)	-3(4)	-12(3)	5(4)
C(17A)	16(5)	22(6)	30(4)	1(5)	-4(3)	5(4)
C(12B)	8(5)	21(6)	14(5)	8(4)	-1(3)	6(3)
C(13B)	25(7)	22(5)	27(5)	-10(4)	-1(4)	2(4)
C(14B)	26(7)	43(6)	36(5)	-17(5)	3(4)	-8(5)
C(15B)	27(6)	53(8)	35(5)	-27(5)	3(4)	-12(5)
C(16B)	18(4)	57(9)	19(4)	1(5)	-7(3)	6(5)
C(17B)	16(5)	26(8)	21(4)	1(6)	-5(3)	7(5)

4. Calculations

4.1. Computational Details

All calculations were performed with the Gaussian16 Revision C.01 program package[9] using Density Functional Theory (DFT)[10] without any symmetry restrictions. Geometry optimizations and single point calculations were carried with the PW6B95-D3 functional[11] and the def2svp (optimization) or def2tzvp (single point) basis set[12]. Solvent corrections were included using the polarizable continuum model (PCM) for THF implemented in the Gaussian package. Harmonic vibrational frequency calculations were performed on the same level of theory. No imaginary frequencies were obtained. NBO analyses and calculation of Wiberg Bond Indices (WBIs) were performed on the same level of theory like single point calculations using the NBO7 program package[13].
For determination of the Tolman Electronic Parameter (TEP) via the minimum of the electrostatic potential at the phosphorus atom (MESP) a geometry optimization with B3LYP[14]/6-31+g*[15] and Grimme's D3[16] dispersion correction was performed followed by a CHelp population analysis[17] on the same level of theory. The electric potential at the phosphorus atom (V_P in a.u.) from the CHelp fit was used to determine the TEP value (cm^{-1}) from our empirical linear correlation:[18]

$$\mathrm{TEP}(\mathrm{cm}^{-1}) = 0.542541 \times (627.5\ \mathrm{V_P}) + 20492.536026$$

For each compound the conformational space was analyzed with the program package CREST[19] by Grimme. Implicit solvation for THF was included by the generalized born model implemented in CREST. The first 10 structures with the lowest energies were used as starting geometries for geometry optimizations as described in the beginning. In addition, the molecular structures from crystals were optimized. In cases where no crystal structure was obtained the crystal structure of a similar compound was used and the substituents at the phosphorus atom (PR_2) were exchanged.
The structure with the lowest Gibbs Free Energy was used to determine energy differences, as well as to perform NBO analysis and calculate TEP values. In case of the ylidic compounds **3a'** and **3b'** the CREST conformer search did not deliver the conformers with the lowest energies as the structure obtained from the crystals were the lowest in energy. For the other compounds the structure with the lowest energy was obtained from the CREST conformer search. The energetically most favored conformer of each compound is provided as cartesian coordinates below. For the visualization of the Connolly surfaces the program Jmol was used.[20]

4.2. Results of DFT calculations

Table S42. SCF energies, thermal corrections to Gibbs free energy and enthalpy in atomic units of ylides **3'** and neutral phosphine ligands **3**. Relative energies in kJ/mol using the ylidic compound (**3a'** or **3b'**) as reference, respectively.

Compound	ΔG corr (a.u.)	ΔH corr (a.u.)	SCF energy (a.u.)	ΔG (kJ/mol)	ΔH (kJ/mol)
**3a'-Cy**	0.532848	0.635898	-2493.037382	0.00	0.00
**3a-Cy**	0.533782	0.636922	-2493.034475	10.08	10.32
**3a'-iPr**	0.406284	0.49938	-2259.163295	0.00	0.00
**3a-iPr**	0.40741	0.498855	-2259.160898	9.25	4.91
**3a'-Ph**	0.396825	0.492914	-2485.781175	0.00	0.00
**3a-Ph**	0.399347	0.493805	-2485.78356	0.36	-3.92
**3b'-Cy**	0.458139	0.55356	-2446.388973	0.00	0.00
**3b-Cy**	0.461641	0.554967	-2446.383781	22.83	17.33
**3b'-iPr**	0.332472	0.416718	-2212.515906	0.00	0.00
**3b-iPr**	0.332387	0.417514	-2212.510943	12.81	15.12
**3b'-Ph**	0.32198	0.411587	-2439.129194	0.00	0.00
**3b-Ph**	0.322489	0.412777	-2439.130889	-3.11	-1.33

Table S43. SCF energies, thermal corrections to Gibbs free energy and enthalpy (in a.u.) of anions **4a** and **4b**.

Compound	ΔG corr (a.u.)	ΔH corr (a.u.)	SCF energy (a.u.)
**4a-Cy**	0.522484	0.623931	-2492.55843
**4a-iPr**	0.394095	0.487029	-2258.68266
**4a-Ph**	0.385538	0.482263	-2485.31221
**4b-Cy**	0.45066	0.541863	-2445.90738
**4b-iPr**	0.321235	0.404606	-2212.03314
**4b-Ph**	0.312783	0.400111	-2438.66006

Table S44. SCF energies, thermal corrections to Gibbs free energy and enthalpy (in a.u.) of anions **5a** and **5b**.

Compound	ΔG corr (a.u.)	ΔH corr (a.u.)	SCF energy (a.u.)
**5a-Cy**	0.54609	0.648309	-2493.47
**5a-iPr**	0.417134	0.51174	-2259.59
**5a-Ph**	0.408894	0.506325	-2486.21
**5b-Cy**	0.471131	0.565949	-2446.82
**5b-iPr**	0.341729	0.428564	-2212.94
**5b-Ph**	0.333934	0.423859	-2439.55

Table S45. Results of NBO analyses and calculation of Wiberg bond indices.

Compound	Wiberg Bond Index			Natural charge	
	P–C	C–S1	C–S2	q_P	q_C
**3a'-Cy**	1.017	0.899	0.918	1.413	-1.346
**3a-Cy**	0.862	0.778	0.777	0.856	-1.062
**3a'-Ph**	1.053	0.881	0.889	1.384	-1.342
**3a-Ph**	0.859	0.763	0.788	0.872	-1.041
**3a'-PiPr**	1.043	0.907	0.883	1.401	-1.347
**3a-iPr**	0.858	0.771	0.771	0.845	-1.054
**3b'-Cy**	1.022	0.920	0.906	1.424	-1.341
**3b-Cy**	0.865	0.789	0.779	0.856	-1.093
**3b'-Ph**	1.036	0.902	0.897	1.398	-1.337
**3b-Ph**	0.866	0.786	0.770	0.867	-1.088
**3b'-iPr**	1.030	0.912	0.903	1.395	-1.346
**3b-iPr**	0.867	0.789	0.779	0.826	-1.092
**4a-Cy**	0.958	0.943	0.948	0.866	-1.310
**4a-Ph**	0.973	0.937	0.925	0.896	-1.309
**4a-iPr**	0.966	0.945	0.938	0.843	-1.312
**4b-Cy**	0.960	0.946	0.956	0.878	-1.294
**4b-Ph**	0.975	0.928	0.948	0.906	-1.291
**4b-iPr**	0.962	0.948	0.953	0.864	-1.297

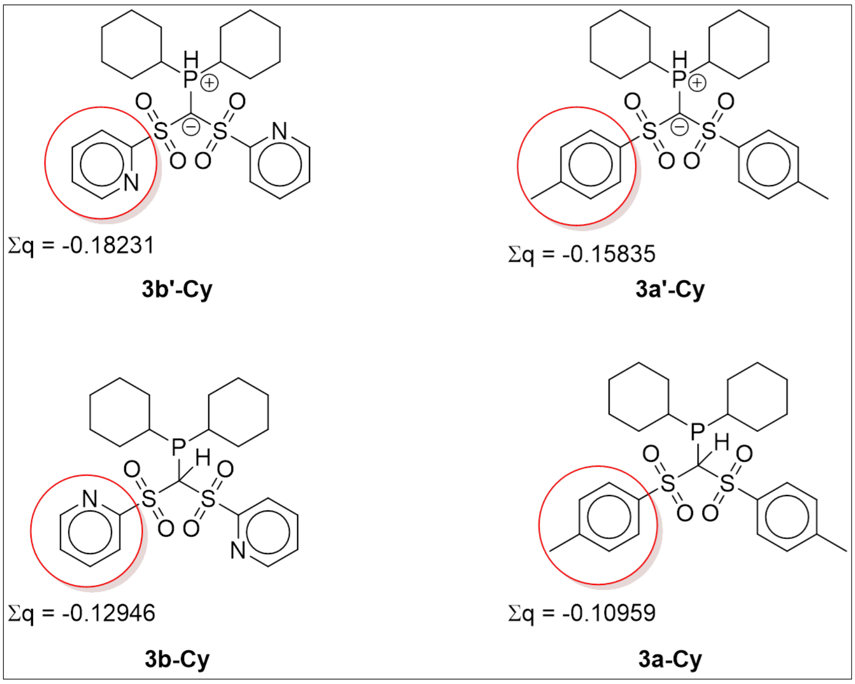

Figure S91. Sum of Natural charges in the tolyl ring of **3a**(‘)**-Cy** and the pyridyl ring of **3b**(‘)**-Cy**.

Comparison of the NBO charges in the phosphines **3b-Cy** and **3a-Cy** with the corresponding PH ylides **3b'-Cy** and **3a'-Cy** showed that more negative charge from the ylidic carbon center is shifted into the pyridly than in the tolyl ring. Accordingly, the sum of NBO charges in the pyridyl group (C_5H_4N) increases by Δq = -0.053e$^-$, whereas it increases only by Δq = -0.049e$^-$ in the tolyl group (C_7H_7). This propensity of the pyridyl moiety to accept more electron density explains the higher preference of the ylidic structure of **3b'-Cy** in comparison to **3a'-Cy**.

4.2 Calculation of Tolman Electronic Parameter (TEP)

1) Determination of the TEP value via the minimum of the electrostatic potential at phosphorus

A geometry optimization with B3LYP[21]/6-31+g*[22] and Grimme's D3[23] dispersion correction was performed followed by a CHelp population analysis[24] on the same level of theory. The electrostatic potential at the phosphorus atom (V_P in a.u.) from the CHelp fit was used to determine the TEP value (cm^{-1}) from our empirical linear correlation:[25]

$$\mathrm{TEP(cm^{-1})} = 0.542541 \times (627.5\ \mathrm{V_P}) + 20492.536026$$

Table S46. Calculated V_P (a.u.) values from Chelp population analysis and determined TEP (cm^{-1}) values from our fit.

Compound	V_P (a.u.) from CHELP	Calculated TEP (cm^{-1}) from fit
**3a-Cy**	-54.148905	2057.84
**3a-iPr**	-54.146631	2058.61
**3a-Ph**	-54.131452	2063.78
**3b-Cy**	-54.156411	2055.28
**4a-Cy**	-54.281945	2012.55
**4a-iPr**	-54.284965	2011.52
**4a-Ph**	-54.260116	2019.98
**4b-Cy**	-54.287898	2010.52

2) Determination of the TEP from calculated CO frequencies in $LNi(CO)_3$ complexes

The $LNi(CO)_3$ complexes were optimized with PBE0/def2-svp and Grimme's D3 dispersion correction followed by a single point calculation with PBE0[26]/def2-tzvp.[12] Harmonic vibrational frequency calculations were performed on the same level of theory.

Calculated A_1 C-O frequencies $\tilde{\nu}_{CO}$ of different phosphines were fitted against their experimental TEP values. TEP values for compounds **3a-R**, **3b-R**, **4a-R** and **4b-R** were calculated accordingly from the fit.

Table S47. Calculated A_1 C-O frequencies $\tilde{\nu}_{CO}$ and known experimental TEP values[27] in cm^{-1} which were used for the fit.

PR_3	Calculated ν_{CO} in cm^{-1}	TEP experimental in cm^{-1}
**$PtBu_3$**	2176.65	2056.1
**$PNMe_2$**	2187.23	2061.9
**PMe_3**	2185.61	2064.1
**$P(C_6H_5OMe)_3$**	2188.70	2066.1
**$P(o\text{-}Tol)_3$**	2189.56	2066.6
**$P(p\text{-}Tol)_3$**	2190.32	2066.7
**PPh_3**	2192.47	2068.9
**3a-Cy**	2186.45	2069

**3b-Cy**	2187.58	2067
**4a-Cy**	2171.41	2044.8
**4b-Cy**	2172.37	2046.1

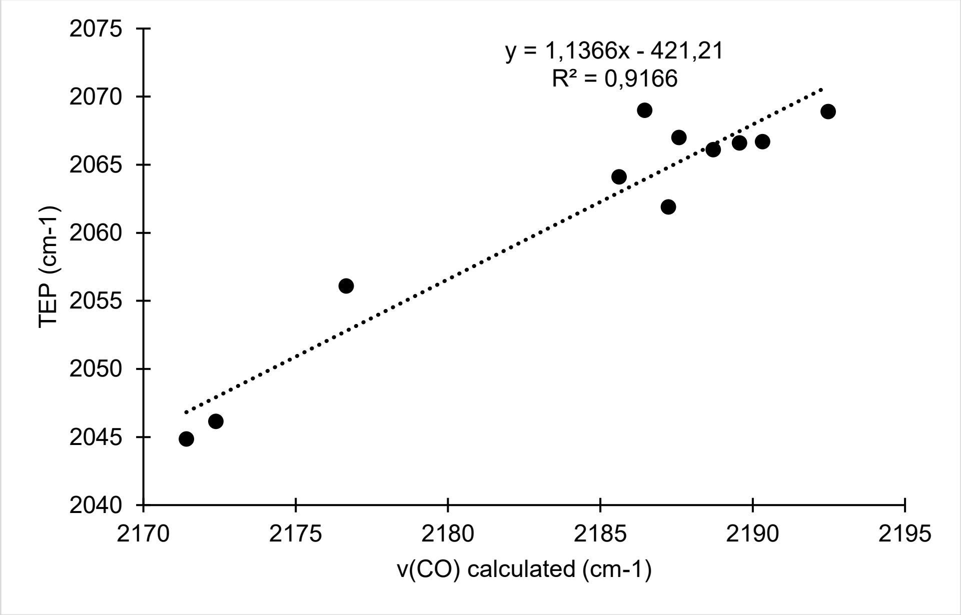

Figure S92. Linear regression of the TEP against the calculated A_1 C-O frequency ν_{CO} in cm^{-1}.

Table S48. Calculated A_1 C-O frequencies $\tilde{\nu}_{CO}$ and calculated TEP values in cm^{-1} from the linear regression.

PR_3	Calculated ν_{CO} in cm^{-1}	TEP_{calc} in cm^{-1}
**3a-Ph**	2194.92	2073.54
**3a-Cy**	2186.45	2063.91
**3a-iPr**	2190.70	2068.74
**3b-Cy**	2187.58	2065.19
**4a-Ph**	2173.45	2049.13
**4a-Cy**	2171.41	2046.81
**4a-iPr**	2171.30	2046.69
**4b-Cy**	2172.37	2047.91

4.3 Proton affinities

Proton affinities PA and gas phase basicities GB were obtained by

$$\mathrm{PA} = \Delta H_g^0(\mathrm{HA}) - \Delta H_g^0(\mathrm{A}^-) - \Delta H_g^0(\mathrm{H}^+)$$

$$\mathrm{GB} = \Delta G_g^0(\mathrm{HA}) - \Delta G_g^0(\mathrm{A}^-) - \Delta G_g^0(\mathrm{H}^+)$$

with $\Delta H_g^0(\mathrm{H}^+) = 2.5\,\mathrm{RT} = 1.48\,\mathrm{kcal/mol}$ and $\Delta G_g^0(\mathrm{H}^+) = 2.5\,\mathrm{RT} - \mathrm{T}\Delta S^0 = 1.48 - 7.76 = -6.28\,\mathrm{kcal/mol}$ at 298 K.[28]

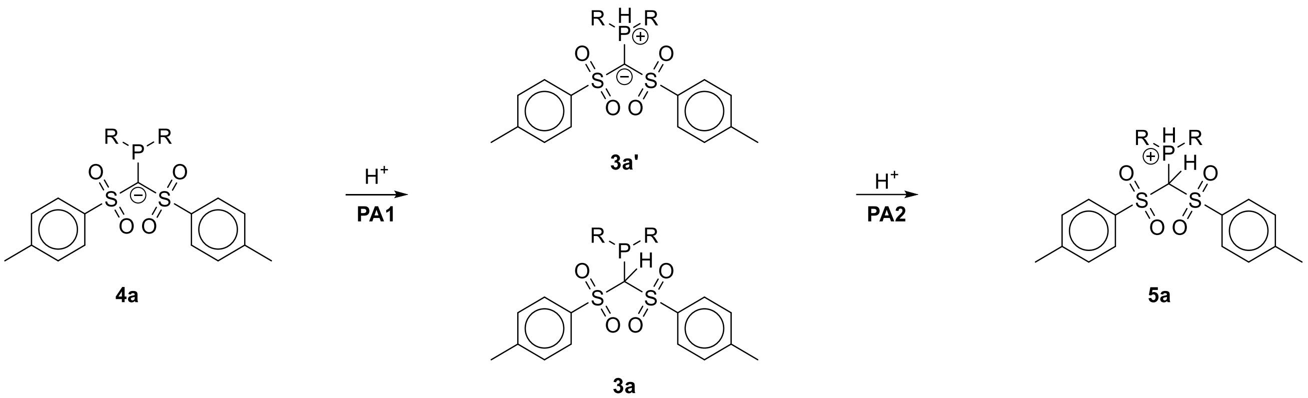

Figure S93. Proton affinities PA1 (**4a** + H⁺ → **3a** or **4a** + H⁺ → **3a'**) and PA2 (**3a** + H⁺ → **5a** or **3a'** + H⁺ → **5a**).

Table S49. Proton affinities PA1/ΔH in kcal/mol (**4a** + H⁺ → **3a** or **4a** + H⁺ → **3a'**) and PA2/ ΔH(**3a** + H⁺ → **5a** or **3a'** + H⁺ → **5a**). @P indicates protonation at the phosphorus center, @C protonation at the carbon atom.

Compound	PA1/ ΔH(**4a + H+** → **3a** or **4a + H+** → **3a'**)	PA2/ ΔH(**3a + H⁺** → **5a** or **3a' + H⁺** → **5a**)
**3a'-Cy**	-294.51 (@P)	-265.16 (@C)
**3a-Cy**	-292.05 (@C)	-267.13 (@P)
**3a'-iPr**	-295.34 (@P)	-261.48 (@C)
**3a-iPr**	-294.15 (@C)	-262.66 (@P)
**3a'-Ph**	-289.07 (@P)	-262.15 (@C)
**3a-Ph**	-290.01 (@C)	-261.21 (@P)
**3b'-Cy**	-296.34 (@P)	-264.18 (@C)
**3b-Cy**	-292.20 (@C)	-268.32 (@P)
**3b'-iPr**	-296.82 (@P)	-260.17 (@C)
**3b-iPr**	-293.20 (@C)	-263.78 (@P)
**3b'-Ph**	-288.66 (@P)	-257.84 (@C)
**3b-Ph**	-288.98 (@C)	-257.52 (@P)

Table S50. Gas phase basicity GB1/ΔG in kcal/mol (**4a** + H^+ → **3a** or **4a** + H^+ → **3a'**) and GB2/ ΔG (**3a** + H^+ → **5a** or **3a'** + H^+ → **5a**). @P indicates protonation at the phosphorus center, @C protonation at the carbon atom.

Compound	GB1/ ΔG(**4a + H+** → **3a** or **4a + H+** → **3a'**)	GB2/ ΔG(**3a + H^+** → **5a** or **3a' + H^+** → **5a**)
**3a'-Cy**	-300.32 (@P)	-269.44 (@C)
**3a-Cy**	-297.91 (@C)	-271.85 (@P)
**3a'-iPr**	-300.23 (@P)	-267.23 (@C)
**3a-iPr**	-298.02 (@C)	-269.44 (@P)
**3a'-Ph**	-293.47 (@P)	-267.79 (@C)
**3a-Ph**	-293.39 (@C)	-267.88 (@P)
**3b'-Cy**	-303.79 (@P)	-268.60 (@C)
**3b-Cy**	-298.33 (@C)	-274.05 (@P)
**3b'-iPr**	-302.16 (@P)	-266.59 (@C)
**3b-iPr**	-299.10 (@C)	-269.65 (@P)
**3b'-Ph**	-294.89 (@P)	-262.83 (@C)
**3b-Ph**	-295.63 (@C)	-262.09 (@P)

4.4. Connolly surfaces mapped with electrostatic potential

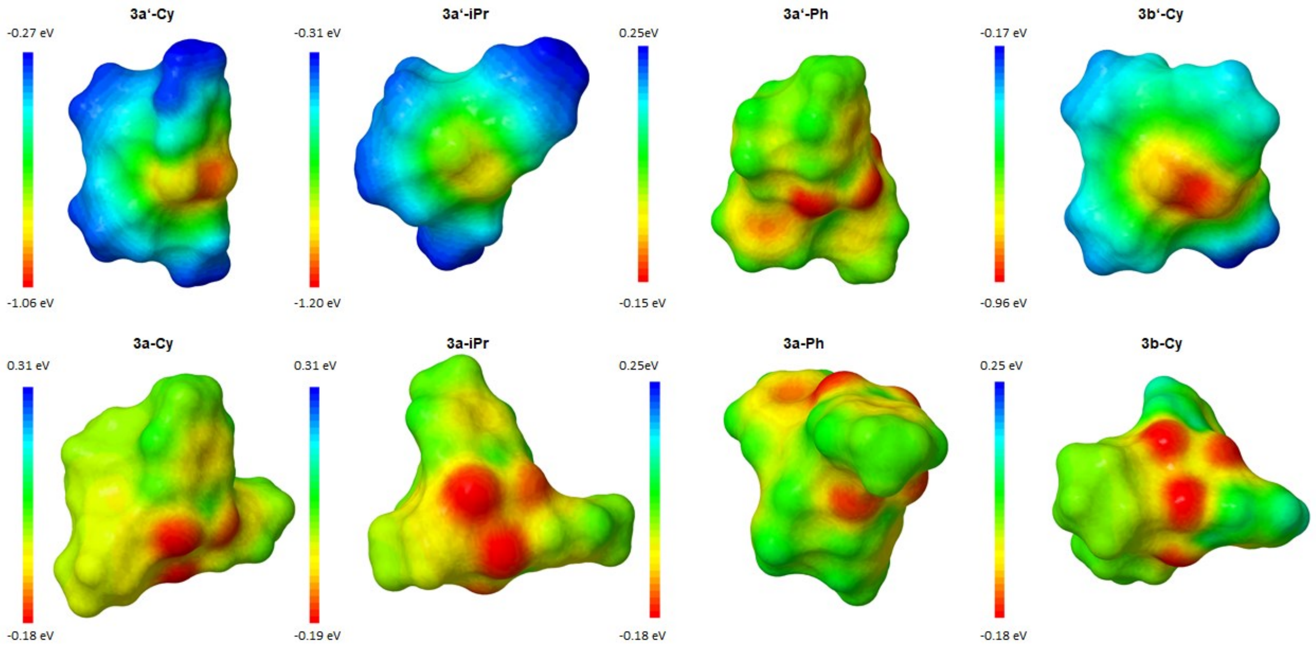

Figure S94. Connolly surfaces (ball size = 2.3 Å) mapped with the electrostatic potential (V_{min} (red), V_{max} (blue) in eV).

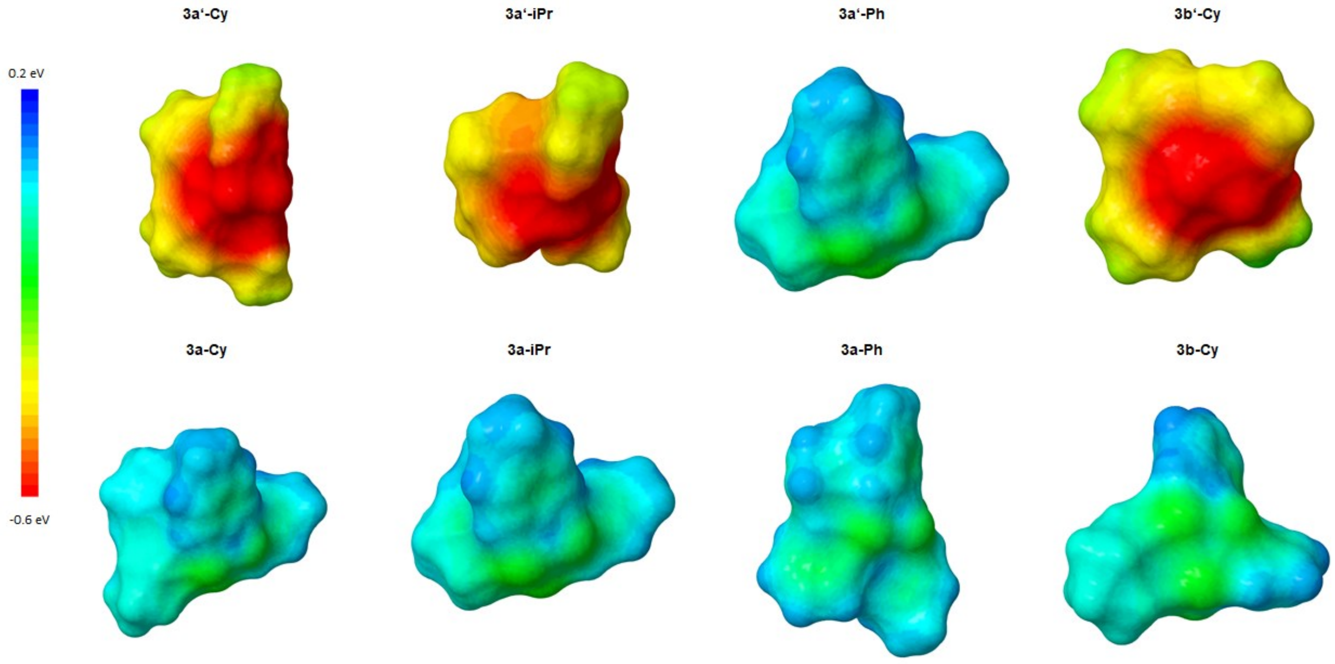

Figure S95. Connolly surfaces (ball size = 2.3 Å) mapped with the electrostatic potential (V =-0.6 eV (red), V = 0.2 eV (blue)).

4.5. Cartesian Coordinates

3b'-Cy

E=-2446.38897348

S 0.345897 -1.000011 1.396257
O 0.66202 0.216135 2.171931
O -0.626318 -1.952588 1.939256
C -0.099399 -0.426878 -0.14698
C 1.889377 -1.910501 1.214253
S -0.559682 -1.528666 -1.364004
P -0.016217 1.282158 -0.430655
N 2.892237 -1.217537 0.711846
C 1.949052 -3.237397 1.610079
O 0.092826 -2.81673 -1.105271
O -0.39584 -0.873152 -2.666021
C -2.324086 -1.813481 -1.157172
H -0.559362 1.434277 -1.71632
C -1.033787 2.307729 0.689004
C 1.721628 1.816889 -0.484033
C 4.056421 -1.841689 0.572745
H 1.065679 -3.729682 2.004817
C 3.17216 -3.882028 1.46243
N -3.087161 -0.763032 -1.400459
C -2.778999 -3.059182 -0.753068
H -0.362971 2.561563 1.522774
C -2.225074 1.520536 1.236513
C -1.499332 3.583452 -0.020416
H 2.122131 1.34394 0.426444
C 2.401307 1.169824 -1.691737
C 1.971922 3.320679 -0.428315
C 4.244453 -3.173854 0.934466
H 4.878598 -1.261187 0.153051
H 3.283441 -4.924836 1.751866
C -4.398842 -0.916657 -1.256459
H -2.073821 -3.862949 -0.564982
C -4.153939 -3.207838 -0.602991
H -1.873437 0.626304 1.760962
H -2.836793 1.170122 0.39336
C -3.061612 2.390939 2.163837
C -2.334759 4.445295 0.917836
H -0.648687 4.157751 -0.404645
H -2.107972 3.299915 -0.892145
H 2.206167 0.091116 -1.69635
H 1.966213 1.579397 -2.616061
C 3.895806 1.452436 -1.658507
C 3.471993 3.595944 -0.415202
H 1.520057 3.805452 -1.306769
H 1.50319 3.759438 0.461323
H 5.21604 -3.642379 0.798813
C -4.978961 -2.120792 -0.861778
H -5.020882 -0.043624 -1.458049
H -4.57341 -4.160678 -0.286905
H -3.924057 1.817006 2.525355
H -2.462644 2.647464 3.050505
C -3.518912 3.669601 1.47629
H -1.7 4.792295 1.7468
H -2.67475 5.342988 0.386408
H 4.323001 0.965727 -0.769514
H 4.380894 1.00109 -2.533048
C 4.175134 2.948154 -1.6003
H 3.648557 4.678815 -0.405545
H 3.895428 3.19964 0.519595
H -6.058331 -2.19606 -0.756006
H -4.201697 3.412606 0.652097
H -4.090796 4.295975 2.172556
H 5.255284 3.134656 -1.547253
H 3.822392 3.417883 -2.531196

3a'-Cy

E=-2493.0373818

P 0.132917 1.115697 0.491043
C 0.048473 -0.591207 0.178571
S 0.703102 -1.712124 1.304069
S -0.641075 -1.034228 -1.326139
H 0.645746 1.205289 1.79566
C -1.552626 1.806888 0.520515
C 1.279646 2.063799 -0.572594
C -2.342446 1.14369 1.650936
C -1.655076 3.327429 0.578378
C 1.867684 3.264371 0.174982
C 2.388617 1.157976 -1.11142
O 0.563219 -1.140988 2.651002
O 0.151033 -3.041384 1.026388
C 2.446423 -1.762982 0.958398
O -0.570825 0.191085 -2.153298
O -0.059625 -2.271095 -1.847958
C -2.381751 -1.315361 -1.058111
C -3.804558 1.559662 1.578935
C -3.122228 3.74399 0.527584
C 2.817498 4.042544 -0.728047
C 3.333432 1.944225 -2.008151
C 3.34172 -1.210546 1.861356
C 2.86908 -2.256291 -0.273865
C -3.30055 -0.65499 -1.862347
C -2.797108 -2.191249 -0.058213
C -3.944261 3.075617 1.62182
C 3.917425 3.148452 -1.282749
C 4.686997 -1.130871 1.51192
C 4.211385 -2.168871 -0.60331
C -4.659098 -0.872223 -1.654927
C -4.154842 -2.387547 0.138926
C 5.141068 -1.597223 0.277486
C -5.108348 -1.733073 -0.652553
C 6.585299 -1.494767 -0.107906
H 7.021703 -2.489547 -0.263728
H 6.702523 -0.943883 -1.049454
H 7.171198 -0.983943 0.662385
C -6.57121 -1.94677 -0.410538
H -6.869858 -1.533293 0.561642
H -7.180252 -1.466355 -1.182471
H -6.817133 -3.015275 -0.392321
H 4.549552 -2.540378 -1.569623
H 2.137249 -2.659102 -0.972216
H 2.979395 -0.83494 2.815003
H 5.396427 -0.691522 2.211524
H -5.384206 -0.356652 -2.282674
H -2.948426 0.02497 -2.634182
H -2.056561 -2.691647 0.563666
H -4.486076 -3.063547 0.925879
H -1.915483 1.457339 2.615497
H -2.238101 0.053434 1.598698
H -4.238103 1.169704 0.64586
H -4.362886 1.096262 2.401838
H -3.602935 3.439451 2.602898
H -4.998535 3.366148 1.531731
H -3.197969 4.835954 0.601028
H -3.531784 3.466402 -0.455088
H -1.109307 3.785487 -0.255688
H -1.19653 3.693955 1.509377
H 1.078292 3.924291 0.553085
H 2.41988 2.898159 1.053645
H 3.246616 4.884358 -0.170406
H 2.243992 4.476953 -1.560331
H 4.571122 3.720834 -1.952873
H 4.549101 2.797258 -0.452477
H 2.784726 2.283097 -2.899561
H 4.133048 1.282529 -2.364713
H 2.956022 0.732938 -0.269691
H 1.945452 0.315691 -1.652703
H 0.664241 2.407181 -1.417095
H -1.962621 1.46959 -0.444157

3b-Cy

E=-2446.38378098

S -1.22026 0.51996 -1.649461
O -0.257172 0.551348 -2.748572
C -0.422413 -0.043984 -0.119759
C -1.594711 2.220596 -1.201332
O -2.473133 -0.213632 -1.812107
P 1.271764 0.72662 -0.020327
H -1.060611 0.375095 0.671637
S -0.596221 -1.833606 0.04976
C -1.227364 3.24304 -2.060105
O -0.309933 -2.475869 -1.232046
O 0.152759 -2.227072 1.243279
C -2.334949 -2.039094 0.440835
C -2.575579 3.595862 0.2986
C -1.591056 4.530947 -1.678977
H -0.67747 3.029223 -2.972139
C -3.095519 -2.954631 -0.265344
H -3.101954 3.706236 1.24659
C -2.273633 4.710265 -0.482591
H -1.335857 5.382203 -2.306115
C -4.023556 -1.412022 1.801419
C -4.420705 -3.089701 0.132882
H -2.663984 -3.514304 -1.090294
H -4.366353 -0.778084 2.618965
C -4.892421 -2.305614 1.179244
H -5.078598 -3.791585 -0.374763
C 2.276527 -0.397846 -1.126637
C 3.267364 0.446592 -1.930879
C 2.97451 -1.582198 -0.465228
H 1.532689 -0.788967 -1.833567
C 4.004895 -0.402679 -2.957617
H 3.994705 0.910642 -1.245069
H 2.732954 1.267982 -2.424961
C 3.700634 -2.426861 -1.505393
H 3.703277 -1.214861 0.273644
H 2.252241 -2.195606 0.081796
C 4.69127 -1.596076 -2.308357
H 4.731932 0.212475 -3.503902
H 3.278594 -0.764905 -3.700999
H 4.209598 -3.26702 -1.015015
H 2.954061 -2.865349 -2.184817
H 5.184279 -2.214712 -3.069613
H 5.485662 -1.234853 -1.63678
C 1.788046 0.343852 1.723456
C 3.054666 1.157054 2.006365
C 0.685569 0.669069 2.730423
H 2.018548 -0.724373 1.806443
C 3.535009 0.960276 3.43792
H 2.84001 2.223441 1.836043
H 3.848553 0.884835 1.296604
C 1.179448 0.500395 4.162569
H 0.336193 1.703222 2.577033
H -0.17279 0.005938 2.569167
C 2.438527 1.310659 4.433391
H 4.431564 1.56732 3.618219
H 3.832484 -0.089828 3.57826
H 0.38223 0.777748 4.864363
H 1.392094 -0.564976 4.337449
H 2.785692 1.14526 5.461519
H 2.205694 2.383483 4.350145
H -2.567287 5.702218 -0.148572
H -5.925115 -2.3778 1.510991
N -2.751514 -1.286807 1.43839
N -2.243248 2.361568 -0.062091

3a-Cy

E=-2493.03447512

P 1.647801 -0.031362 0.208348
C -0.188105 -0.224962 -0.073874
S -0.519974 0.338861 -1.78136
S -0.949593 -1.834168 0.280718
O 0.568122 -0.126929 -2.639318
C -0.380939 2.088331 -1.557458
O -1.903674 0.011164 -2.118192
H -0.708677 0.496334 0.567326
C -1.474846 2.784162 -1.053696
C 0.836277 2.715776 -1.798048
O -0.667086 -2.724112 -0.844783
O -0.495817 -2.177777 1.631379
C -2.696455 -1.575439 0.387765
H -2.420738 2.270962 -0.89243
C -1.331856 4.134218 -0.766421
C 0.957349 4.066881 -1.507572
H 1.66875 2.141714 -2.196009
C -3.512356 -1.95235 -0.672812
C -3.222778 -1.040072 1.557926
H -2.180913 4.690368 -0.3733
C -0.114898 4.792273 -0.975832
H 1.90509 4.569499 -1.692193
H -3.065258 -2.35998 -1.57467
C -4.881715 -1.77878 -0.55324
C -4.596281 -0.860525 1.652295
H -2.5687 -0.781334 2.388194
C 0.046631 6.23735 -0.618837
H -5.530163 -2.07164 -1.377268
C -5.445231 -1.227656 0.60405
H -5.019282 -0.438053 2.561844
H -0.918392 6.752033 -0.578657
H 0.692196 6.756465 -1.335298
H 0.515407 6.335613 0.36987
C -6.928518 -1.055338 0.715376
H -7.334731 -0.551051 -0.169263
H -7.201144 -0.47462 1.601736
H -7.426971 -2.031088 0.784448
C 2.353209 -1.621013 -0.4691
C 3.638898 -1.292142 -1.231813
C 2.573489 -2.765791 0.513609
H 1.605818 -1.932883 -1.210567
C 4.193236 -2.525107 -1.932885
H 4.391295 -0.896394 -0.530115
H 3.433807 -0.496027 -1.95904
C 3.119705 -3.993999 -0.204515
H 3.29239 -2.450992 1.286337
H 1.639512 -3.00995 1.029019
C 4.402516 -3.676635 -0.959541
H 5.130948 -2.276942 -2.447276
H 3.480835 -2.836589 -2.711832
H 3.286913 -4.805018 0.516473
H 2.358167 -4.355098 -0.912246
H 4.766529 -4.56569 -1.490965
H 5.186762 -3.401633 -0.237021
C 1.799384 -0.11658 2.067911
C 3.196074 0.413559 2.424816
C 0.739669 0.720936 2.783826
H 1.697972 -1.159557 2.381593
C 3.34018 1.909201 2.155316
H 3.972809 -0.145912 1.883112
H 3.36626 0.21443 3.493601
C 0.851349 2.20528 2.45743
H -0.264886 0.328681 2.584851
H 0.89411 0.587119 3.865504
C 2.237878 2.722347 2.82355
H 3.304951 2.085652 1.070767
H 4.325725 2.252576 2.494934
H 0.665481 2.376412 1.38318
H 0.07892 2.768863 2.996187
H 2.356304 2.664314 3.916641
H 2.335436 3.782436 2.555545

3b'-iPr

E=-2212.51590569

P 0.325478 1.987252 0.363766
C 0.018835 0.290093 0.178548
S -0.734278 -0.356508 -1.211397
S 0.67734 -0.812568 1.309758
O 0.070559 -1.440931 -1.785653
O -1.097029 0.782765 -2.069151
C -2.276125 -1.121563 -0.677234
C -2.496227 -2.463806 -0.94474
H 1.363629 2.038263 1.305857
C 0.930662 2.825275 -1.143843
C -1.063853 2.923035 1.096823
O 0.975614 -0.075549 2.543696
O -0.158016 -2.01613 1.361769
C 2.264776 -1.326465 0.637432
C -3.714922 -2.993854 -0.536178
H -1.732273 -3.053114 -1.442225
C -4.286364 -0.831315 0.319152
H 0.064457 2.820695 -1.815894
C 2.050176 2.018713 -1.787063
C 1.329063 4.259331 -0.815075
H -0.619843 3.879979 1.407955
C -1.565805 2.170276 2.322709
C -2.160238 3.162096 0.066449
C 2.489307 -2.655318 0.315338
H -3.946536 -4.041876 -0.714091
C -4.626192 -2.165456 0.10701
H -4.97799 -0.155755 0.823443
H 2.927855 1.957208 -1.133786
H 2.349451 2.502393 -2.724362
H 1.723591 0.998736 -2.016969
H 2.139829 4.294978 -0.076575
H 0.487274 4.84849 -0.433459
H 1.687354 4.751971 -1.726394
H -0.765029 1.971313 3.043101
H -1.99671 1.207704 2.022196
H -2.34523 2.760248 2.819174
H -2.529034 2.207183 -0.324586
H -1.811952 3.77467 -0.773412
H -2.996588 3.688696 0.541498
C 4.326124 -0.657655 -0.010677
C 3.740562 -2.972498 -0.202985
H 1.705715 -3.392199 0.463031
H 5.036749 0.160234 -0.136554
C 4.675687 -1.958569 -0.367707
H 3.979041 -3.997832 -0.477905
H 5.663382 -2.165147 -0.772324
H -5.587929 -2.542918 0.445445
N 3.134571 -0.344644 0.485539
N -3.124886 -0.31716 -0.069321

3a'-iPr

E=-2259.16329537

S -0.9643 -0.050831 -1.221293
O -0.374618 -1.17849 -1.955956
C -0.069879 0.389914 0.174092
O -1.13368 1.217724 -1.960148
C -2.566998 -0.594295 -0.680427
P 0.748284 1.920891 0.205883
S 0.390874 -0.80134 1.329121
C -3.085182 -1.770938 -1.19917
C -3.303619 0.185121 0.205342
H 1.758155 1.72893 1.166073
C 1.637838 2.361 -1.33069
C -0.222046 3.322384 0.866221
O 0.550159 -0.135284 2.629012
O -0.529153 -1.931868 1.18616
C 2.016625 -1.352089 0.859003

C -4.362247 -2.170426 -0.820993
H -2.483579 -2.366508 -1.880391
C -4.573683 -0.227069 0.573895
H -2.87586 1.095076 0.619661
H 0.84511 2.568308 -2.059191
C 2.437098 1.150284 -1.801636
C 2.490848 3.604313 -1.111938
H 0.523522 4.118868 1.004213
C -0.778581 2.921527 2.227194
C -1.287938 3.79461 -0.112566
C 3.124887 -0.890229 1.556677
C 2.157056 -2.172017 -0.258297
H -4.772261 -3.096212 -1.221282
C -5.125354 -1.410842 0.067096
H -5.14949 0.37709 1.273201
H 3.177388 0.832969 -1.054359
H 2.974724 1.408242 -2.721511
H 1.782357 0.298712 -2.015174
H 3.235754 3.451136 -0.320622
H 1.888449 4.483061 -0.855458
H 3.031615 3.83737 -2.036448
H 0.019565 2.694809 2.942184
H -1.402837 2.023267 2.150833
H -1.386405 3.739152 2.631303
H -2.007669 3.000416 -0.333003
H -0.855648 4.115245 -1.067475
H -1.826234 4.645765 0.320271
H 2.983784 -0.266839 2.43653
C 4.396331 -1.240565 1.113395
C 3.431396 -2.51143 -0.683259
H 1.273963 -2.506184 -0.797272
C -6.496817 -1.846868 0.483176
H 5.269876 -0.880573 1.654712
C 4.571071 -2.049042 -0.011199
H 3.549371 -3.147087 -1.559692
H -6.540323 -2.022225 1.565564
H -6.79484 -2.770304 -0.022613
H -7.241847 -1.074721 0.254388
C 5.939306 -2.43085 -0.487402
H 6.720317 -1.907124 0.072362
H 6.065325 -2.201225 -1.552357
H 6.105947 -3.50964 -0.371571

3b-iPr

E=-2212.51094278

S 0.015364 0.993124 -1.289132
O 0.972872 0.691775 -2.351673
C 0.132601 -0.243172 0.034862
C 0.603848 2.452543 -0.41959
O -1.394745 1.178537 -1.622731
P 1.932561 -0.639731 0.312209
H -0.260202 0.280967 0.918108
S -1.036157 -1.586672 -0.266269
C 1.595857 3.227529 -0.996921
O -0.97449 -1.97699 -1.673535
O -0.813735 -2.588079 0.776657
C -2.628993 -0.83307 0.068922
C 0.36702 3.788958 1.386394
C 1.968291 4.373459 -0.301402
H 2.050815 2.933627 -1.938471
C -3.655243 -0.960601 -0.851078
H -0.13173 3.9809 2.33631
C 1.346003 4.658 0.90736
H 2.740457 5.029664 -0.696598
C -3.863214 0.331103 1.557137
C -4.873169 -0.392073 -0.495726
H -3.490408 -1.467094 -1.79776
H -3.912994 0.84668 2.516021
C -4.978121 0.265334 0.724607
H -5.725886 -0.453667 -1.168228
H 1.615898 5.540323 1.482151
H -5.911995 0.729609 1.031341

N -2.699102 -0.221741 1.233225
N -0.004104 2.69882 0.724326
C 1.885406 -1.605786 1.904937
C 1.034069 -0.892654 2.946819
C 3.330646 -1.775284 2.362669
H 1.445191 -2.588313 1.707618
H 1.178418 -1.359844 3.929371
H 1.307551 0.167844 3.041929
H -0.032505 -0.959616 2.704194
H 3.948393 -2.273342 1.604387
H 3.792995 -0.804674 2.583936
H 3.370152 -2.38621 3.273553
C 2.29094 -1.878041 -1.053198
C 2.10193 -3.345407 -0.702641
C 3.687567 -1.593569 -1.595924
H 1.565689 -1.605335 -1.8293
H 1.083385 -3.559459 -0.367422
H 2.303359 -3.960538 -1.589356
H 2.796257 -3.669559 0.083299
H 3.776887 -0.553301 -1.928961
H 4.461403 -1.774571 -0.837435
H 3.903747 -2.246486 -2.451372

3a-iPr

E=-2259.16089846

S 0.443038 0.450548 -1.578019
O 1.076032 -0.381167 -2.596828
C 0.081135 -0.598275 -0.118151
C 1.64599 1.563801 -0.912753
O -0.763522 1.212477 -1.89791
P 1.639372 -1.580296 0.288716
H -0.07879 0.107116 0.707414
S -1.544325 -1.404 -0.342665
C 1.211758 2.648599 -0.15618
C 2.996867 1.312432 -1.118999
O -1.659944 -1.794162 -1.746017
O -1.6675 -2.405504 0.716867
C -2.751725 -0.155558 0.00427
H 0.147214 2.834627 -0.027985
C 2.158397 3.480026 0.4218
C 3.929091 2.162811 -0.539759
H 3.298482 0.456151 -1.716244
C -3.45199 0.428842 -1.042821
C -3.014599 0.172837 1.330475
H 1.831126 4.327929 1.020971
C 3.528463 3.250454 0.24228
H 4.990442 1.974768 -0.691286
H -3.213814 0.153671 -2.065945
C -4.428005 1.369405 -0.74833
C -3.983801 1.126742 1.603508
H -2.474552 -0.317243 2.138114
C 4.538432 4.17103 0.853916
H -4.981488 1.835385 -1.561658
C -4.707317 1.736199 0.572222
H -4.19117 1.397114 2.63732
H 4.238263 4.478718 1.861557
H 4.637 5.085193 0.25302
H 5.526298 3.703555 0.909784
C -5.778669 2.736579 0.878294
H -5.5668 3.280671 1.804553
H -6.74628 2.232981 1.009242
H -5.895683 3.459906 0.064748
C 1.215291 -3.364433 -0.023716
C 2.277492 -4.228433 0.646662
C 1.170935 -3.596834 -1.529384
H 0.237292 -3.590528 0.411477
H 3.289687 -3.942065 0.328701
H 2.233454 -4.171767 1.740575
H 2.128032 -5.278497 0.36538
H 2.147793 -3.393185 -1.986525
H 0.907703 -4.641985 -1.738011

H 0.431392 -2.9544 -2.016427
C 1.688049 -1.388598 2.141279
C 0.516715 -1.942967 2.931332
C 1.975104 0.069301 2.489869
H 2.591083 -1.967639 2.38885
H -0.396147 -1.367789 2.735728
H 0.300788 -2.987107 2.680224
H 0.722985 -1.884443 4.008378
H 1.091126 0.704398 2.337209
H 2.246115 0.159822 3.549075
H 2.792265 0.487384 1.888211

3b'-Ph

E=-2439.12919381

S -0.336162 -0.80387 -1.260023
O -0.732004 0.452429 -1.915232
O 0.654678 -1.671359 -1.903683
C 0.165222 -0.361124 0.314228
C -1.83413 -1.778919 -1.060203
S 0.917445 -1.522686 1.317832
P -0.029178 1.276071 0.816844
N -2.842649 -1.120609 -0.52457
C -1.856686 -3.107247 -1.453945
O 0.379415 -2.850975 1.014622
O 0.891108 -1.024844 2.69783
C 2.642959 -1.531861 0.812545
H 0.434263 1.27275 2.14017
C -3.978523 -1.782616 -0.346505
H -0.969552 -3.568747 -1.876862
C -3.052655 -3.792892 -1.26638
N 3.230335 -0.351455 0.885846
C 3.244048 -2.704577 0.385861
C -4.131493 -3.121626 -0.703751
H -4.802506 -1.22647 0.103047
H -3.137746 -4.839248 -1.552039
C 4.506147 -0.28165 0.524447
H 2.67487 -3.628587 0.345766
C 4.580928 -2.621076 0.009571
H -5.080718 -3.625192 -0.537658
C 5.223532 -1.391419 0.080296
H 4.980278 0.698934 0.581904
H 5.111143 -3.504828 -0.339101
H 6.265857 -1.284989 -0.209835
C 0.924986 2.483704 -0.115538
C 0.76982 3.845783 0.153224
C 1.842003 2.049793 -1.072644
C 1.539661 4.773348 -0.537713
H 0.047456 4.184144 0.894999
C 2.607803 2.984354 -1.758538
H 1.946637 0.987258 -1.27965
C 2.458001 4.34203 -1.491167
H 1.420923 5.834878 -0.332031
H 3.321473 2.649827 -2.508198
H 3.058528 5.070483 -2.032352
C -1.751552 1.776594 0.8867
C -2.519889 1.288782 1.945892
C -2.346226 2.473586 -0.163376
C -3.888615 1.517014 1.960492
H -2.050988 0.715965 2.745081
C -3.718224 2.694504 -0.142542
H -1.742317 2.816313 -1.000531
C -4.486567 2.216613 0.914617
H -4.491594 1.14351 2.785363
H -4.190526 3.234156 -0.960521
H -5.561024 2.38953 0.924175

3a'-Ph

E=-2485.78117484

P -0.115308 1.248552 0.959496
H 0.087198 1.280954 2.34555
C 0.201011 -0.374687 0.466938
S 1.171495 -1.41179 1.441501
S -0.383692 -0.859731 -1.07728
O 1.331959 -0.717706 2.728747
O 0.64007 -2.776781 1.426925
C 2.783306 -1.476721 0.694895
O -0.412242 0.341796 -1.930118
O 0.369673 -2.03349 -1.520825
C -2.082797 -1.333892 -0.849152
C 3.680575 -0.446303 0.951827
C 3.114876 -2.528081 -0.150689
C -3.05909 -0.774143 -1.659881
C -2.404181 -2.248069 0.150001
C 4.925848 -0.468099 0.338681
C 4.365584 -2.534919 -0.752103
C -4.386897 -1.133564 -1.457552
C -3.734383 -2.587316 0.341461
C 5.288683 -1.509279 -0.521235
C -4.746929 -2.033273 -0.452967
C 6.641418 -1.542369 -1.164782
H 7.296829 -2.260464 -0.653637
H 6.576361 -1.85678 -2.212584
H 7.12886 -0.563066 -1.125498
C -6.184058 -2.374503 -0.201699
H -6.569631 -1.810495 0.658862
H -6.81188 -2.129971 -1.06446
H -6.308778 -3.438312 0.029508
H 4.631601 -3.353406 -1.419395
H 2.387468 -3.313142 -0.33572
H 3.40291 0.361158 1.626128
H 5.632064 0.337765 0.532275
H -5.160807 -0.693105 -2.08442
H -2.776307 -0.05067 -2.420324
H -1.615198 -2.659842 0.778191
H -3.99748 -3.294337 1.126974
C -1.835034 1.694028 0.675998
C -2.202332 2.503642 -0.397683
C -2.806563 1.11186 1.49555
C -3.5501 2.749521 -0.635906
H -1.439914 2.923474 -1.050278
C -4.148726 1.364375 1.25073
H -2.51507 0.455448 2.314393
C -4.518752 2.184544 0.186918
H -3.843277 3.380401 -1.472095
H -4.90853 0.91576 1.886981
H -5.572181 2.378028 -0.005393
C 0.961714 2.495635 0.235334
C 0.871296 3.824832 0.658874
C 1.903004 2.117575 -0.721581
C 1.730747 4.773824 0.122208
H 0.133045 4.118925 1.404116
C 2.761211 3.074472 -1.251766
H 1.949625 1.082415 -1.051677
C 2.67554 4.397383 -0.830601
H 1.665867 5.809354 0.448864
H 3.497427 2.783079 -1.997582
H 3.349061 5.143501 -1.247498

3b-Ph

E=-2439.130889

S -1.394637 0.295408 -1.630145
O -0.681389 0.284827 -2.905063
C -0.260646 -0.076005 -0.261224
C -1.801912 2.003447 -1.245372
O -2.585999 -0.530758 -1.454953
P 1.282425 0.960266 -0.421292
H -0.808434 0.26455 0.630214
S -0.134121 -1.881174 -0.082669

C -1.734674 2.958017 -2.246588
C 2.538603 -0.26406 -0.947488
C 1.682557 1.299819 1.3276
O -0.187153 -2.4997 -1.403603
O 0.97473 -2.163445 0.821727
C -1.652954 -2.267238 0.794536
C -2.512896 3.454642 0.333507
C -2.107739 4.251022 -1.893058
H -1.396583 2.691748 -3.24397
C 3.659781 -0.567213 -0.174046
C 2.39147 -0.846255 -2.209998
C 1.200877 0.581296 2.426093
C 2.526186 2.395387 1.5471
C -2.491689 -3.258156 0.315108
H -2.805479 3.624381 1.369526
C -2.501592 4.503149 -0.585012
H -2.081252 5.051172 -2.62934
H 3.7785 -0.12509 0.812764
C 4.621564 -1.447284 -0.657496
C 3.347725 -1.738786 -2.67974
H 1.514045 -0.615856 -2.812219
H 0.548425 -0.276727 2.287644
C 1.56215 0.956022 3.716078
C 2.90131 2.753366 2.836824
H 2.890055 2.974992 0.698859
C -2.919143 -1.823193 2.611924
C -3.619634 -3.532674 1.081417
H -2.268619 -3.769469 -0.616866
H 5.492444 -1.680138 -0.047525
C 4.466875 -2.037334 -1.907984
H 3.218459 -2.199637 -3.657165
H 1.17689 0.39278 4.563878
C 2.41561 2.034909 3.924887
H 3.56324 3.603071 2.991773
H -3.06673 -1.228843 3.513342
C -3.837149 -2.804717 2.245196
H -4.323786 -4.299793 0.767065
H 5.218069 -2.730903 -2.281018
H 2.698469 2.31919 4.936619
H -2.791836 5.502448 -0.270542
H -4.712087 -2.987304 2.86393
N -2.17144 2.214535 0.002826
N -1.832796 -1.562915 1.892742

3a-Ph

E=-2485.78355964

S 2.042066 1.168229 -1.191393
O 2.151285 0.971334 -2.631834
C 0.419099 0.466914 -0.661827
C 3.280768 0.239388 -0.350459
O 2.053985 2.525006 -0.638653
P -0.081554 1.297782 0.947257
H -0.242403 0.696143 -1.507562
S 0.528067 -1.349682 -0.587266
C 3.531935 0.521694 0.989515
C 3.989329 -0.737344 -1.039511
C -1.541992 0.30611 1.461066
C -0.950802 2.782114 0.297914
O 1.293445 -1.758074 -1.763993
O 0.990421 -1.713174 0.750889
C -1.127527 -1.922084 -0.807949
H 2.97652 1.306823 1.496592
C 4.502536 -0.214077 1.651133
C 4.964548 -1.451953 -0.360973
H 3.763484 -0.927406 -2.084565
C -2.784407 0.374176 0.819858
C -1.402979 -0.488648 2.600217
C -0.868503 3.274991 -1.005341
C -1.724668 3.482106 1.232618
C -1.832331 -1.568864 -1.954307
C -1.655302 -2.79481 0.133546

H 4.708628 -0.005156 2.699301
C 5.228705 -1.211997 0.991573
H 5.532681 -2.214647 -0.890486
H -2.904681 0.995265 -0.066431
C -3.866737 -0.339907 1.314719
C -2.489801 -1.203683 3.095987
H -0.437094 -0.545102 3.099275
H -0.246228 2.789363 -1.751664
C -1.562139 4.426198 -1.369655
C -2.414864 4.630313 0.868234
H -1.797759 3.115931 2.256785
H -1.388877 -0.919253 -2.70689
C -3.116397 -2.062857 -2.123773
C -2.93068 -3.300724 -0.070225
H -1.073523 -3.053648 1.01427
C 6.252869 -2.020451 1.725482
H -4.82841 -0.286633 0.8075
C -3.721622 -1.125401 2.457216
H -2.373592 -1.816493 3.98775
H -1.4876 4.792635 -2.391644
C -2.338255 5.105385 -0.438485
H -3.014766 5.156328 1.60839
H -3.684851 -1.779666 -3.00768
C -3.687941 -2.928677 -1.184179
H -3.356087 -3.981829 0.664617
H 5.78083 -2.891039 2.201185
H 6.731648 -1.436581 2.518501
H 7.027103 -2.39716 1.049369
H -4.573384 -1.679686 2.847069
H -2.878253 6.004929 -0.72711
C -5.09313 -3.417595 -1.347981
H -5.215003 -4.429854 -0.948468
H -5.402418 -3.414086 -2.397998
H -5.78616 -2.765317 -0.798144

4b-Cy

E=-2445.90738253

S -0.295348 -0.941314 -1.257378
O -0.692714 0.191848 -2.111677
O 0.750862 -1.83686 -1.791853
C 0.076237 -0.354413 0.301543
C -1.771713 -1.983098 -1.141883
S 0.671606 -1.524665 1.381009
P 0.021762 1.359549 0.806106
N -2.862224 -1.382653 -0.705844
C -1.704058 -3.315361 -1.528304
O 0.148397 -2.852717 1.006108
O 0.507797 -1.082046 2.770238
C 2.457706 -1.648653 1.119582
C 0.877556 2.30961 -0.562934
C -1.790733 1.729448 0.537179
C -3.974232 -2.105792 -0.624153
H -0.759226 -3.72816 -1.867004
C -2.870906 -4.063205 -1.443384
N 3.195072 -0.703628 1.678059
C 2.959745 -2.677666 0.330113
H 0.177907 2.414907 -1.404716
C 2.121906 1.578601 -1.061734
C 1.27665 3.695144 -0.047831
H -2.073056 1.237017 -0.40358
C -2.573854 1.068519 1.668593
C -2.136708 3.210176 0.436094
C -4.030056 -3.44983 -0.980703
H -4.864735 -1.593842 -0.255454
H -2.875403 -5.113402 -1.729313
C 4.507646 -0.744908 1.471153
H 2.275727 -3.399493 -0.10382
C 4.332014 -2.710927 0.120816
H 1.85369 0.595381 -1.460614
H 2.789489 1.391206 -0.205297
C 2.85942 2.383674 -2.124093
C 1.994312 4.511296 -1.115859

H 0.402693 4.243494 0.324947
H 1.9443 3.56452 0.818599
H -2.312384 0.004033 1.714643
H -2.26401 1.513835 2.628047
C -4.072394 1.255603 1.485226
C -3.640433 3.412931 0.274716
H -1.791 3.735622 1.34208
H -1.614171 3.668334 -0.41343
H -4.963744 -4.000383 -0.893266
C 5.124999 -1.727465 0.702424
H 5.101411 0.043131 1.937811
H 4.77798 -3.493771 -0.490005
H 3.759859 1.843831 -2.448271
H 2.213499 2.472196 -3.011292
C 3.223848 3.776889 -1.63116
H 1.303646 4.695026 -1.953629
H 2.272807 5.497149 -0.71875
H -4.377133 0.744127 0.560007
H -4.624158 0.781113 2.308546
C -4.42843 2.733037 1.386297
H -3.877027 4.485286 0.235681
H -3.949864 2.989037 -0.69289
H 6.203523 -1.716439 0.563583
H 3.955313 3.688254 -0.812638
H 3.71442 4.353723 -2.427156
H -5.507287 2.86376 1.224619
H -4.195442 3.223438 2.344691

4a-Cy

E=-2492.55842614

P 0.255522 1.158428 0.875986
C 0.104773 -0.50683 0.225693
C -1.520552 1.713209 0.716319
C 1.164391 2.14669 -0.427325
S 0.751748 -1.752198 1.201468
S -0.57387 -0.873155 -1.297509
C -2.336303 0.971225 1.772426
C -1.76283 3.214113 0.816161
C 1.728162 3.420559 0.2079
C 2.302765 1.329715 -1.03361
O 0.628065 -1.393906 2.624577
O 0.237421 -3.073737 0.799485
C 2.507469 -1.7744 0.865403
O -0.564351 0.347112 -2.131281
O 0.006516 -2.082509 -1.907945
C -2.321467 -1.229442 -1.073698
C -3.821825 1.254953 1.616921
C -3.251966 3.524234 0.684478
C 2.52685 4.246943 -0.792931
C 3.115785 2.145022 -2.029884
C 3.405697 -1.330152 1.822747
C 2.940511 -2.153566 -0.403468
C -3.242738 -0.631572 -1.922952
C -2.739916 -2.110485 -0.081075
C -4.092394 2.752518 1.694421
C 3.652441 3.426465 -1.407392
C 4.759103 -1.254605 1.50085
C 4.29024 -2.077838 -0.705888
C -4.596981 -0.914068 -1.771117
C -4.093548 -2.377529 0.062026
C 5.222347 -1.62205 0.237545
C -5.045602 -1.783904 -0.776346
C 6.67725 -1.53353 -0.114344
H 7.080483 -2.518471 -0.382964
H 6.835546 -0.877847 -0.980092
H 7.268405 -1.142056 0.719788
C -6.506703 -2.062454 -0.588504
H -6.877984 -1.599618 0.335728
H -7.100195 -1.668625 -1.419997
H -6.700692 -3.138721 -0.506784
H 4.633789 -2.366467 -1.698902

H 2.20296 -2.460711 -1.144055
H 3.03488 -1.037092 2.801806
H 5.468893 -0.899592 2.247244
H -5.320335 -0.442964 -2.435793
H -2.890192 0.056906 -2.686837
H -1.998164 -2.569254 0.570999
H -4.422582 -3.061069 0.844081
H -2.001077 1.297891 2.76996
H -2.126831 -0.103812 1.714564
H -4.156452 0.864593 0.643448
H -4.399042 0.720273 2.38379
H -3.84805 3.105873 2.708567
H -5.159427 2.964058 1.540903
H -3.426023 4.604128 0.787665
H -3.576333 3.251731 -0.331696
H -1.208473 3.745403 0.031756
H -1.387259 3.588603 1.782638
H 0.926826 4.027616 0.648608
H 2.383601 3.130688 1.044633
H 2.927436 5.148001 -0.308389
H 1.853802 4.594418 -1.592181
H 4.201278 4.019423 -2.15177
H 4.376171 3.166621 -0.618896
H 2.474072 2.401048 -2.887252
H 3.940177 1.537317 -2.428307
H 2.963146 0.973404 -0.225063
H 1.898904 0.434179 -1.515349
H 0.46179 2.412306 -1.230382
H -1.862876 1.396037 -0.279417

4b-iPr

E=-2212.03313632

S -0.774694 -0.272308 -1.13372
O -0.0055 -1.374132 -1.748268
C 0.016849 0.372971 0.235161
O -1.188655 0.814596 -2.036604
C -2.324184 -1.051778 -0.602468
P 0.570468 2.050617 0.524132
S 0.643465 -0.822158 1.282178
C -2.561365 -2.382513 -0.922564
C 0.99824 2.729489 -1.161738
C -0.957358 3.016771 1.022185
O 0.932474 -0.25022 2.60314
O -0.200331 -2.031454 1.222291
C 2.24741 -1.349448 0.631856
C -3.7812 -2.923555 -0.538664
H -1.799477 -2.953003 -1.444106
C -4.342054 -0.796558 0.398578
H 0.108257 2.682889 -1.796826
C 2.080783 1.865351 -1.792669
C 1.442432 4.178459 -1.012421
H -0.550597 4.00007 1.310235
C -1.534178 2.355595 2.267624
C -2.023338 3.209891 -0.042079
C 2.365149 -2.591757 0.018546
H -4.018095 -3.962548 -0.760342
C -4.691426 -2.117928 0.137268
H -5.031941 -0.135885 0.926047
H 2.979003 1.826686 -1.16148
H 2.371397 2.265642 -2.774218
H 1.730732 0.836096 -1.936279
H 2.305697 4.262887 -0.337309
H 0.641981 4.812623 -0.609629
H 1.735787 4.597776 -1.984915
H -0.770179 2.199932 3.03942
H -1.94402 1.37158 2.007291
H -2.343487 2.964619 2.694194
H -2.415611 2.239138 -0.362481
H -1.635 3.728384 -0.92741
H -2.857784 3.808524 0.353776
C 4.424748 -0.824998 0.279467

C 3.612505 -2.941717 -0.48156
H 1.495011 -3.235564 -0.058324
H 5.229363 -0.096691 0.395654
C 4.665705 -2.043218 -0.349555
H 3.761345 -3.903254 -0.96953
H 5.658279 -2.277207 -0.727318
H -5.65609 -2.502812 0.459446
N 3.236184 -0.482171 0.76514
N -3.177349 -0.271879 0.032078

4a-iPr

E=-2258.68265636

S -0.833567 -0.273349 -1.192019
O -0.31051 -1.488057 -1.844295
C 0.038181 0.141626 0.21877
O -0.952789 0.918398 -2.055037
C -2.528014 -0.667956 -0.741328
P 0.49476 1.786308 0.768453
S 0.796895 -1.098664 1.135841
C -2.789981 -1.598558 0.261284
C -3.568155 -0.058648 -1.42894
C 1.016281 2.73702 -0.748388
C -1.097293 2.616429 1.312754
O 0.863435 -0.711031 2.555586
O 0.219128 -2.423009 0.841691
C 2.495568 -1.169053 0.580106
C -4.105659 -1.90457 0.574347
H -1.957928 -2.061767 0.788491
C -4.883625 -0.377081 -1.103078
H -3.336816 0.665014 -2.206621
H 0.164701 2.825738 -1.430166
C 2.118446 1.967864 -1.461675
C 1.471894 4.123735 -0.312027
H -0.745866 3.490524 1.88418
C -1.808512 1.675608 2.277675
C -2.030979 3.097935 0.214639
C 3.524252 -0.784928 1.425425
C 2.754839 -1.570336 -0.728664
H -4.311385 -2.628972 1.3619
C -5.175418 -1.300358 -0.099033
H -5.699912 0.104723 -1.64016
H 2.955583 1.737998 -0.785567
H 2.515451 2.551066 -2.304564
H 1.734744 1.019152 -1.852229
H 2.314003 4.061866 0.391054
H 0.667203 4.682312 0.184689
H 1.798984 4.717552 -1.176855
H -1.110009 1.222991 2.992318
H -2.275459 0.850158 1.725623
H -2.595303 2.203674 2.833439
H -2.333429 2.264502 -0.428044
H -1.56151 3.854629 -0.425623
H -2.937056 3.546711 0.647594
H 3.288136 -0.475697 2.44042
C 4.833934 -0.795515 0.950455
C 4.063161 -1.580279 -1.184001
H 1.918639 -1.832508 -1.376197
C -6.591016 -1.634025 0.265093
H 5.645654 -0.488863 1.609238
C 5.125093 -1.191127 -0.354949
H 4.271382 -1.888155 -2.208301
H -6.823583 -1.304837 1.286384
H -6.764811 -2.716752 0.232249
H -7.303423 -1.153269 -0.413078
C 6.53355 -1.201186 -0.869332
H 7.24007 -0.849792 -0.110448
H 6.636941 -0.558909 -1.753194
H 6.836955 -2.2115 -1.172846

4b-Ph

E=-2438.6600638

S -0.443696 -0.744296 -1.105322
O -0.892882 0.458073 -1.819884
O 0.568571 -1.588855 -1.77179
C 0.036622 -0.301032 0.476751
C -1.907587 -1.798792 -0.961603
S 0.791058 -1.534766 1.377129
P 0.102081 1.356913 1.09901
N -2.96094 -1.239205 -0.39832
C -1.872365 -3.090669 -1.469287
O 0.257929 -2.848811 0.977781
O 0.802885 -1.192599 2.803908
C 2.523731 -1.545662 0.865524
C -4.068569 -1.965949 -0.305331
H -0.956603 -3.470484 -1.911389
C -3.036108 -3.843076 -1.370869
N 3.1988 -0.445814 1.153762
C 3.042718 -2.638551 0.185268
C -4.156683 -3.273064 -0.777412
H -4.928563 -1.487974 0.166757
H -3.067146 -4.863065 -1.74944
C 4.467236 -0.384278 0.765803
H 2.407182 -3.49398 -0.020798
C 4.371648 -2.565556 -0.214948
H -5.086024 -3.829028 -0.677952
C 5.099873 -1.418663 0.079673
H 5.009206 0.532017 1.005591
H 4.83193 -3.391398 -0.75426
H 6.140588 -1.319348 -0.219569
C 1.049448 2.339165 -0.126249
C 1.054783 3.734223 -0.008266
C 1.881197 1.749702 -1.081962
C 1.85151 4.520161 -0.833235
H 0.419693 4.212755 0.7383
C 2.689798 2.534396 -1.897954
H 1.888474 0.667474 -1.19579
C 2.677346 3.921355 -1.781025
H 1.831999 5.604237 -0.731123
H 3.329765 2.055824 -2.637909
H 3.307261 4.532836 -2.424879
C -1.620253 1.968601 0.887316
C -2.515178 1.639324 1.909909
C -2.087244 2.706517 -0.201841
C -3.8496 2.021474 1.840951
H -2.157962 1.059516 2.761407
C -3.419404 3.102849 -0.265329
H -1.406696 2.946552 -1.014973
C -4.305408 2.760406 0.752121
H -4.534419 1.74905 2.642705
H -3.771377 3.673524 -1.123696
H -5.348409 3.068162 0.696731

4a-Ph

E=-2485.31221476

P 0.096287 1.114708 1.237655
C 0.214127 -0.411936 0.332197
S 1.067093 -1.69747 1.076013
S -0.489653 -0.600525 -1.220911
O 1.077261 -1.487809 2.532532
O 0.606479 -3.006173 0.58309
C 2.779617 -1.56302 0.570644
O -0.568046 0.712751 -1.882956
O 0.147345 -1.69107 -1.980788
C -2.190861 -1.099941 -0.971246
C 3.751053 -1.260907 1.512422
C 3.114697 -1.744008 -0.770175
C -3.201972 -0.411767 -1.62364
C -2.478852 -2.171387 -0.131759

C 5.076602 -1.131591 1.105644
C 4.438748 -1.611908 -1.157748
C -4.523535 -0.797048 -1.42322
C -3.801729 -2.538885 0.062138
C 5.442553 -1.30314 -0.229303
C -4.846187 -1.85551 -0.573554
C 6.868236 -1.160762 -0.67084
H 7.239414 -2.092783 -1.1162
H 6.969257 -0.380126 -1.435566
H 7.523343 -0.90139 0.166957
C -6.274432 -2.231362 -0.314766
H -6.62476 -1.798911 0.632888
H -6.936249 -1.864802 -1.106592
H -6.396617 -3.317739 -0.236365
H 4.704428 -1.747112 -2.205814
H 2.326461 -1.956587 -1.491068
H 3.458887 -1.126003 2.55075
H 5.841916 -0.891109 1.842681
H -5.321977 -0.252143 -1.925787
H -2.944471 0.43079 -2.259904
H -1.663443 -2.688367 0.371844
H -4.03389 -3.371279 0.725805
C -1.670679 1.574494 1.014356
C -2.150373 2.537693 0.126168
C -2.588028 0.871269 1.804334
C -3.51443 2.807086 0.04803
H -1.453716 3.061448 -0.524091
C -3.950117 1.125468 1.712519
H -2.220998 0.102624 2.485528
C -4.417547 2.10559 0.839668
H -3.874173 3.562393 -0.649407
H -4.64972 0.559361 2.32574
H -5.483963 2.313772 0.770378
C 1.01394 2.358664 0.250848
C 0.907372 3.71543 0.578229
C 1.952619 1.976757 -0.710742
C 1.697457 4.667489 -0.057169
H 0.192083 4.031803 1.338431
C 2.755587 2.925568 -1.334092
H 2.039698 0.925019 -0.976403
C 2.628694 4.274742 -1.015135
H 1.590708 5.719784 0.202202
H 3.481128 2.607596 -2.081509
H 3.253627 5.017 -1.508848

5b-Ph

E = -2439.55477185

S -1.315123 -0.042552 -1.663062
O -0.422159 -0.074954 -2.817797
C -0.239471 -0.161171 -0.189667
C -1.951309 1.614163 -1.438161
O -2.398813 -1.002621 -1.532360
P 1.394978 0.636734 -0.469362
H -0.741062 0.343745 0.648674
S -0.098933 -1.900051 0.370370
C -3.264302 1.913514 -1.751282
C 2.655754 -0.583899 -0.797167
C 1.745999 1.699730 0.912312
O 0.011393 -2.784562 -0.777045
O 0.940918 -1.864288 1.394801
C -1.691593 -2.124501 1.154039
C -1.423439 3.717140 -0.791582
C -3.644443 3.241274 -1.571280
H -3.948912 1.147992 -2.106681
C 3.749336 -0.728502 0.059098
C 2.518191 -1.378048 -1.940538
C 1.647023 1.210617 2.219357
C 2.119995 3.023398 0.660899
C -2.423332 -3.266126 0.880433
H -0.674913 4.402363 -0.394984
C -2.714235 4.152380 -1.087176
H -4.660566 3.554719 -1.798907

H 3.844968 -0.108889 0.947908
C 4.717266 -1.677656 -0.240152
C 3.493643 -2.322974 -2.223668
H 1.650985 -1.267492 -2.588837
H 1.347711 0.180005 2.402375
C 1.931824 2.062060 3.278502
C 2.401671 3.860690 1.732182
H 2.185694 3.399201 -0.358309
C -3.182214 -1.284637 2.623784
C -3.624768 -3.396869 1.570203
H -2.071984 -3.999444 0.160225
H 5.573706 -1.798768 0.418711
C 4.588858 -2.470815 -1.376832
H 3.396153 -2.947657 -3.108211
H 1.856917 1.693225 4.298572
C 2.308368 3.380282 3.035283
H 2.691737 4.892262 1.547690
H -3.463413 -0.476803 3.298429
C -4.008012 -2.394706 2.452498
H -4.255768 -4.268107 1.411011
H 5.350300 -3.213785 -1.604331
H 2.528003 4.041651 3.870608
H -2.982900 5.193641 -0.930040
H -4.943709 -2.462196 3.001463
N -1.047902 2.455686 -0.972498
N -2.027229 -1.155771 1.979758
H 1.227619 1.424891 -1.609969

5a-Ph

E = -2486.20665974

S -1.969209 1.143068 0.146509
O -1.949215 1.911817 1.393112
C -0.380105 0.206208 0.271711
C -3.260900 -0.039281 0.227142
O -1.962888 1.803205 -1.148217
P 0.876927 1.362705 0.961812
H -0.532774 -0.590193 1.010783
S 0.089711 -0.578290 -1.318384
C -3.895139 -0.430032 -0.948327
C -3.659914 -0.513746 1.474687
C 0.749426 3.015106 0.309539
C 2.500201 0.623371 0.873172
O -1.162449 -1.017181 -1.914610
O 0.965922 0.365914 -2.008742
C 1.035022 -1.977848 -0.827740
H -3.561085 -0.031924 -1.901704
C -4.941035 -1.334474 -0.863184
C -4.702327 -1.425043 1.532485
H -3.171962 -0.163705 2.381284
C 0.861317 3.305764 -1.054327
C 0.522770 4.031660 1.245492
C 2.821673 -0.387142 1.787454
C 3.418429 1.036969 -0.094200
C 2.363310 -2.058460 -1.222754
C 0.421895 -2.991273 -0.091447
H -5.445447 -1.651062 -1.774028
C -5.357788 -1.849124 0.370239
H -5.022305 -1.808449 2.499351
H 0.990157 2.507838 -1.779640
C 0.770552 4.627411 -1.468178
C 0.435762 5.346605 0.813553
H 0.398640 3.799170 2.300845
H 2.113844 -0.703390 2.552025
C 4.070558 -0.986994 1.721624
C 4.666368 0.429198 -0.144660
H 3.162926 1.820738 -0.802913
H 2.805471 -1.258044 -1.809871
C 3.101569 -3.170742 -0.841751
C 1.181836 -4.083357 0.289083
H -0.631676 -2.930106 0.177908
C -6.503456 -2.807946 0.445118
H 0.852954 4.861589 -2.526688
C 0.563946 5.643605 -0.539989

H 0.258632 6.139331 1.536211
H 4.328426 -1.771894 2.428375
C 4.989596 -0.579349 0.757899
H 5.388415 0.747475 -0.892592
H 4.145992 -3.242297 -1.138638
C 2.532136 -4.190094 -0.075755
H 0.719481 -4.876381 0.873608
H -6.463776 -3.412574 1.356307
H -6.522171 -3.475633 -0.422448
H -7.455525 -2.259941 0.453266
H 0.491410 6.675811 -0.875745
H 5.968726 -1.051093 0.712142
C 3.333833 -5.382385 0.340865
H 2.952171 -6.291218 -0.141550
H 3.265275 -5.542787 1.423305
H 4.388107 -5.271746 0.071680
H 0.525997 1.441791 2.312708

5b-Cy

E = -2446.81579125

S 0.473238 -1.565512 1.057293
O -0.333931 -1.161733 2.207761
C 0.400850 -0.116362 -0.040972
C 2.202362 -1.578549 1.504899
O 0.133827 -2.769520 0.314216
P -1.329012 0.423174 -0.294383
H 0.966924 0.660690 0.488715
S 1.218212 -0.415308 -1.653829
C 2.904361 -2.769659 1.475137
O 2.222585 -1.454089 -1.491976
O 0.132071 -0.561926 -2.620254
C 2.030994 1.158718 -1.897890
C 3.944189 -0.339137 2.222536
C 4.235114 -2.698699 1.876686
H 2.433997 -3.691419 1.144621
C 3.350943 1.198541 -2.307378
H 4.332733 0.639846 2.500571
C 4.760301 -1.469398 2.254900
H 4.853814 -3.592887 1.883388
C 1.781263 3.402856 -1.833774
C 3.890907 2.468178 -2.500541
H 3.920238 0.285275 -2.457443
H 1.139386 4.258081 -1.625704
C 3.096863 3.581871 -2.261656
H 4.922298 2.580771 -2.826164
C -1.946802 1.285693 1.175977
C -0.857295 2.146423 1.822923
C -3.185634 2.099404 0.778461
H -2.230671 0.488080 1.876218
C -1.430048 2.934887 2.994362
H -0.434821 2.837107 1.076449
H -0.045703 1.501487 2.180710
C -3.721206 2.869339 1.978840
H -2.915414 2.807636 -0.018327
H -3.964390 1.442186 0.371207
C -2.646210 3.754459 2.590588
H -0.646180 3.578485 3.410825
H -1.712407 2.229560 3.789187
H -4.586862 3.464890 1.665927
H -4.084214 2.155055 2.731730
H -3.045596 4.290633 3.459673
H -2.345975 4.520022 1.859587
C -2.339450 -0.988998 -0.848930
C -3.147736 -1.620459 0.286177
C -3.213150 -0.593900 -2.043492
H -1.588520 -1.710796 -1.199000
C -3.911126 -2.827545 -0.243374
H -3.861285 -0.884390 0.684130
H -2.488502 -1.914296 1.110205
C -3.988524 -1.808268 -2.537852
H -3.915869 0.198549 -1.746880
H -2.579245 -0.191120 -2.842456
C -4.797971 -2.454246 -1.422434

H -4.505582 -3.263387 0.568278
H -3.187626 -3.595767 -0.552602
H -4.639003 -1.508933 -3.368368
H -3.275835 -2.540640 -2.944215
H -5.320064 -3.341552 -1.800227
H -5.575997 -1.753409 -1.083923
H 5.797595 -1.378180 2.566415
H 3.489175 4.586404 -2.396778
N 1.252614 2.197105 -1.657507
N 2.669670 -0.397651 1.852896
H -1.204813 1.358611 -1.330284

5a-Cy

E = -2493.46840441

P 1.436919 -0.377973 0.388572
C -0.311707 -0.201861 -0.142609
S -0.374265 0.426423 -1.865650
S -1.147019 -1.816067 0.084113
O 0.793147 -0.148392 -2.529459
C -0.106154 2.142795 -1.583120
O -1.724270 0.193973 -2.351274
H -0.826962 0.522984 0.498067
C 1.166617 2.673774 -1.767487
C -1.154618 2.907992 -1.074559
O -0.909808 -2.596608 -1.121258
O -0.597076 -2.238762 1.377616
C -2.851742 -1.467767 0.266271
H 1.958225 2.062954 -2.195723
C 1.396255 3.992469 -1.399110
C -0.902418 4.221602 -0.716381
H -2.146560 2.476443 -0.956981
C -3.332581 -1.122234 1.527506
C -3.685086 -1.561508 -0.844530
H 2.388978 4.417267 -1.532843
C 0.374816 4.781585 -0.860820
H -1.710549 4.827764 -0.311763
H -2.663203 -1.087187 2.384273
C -4.683310 -0.847008 1.664951
C -5.033163 -1.287291 -0.678550
H -3.274626 -1.833830 -1.812445
C 0.627276 6.203454 -0.472714
H -5.074952 -0.574676 2.642865
C -5.552793 -0.924826 0.569669
H -5.698181 -1.353898 -1.537228
H 1.695059 6.400545 -0.339565
H 0.101506 6.462109 0.452494
H 0.257795 6.880641 -1.254421
C -7.014933 -0.658147 0.736977
H -7.205034 0.038773 1.559235
H -7.544203 -1.592274 0.969815
H -7.455812 -0.254279 -0.179877
C 2.214751 -1.856404 -0.353250
C 3.484476 -1.446482 -1.109206
C 2.485743 -2.973317 0.656182
H 1.468473 -2.193264 -1.086787
C 4.087823 -2.661270 -1.801195
H 4.213529 -1.022925 -0.402072
H 3.242706 -0.669873 -1.843849
C 3.089936 -4.173100 -0.063444
H 3.193208 -2.614696 1.418403
H 1.561301 -3.256703 1.169103
C 4.353746 -3.794686 -0.821452
H 5.010734 -2.363794 -2.313341
H 3.391737 -3.004262 -2.580286
H 3.296648 -4.964070 0.667233
H 2.345551 -4.578335 -0.764155
H 4.754469 -4.666984 -1.351665
H 5.126821 -3.481347 -0.103733
C 1.621438 -0.213262 2.195472
C 3.029131 0.321452 2.510211
C 0.541600 0.677337 2.809753
H 1.504147 -1.240961 2.559451
C 3.193674 1.804597 2.192769

H 3.801798 -0.274500 2.005905
H 3.178768 0.157420 3.585586
C 0.703297 2.135455 2.402468
H -0.456135 0.282278 2.583022
H 0.650254 0.591352 3.899256
C 2.081051 2.652685 2.795230
H 3.222459 1.966455 1.104816
H 4.171007 2.137564 2.560707
H 0.566601 2.253125 1.312592
H -0.084566 2.739762 2.866321
H 2.164140 2.637783 3.891613
H 2.197404 3.698081 2.485343
H 2.029538 0.740571 -0.212552

5b-iPr

E = -2212.94085019

S 0.414124 -0.812104 -1.380003
O -0.734489 -1.156499 -2.214938
C -0.279033 0.120325 0.037472
C 1.009491 -2.277797 -0.548470
O 1.544411 -0.076121 -1.922784
P -2.074290 -0.200094 0.193220
H 0.244307 -0.200339 0.948206
S 0.051135 1.906696 -0.081847
C 2.276282 -2.760352 -0.821248
O -0.210534 2.366750 -1.436742
O -0.699728 2.467118 1.040822
C 1.800453 1.965577 0.269837
C 0.494820 -3.910251 0.925615
C 2.639280 -3.929057 -0.155713
H 2.938172 -2.243378 -1.510593
C 2.629543 2.735893 -0.524889
H -0.226807 -4.336058 1.621711
C 1.737928 -4.510566 0.725774
H 3.618252 -4.372257 -0.322039
C 3.440321 1.267639 1.651214
C 3.971239 2.759638 -0.155507
H 2.242791 3.272964 -1.386306
H 3.735085 0.665681 2.509904
C 4.380470 2.017384 0.945127
H 4.686939 3.344289 -0.728468
H 1.991695 -5.419561 1.264854
H 5.422104 2.008329 1.255436
N 2.155248 1.247765 1.315209
N 0.133324 -2.802756 0.287374
C -2.645435 0.201770 1.875095
C -1.588053 -0.165769 2.910128
C -3.991569 -0.482660 2.099164
H -2.774682 1.291202 1.865183
H -2.011171 -0.021372 3.910168
H -1.274495 -1.214342 2.827775
H -0.709841 0.483629 2.826387
H -4.735858 -0.196948 1.347034
H -3.894898 -1.574444 2.094323
H -4.380735 -0.180907 3.077401
C -2.982764 0.638353 -1.160636
C -3.592589 1.957465 -0.704222
C -3.984011 -0.327638 -1.785140
H -2.188518 0.836311 -1.891411
H -4.039291 2.448720 -1.575262
H -4.386792 1.805246 0.036098
H -2.841760 2.634248 -0.284878
H -4.771559 -0.622352 -1.081768
H -4.465345 0.170709 -2.633627
H -3.482058 -1.226176 -2.158852
H -2.140852 -1.590045 0.029477

5a-iPr

E = -2259.59258748

S -0.568847 -0.642422 -1.429766
O -1.434922 0.190890 -2.260947

C -0.104084 0.417085 0.002147
C -1.533740 -1.907274 -0.683845
O 0.669634 -1.205642 -1.941998
P -1.459371 1.604083 0.364669
H 0.058393 -0.232617 0.869609
S 1.435257 1.371321 -0.300234
C -2.920128 -1.848847 -0.779982
C -0.884246 -2.949186 -0.022717
O 1.394082 1.778979 -1.697320
O 1.374485 2.369505 0.773427
C 2.793346 0.304864 -0.008911
H -3.399230 -1.040768 -1.328122
C -3.668967 -2.853625 -0.182003
C -1.652669 -3.936362 0.569196
H 0.202554 -2.986524 0.016771
C 3.386113 -0.347271 -1.086873
C 3.285139 0.183773 1.289898
H -4.754277 -2.822179 -0.252268
C -3.052981 -3.906165 0.500725
H -1.159591 -4.755177 1.089429
H 2.981133 -0.226065 -2.087024
C 4.485689 -1.154394 -0.843189
C 4.382485 -0.633131 1.506759
H 2.826708 0.733368 2.108449
C -3.861163 -4.995710 1.130381
H 4.957849 -1.673069 -1.675109
C 4.999003 -1.313381 0.449252
H 4.776607 -0.738685 2.515511
H -4.926953 -4.750712 1.144788
H -3.530594 -5.187834 2.157512
H -3.736595 -5.933382 0.573293
C 6.204253 -2.165123 0.691542
H 6.269358 -2.983121 -0.032805
H 6.202426 -2.585075 1.702327
H 7.117811 -1.563637 0.588382
C -1.576261 2.903276 -0.928790
C -1.022642 4.241380 -0.455613
C -3.012832 2.978470 -1.436532
H -0.938315 2.505466 -1.727197
H 0.015506 4.159661 -0.122021
H -1.054465 4.936763 -1.301346
H -1.624438 4.675651 0.350884
H -3.057733 3.719282 -2.242073
H -3.339267 2.015875 -1.842562
H -3.711795 3.294350 -0.652976
C -1.404883 2.237144 2.073140
C -0.903584 1.185655 3.053194
C -2.793809 2.771032 2.417452
H -0.682704 3.061639 2.024662
H -0.968618 1.594290 4.067385
H -1.507540 0.270148 3.025915
H 0.145874 0.933786 2.870212
H -2.748839 3.261278 3.395960
H -3.152410 3.512730 1.694937
H -3.530928 1.962029 2.477076
H -2.592400 0.775784 0.283687

L-Ni(CO)$_3$ complexes

4a-Cy-Ni(CO)$_3$

E = -4335.93690083

P -0.021457 0.892531 0.157252
C 0.145719 -0.891720 -0.029075
C 1.752114 1.381741 0.449759
C -0.887658 1.150697 1.795363
S -0.568745 -1.740188 -1.340828
S 0.970126 -1.809947 1.173363
C 2.525373 1.289379 -0.863857
C 1.977481 2.735764 1.115543
C -1.428109 2.571152 1.963712
C -2.018995 0.137250 1.966084
O -0.649221 -0.882309 -2.532747

O 0.089577 -3.048591 -1.520221
C -2.264244 -2.100766 -0.898222
O 1.025137 -1.025071 2.426103
O 0.444009 -3.180794 1.302237
C 2.688473 -1.974928 0.677925
C 4.015684 1.514043 -0.641186
C 3.470436 2.975629 1.337377
C -2.116978 2.745937 3.313896
C -2.735965 0.312327 3.299699
C -3.296409 -1.568795 -1.659886
C -2.534179 -2.902483 0.213502
C 3.680404 -1.730514 1.623769
C 3.023065 -2.355924 -0.621148
C 4.268931 2.852800 0.044041
C -3.244183 1.735975 3.492482
C -4.618472 -1.819755 -1.290993
C -3.855143 -3.148489 0.563562
C 5.020082 -1.853379 1.258906
C 4.364146 -2.470350 -0.970234
C -4.920358 -2.606896 -0.176159
C 5.385425 -2.215859 -0.042302
C -6.340154 -2.874858 0.228840
H -6.574381 -3.949671 0.164706
H -6.519366 -2.570016 1.271918
H -7.051135 -2.334560 -0.411365
C 6.827008 -2.299020 -0.449017
H 7.128142 -1.393554 -1.001913
H 7.488696 -2.388822 0.423899
H 7.008689 -3.157180 -1.112917
H -4.071206 -3.768737 1.438318
H -1.701666 -3.298135 0.802013
H -3.053280 -0.951387 -2.525413
H -5.430924 -1.386573 -1.880733
H 5.798506 -1.655260 2.001006
H 3.393866 -1.429324 2.632873
H 2.226427 -2.555507 -1.342375
H 4.627098 -2.761410 -1.991120
H 2.134308 2.057057 -1.554152
H 2.342786 0.313109 -1.339712
H 4.412453 0.693713 -0.017313
H 4.554495 1.464917 -1.601941
H 3.973037 3.668960 -0.640492
H 5.344579 2.989597 0.244546
H 3.628797 3.966266 1.794898
H 3.844513 2.232046 2.064304
H 1.452224 2.784008 2.081690
H 1.564704 3.541368 0.484289
H -0.629005 3.318071 1.846728
H -2.153238 2.773258 1.157641
H -2.501644 3.774959 3.408826
H -1.375078 2.612931 4.122482
H -3.714248 1.849422 4.483498
H -4.032524 1.943095 2.745739
H -2.032428 0.064186 4.115078
H -3.566502 -0.409375 3.374154
H -2.741853 0.249301 1.136849
H -1.617344 -0.881017 1.887405
H -0.132199 0.947053 2.571412
H 2.143981 0.620510 1.142353
Ni -0.980513 2.154065 -1.462484
C -2.724198 1.807320 -1.430993
C -0.180668 1.753922 -3.019874
C -0.659040 3.844086 -1.020197
O -3.859377 1.639007 -1.390137
O 0.336844 1.565659 -4.023450
O -0.478555 4.952270 -0.773642

4b-Cy-Ni(CO)$_3$

E = -4289.42284556

P -0.057133 0.761806 0.162648
C 0.251169 -1.001643 -0.063360
C 1.675697 1.380413 0.460507
C -0.931807 0.906527 1.810771

S -0.420040 -1.872547 -1.381627
S 1.140594 -1.875340 1.126869
C 2.443413 1.360478 -0.860453
C 1.785287 2.747599 1.129254
C -1.589743 2.272229 2.012004
C -1.972008 -0.203366 1.963706
O -0.556328 -1.024650 -2.567173
O 0.281194 -3.159460 -1.561740
O 1.163858 -1.106637 2.381303
O 0.725865 -3.285943 1.232553
C 3.907101 1.732541 -0.658437
C 3.251099 3.129226 1.330563
C -2.286026 2.358773 3.366885
C -2.697659 -0.116998 3.301379
C 4.035541 3.090182 0.023529
C -3.323925 1.254121 3.524043
H 1.972265 2.084842 -1.547470
H 2.347954 0.371011 -1.336143
H 4.394584 0.960931 -0.037488
H 4.434914 1.734612 -1.626415
H 3.646289 3.871818 -0.654451
H 5.095182 3.334889 0.205248
H 3.319287 4.128808 1.790783
H 3.707301 2.422579 2.047080
H 1.271119 2.741932 2.102571
H 1.289289 3.512482 0.507703
H -0.855876 3.085387 1.910080
H -2.332088 2.431152 1.212114
H -2.755369 3.349490 3.485027
H -1.533183 2.270517 4.171363
H -3.799807 1.306182 4.517353
H -4.128463 1.409794 2.782057
H -1.973179 -0.321137 4.110335
H -3.463789 -0.907951 3.361718
H -2.704645 -0.134910 1.138174
H -1.486059 -1.182924 1.866231
H -0.156658 0.752194 2.578444
H 2.130365 0.651470 1.150911
Ni -1.125693 1.975327 -1.423549
C -2.849600 1.522824 -1.383359
C -0.328738 1.669810 -3.002030
C -0.929512 3.677139 -0.957789
O -3.978643 1.324430 -1.334020
O 0.180093 1.550292 -4.020665
O -0.834010 4.793690 -0.700403
C 2.874826 -1.940503 0.573952
C 3.170145 -2.441478 -0.694554
N 3.774424 -1.514445 1.441504
C 4.509828 -2.483112 -1.064764
H 2.360791 -2.770903 -1.351611
C 5.057239 -1.563113 1.077877
C 5.476594 -2.035273 -0.163446
H 4.797484 -2.859164 -2.049520
H 5.787249 -1.202374 1.810704
H 6.538169 -2.047901 -0.417958
C -2.098628 -2.336782 -0.879839
C -2.243987 -3.282946 0.137445
N -3.097173 -1.725228 -1.486159
C -3.538790 -3.589181 0.538115
H -1.356948 -3.716882 0.605294
C -4.336068 -2.020815 -1.089727
C -4.610022 -2.944003 -0.083670
H -3.713015 -4.317947 1.333464
H -5.149400 -1.489197 -1.595216
H -5.641774 -3.150817 0.207538

4a-iPr-Ni(CO)$_3$

E = -4102.63193394

P -0.077265 1.188491 0.610406
C 0.358667 -0.524240 0.237244
S -0.331594 -1.340752 -1.109541
S 1.504706 -1.381803 1.202693
O -0.673858 -0.389998 -2.178923

O 0.506433 -2.491378 -1.502822
C -1.882673 -2.068227 -0.590041
O 1.656994 -0.677539 2.495365
O 1.202072 -2.822307 1.277833
C 3.137761 -1.266948 0.460306
C -3.011468 -1.906064 -1.382922
C -1.928519 -2.846472 0.568913
C 4.160994 -0.675061 1.193666
C 3.382159 -1.775568 -0.816769
C -4.209058 -2.509823 -0.998750
C -3.127535 -3.443124 0.936354
C 5.436011 -0.574775 0.637256
C 4.658019 -1.667151 -1.356487
C -4.289647 -3.282730 0.163114
C 5.706864 -1.063900 -0.644378
C -5.579469 -3.921232 0.587543
H -5.458857 -5.007014 0.726932
H -5.925072 -3.511349 1.550200
H -6.373802 -3.757701 -0.153800
C 7.072335 -0.943695 -1.254329
H 7.051862 -0.289776 -2.141065
H 7.797378 -0.525065 -0.542725
H 7.446316 -1.924023 -1.588583
H -3.168319 -4.047305 1.847248
H -1.022629 -2.965348 1.170182
H -2.948881 -1.293967 -2.283528
H -5.101362 -2.369956 -1.614982
H 6.237348 -0.105063 1.214463
H 3.947725 -0.291402 2.192416
H 2.557828 -2.220173 -1.379884
H 4.847167 -2.057527 -2.360657
Ni -1.480516 2.362637 -0.719401
C -3.034019 1.508741 -0.900987
C -0.642121 2.509689 -2.291977
C -1.739520 3.920310 0.092181
O -4.072687 1.041397 -1.036217
O -0.120650 2.602837 -3.307914
O -1.947094 4.939909 0.581336
C 1.596943 2.027935 0.695502
C 1.530201 3.523338 0.981158
C 2.335443 1.805549 -0.622497
H 2.136915 1.517639 1.509820
H 1.030997 3.771286 1.924990
H 2.551903 3.934878 1.025829
H 0.997311 4.043403 0.170690
H 2.274296 0.766237 -0.969219
H 1.919817 2.448671 -1.412325
H 3.400216 2.063254 -0.505537
C -0.772113 1.064809 2.348735
C -2.179424 0.488563 2.255207
C -0.762677 2.350613 3.161416
H -0.117069 0.334114 2.840838
H -2.216836 -0.406682 1.617412
H -2.536891 0.201808 3.257907
H -2.882585 1.228119 1.842078
H 0.255315 2.671284 3.422523
H -1.266686 3.179779 2.641015
H -1.302376 2.184962 4.108457

4a-Ph-Ni(CO)$_3$

E = -4328.69107547

P 0.133490 1.039533 0.076815
C -0.209278 -0.682682 -0.199079
S -1.174121 -1.232132 -1.514863
S 0.349418 -1.860504 0.926102
O -1.414845 -0.103861 -2.428368
O -0.638293 -2.472205 -2.102508
C -2.784045 -1.645238 -0.855869
O 0.383256 -1.293535 2.286172
O -0.394670 -3.119089 0.749150
C 2.050833 -2.210549 0.506513
C -3.697395 -0.617732 -0.628131
C -3.112086 -2.967672 -0.568620

C 3.016594 -2.200555 1.505143
C 2.383198 -2.509693 -0.814523
C -4.943262 -0.921529 -0.085784
C -4.362759 -3.255630 -0.031418
C 4.340538 -2.478266 1.172059
C 3.709001 -2.782388 -1.130051
C -5.295476 -2.240928 0.226500
C 4.710298 -2.764446 -0.146280
C -6.628423 -2.565678 0.834406
H -7.106431 -3.414325 0.321831
H -6.517061 -2.851400 1.893317
H -7.312269 -1.706676 0.790668
C 6.145999 -3.004482 -0.510538
H 6.617084 -2.073318 -0.868441
H 6.728894 -3.355466 0.352878
H 6.239715 -3.745189 -1.318131
H -4.622226 -4.293552 0.195979
H -2.374298 -3.748051 -0.753967
H -3.434674 0.412451 -0.876222
H -5.659838 -0.115851 0.095667
H 5.106057 -2.455855 1.952611
H 2.726619 -1.945804 2.525341
H 1.602085 -2.503910 -1.580479
H 3.978308 -3.008553 -2.165697
Ni 0.063014 2.528789 -1.597647
C -1.690832 2.822999 -1.774078
C 0.796784 1.694711 -2.999648
C 1.029808 3.893281 -0.991818
O -2.829624 2.947343 -1.819597
O 1.289546 1.126902 -3.863862
O 1.668977 4.753544 -0.579288
C 1.833580 1.053623 0.771316
C 2.897745 0.892404 -0.126651
C 2.110711 1.237128 2.128247
C 4.213497 0.911847 0.323931
H 2.689655 0.740137 -1.188284
C 3.431524 1.275384 2.576414
H 1.289335 1.336269 2.839385
C 4.484736 1.112558 1.678498
H 5.031616 0.770854 -0.386571
H 3.636239 1.423746 3.639835
H 5.518003 1.135567 2.033821
C -0.934910 1.589725 1.464149
C -0.843870 2.911585 1.922622
C -1.913782 0.750989 2.002752
C -1.703131 3.377130 2.915012
H -0.090836 3.583114 1.502286
C -2.788129 1.223083 2.981500
H -1.982700 -0.279458 1.651497
C -2.683328 2.534284 3.443481
H -1.614447 4.406819 3.270511
H -3.553617 0.556619 3.387148
H -3.366295 2.902552 4.213156

3a-Cy-Ni(CO)$_3$

E = -4336.39050945

P -1.685658 0.085507 0.241932
C 0.210166 0.239414 0.117136
S 0.953425 -0.223303 -1.505224
S 0.917031 1.819298 0.742501
O 0.154613 -1.296535 -2.088364
C 2.510024 -0.957663 -1.078300
O 1.183894 1.004936 -2.262249
H 0.584651 -0.528496 0.811606
C 3.667684 -0.449052 -1.654468
C 2.535091 -2.105535 -0.283759
O 0.543010 2.912744 -0.149929
O 0.529244 1.877513 2.156168
C 2.673467 1.645189 0.696900
H 3.613217 0.433100 -2.293508
C 4.880670 -1.077113 -1.389053
C 3.757567 -2.706275 -0.015072
H 1.613010 -2.535799 0.115209

C 3.400913 2.453566 -0.169895
C 3.303141 0.770131 1.584960
H 5.795380 -0.679302 -1.835220
C 4.950253 -2.197575 -0.553670
H 3.788331 -3.596762 0.617533
H 2.875941 3.128248 -0.847248
C 4.790567 2.370251 -0.153727
C 4.686820 0.686160 1.569380
H 2.721215 0.162757 2.281184
C 6.268109 -2.825212 -0.216250
H 5.372400 3.001234 -0.829776
C 5.452580 1.483005 0.701919
H 5.189101 -0.007928 2.247640
H 7.035543 -2.580083 -0.963236
H 6.186999 -3.919025 -0.141134
H 6.626366 -2.458511 0.760362
C 6.944746 1.359334 0.690875
H 7.244410 0.349066 0.368533
H 7.361988 1.510657 1.698191
H 7.407945 2.086155 0.010665
C -2.288655 1.775423 -0.323581
C -3.590060 1.683446 -1.124121
C -2.430072 2.846709 0.761500
H -1.492126 2.093259 -1.018674
C -3.948137 3.032369 -1.740022
H -4.410488 1.350649 -0.463584
H -3.501174 0.925483 -1.913063
C -2.773421 4.198885 0.144062
H -3.237238 2.552886 1.455413
H -1.513737 2.941781 1.356314
C -4.045891 4.130090 -0.689127
H -4.893041 2.944049 -2.300116
H -3.170768 3.301703 -2.477533
H -2.872185 4.953334 0.941351
H -1.927964 4.519181 -0.489673
H -4.250424 5.102628 -1.165494
H -4.903600 3.920506 -0.024379
C -1.897468 0.113080 2.110223
C -3.352348 -0.217221 2.485913
C -0.922025 -0.707444 2.962372
H -1.707886 1.162058 2.362049
C -3.643157 -1.708334 2.632145
H -4.055393 0.237444 1.767834
H -3.550693 0.282536 3.450614
C -1.229648 -2.193708 3.049476
H 0.119976 -0.523947 2.667614
H -0.990794 -0.283749 3.980266
C -2.652020 -2.411532 3.547874
H -3.583871 -2.184794 1.642882
H -4.676698 -1.844414 2.988380
H -1.107613 -2.671319 2.063568
H -0.502713 -2.678714 3.720569
H -2.739952 -2.016813 4.576708
H -2.884920 -3.486958 3.602624
Ni -2.655908 -1.591023 -0.917967
C -1.766587 -3.081271 -0.491745
C -4.401348 -1.549543 -0.536582
C -2.480672 -1.265596 -2.684564
O -1.147442 -4.013801 -0.246605
O -5.534219 -1.539055 -0.365106
O -2.416842 -1.100281 -3.812862

3b-Cy-Ni(CO)$_3$

E = -4289.86643226

P -1.353281 0.115543 0.237943
C 0.549506 0.183096 0.126512
S 1.285621 -0.315518 -1.486816
S 1.320340 1.733944 0.744917
O 0.465867 -1.372942 -2.071093
O 1.590487 0.889448 -2.240956
H 0.882113 -0.595747 0.830702
O 1.015080 2.841384 -0.147230
O 0.949774 1.806721 2.163343

C -1.874690 1.834293 -0.320269
C -3.180575 1.805814 -1.118649
C -1.965114 2.906177 0.769735
H -1.065384 2.116615 -1.016081
C -3.478151 3.172625 -1.727209
H -4.013860 1.507269 -0.457852
H -3.127569 1.048787 -1.911615
C -2.246730 4.275332 0.158557
H -2.784514 2.646631 1.462928
H -1.045486 2.957066 1.364983
C -3.523319 4.268586 -0.670947
H -4.427429 3.129616 -2.285133
H -2.691104 3.409732 -2.465377
H -2.308068 5.030054 0.959238
H -1.389263 4.558521 -0.476549
H -3.684327 5.251565 -1.142414
H -4.387946 4.095232 -0.004706
C -1.570701 0.141851 2.105623
C -3.040202 -0.129458 2.471709
C -0.636648 -0.725906 2.957015
H -1.337630 1.180404 2.365174
C -3.393949 -1.608720 2.599263
H -3.720067 0.362834 1.756149
H -3.221258 0.367586 3.441123
C -1.004783 -2.199442 3.025538
H 0.414728 -0.581294 2.673332
H -0.697224 -0.310239 3.978685
C -2.438526 -2.363471 3.512077
H -3.349696 -2.076155 1.604879
H -4.434115 -1.705233 2.948881
H -0.895104 -2.671650 2.035389
H -0.303448 -2.720665 3.696691
H -2.516651 -1.977658 4.545021
H -2.716292 -3.428781 3.552455
Ni -2.380618 -1.504898 -0.946957
C -1.560883 -3.037933 -0.533144
C -4.126788 -1.392245 -0.579949
C -2.170520 -1.165670 -2.707592
O -0.979855 -3.996414 -0.294302
O -5.258824 -1.329981 -0.416328
O -2.083840 -0.989638 -3.832484
C 3.104307 1.465201 0.730180
C 3.658353 0.587835 1.659775
N 3.777172 2.207081 -0.121835
C 5.046548 0.480752 1.664631
H 3.038086 0.026372 2.360185
C 5.103698 2.108575 -0.101346
C 5.782411 1.258023 0.772495
H 5.545337 -0.193235 2.363900
H 5.654214 2.733191 -0.811556
H 6.872543 1.208171 0.751044
C 2.813755 -1.164680 -1.017419
C 2.713951 -2.362216 -0.311602
N 3.927784 -0.639196 -1.479777
C 3.905781 -3.028837 -0.040738
H 1.749308 -2.769909 -0.002731
C 5.060353 -1.296513 -1.235723
C 5.100550 -2.487891 -0.511217
H 3.897400 -3.967447 0.517236
H 5.979261 -0.852085 -1.630139
H 6.054574 -2.985241 -0.327950

3a-iPr-Ni(CO)$_3$

E = -4103.10522943

P 2.163073 -0.568359 -0.116445
C 0.263808 -0.462830 0.105289
S -0.506610 0.330068 -1.350008
S -0.625097 -1.967915 0.690819
O 0.429213 1.355325 -1.815418
C -1.940280 1.175021 -0.747507
O -0.921692 -0.732571 -2.266171
H 0.120329 0.237359 0.941002
C -3.144312 0.968006 -1.412493

C -1.826870 2.112983 0.280036
O -0.281516 -3.107284 -0.157751
O -0.324490 -2.015866 2.124203
C -2.356074 -1.662205 0.535519
H -3.198790 0.241654 -2.224223
C -4.263914 1.689034 -1.011489
C -2.961721 2.806727 0.678680
H -0.872108 2.305279 0.774098
C -3.062203 -2.256314 -0.506119
C -2.996241 -0.896928 1.513488
H -5.213517 1.528205 -1.527645
C -4.198630 2.601511 0.047835
H -2.885541 3.529770 1.494279
H -2.534682 -2.855602 -1.248543
C -4.438241 -2.060996 -0.574959
C -4.365996 -0.703907 1.418029
H -2.426810 -0.466277 2.338801
C -5.426583 3.316474 0.521452
H -5.003969 -2.521891 -1.388111
C -5.108073 -1.279270 0.373914
H -4.875467 -0.096503 2.170182
H -6.170097 3.416732 -0.281300
H -5.188412 4.316938 0.909099
H -5.901032 2.752415 1.342147
C -6.583180 -1.039924 0.281252
H -6.791821 0.034291 0.151788
H -7.092864 -1.355599 1.204553
H -7.030859 -1.580172 -0.563284
Ni 3.073277 1.404070 0.394933
C 2.040695 2.020970 1.704016
C 4.769117 1.114586 0.874106
C 2.983511 2.442479 -1.069284
O 1.304863 2.375622 2.509612
O 5.858656 0.949338 1.184931
O 2.948964 3.121308 -1.986713
C 2.646241 -1.933688 1.059679
C 2.623386 -1.411430 2.492971
C 4.032110 -2.474610 0.718871
H 1.915810 -2.748352 0.945668
H 1.657843 -0.967301 2.764254
H 2.804324 -2.247683 3.186311
H 3.414056 -0.662614 2.646108
H 4.058366 -3.005079 -0.241285
H 4.792174 -1.678898 0.698291
H 4.331046 -3.190750 1.499939
C 2.423401 -1.156824 -1.867274
C 1.943495 -2.558210 -2.210099
C 3.873692 -0.914624 -2.288008
H 1.814973 -0.431108 -2.429377
H 0.872308 -2.677607 -2.017059
H 2.129717 -2.747066 -3.279450
H 2.478675 -3.332314 -1.640323
H 4.213925 0.095841 -2.021862
H 4.567679 -1.636748 -1.836572
H 3.949370 -1.023119 -3.380886

3a-Ph-Ni(CO)$_3$

E = -4329.15010326

P 1.773279 -0.019568 0.025036
C -0.092256 -0.289014 -0.052195
S -0.869112 0.439265 -1.560727
S -0.673226 -2.003510 0.269087
O 0.002886 1.517476 -2.019019
C -2.361728 1.168901 -0.941363
O -1.217858 -0.643411 -2.474255
H -0.500887 0.297229 0.784704
C -3.574451 0.745454 -1.472212
C -2.299449 2.196783 0.001782
O -0.385055 -2.862171 -0.871953
O -0.130229 -2.342845 1.585788
C -2.425429 -1.829770 0.421117
H -3.590055 -0.043656 -2.224632
C -4.749948 1.334793 -1.016709

C -3.484249 2.760783 0.455227
H -1.342566 2.561355 0.381164
C -3.243650 -2.419713 -0.536378
C -2.956513 -1.139888 1.513215
H -5.706469 1.002505 -1.427453
C -4.728098 2.332927 -0.036534
H -3.444586 3.557587 1.202001
H -2.794198 -2.950321 -1.376842
C -4.624113 -2.299822 -0.401528
C -4.333686 -1.012744 1.616965
H -2.302439 -0.714393 2.277275
C -5.999934 2.915468 0.499463
H -5.276703 -2.758464 -1.148300
C -5.188919 -1.586857 0.662077
H -4.759569 -0.462171 2.459403
H -6.841270 2.750464 -0.187312
H -5.902364 3.995044 0.683455
H -6.259014 2.444926 1.462683
C -6.671491 -1.412367 0.779897
H -6.945685 -0.354276 0.637905
H -7.027669 -1.704316 1.779397
H -7.211265 -2.006697 0.030865
Ni 2.589086 2.038710 -0.216735
C 1.523899 3.069591 0.768436
C 4.231791 1.830987 0.468618
C 2.627375 2.562596 -1.938913
O 0.805966 3.705027 1.396292
O 5.276932 1.689901 0.909783
O 2.663612 2.916607 -3.023838
C 2.154457 -0.451321 1.762076
C 1.376106 0.061809 2.807636
C 3.354581 -1.102817 2.067287
C 1.766803 -0.113216 4.130790
H 0.455798 0.614538 2.604898
C 3.745147 -1.274485 3.395237
H 3.998075 -1.477963 1.269124
C 2.951204 -0.788026 4.430655
H 1.142084 0.284193 4.933959
H 4.681573 -1.791843 3.616307
H 3.256810 -0.925726 5.470257
C 2.455452 -1.357819 -1.004085
C 2.536062 -2.690051 -0.575743
C 2.918058 -1.012084 -2.278842
C 3.085681 -3.656102 -1.412250
H 2.161980 -2.974430 0.409292
C 3.449111 -1.986905 -3.121245
H 2.856419 0.024397 -2.615574
C 3.539612 -3.307543 -2.685301
H 3.150425 -4.691709 -1.071003
H 3.800813 -1.709602 -4.117371
H 3.966006 -4.071061 -3.340375

PMe_3-Ni(CO)$_3$

E = -2307.98865272

P 1.407455 -0.000076 0.000551
Ni -0.802178 0.000319 -0.000697
C -1.262279 1.592928 -0.653988
C -1.262217 -1.362142 -1.052837
C -1.264066 -0.230118 1.704819
O -1.515092 2.627102 -1.077543
O -1.515474 -2.246990 -1.735283
O -1.518402 -0.379807 2.811939
C 2.189116 -1.523729 0.640109
H 1.859322 -2.379922 0.033860
H 3.287796 -1.459160 0.612995
H 1.859543 -1.690234 1.675930
C 2.192295 0.207763 -1.637399
H 3.290934 0.199699 -1.565910
H 1.864396 -0.606313 -2.300095
H 1.862785 1.160513 -2.076629
C 2.190937 1.314438 1.000623
H 1.858783 1.219906 2.044655
H 3.289606 1.255196 0.960631

H 1.865036 2.295234 0.624938

PNMe$_2$-Ni(CO)$_3$

E = -2592.62079671

P 0.745588 -0.000110 0.087592
Ni -1.470313 0.000157 -0.041994
C -1.863789 1.485367 -0.953961
C -1.863929 -1.483741 -0.956105
C -2.222090 -0.001227 1.577879
O -2.119392 2.440530 -1.531145
O -2.119601 -2.438106 -1.534569
O -2.706178 -0.002231 2.615659
N 1.423560 -1.404129 0.758338
N 1.423667 1.402810 0.760581
N 1.570990 0.001036 -1.424819
C 0.673952 -2.187334 1.709426
H -0.399056 -1.985340 1.595383
H 0.840065 -3.264421 1.532341
H 0.959215 -1.971066 2.757169
C 2.846381 -1.656903 0.770156
H 3.336478 -1.106412 -0.042736
H 3.317487 -1.366925 1.728524
H 3.038652 -2.732890 0.614501
C 1.446697 -1.198202 -2.226453
H 1.500551 -2.091944 -1.592434
H 0.492328 -1.232431 -2.789676
H 2.268940 -1.241240 -2.959431
C 1.446944 1.201554 -2.224552
H 0.492464 1.237020 -2.787514
H 1.501282 2.094299 -1.589173
H 2.269034 1.245402 -2.957650
C 2.846491 1.655462 0.772846
H 3.317474 1.364358 1.730938
H 3.336657 1.105872 -0.040611
H 3.038893 2.731612 0.618437
C 0.674008 2.184887 1.712536
H -0.398998 1.983089 1.598136
H 0.959160 1.967335 2.760045
H 0.840184 3.262189 1.536789

PPh$_3$-Ni(CO)$_3$

E = -2883.60354624

P -0.071384 -0.000040 0.000362
Ni 2.146303 -0.003244 -0.008896
C 2.619083 -1.713466 -0.229857
C 2.618124 1.040897 -1.381649
C 2.629788 0.661060 1.579271
O 2.891056 -2.816648 -0.362853
O 2.889427 1.706563 -2.271559
O 2.908689 1.097768 2.599148
C -0.839689 1.348489 0.968006
C -1.985476 1.164262 1.750158
C -0.247760 2.617008 0.902880
C -2.535130 2.238136 2.450352
H -2.450112 0.177704 1.814711
C -0.805180 3.689552 1.594079
H 0.658799 2.759440 0.308476
C -1.949420 3.500962 2.370870
H -3.427476 2.085071 3.061953
H -0.338612 4.675593 1.534016
H -2.381873 4.340339 2.920572
C -0.845548 -1.507735 0.687980
C -1.997676 -2.085698 0.142822
C -0.251402 -2.089991 1.815701
C -2.551290 -3.226058 0.724769
H -2.464283 -1.644933 -0.741119
C -0.812635 -3.221953 2.400939
H 0.659907 -1.652285 2.232055
C -1.963180 -3.793037 1.854716
H -3.448611 -3.673436 0.290681

H -0.344222 -3.666429 3.282186
H -2.398700 -4.686511 2.308336
C -0.852846 0.162904 -1.645237
C -2.001807 0.930491 -1.867277
C -0.268230 -0.526169 -2.716298
C -2.561837 0.999507 -3.142891
H -2.460923 1.478442 -1.041260
C -0.835931 -0.464205 -3.986222
H 0.640682 -1.110716 -2.550074
C -1.983318 0.300829 -4.201773
H -3.456651 1.604143 -3.308681
H -0.374990 -1.008016 -4.814093
H -2.423887 0.356856 -5.200026

P(*p*-$C_6H_4OMe)_3$-Ni(CO)$_3$

E = -3226.95539097

P -0.068740 -0.001157 0.400060
Ni -0.284403 0.210502 2.601439
C -1.698808 -0.792013 3.033207
C 1.250181 -0.405744 3.278402
C -0.538932 1.955770 2.885207
O -2.613953 -1.436237 3.272889
O 2.241110 -0.817417 3.676467
O -0.690740 3.081033 3.029864
C -1.636910 -0.202720 -0.509540
C -2.756455 0.514284 -0.076915
C -1.767559 -1.032308 -1.633710
C -3.974525 0.432349 -0.747353
H -2.678306 1.151363 0.808348
C -2.976948 -1.130531 -2.305457
H -0.910877 -1.612485 -1.985072
C -4.091827 -0.396488 -1.871594
H -4.825144 1.007466 -0.381479
H -3.087913 -1.777820 -3.177731
C 0.920215 -1.437996 -0.133564
C 1.822824 -1.391331 -1.198003
C 0.758221 -2.652380 0.554017
C 2.544400 -2.521252 -1.584595
H 1.975048 -0.456090 -1.742086
C 1.459921 -3.784104 0.174323
H 0.070765 -2.704782 1.402634
C 2.362957 -3.729437 -0.900551
H 3.243964 -2.446185 -2.417271
H 1.336906 -4.731265 0.703244
C 0.735771 1.407883 -0.433115
C 1.785735 2.058496 0.221923
C 0.361271 1.859348 -1.707809
C 2.466687 3.118387 -0.372142
H 2.079156 1.732530 1.223587
C 1.024507 2.919589 -2.307473
H -0.464009 1.376585 -2.236671
C 2.086584 3.557509 -1.647836
H 3.281089 3.599638 0.169459
H 0.735551 3.279715 -3.296913
O 2.670876 4.573687 -2.304052
O -5.221153 -0.552930 -2.582017
O 3.006696 -4.871486 -1.194113
C -6.374527 0.151514 -2.192472
H -6.697028 -0.127516 -1.175210
H -6.217597 1.242591 -2.232483
H -7.163101 -0.121634 -2.903981
C 3.927873 -4.880211 -2.256642
H 3.444764 -4.627549 -3.215592
H 4.760345 -4.178739 -2.078708
H 4.327247 -5.899850 -2.316323
C 3.737591 5.256609 -1.692444
H 4.588132 4.584160 -1.489656
H 4.057292 6.033134 -2.397784
H 3.425929 5.735495 -0.748987

$PtBu_3$-Ni(CO)$_3$

E = -2662.34773436

P 0.601447 0.000075 -0.000131
Ni -1.679262 -0.000194 0.000205
C -2.221340 1.652752 0.392464
C -2.221564 -0.487045 -1.627314
C -2.220627 -1.166427 1.235777
O -2.599945 2.703706 0.650238
O -2.600444 -0.789366 -2.666240
O -2.598576 -1.915213 2.017306
C 1.289373 0.161704 1.786311
C 2.761093 0.573000 1.883696
C 0.428907 1.188218 2.536611
C 1.108797 -1.161697 2.539486
H 3.432477 -0.119069 1.360902
H 2.944580 1.584574 1.500696
H 3.054289 0.574802 2.946738
H 0.455970 2.190202 2.095025
H -0.619898 0.865034 2.581491
H 0.804819 1.270286 3.569772
H 1.773328 -1.958108 2.183217
H 1.353254 -0.986271 3.599767
H 0.072924 -1.524581 2.501598
C 1.289323 -1.627886 -0.753615
C 1.108797 -1.618725 -2.276317
C 2.761053 -1.917573 -0.445935
C 0.428759 -2.790808 -0.239711
H 1.353459 -2.624721 -2.654250
H 0.072786 -1.404968 -2.571486
H 1.773170 -0.912053 -2.788270
H 2.944442 -2.090844 0.621746
H 3.054330 -2.839455 -0.975215
H 3.432379 -1.118933 -0.784464
H 0.805104 -3.726749 -0.684525
H 0.454989 -2.908761 0.848912
H -0.619871 -2.668401 -0.542783
C 1.288676 1.466680 -1.033419
C 0.427538 1.602973 -2.297045
C 1.108276 2.780644 -0.263803
C 2.760269 1.345405 -1.438734
H 0.802447 2.457101 -2.884733
H 0.454758 0.719691 -2.944175
H -0.621237 1.802578 -2.038802
H 1.773041 2.870477 0.603878
H 1.352605 3.611113 -0.945979
H 0.072438 2.929337 0.069524
H 2.943498 0.507832 -2.123156
H 3.053344 2.264965 -1.972162
H 3.431979 1.238926 -0.578182

P(*p*-tolyl)-Ni(CO)$_3$

E = -3001.46003033

P 0.000097 -0.000943 0.202264
Ni 0.001725 0.000087 2.421915
C -0.908904 1.464706 2.891113
C -0.809670 -1.520745 2.894208
C 1.725171 0.056739 2.891785
O -1.488635 2.414928 3.156442
O -1.341047 -2.498111 3.162192
O 2.837745 0.083522 3.158045
C 0.005033 1.655080 -0.568434
C 0.793558 2.654711 0.017626
C -0.727434 1.962933 -1.718681
C 0.859223 3.923413 -0.546009
H 1.359422 2.432092 0.926452
C -0.664522 3.241062 -2.272059
H -1.353187 1.200429 -2.188495
C 0.128035 4.242167 -1.699899
H 1.484816 4.688175 -0.077342
H -1.245230 3.464337 -3.171284
C -1.437736 -0.823835 -0.566925
C -1.340952 -1.604862 -1.723132
C -2.695492 -0.645256 0.023558
C -2.479985 -2.186129 -2.276848
H -0.369021 -1.760941 -2.197243

C -3.828965 -1.219673 -0.541073
H -2.783735 -0.048952 0.935870
C -3.742305 -2.005073 -1.699247
H -2.385300 -2.793847 -3.181048
H -4.802881 -1.062455 -0.069383
C 1.430886 -0.833791 -0.569382
C 1.903214 -2.015606 0.016703
C 2.061348 -0.354753 -1.722032
C 2.968071 -2.707856 -0.548772
H 1.429061 -2.393854 0.926565
C 3.135233 -1.048671 -2.276737
H 1.712302 0.567479 -2.192572
C 3.607577 -2.235152 -1.703777
H 3.317591 -3.632128 -0.080421
H 3.616369 -0.658940 -3.178084
C -4.961373 -2.657951 -2.279734
H -5.159346 -3.622801 -1.783622
H -5.856052 -2.033558 -2.142691
H -4.838089 -2.860627 -3.352967
C 0.178997 5.623906 -2.280954
H -0.237357 5.650399 -3.297428
H -0.401809 6.328983 -1.663742
H 1.210847 6.004172 -2.318094
C 4.782204 -2.964399 -2.284958
H 4.930636 -2.711293 -3.344073
H 5.708895 -2.699318 -1.749153
H 4.660558 -4.054146 -2.200283

P(*p*-tolyl)-Ni(CO)$_3$

E = -3001.45177242

P -0.021077 -0.059763 -0.043378
Ni 0.811799 2.016176 -0.398260
C -0.454294 3.170246 -0.910278
C 1.491775 2.488747 1.187021
C 2.031813 1.732819 -1.676596
O -1.234175 3.944998 -1.228917
O 1.901042 2.762781 2.219581
O 2.803365 1.509253 -2.491138
C -1.546936 -0.432272 -1.007175
C -1.746893 0.382649 -2.129682
C -2.474172 -1.463691 -0.723954
C -2.850548 0.226171 -2.961962
H -1.006540 1.153587 -2.351838
C -3.588152 -1.589720 -1.565482
C -3.786661 -0.760146 -2.665989
H -2.976641 0.875563 -3.830983
H -4.314664 -2.377939 -1.350561
C -0.432559 -0.451334 1.704953
C 0.217415 -1.492079 2.380702
C -1.399639 0.322853 2.385435
C -0.061280 -1.771967 3.714805
H 0.939572 -2.114102 1.848099
C -1.666057 0.021684 3.726922
C -1.008350 -1.008676 4.393289
H 0.458122 -2.590466 4.218223
H -2.414869 0.616688 4.256646
C 1.143720 -1.388407 -0.567270
C 2.473582 -1.438304 -0.085054
C 0.728132 -2.321098 -1.528542
C 3.316894 -2.444486 -0.573333
C 1.590102 -3.303051 -2.008959
H -0.291252 -2.277090 -1.915470
C 2.892951 -3.368024 -1.524527
H 4.340759 -2.495000 -0.193005
H 1.237139 -4.015950 -2.757499
H 3.581752 -4.135476 -1.885407
H -1.238999 -1.215938 5.440930
H -4.668280 -0.895228 -3.297190
C -2.165853 1.419176 1.710969
H -1.484592 2.107300 1.189766
H -2.851723 1.018059 0.947503
H -2.759147 1.991198 2.436477
C -2.324731 -2.444741 0.402181

H -1.301927 -2.840328 0.465969
H -2.549238 -1.987056 1.377484
H -3.011586 -3.290004 0.259529
C 3.040612 -0.458298 0.901602
H 3.402969 0.445005 0.387159
H 2.311990 -0.132940 1.654396
H 3.895849 -0.900939 1.431041

5. References

[1] W. Voskuil, J. F. Arens, *Recl. Trav. Chim. Pays-Bas* **1963**, *82*, 302.
[2] Y. Brussaard, F. Olbrich, E. Schaumann, *Inorg. Chem.* **2013**, *52*, 13160.
[3] K. Ajiki, M. Hirano, K. Tanaka, *Org. Lett.* **2005**, *7*, 4193.
[4] A. Amoeda-Portela, R. Caballo, J. S. Casas, E. García-Martínez, C. Gómez-Alsonso, A. Sánchez-González, J. Sordo, E. M. Vázquez-López, *Z. Anorg. Allg. Chem.* **2002**, *628*, 939.
[5] G. M. Sheldrick, *Acta Crystallogr.* **2008**, *A64*, 112.
[6] G. M. Sheldrick, *Acta Cryst.* **2015**, *C71*, 3.
[7] A. Thorn, B. Dittrich, G. M. Sheldrick, *Acta Cryst.* **2012**, *A68*, 448.
[8] G. M Sheldrick, *Acta Cryst*. **2015***, A71*, 3.
[9] M. J. Frisch, G. W. Trucks, H. B. Schlegel, G. E. Scuseria, M. A. Robb, J. R. Cheeseman, G. Scalmani, V. Barone, G. A. Petersson, H. Nakatsuji et al., Gaussian 16, Revision C.01, Gaussian, Inc., Wallingford CT, 2016.
[10] a) P. Hohenberg, W. Kohn, *Phys. Rev.* **1964**, *136*, B864-B871; b) W. Kohn, L. J. Sham, *Phys. Rev*. **1965**, *140*, A1133-A1138.
[11] Y. Zhao, D. G. Truhlar, *J. Phys. Chem. A* **2005**, *109*, 5656.
[12] F. Weigend, R. Ahlrichs, *Phys. Chem. Chem. Phys*. **2005**, *7*, 3297.
[13] a) NBO 7.0. E. D. Glendening, J, K. Badenhoop, A. E. Reed, J. E. Carpenter, J. A. Bohmann, C. M. Morales, P. Karafiloglou, C. R. Landis, and F. Weinhold, Theoretical Chemistry Institute, University of Wisconsin, Madison (2018). b) L. Suidan, J. K. Badenhoop, E. D. Glendening, and F. Weinhold, *J. Chem. Educ.* **1995**, *72*, 583.
[14] a) A. D. Becke, *J. Chem. Phys.* **1993**, *98*, 5648; b) C. Lee, W. Yang, R. G. Parr, *Phys. Rev. B* **1988**, *37*, 785; c) S. H. Vosko, L. Wilk, M. Nusair, *Can. J. Phys.* **1980**, *58*, 1200; d) P. J. Stephens, F. J. Devlin, C. F. Chabalowski, M. J. Frisch, *J. Phys. Chem*. **1994**, *98*, 11623.
[15] a) A. D. McLean, G. S. Chandler, *J. Chem. Phys.***1980**, *72*, 5639; b) R. Krishnan, J. S. Binkley, R. Seeger, J. A. Pople, *J. Chem. Phys.***1980**, *72*, 650.
[16] a) S. Grimme, J. Antony, S. Ehrlich, H. Krieg, *J. Chem. Phys*. **2010**, *132*, 154104; b) S. Grimme, S. Ehrlich, L. Goerigk, *J. Comput. Chem*. **2011**, *32*, 1456.
[17] L. E. Chirlian, M. M. Francl, *J. Comp. Chem*. **1987**, *8*, 894.
[18] T. Scherpf, C. Schwarz, L. T. Scharf, J.-A. Zur, A. Helbig, V. H. Gessner, *Angew. Chem.* **2018**, *130*, 13041.
[19] P. Pracht, F. Bohle, S. Grimme, *Phys. Chem. Chem. Phys.* **2020**, *22*, 7169.
[20] Jmol: an open-source Java viewer for chemical structures in 3D. http://www.jmol.org/
[21] a) A. D. Becke, *J. Chem. Phys.* **1993**, *98*, 5648; b) C. Lee, W. Yang, R. G. Parr, *Phys. Rev. B* **1988**, *37*, 785; c) S. H. Vosko, L. Wilk, M. Nusair, *Can. J. Phys.* **1980**, *58*, 1200; d) P. J. Stephens, F. J. Devlin, C. F. Chabalowski, M. J. Frisch, *J. Phys. Chem*. **1994**, *98*, 11623.
[22] a) A. D. McLean, G. S. Chandler, *J. Chem. Phys.***1980**, *72*, 5639; b) R. Krishnan, J. S. Binkley, R. Seeger, J. A. Pople, *J. Chem. Phys.***1980**, *72*, 650.
[23] a) S. Grimme, J. Antony, S. Ehrlich, H. Krieg, *J. Chem. Phys*. **2010**, *132*, 154104; b) S. Grimme, S. Ehrlich, L. Goerigk, *J. Comput. Chem*. **2011**, *32*, 1456.
[24] L. E. Chirlian, M. M. Francl, *J. Comp. Chem*. **1987**, *8*, 894.
[25] T. Scherpf, C. Schwarz, L. T. Scharf, J.-A. Zur, A. Helbig, V. H. Gessner, *Angew. Chem.* **2018**, *130*, 13041.
[26] C. Adamo, V. Barone, *J. Chem. Phys.* **1999**, *110*, 6158.
[27] Tolman, C.A. *Chem. Rev.* **1977**, *77*, *3*, 313-348
[28] a) Jang, Y. H.; Goddard III, W. A.; Noyes, K. T.; Sowers, L. C.; Hwang, S.; Chung, D. S. *Chem. Res. Toxicol.* **2002**, *15*, 1023 b) Topol, I. A.; Tawa, G. J.; Burt, S. K.; Rashin, A. A. *J. Phys. Chem.A* **1997**, *101*, 10075.
